# Androgen receptor pathway inhibitors and taxanes in metastatic prostate cancer: an outcome-adaptive randomized platform trial

**DOI:** 10.1038/s41591-024-03204-2

**Published:** 2024-08-20

**Authors:** Bram De Laere, Alessio Crippa, Andrea Discacciati, Berit Larsson, Maria Persson, Susanne Johansson, Sanne D’hondt, Rebecka Bergström, Venkatesh Chellappa, Markus Mayrhofer, Mahsan Banijamali, Anastasijia Kotsalaynen, Céline Schelstraete, Jan Pieter Vanwelkenhuyzen, Marie Hjälm-Eriksson, Linn Pettersson, Anders Ullén, Nicolaas Lumen, Gunilla Enblad, Camilla Thellenberg Karlsson, Elin Jänes, Johan Sandzén, Peter Schatteman, Maria Nyre Vigmostad, Martha Olsson, Christophe Ghysel, Brieuc Sautois, Wendy De Roock, Siska Van Bruwaene, Mats Anden, Ingrida Verbiene, Daan De Maeseneer, Els Everaert, Jochen Darras, Bjørg Y. Aksnessether, Daisy Luyten, Michiel Strijbos, Ashkan Mortezavi, Jan Oldenburg, Piet Ost, Martin Eklund, Henrik Grönberg, Johan Lindberg

**Affiliations:** 1 Department of Medical Epidemiology and Biostatistics, Karolinska Institutet, Stockholm, Sweden; 2 Department of Human Structure and Repair, Ghent University, Ghent, Belgium; 3 Health, Innovation and Research Institute (Clinical Trial Unit), University Hospital Ghent, Ghent, Belgium; 4 National Bioinformatics Infrastructure Sweden, Science for Life Laboratory, Department of Cell and Molecular Biology, Uppsala University, Uppsala, Sweden; 5 Department of Urology, University Hospital Ghent, Ghent, Belgium; 6 Department of Oncology, Capio Saint Göran’s Hospital, Stockholm, Sweden; 7 Department of Oncology, Länssjukhuset Ryhov, Jönköping, Sweden; 8 Department of Oncology, Karolinska University Hospital, Stockholm, Sweden; 9 Department of Oncology, Uppsala University Hospital, Uppsala, Sweden; 10 Department of Oncology, University Hospital of Umeå, Umeå, Sweden; 11 Department of Oncology, Sundsvalls sjukhus, Sundsvall, Sweden; 12 Department of Oncology, Centralsjukhuset Karlstad, Karlstad, Sweden; 13 Department of Urology, Onze Lieve Vrouwziekenhuis, Aalst, Belgium; 14 Department of Oncology, Stavanger University Hospital, Stavanger, Norway; 15 Department of Oncology, Centrallasarettet Växjö, Växjö, Sweden; 16 Department of Urology, AZ Sint Jan Brugge AV, Bruges, Belgium; 17 Department of Oncology, CHU de Liège - site Sart Tilman, Liège, Belgium; 18 Department of Oncology, Ziekenhuis Oost- Limburg, Genk, Belgium; 19 Department of Urology, AZ Groeninge, Kortrijk, Belgium; 20 Department of Oncology, Länssjukhuset i Kalmar, Kalmar, Sweden; 21 Department of Oncology, Falu lasarett, Falu, Sweden; 22 Department of Oncology, AZ Sint-Lucas, Bruges, Belgium; 23 Department of Oncology, Vitaz campus Sint-Niklaas Lodewijk, Sint-Niklaas, Belgium; 24 Department of Urology, AZ Damiaan, Oostende, Belgium; 25 Department of Oncology, Ålesund Hospital, Ålesund, Norway; 26 Department of Oncology, Virga Jessa, Hasselt, Belgium; 27 Department of Oncology, GZA Sint-Augustinus, Antwerpen, Belgium; 28 Department of Urology, Universitätsspital Basel, Basel, Switzerland; 29 Department of Urology, Universitätsspital Zürich, Zürich, Switzerland; 30 Department of Oncology, Akershus University Hospital, Nordbyhagen, Norway; 31 Department of Radiation Oncology, GZA Sint-Augustinus, Antwerpen, Belgium; 32 Prostatacancer Centrum, Capio S:t Görans Sjukhus, Stockholm, Sweden; 33 Department of Medical Epidemiology and Biostatistics, Science for Life Laboratory, Karolinska Institutet, Stockholm, Sweden

**Keywords:** Predictive markers, Tumour biomarkers, Randomized controlled trials, Prostate cancer

## Abstract

ProBio is the first outcome-adaptive platform trial in prostate cancer utilizing a Bayesian framework to evaluate efficacy within predefined biomarker signatures across systemic treatments. Prospective circulating tumor DNA and germline DNA analysis was performed in patients with metastatic castration-resistant prostate cancer before randomization to androgen receptor pathway inhibitors (ARPIs), taxanes or a physician’s choice control arm. The primary endpoint was the time to no longer clinically benefitting (NLCB). Secondary endpoints included overall survival and (serious) adverse events. Upon reaching the time to NLCB, patients could be re-randomized. The primary endpoint was met after 218 randomizations. ARPIs demonstrated ~50% longer time to NLCB compared to taxanes (median, 11.1 versus 6.9 months) and the physician’s choice arm (median, 11.1 versus 7.4 months) in the biomarker-unselected or ‘all’ patient population. ARPIs demonstrated longer overall survival (median, 38.7 versus 21.7 and 21.8 months for taxanes and physician’s choice, respectively). Biomarker signature findings suggest that the largest increase in time to NLCB was observed in *AR* (single-nucleotide variant/genomic structural rearrangement)-negative and *TP53* wild-type patients and *TMPRSS2–ERG* fusion-positive patients, whereas no difference between ARPIs and taxanes was observed in *TP53*-altered patients. In summary, ARPIs outperform taxanes and physician’s choice treatment in patients with metastatic castration-resistant prostate cancer with detectable circulating tumor DNA. ClinicalTrials.gov registration: NCT03903835.

## Abstract (web summary)

In a biomarker-driven, outcome-adaptive platform trial for patients with metastatic castration-resistant prostate cancer, androgen receptor pathway inhibitors showed longer survival with respect to taxanes and physician’s choice treatment.

## Main

Metastatic castration-resistant prostate cancer (mCRPC) is a genetically and clinically heterogeneous disease that can be treated with different classes of systemic agents, such as androgen receptor pathway inhibitors (ARPIs) or taxanes. Retrospective analyses suggest that different tumor genotypes yield varying therapeutic benefits^1^. Although several prognostic biomarkers have been identified, the number of biomarkers predictive of therapeutic and clinical benefit remains small.

One reason for this is that traditional clinical trials have not been biomarker-driven and have not focused on prospectively investigating patient subgroups to identify patients who benefit more from a specific treatment^2^. The advent of precision oncology, where treatment selection is optimized based on biomarkers, provides an opportunity to tailor treatments based on the molecular characteristics of the cancer but will inevitably lead to testing of a large number of hypotheses to evaluate combinations of treatments and biomarkers^3^. While traditional two-armed randomized trials have focused on demonstrating a treatment effect in an all-comer population, precision oncology necessitates development of clinical trials that can accommodate this consequential multiplicative hypothesis testing scenario. In addition, precision oncology trials require prospective biomarker profiling that ideally is minimally invasive, representative and scalable, and provides real-time analysis^4^.

To meet these challenges, we have developed the Prostate Biomarkers (ProBio) platform trial. In contrast to traditional trials, platform trials provide an effective means to evaluate and screen for novel biomarker–treatment combinations^5,6^. ProBio aims to infer treatment predictiveness or, in other words, to identify subsets of patients who benefit from a particular treatment class. In ProBio, treatment benefit is not approached as a binary variable (that is, benefit versus no benefit), but as a continuum (that is, a degree or extent of therapeutic benefit). Such differential treatment effects do not necessarily imply that a treatment will be effective exclusively in one subset of patients, but rather that its efficacy may vary across different patient groups. Thus, ProBio aims to elucidate whether certain biomarker signatures could help identify subsets of patients who might derive greater or longer clinical benefit from treatment. In ProBio, such treatment effects are measured as the relative impact (that is, an increase or decrease) on the expected survival time, referred to as the survival time ratio (STR; Methods).

ProBio uses a number of design elements to maximize the information gained from each patient entering the trial in order to rapidly sieve through multiple hypotheses. Specifically, similarly to for example the I-SPY breast cancer trial that shares the same objective, it uses a common control arm for all comparative contrasts together with outcome-adaptive randomization. Outcome-adaptive randomization, or the updating of randomization probabilities over time based on observed outcomes, aims to allocate more patients to investigational biomarker–treatment combinations that are more effective, and thus works as a screening device to focus enrollment on the combinations that are most probable to be successful. For trial participants, this means that they have higher probability to be randomized to agents that are performing well for patients with a similar biomarker profile. In addition, ProBio allows for re-randomizations of patients within the investigational arms when the primary no longer clinically benefitting (NLCB) endpoint is reached. This increases the amount of information provided by each patient enrolled in the trial. Upon NLCB, control arm patients remain and start a new line of therapy within the control arm.

ProBio uses predefined genetic biomarkers (Supplementary Fig. 1), assessed through synchronous circulating tumor DNA (ctDNA) and germline DNA analysis^7^, to randomize patients and prospectively compare the efficacy of different treatment classes to physician’s choice of treatment. ctDNA allows for representative analysis of the cancer genome in a clinically acceptable time frame through a simple blood sample.

**Fig. 1:**
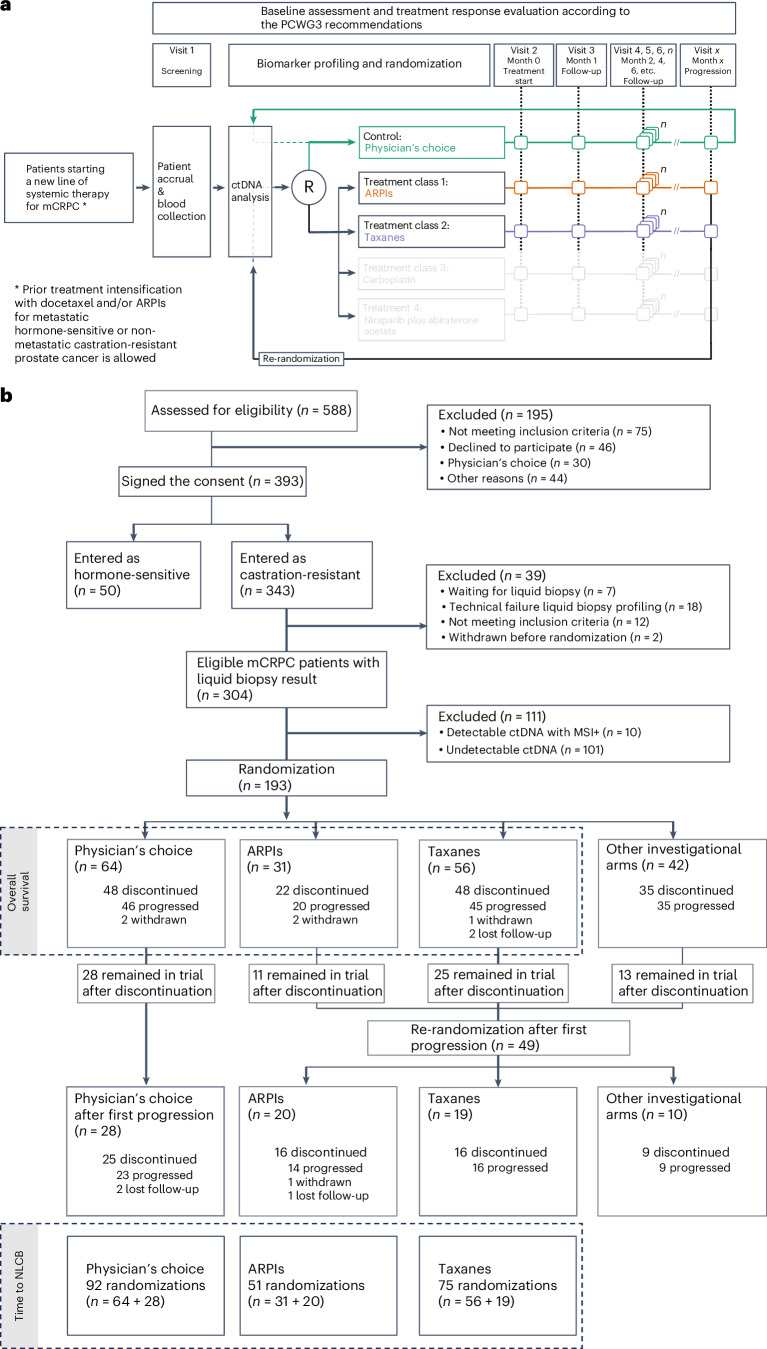
Trial design. **a**, The ProBio platform design, including control and investigational arms, with adaptive randomization after screening and ctDNA analysis. Upon reaching progression, patients were re-randomized. Follow-up and treatment response evaluation followed the PCWG3 recommendations. Investigational arms in transparent gray denote other investigational arms to which patients could have been randomized, which do not fall within the scope of the current paper. **b**, The CONSORT diagram detailing patient screening, randomization and follow-up. Inclusion and randomization updated until 25 November 2022, with results for ARPIs and taxanes. Data on other investigational arms pending. As of this date, 7 patients awaited liquid biopsy results and had not been randomized. An extra 4 months of follow-up were allowed until March 2023 for 193 randomized patients. Of 129 patients in the investigational arms, 49 reached the progression endpoint, remained in the trial, and were re-randomized. In the physician’s choice group, 28 of 64 patients continued with new standard-of-care treatments. Follow-up included all-cause mortality from electronic health records. MSI+, microsatellite instability positive.

A knowledge gap in treatment of men with mCRPC is that randomized data to support which of the two main treatment classes, that is, taxanes and ARPIs, provide superior outcomes or to guide treatment decisions are limited and restricted to selected patients with mCRPC who have a poor prognosis^8^. Here we present the first results of ProBio upon reaching the stopping rule for graduation, that is, a probability over 85% that an investigational biomarker–treatment combination is superior compared to a physician’s choice of treatment within that biomarker or patient group. The primary (that is, time to NLCB) and secondary (that is, overall survival) outcomes are compared in patients with first-line and second-line mCRPC, who were randomized to the investigational ARPIs and taxane-based chemotherapy arms, or the physician’s choice of treatment control arm, across five different biomarker signatures or groups: (1) any biomarker subgroup combination (that is, biomarker-unselected or ‘all’ patients); (2) no single-nucleotide variants (SNVs) or genomic structural rearrangements (GSRs) in the *AR* gene and no alterations in the *TP53* gene (that is, *AR* (SNV/GSR) negative and *TP53* wild type); (3) homologous recombination deficiency (that is, deactivating somatic or high-impact germline alteration in DNA repair genes); (4) alterations in *TP53* (that is, *TP53* altered); and (5) presence of the transmembrane protease, serine 2–ETS-related gene (*TMPRSS2–ERG*) fusion; Fig. 1 and Supplementary Fig. 1). Additional planned secondary endpoints not reported here are health-related quality-of-life outcomes and cost-effectiveness of ARPIs versus taxanes in mCRPC.

## Results

### Patients

Between February 2019 and November 2022, 343 patients with mCRPC were enrolled at 24 sites across three countries (Sweden, Belgium and Norway). Thirty-nine patients were excluded if they did not meet inclusion criteria, were not yet randomized, were withdrawn before randomization, or because of a technical failure during liquid biopsy profiling. ctDNA was detected in 203/304 (66.8%) evaluable patients, of whom 10 patients were excluded due to detection of microsatellite instability. The ctDNA fraction was estimated and the biomarker subgroup combinations were determined in eligible men with detectable ctDNA (*n* = 193) and subsequently used for randomization (Fig. 1). The ctDNA fraction levels were comparable across the different treatment arms (Table 1). At first randomization, 39% of patients were *AR* (SNV/GSR) negative and *TP53* wild type, 12% were homologous recombination deficient, 45% carried *TP53* alterations and 31% were positive for the *TMPRSS2–ERG* gene fusion.

**Table 1:** Baseline characteristics of the randomized patients stratified by randomization round

	Randomization round: 1 (*N* = 151)			Randomization round: 2 (*N* = 67)		
Characteristic	Physician’s choice (*N* = 64)	ARPIs (*N* = 31)	Taxanes (*N* = 56)	Physician’s choice (*N* = 28)	ARPIs (*N* = 20)	Taxanes (*N* = 19)
Age at study entry (median, range), years	71 (68, 76)	71 (65, 75)	69 (64, 73)	71 (68, 75)	69 (66, 73)	68 (66, 72)
ECOG status score of 0 or 1 at study entry, no. (%)	59 (92%)	30 (97%)	52 (93%)	27 (96%)	17 (85%)	18 (95%)
Type of CRPC progression at study entry (PCWG3), *n* (%)						
PSA alone	10 (16%)	2 (6.5%)	2 (3.6%)	4 (14%)	1 (5.0%)	0 (0%)
Bone ± nodes by location	45 (70%)	18 (58%)	41 (73%)	17 (61%)	14 (70%)	12 (63%)
Nodes by location only	3 (4.7%)	5 (16%)	6 (11%)	3 (11%)	2 (10%)	3 (16%)
Viscera (±other sites)	6 (9.4%)	6 (19%)	7 (13%)	4 (14%)	3 (15%)	4 (21%)
Location metastases at study entry, no. (%)						
LN only	2 (3.1%)	4 (13%)	4 (7.1%)	2 (7.1%)	2 (10%)	2 (11%)
Bone ± LN	54 (84%)	18 (58%)	46 (82%)	21 (75%)	15 (75%)	13 (68%)
Viscera (±other sites)	6 (9.4%)	7 (23%)	6 (11%)	4 (14%)	3 (15%)	4 (21%)
Undetectable metastasis	2 (3.1%)	2 (6.5%)	0 (0%)	1 (3.6%)	0 (0%)	0 (0%)
Metastatic disease (M1) at diagnosis	37 (58%)	15 (48%)	33 (59%)	12 (43%)	14 (70%)	10 (53%)
Previous systemic therapy for mHSPC/nmCRPC, *n* (%)						
ADT monotherapy	28 (44%)	13 (42%)	24 (43%)	15 (54%)	8 (40%)	12 (63%)
ADT + docetaxel	24 (38%)	18 (58%)	19 (34%)	11 (39%)	10 (50%)	3 (16%)
ADT + ARPI	12 (19%)	0 (0%)	13 (23%)	2 (7.1%)	2 (10%)	4 (21%)
Treatment line for mCRPC at randomization, *n* (%)						
1st line	52 (81%)	28 (90%)	42 (75%)	0 (0%)	0 (0%)	0 (0%)
2nd line	12 (19%)	3 (9.7%)	13 (23%)	22 (79%)	15 (75%)	13 (68%)
3rd line	0 (0%)	0 (0%)	1 (1.8%)	6 (21%)	4 (20%)	6 (32%)
4th line	0 (0%)	0 (0%)	0 (0%)	0 (0%)	1 (5.0%)	0 (0%)
Prior ARPI treatment, *n* (%)						
None	42 (66%)	31 (100%)	32 (57%)	9 (32%)	15 (75%)	3 (16%)
One regimen	22 (34%)	0 (0%)	24 (43%)	19 (68%)	5 (25%)	16 (84%)
Prior taxane treatment, *n* (%)						
None	38 (59%)	11 (35%)	36 (64%)	5 (18%)	2 (10%)	11 (58%)
One regimen	26 (41%)	19 (61%)	18 (32%)	18 (64%)	11 (55%)	7 (37%)
More than one regimen	0 (0%)	1 (3.2%)	2 (3.6%)	5 (18%)	7 (35%)	1 (5.3%)
PSA level at study entry (median, range), ng ml^−1^	27 (12, 60)	18 (6, 61)	18 (10, 54)	36 (14, 61)	25 (14, 50)	10 (6, 24)
ctDNA fraction at study entry, *n* (%)						
Low (<5%)	20 (31%)	9 (29%)	13 (23%)	8 (29%)	3 (15%)	3 (16%)
Medium (5–40%)	31 (48%)	16 (52%)	33 (59%)	13 (46%)	11 (55%)	13 (68%)
High (≥40%)	13 (20%)	6 (19%)	10 (18%)	7 (25%)	6 (30%)	3 (16%)
Biomarker signatures, *n* (%)						
*AR* (SNV/GSR) negative and *TP53* wild type	27 (42%)	15 (48%)	17 (30%)	12 (43%)	4 (20%)	8 (42%)
Homologous recombination deficiency	10 (16%)	2 (6.5%)	6 (11%)	6 (21%)	1 (5.0%)	6 (32%)
*TP53* altered	27 (42%)	13 (42%)	28 (50%)	12 (43%)	11 (55%)	7 (37%)
*TMPRSS2–ERG* fusion positive	16 (25%)	10 (32%)	20 (36%)	8 (29%)	7 (35%)	2 (11%)

Information regarding treatment line and exposure to ARPIs and taxanes is updated up until the time of randomization. Continuous variables are shown as medians and interquartile ranges; categorical and binary variables are shown as numbers and percentages. ECOG, Eastern Cooperative Oncology Group performance status; PCWG3, Prostate Cancer Working Group 3; LN, lymph node; mHSPC, metastatic hormone-sensitive prostate cancer; nmCRPC, non-metastatic castration-resistant prostate cancer; AR*,* androgen receptor.

Sixty-four patients were assigned to the physician’s choice control arm. Following disease progression, 28 patients received a subsequent line of treatment, for a total of 92 control arm randomizations available for primary outcome analysis (that is, time to NLCB). In the control arm, the treating physician chose an ARPI (37/92, 40%), a taxane (48/92, 52%) or other systemic agents (7/92, 8%; Supplementary Table 1). Concurrently, 129 patients were assigned to the investigational arms, of which 31 were assigned to ARPI and 56 were assigned to taxanes. Forty-nine patients were re-randomized upon progression, of which 20 were assigned to ARPIs and 19 were assigned to taxanes. Thus, a total of 51 and 75 randomizations were available for primary outcome analysis in the investigational ARPI and taxane arms, respectively. For the secondary outcome measure (that is, overall survival), analyses were restricted to patients from first randomization (Fig. 1).

Clinical patient characteristics at baseline were evenly distributed between the physician’s choice control and the investigational arms with comparable utilization rates of androgen deprivation therapy (ADT) monotherapy and ADT intensification (that is, either with docetaxel or ARPIs) before metastatic castration-resistant disease (Table 1). The lines of systemic therapy that were initiated for mCRPC after randomization were comparable across all treatment arms. The type of progressive disease at trial entry was comparable with bone (with or without nodal disease) progression most commonly observed in 104/151 (68.9%) patients. However, the systemic therapy class(es) to which the patients had been exposed before ProBio differed at randomization. There were differences across the arms in the patients’ prior exposure to ARPIs or taxanes since treatment class rechallenge was per protocol not allowed in the investigational ARPI arm (Table 1). More prior taxane exposure was observed in the ARPI arm, and vice versa more prior ARPI exposure was observed in the taxanes arm. Patients randomized to the ARPI arm were typically ARPI-naive (46/51 (90.2%) randomizations), whereas 28/75 (37.3%) patients randomized to the taxanes arm had previously received a treatment with taxanes.

At the time of analysis, the progression component event(s) to deem a patient as NLCB typically encompassed a composite of prostate-specific antigen (PSA), radiologic or clinical progression, and were similar across treatment arms (Supplementary Table 2).

### Primary outcome

**Fig. 2:**
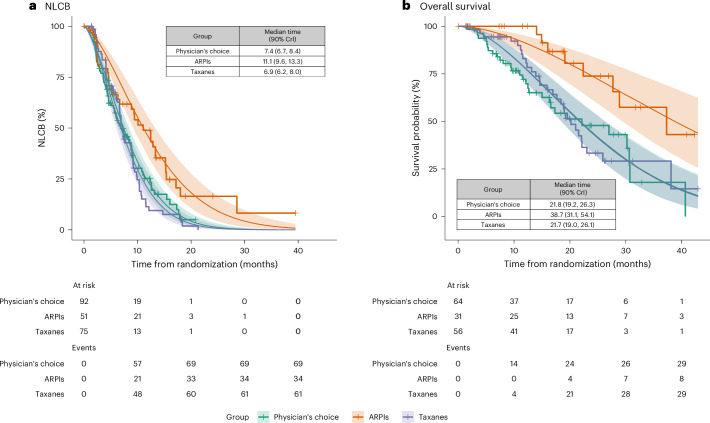
Time to NLCB and overall survival. **a**,**b**, Posterior survival curves (smooth) and Kaplan–Meier (stepped) estimates for the time to NLCB (**a**) and overall survival (**b**) in all patients (that is, biomarker-unselected or any biomarker subgroup combination), grouped by randomization. Tick marks on Kaplan–Meier curves denote censored patients. Weibull accelerated failure time models were used for survival estimates. Colored lines depict median posterior distribution, and shaded areas show 90% CrI. Insets: Patients at risk, cumulative events and estimated median survival times (in months) with 90% CrI.

Randomization probabilities for investigational and control arms adapted over time (Supplementary Fig. 2). The prespecified stopping rules for graduation and primary outcome evaluation were met after 218 randomizations across the physician’s choice and investigational treatment arms. ARPIs reached the prespecified threshold of superiority in the biomarker-unselected ‘all’ patients group (Table 2 and Supplementary Fig. 3). The decision to publish the results was based on meeting the graduation criteria outlined in the protocol (see stopping rules for graduation and futility in the Methods and Supplementary Information), along with the recommendations provided by the data and safety monitoring board. These criteria and recommendations were carefully reviewed and followed, leading to the decision to publish the findings. The STR for the time to NLCB for ARPIs was 1.50 (90% credible intervals (CrI) 1.20, 1.86) compared to the physician’s choice (median 11.1 versus 7.4 months) and 1.60 (90% CrI 1.28, 2.01) compared to taxanes (median 11.1 versus 6.9 months; Fig. 2, Table 2 and Supplementary Fig. 3). The STR for the time to NLCB for taxanes was 0.94 (90% CrI 0.78, 1.12) compared to the physician’s choice (median 6.0 versus 7.4 months).

**Table 2:** Efficacy results for the time to NLCB (left) and overall survival (right) in the five predefined biomarker signatures or groups

	NLCB				Overall survival			
Biomarker signature or group	Arm (events/*N*)	Reference (events/*N*)	PPS	STR (90% CrI)	Arm (events/*N*)	Reference (events/*N*)	PPS	STR (90% CrI)
All patients	ARPIs (34/51)	Physician’s choice (69/92)	1.00	1.50 (1.20, 1.86)	ARPIs (8/31)	Physician’s choice (30/64)	1.00	1.77 (1.29, 2.51)
	ARPIs (34/51)	Taxanes (61/75)	1.00	1.60 (1.28, 2.01)	ARPIs (8/31)	Taxanes (29/56)	1.00	1.78 (1.28, 2.61)
	Taxanes (61/75)	Physician’s choice (69/92)	0.27	0.94 (0.78, 1.12)	Taxanes (29/56)	Physician’s choice (30/64)	0.48	0.99 (0.78, 1.26)
*AR* (SNV/GSR) negative and *TP53* wild-type	ARPIs (11/19)	Physician’s choice (31/42)	1.00	1.76 (1.26, 2.51)	ARPIs (4/15)	Physician’s choice (11/27)	0.90	1.45 (0.90, 2.40)
	ARPIs (11/19)	Taxanes (15/22)	0.99	1.74 (1.15, 2.62)	ARPIs (4/15)	Taxanes (4/17)	0.64	1.14 (0.61, 2.11)
	Taxanes (15/22)	Physician’s choice (31/42)	0.53	1.01 (0.73, 1.42)	Taxanes (4/17)	Physician’s choice (11/27)	0.79	1.27 (0.78, 2.15)
Homologous recombination deficiency	ARPIs (1/4)	Physician’s choice (13/16)	0.96	1.89 (1.06, 3.53)	ARPIs (0/2)	Physician’s choice (6/10)	0.63	1.15 (0.52, 2.57)
	ARPIs (1/4)	Taxanes (9/12)	0.92	1.83 (0.88, 3.89)	ARPIs (0/2)	Taxanes (0/6)	0.39	0.82 (0.27, 2.46)
	Taxanes (9/12)	Physician’s choice (13/16)	0.55	1.04 (0.62, 1.72)	Taxanes (0/6)	Physician’s choice (6/10)	0.75	1.42 (0.62, 3.20)
*TP53* altered	ARPIs (19/24)	Physician’s choice (30/37)	0.78	1.14 (0.88, 1.48)	ARPIs (4/13)	Physician’s choice (15/27)	0.96	1.60 (1.03, 2.61)
	ARPIs (19/24)	Taxanes (31/36)	0.63	1.05 (0.81, 1.38)	ARPIs (4/13)	Taxanes (18/28)	0.98	1.79 (1.13, 3.03)
	Taxanes (31/36)	Physician’s choice (30/37)	0.69	1.07 (0.84, 1.37)	Taxanes (18/28)	Physician’s choice (15/27)	0.28	0.89 (0.63, 1.24)
*TMPRSS2–ERG* fusion positive	ARPIs (9/16)	Physician’s choice (20/24)	0.99	1.80 (1.21, 2.64)	ARPIs (2/10)	Physician’s choice (12/16)	0.99	2.01 (1.26, 3.38)
	ARPIs (9/16)	Taxanes (17/22)	0.99	1.99 (1.30, 3.02)	ARPIs (2/10)	Taxanes (12/20)	0.99	2.02 (1.23, 3.55)
	Taxanes (17/22)	Physician’s choice (20/24)	0.31	0.90 (0.63, 1.28)	Taxanes (12/20)	Physician’s choice (12/16)	0.49	0.99 (0.68, 1.46)

Efficacy assessed using STR comparing investigational arms (ARPIs and taxanes) to physician’s choice (control group) and AR pathway to taxanes across the five predefined biomarker signatures or groups: (1) any biomarker subgroup combination (that is, biomarker-unselected or ‘all’ patients); (2) no SNV or GSRs in the *AR* gene and no alterations in the *TP53* gene (that is, *AR* (SNV/GSR) negative and *TP53* wild type); (3) homologous recombination deficiency (that is, deactivating somatic or high-impact germline alteration in DNA repair genes); (4) alterations in *TP53* (that is, *TP53* altered); and (5) presence of the *TMPRSS2–ERG* fusion (that is, *TMPRSS2–ERG* fusion positive). Posterior STR distributions summarized with medians and 90% CrI. Forest plot visualizations for the STRs for NLCB and overall survival are available in Supplementary Fig. 3. Posterior probability of superiority (PPS) calculated as STR exceeding one, guiding early termination decisions. Additional information on number of events and total number of randomizations (events/*N*) provided for each comparison.

The longer time to NLCB for patients treated with ARPIs compared to physician’s choice and to taxanes was primarily observed in *AR* (SNV/GSR) negative and *TP53* wild-type patients and in *TMPRSS2–ERG* gene fusion-positive patients. No difference in the time to NLCB between ARPIs, taxanes or physician’s choice was observed for *TP53*-altered patients. The time to NLCB was assessed for homologous recombination-deficient patients, albeit with marked uncertainty due to few randomizations to ARPIs for patients in this biomarker subgroup (Table 2 and Supplementary Figs. 3 and 4). Results were robust in sensitivity analyses restricted to patients starting first-line treatment for mCRPC, patients starting first-line treatment for mCRPC who previously only received ADT monotherapy for hormone-sensitive disease and patients who did not undergo a drug class rechallenge and thus had not previously received the drug class to which they were randomized (Supplementary Table 3).

**Fig. 3:**
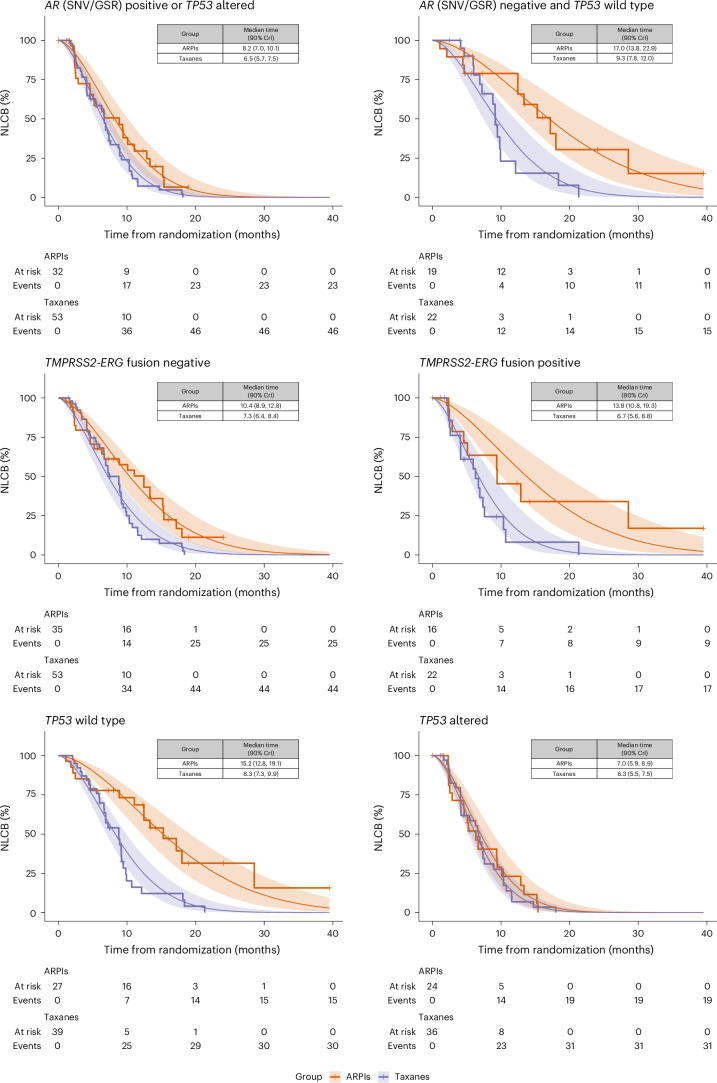
Posterior survival curves and Kaplan–Meier estimates by therapy arm and biomarker signature status (positive versus negative). Posterior survival curves (smooth) and Kaplan–Meier estimates (step function) for the time to NLCB with ARPIs and taxanes. Left: Patients negative for a specific biomarker signature. Right: Patients positive for the respective biomarker signature. Survival curves were estimated using Weibull accelerated failure time survival models with an interaction term, enabling the assessment of differential treatment effects. The smoothed colored lines represent medians of the posterior distribution, while shaded areas depict corresponding 90% CrI. Top: *AR* (SNV/GSR) positive or *TP53* altered versus *AR* (SNV/GSR) negative and *TP53* wild type. Middle: *TMPRSS2–ERG* fusion negative versus *TMPRSS2–ERG* positive. Bottom: *TP53* wild type versus *TP53* altered. Homologous recombination deficiency was left out due to very few randomizations to ARPIs; preliminary results are available in Supplementary Fig. 5 and Table 3.

We observed differential treatment effects on the time to NLCB, which suggests a treatment-predictive value of the prespecified biomarker signatures (Supplementary Table 4 and Supplementary Fig. 5). Comparing the STRs, the effect of ARPIs versus taxanes was 44% (STR ratio 1.44, 90% CrI 1.05, 1.95) higher in *AR* (SNV/GSR)-negative and *TP53* wild-type patients compared to patients with alterations in these genes. Similarly, the effect was 42% (STR ratio 1.42, 90% CrI 1.03, 1.97) higher in *TMPRSS2–ERG* gene fusion-positive patients compared to *TMPRSS2–ERG* gene fusion-negative patients. Conversely, the STR decreased by 39% (STR ratio 0.61, 90% CrI 0.46, 0.80) in *TP53*-altered patients compared to *TP53* wild-type patients (Fig. 3). For completeness, differential treatment effects were also assessed for homologous recombination deficiency, albeit with marked uncertainty due to few randomizations to ARPIs (Supplementary Table 4 and Supplementary Fig. 5).

### Secondary outcome

The STR for overall survival in the biomarker-unselected ‘all’ patients group was 1.77 (90% CrI 1.29, 2.51) for ARPIs compared to physician’s choice (median 38.7 versus 21.8 months) and 1.78 (90% CrI 1.28, 2.61) compared to the taxane arm (median 38.7 versus median 21.7 months; Fig. 2 and Table 2). The STR for overall survival for taxanes was 0.99 (90% CrI 0.78, 1.26) compared to the physician’s choice (median 21.7 versus 21.8 months). The STR increase for ARPIs was observed in *TMPRSS2–ERG* fusion-positive patients, and in *TP53*-altered patients, whereas no difference was noted at the time of analysis for *AR* (SNV/GSR)-negative and *TP53* wild-type patients (Table 2 and Supplementary Figs. 3 and 4).

### Safety

The safety profile comparison between ARPIs and taxanes, both within the physician’s choice control and investigational arms, indicates notable differences in adverse event (AE) and serious adverse event (SAE) rates, specific event prevalence estimates and treatment relatedness (Table 3). The overall AE rates (that is, all grades) were higher in the taxane groups compared to the ARPI groups, with at least half of them being treatment related. AEs resulting in investigational medicinal product interruption, dose adjustment or permanent discontinuation were more commonly observed in taxane-treated patients.

**Table 3:** Summary of AEs/SAEs

AE	ARPIs		ARPIs (physician’s choice control)		Taxanes		Taxanes (physician’s choice control)	
	(*N* = 51)		(*N* = 37)		(*N* = 75)		(*N* = 48)	
Any AE	37	72.5%	28	75.7%	64	85.3%	45	93.8%
Treatment-related AE	26	51.0%	19	51.4%	50	66.7%	38	79.2%
Any SAE/AE resulting in IMP interruption	4	7.8%	6	16.2%	18	24.0%	8	16.7%
An SAE/AE resulting in IMP (dose) adjustment	2	3.9%	2	5.4%	12	16.0%	8	16.7%
Any SAE/AE resulting in permament discontinuation of IMP	5	9.8%	6	16.2%	14	18.7%	7	14.6%
Most common AE (all grades, occurring in ≥5% of patients)								
Fatigue	7	13.7%	9	24.3%	25	33.3%	17	35.4%
Back pain	9	17.6%	3	8.1%	19	25.3%	9	18.8%
Anemia	6	11.8%	2	5.4%	9	12.0%	9	18.8%
Pain (hip)	5	9.8%	3	8.1%	6	8.0%	6	12.5%
Nausea	3	5.9%	1	2.7%	4	5.3%	8	16.7%
Diarrhea	1	2.0%	0	0.0%	8	10.7%	7	14.6%
Dysgeusia	0	0.0%	1	2.7%	9	12.0%	5	10.4%
Dyspnea	3	5.9%	2	5.4%	8	10.7%	2	4.2%
Neuropathy/peripheral sensory neuropathy	2	3.9%	2	5.4%	14	18.7%	8	16.7%
Constipation	1	2.0%	1	2.7%	6	8.0%	5	10.4%
Fever	2	3.9%	3	8.1%	5	6.7%	4	8.3%
Pain (leg/extremities)	2	3.9%	1	2.7%	6	8.0%	3	6.3%
Thromboembolic event	0	0.0%	0	0.0%	7	9.3%	6	12.5%
Neutropenia	0	0.0%	0	0.0%	6	8.0%	6	12.5%
Weight loss	1	2.0%	3	8.1%	4	5.3%	5	10.4%
SAEs	16	31.4%	13	35.1%	26	34.7%	21	43.8%
Treatment-related SAEs	5	9.8%	6	16.2%	17	22.7%	18	37.5%
Number of SAEs								
1	10	19.6%	9	24.3%	14	18.7%	10	20.8%
2	4	7.8%	2	5.4%	10	13.3%	6	12.5%
≥3	2	3.9%	2	5.4%	2	2.7%	5	10.4%
SAE system organ class								
Infections and infestations	5	9.8%	4	10.8%	8	10.7%	8	16.7%
Gastrointestinal disorders	4	7.8%	2	5.4%	1	1.3%	4	8.3%
Vascular disorders	2	3.9%	0	0.0%	6	8.0%	6	12.5%
Cardiac disorders	2	3.9%	3	8.1%	4	5.3%	3	6.3%
Musculoskeletal and connective tissue disorders	1	2.0%	0	0.0%	2	2.7%	4	8.3%
General disorders and administrations site conditions	2	3.9%	1	2.7%	3	4.0%	1	2.1%
Nervous system disorders	1	2.0%	3	8.1%	2	2.7%	1	2.1%
Blood and lymphatic disorders	1	2.0%	0	0.0%	4	5.3%	1	2.1%
Renal and urinary disorders	0	0.0%	3	8.1%	0	0.0%	0	0.0%
Injury, poisoning and procedural complications	2	3.9%	1	2.7%	1	1.3%	1	2.1%
Metabolism and nutrition disorders	1	2.0%	0	0.0%	1	1.3%	1	2.1%
Respiratory, thoracic and mediastinal disorders	0	0.0%	1	2.7%	1	1.3%	2	4.2%
Grade 5 AEs	0	0.0%	0	0.0%	2^a^	2.7%	1^a^	2.1%

The table provides a descriptive comparison of the occurrence of the most common SAEs/AEs reported in patients who received an ARPI or taxanes, separately for those randomized to physician’s choice (control group) and those in the experimental arm. IMP, investigational medicinal product.

^a^None were considered treatment related.

Among common AEs (that is, any grade), occurring in ≥5% of both ARPI-treated and taxane-treated patients, varying patterns were noted. Fatigue, back pain and anemia were more prevalent in the taxane groups compared to the ARPI groups. Rates of patients experiencing pain in the hip, dyspnea, fever or weight loss were similar across groups. Higher rates of nausea, diarrhea, dysgeusia, peripheral sensory and general neuropathy, and constipation were observed in taxane-treated patients. Thromboembolic events and neutropenia were observed in the taxane groups, but were not observed in the ARPI-treated patients.

Although the overall SAE rates were comparable in the taxane and ARPI groups, these were more treatment related in taxane-treated patients. Additionally, a higher rate of patients experiencing more than two SAEs was reported in the taxane groups. After system organ class classifications, the most common SAEs were infections and infestations, gastrointestinal disorders and cardiovascular disorders. Serious infections, vascular, musculoskeletal and connective tissue disorders were more commonly observed in taxane-treated patients. Grade 5 AEs were rare, with two occurrences in the taxane group, and one in the taxane physician’s choice group, which were reported not to be treatment related. Overall, taxanes were associated with a higher incidence of both general and treatment-related AEs and SAEs compared to ARPIs.

## Discussion

We present the first results from ProBio, a study that has established a framework for addressing key challenges faced by clinical trials in the precision oncology era. Specifically, ProBio’s platform design, trial infrastructure and real-time liquid biopsy profiling have successfully enabled continuous and seamless prospective evaluation of systemic agents and cell-free DNA biomarkers, providing a new way to identify subsets of patients benefiting differentially from a particular treatment class.

The initial results demonstrate the superiority of ARPIs for the biomarker-unselected ‘all’ patient population compared to physician’s choice and taxanes in patients with mCRPC and detectable ctDNA. On average, patients treated with ARPIs experienced a 55% and 77% longer time to NLCB and overall survival, respectively. To our knowledge, these are the first randomized data for comparing outcomes of ARPIs versus taxanes in patients with first-line or second-line mCRPC with detectable ctDNA. Although ARPIs are considered the standard of care for treatment of metastatic prostate cancer, it should be emphasized that the abiraterone acetate and enzalutamide registration trials in docetaxel-naive mCRPC were placebo-controlled, and thus not designed to infer which treatment modality (ARPIs or taxane-based chemotherapy) would have superior outcomes in patients with mCRPC^9,10^. Randomized data on ARPIs versus cabazitaxel are limited to a single phase 2 trial in poor prognosis ARPI-naive mCRPC, which reported improved clinical benefit rates for cabazitaxel without time to progression or overall survival being statistically different between treatment classes^8^. Our evidence on ARPIs being superior over taxanes is supported by results from observational and registry-based studies^11–13^. Additionally, the STAMPEDE trial results indicated improved progression-free and failure-free survival outcomes for patients with metastatic hormone-sensitive prostate cancer treated with abiraterone acetate compared to docetaxel^14^.

We further provided randomized-controlled insights into the treatment-predictive potential of the selected biomarker signatures and proposed novel ctDNA-defined patient subpopulations with differential treatment outcomes. Specifically, our data suggest that *AR* (SNV/GSR)-negative and *TP53* wild-type patients, or *TMPRSS2–ERG* fusion-positive patients, may derive greater benefits from ARPIs compared to taxanes. The results for *AR* (SNV/GSR)-negative and *TP53* wild-type patients were consistent with prior retrospective studies that reported superior outcomes on ARPIs^15–17^. However, for *AR* (SNV/GSR)-negative and *TP53* wild-type patients, no difference in overall survival between ARPIs and taxanes was observed at the time of analysis. Longer follow-up will show whether the extended time to NLCB with ARPIs compared to taxanes for *AR* (SNV/GSR)-negative and *TP53* wild-type patients ultimately also translates into better overall survival.

Our findings suggest that *TMPRSS2–ERG* fusion-positive patients may benefit more from ARPIs compared to taxanes. ProBio initially aimed to clarify the potential predictive value of the *TMPRSS2–ERG* fusion in the context of taxane-based chemotherapy since retrospective analyses were conflicting^18–24^. Anticipating that *TMPRSS2–ERG*-positive cases would define a subpopulation with increased benefit of taxane-based chemotherapy, data from ProBio suggested that the *TMPRSS2–ERG* status may be rather more informative in the context of ARPIs. This observation aligns with previous translational research. Upon castration resistance, *TMPRSS2–ERG* expression can be restored as testosterone-independent androgen biosynthesis is increased. As the *TMPRSS2–ERG* fusion promoter is regulated by the AR, its transcriptional output may be sensitized to novel ARPIs^25,26^.

In addition, ProBio provides a biomarker-driven randomized comparison between ARPIs and taxanes in patients with mCRPC with *TP53*-altered disease. Previous studies reported poor outcomes in ARPI-treated patients with mCRPC with a *TP53* inactivation^15,16^. Whether these patients would have better outcomes if treated with a taxane was not clearly known. An exploratory subgroup analysis in patients with mCRPC who have a poor prognosis with a *TP53* defect during the ARPI versus cabazitaxel phase 2 trial was suggestive of no difference between both schedules^8^. Here, ProBio provides supporting evidence that the inferior outcomes in *TP53*-altered mCRPC are regardless of whether ARPIs or taxanes were administered. This emphasizes the need for alternative treatment strategies and echoes the previously made recommendation for careful monitoring of *TP53*-altered patients who are more likely to rapidly progress^15,16^. Our data on overall survival indicate that these patients may still benefit from ARPI, although to a lesser extent compared to patients harboring other biomarker signatures.

Some limitations need to be considered when interpreting our results. Firstly, the chosen endpoint of NLCB as the termination point for treatment may lead to bias in open-label trials. While aligned with current standard-of-care practices to decide when to change a therapy, the NLCB endpoint deviates from the commonly used radiographic progression-free survival endpoint in metastatic prostate cancer trials^27,28^, known to be associated with overall survival. Pragmatic considerations (for example, mimicking standard of care, decreasing costs and the logistics of centralized imaging review) and common discordance in clinical, imaging and PSA progression events, both with ARPIs^29,30^ and taxanes^31^, guided this choice. We recognize that such composite events can be differentially affected in different therapeutic contexts^32^. To exemplify, as ARPIs directly target AR signaling, the NLCB endpoint might have been more impacted by, for example, PSA-only progressive disease in ARPI-treated patients. However, the rates of PSA-only progressive disease, which might have driven the treating physician to deem a patient as NLCB, were overall low and comparable across the investigational treatment arms. The component events within a composite endpoint may also vary in frequency, warranting a detailed description of the different component events to infer the validity of the findings. Importantly, per PCWG3 recommendations, ProBio collects and provides the specific reasons for therapy discontinuation for NLCB reporting. The NLCB events during ProBio typically encompassed a combination of PSA, radiologic or clinical progression, with the different component events frequencies being comparable across the treatment arms (Supplementary Table 2). Results on overall survival for ARPIs in the biomarker-unselected ‘all’ patient population also supported the results on the NLCB endpoint.

Secondly, the absence of data on race and ethnicity may impact the generalizability of our findings, as these factors can affect treatment outcomes^33,34^. Next, the smaller sample size used in ProBio compared to traditional clinical trials leads to wider credible intervals around point estimates. The design choice to use a relatively small maximum number of patients allowed in each treatment–signature combination (*n* = 150) needs to be contextualized within the framework of ProBio, whose purpose is to efficiently screen a large number of hypotheses to prioritize those most likely to be true for continued evaluation. The adaptive randomization allocates more patients to hypotheses that the data generated within the trial have shown to be plausible. This means that fewer patients are ‘wasted’ on hypotheses that are unlikely to be true. The shared control arm means that a larger proportion of patients are allocated to investigational treatment arms, decreasing the overall need for a large sample size. The relatively small maximum number of patients means that hypotheses graduating from the trial need to have large effect sizes such that they can have a real impact on improving patient outcomes and change standard of care. In fact, the observed sample size reported here is consistent with the expected size from extensive simulation studies in the planning phase of the trial^35^. We presented several analyses comparing efficacy across arms and biomarker signatures or groups, which might raise concerns about multiple comparisons. ProBio was powered for the reported primary analysis (that is, the superiority of ARPIs in the biomarker-unselected ‘all’ patient population), which was planned and powered through statistical simulations. This primary analysis is based on predefined thresholds and forms the basis for our main conclusions. However, we also present results from additional analyses, such as comparisons between investigational arms and across the other predefined biomarker signatures. Akin to most clinical trials, ProBio is not powered to maintain a certain alpha level across all these additional analyses. For these secondary objectives, we used vague priors centered around null values of no differences in the Bayesian framework, which decreases the risk of spurious finding. Still, results from any secondary and exploratory analyses should be interpreted with caution as these are not as robust as the results from the primary analysis, but do serve to further corroborate the observed superiority and generate further valuable insights.

ctDNA was undetectable in 33% of the screened men, which is in line with other cohorts that synchronously analyze cell-free DNA and germline DNA^16,36^. Conversely, higher ctDNA detection rates have been reported with commercial cell-free DNA-only analysis approaches, which are unable to control for clonal hematopoiesis of indeterminate potential and, therefore, may incorrectly classify patients as ctDNA positive due to false-positive biomarker findings in the white blood cell subpopulations^37^. While having detectable ctDNA is necessary to infer the patients’ biomarker signature profiles, it may limit the generalizability of our findings to a subset of patients who have a higher disease burden and poorer prognosis^15,16^, which could potentially lead to inconsistencies when comparing biomarker findings from tissue-based studies. Encouragingly, exploratory analyses from the PROfound trial demonstrated consistent results of superior PARP inhibitor outcomes in mCRPC with homologous recombination deficiency, regardless if ctDNA was detectable or not^38^.

Within our predefined ctDNA biomarker signatures, the *AR* biomarker definition excluded whole-gene *AR* copy number amplifications despite the ProBio assay being capable of detecting this event^7^. We chose to limit our definition to known AR ligand-binding domain mutations, GSRs and intra-AR copy number alterations based on prior research demonstrating the lack of independent prognostic value of whole-gene *AR* copy number amplification when correcting for ctDNA fraction^15,16^. Additionally, *AR* structural rearrangement commonly co-occur with *AR* amplification and are typically driving the poor prognosis properties of *AR*-amplified disease^15^. Recently, extrachromosomal DNA carrying multiple *AR* copies has been described as a source of structural rearrangement complexity and was suggested as a novel treatment resistance mechanism^39^. Unfortunately, circulating free DNA consists of short pre-fragmented DNA that allows for the detection of *AR* rearrangement breakpoints but precludes any recreation of the original DNA molecules or infer the potential extrachromosomal DNA origin of these *AR* amplification events.

Although ProBio’s prespecified biomarker signatures suggested differential treatment effects for ARPIs, they were unable to identify a patient subgroup sensitive to taxanes, warranting further refinement and enhancement of treatment selection strategies. Additionally, the tested biomarker signatures did not include novel biomarkers (for example, *SPOP*) reported after the finalization of the protocol^23^. However, the ProBio assay covers genes beyond the prespecified biomarkers (Supplementary Information), allowing for retrospective analysis of, for example, quantitative analysis of *AR* copy number^40^ or novel biomarker–treatment associations, which may improve the precision and efficacy of taxane therapy selection.

Clinical diversity (for example, stage and timing of metastasis) and heterogeneity in the prior treatment history introduce risks for potential bias in the comparative analyses. Initially, ProBio allowed enrollment of later-line patients with mCRPC, which, upon protocol amendment, was restricted to patients starting systemic therapy for first-line mCRPC. The graduation within the biomarker-unselected ‘all’ patient population may exhibit bias favoring ARPI therapy due to higher rates of rechallenging of the same drug class in the physician’s choice and taxane arms, which in general is less effective than de novo therapy. Rechallenge or sequencing with a treatment from the same drug class was to be avoided in the ARPI arm (for example, no enzalutamide after prior abiraterone)^41^, whereas sequencing with taxanes was permitted (for example, cabazitaxel after prior docetaxel)^42^. Similarly, in order to permit analyses of overall survival, patients who undergo re-randomization in ProBio are not allowed to cross from the physician’s choice control arm to the investigational arms, or vice versa. This has the consequence that re-randomizations are post-randomization events to the first randomization, which causes risk for potential bias in comparative analyses involving re-randomized patients. The robustness of the main analyses with respect to these different risks for potential biases is, however, substantiated by the concordance of results in all sensitivity analyses. ARPIs remained superior over taxanes in patients initiating first-line treatment for mCRPC, in patients initiating first-line treatment for mCRPC who were previously treated with ADT monotherapy for hormone-sensitive disease, and in patients naive for the investigational drug class under evaluation. Additionally, the ProBio findings are supported by results from previous observational studies^11–13^, thereby enhancing the overall level of evidence.

Intensification trials in metastatic hormone-sensitive disease have demonstrated that both metachronous and de novo metastatic patients overall benefit from therapy intensification with ARPIs^43–45^ or docetaxel^46^, while the results presented here are from using these drugs in the castration-resistant setting. However, the uptake of doublet and triplet treatment modalities has been slow. A substantial group of patients with metastatic hormone-sensitive prostate cancer continues to receive ADT monotherapy and thus are ARPI- and taxane-naive at castration-resistant disease onset. This has been exemplified by real-world data^47^ and recent population-based registry studies in Sweden and the United Kingdom^48–50^ meaning that ARPIs and docetaxel remain as effective treatment options in the castration-resistant setting. Therefore, there is a continued need for refined treatment strategies once patients reach castration-resistant disease. Once reached, current European Association of Urology (EAU)/National Comprehensive Cancer Network (NCCN) guidelines acknowledge the validity of both taxane-based chemotherapy and ARPIs for first-line metastatic castration-resistant disease without a clear preference^51,52^. The limited number of comparative trials has left treatment selection largely reliant on individual clinician and patient preference. The results presented in this Article provide therapeutic direction, offering clinicians evidence-based guidance for the therapeutic management of this still-prevalent patient population. Our findings underscore the importance of favoring ARPIs over taxane-based chemotherapy in patients with mCRPC with detectable ctDNA who are progressing on prior ADT monotherapy, and may be considered as contributing evidence to the field with the potential to refine existing guidelines upon further validation.

Our results demonstrate that, on average, ARPIs outperform taxanes in all ctDNA-positive patients with mCRPC who are ARPI-naive and with or without prior docetaxel exposure. This may be particularly the case in *AR* (SNV/GSR)-negative and *TP53* wild-type patients or *TMPRSS2–ERG* fusion-positive patients. Conversely, new treatment strategies might be relevant to *TP53*-altered patients considering their poor and similar short-term outcomes, irrespective of a treatment with ARPIs or taxanes. Furthermore, ProBio’s biomarker-driven and outcome-adaptive design shows the feasibility of using ctDNA for treatment personalization and may represent a new model for precision medicine trials in oncology.

## Methods

### Trial design

ProBio is an ongoing, multicenter, randomized, outcome-adaptive, biomarker-driven platform trial (ClinicalTrials.gov registration: NCT03903835) in men with metastatic prostate cancer^35,53^, approved by ethics boards in Sweden (ID: 2018/2206–32; 22 October 2018), Belgium (ID: BC-06057; 20 March 2020), Norway (ID: Søknadsnummer 81005/58005; 24 June 2020) and Switzerland (ID: BASEC 2021–02495; 01 March 2022). At inclusion and upon receiving the patient’s written informed consent, the patient is enrolled in the electronic case report form system (SMART-TRIAL, Greenlight Guru) for data collection and treatment follow-up. Upon peripheral blood collection, synchronous analyses of plasma-derived ctDNA and whole-blood germline DNA are applied for biomarker assessment using an in-house laboratory-developed test (see ‘Sample processing, bioinformatic analysis and variant assessment’ in the Supplementary Information)^7^. These analyses determine whether patients’ tumors have genetic alterations in the *AR*, *TP53* or DNA repair genes, or harbor the *TMPRSS2–ERG* gene fusion. The four biomarkers categorize patients into sixteen (4 × 4) biomarker subgroup combinations, which are used for randomization (Supplementary Fig. 1). Since the prevalence of subgroup combinations can be low, we evaluated efficacy within five biomarker signatures or groups (that is, prespecified sets of subgroup combinations): (1) any biomarker subgroup combination (that is, biomarker-unselected or *‘*all’ patients); (2) no SNV or GSR in the *AR* gene and no alterations in the *TP53* gene (that is, *AR* (SNV/GSR) negative and *TP53* wild type); (3) homologous recombination deficiency (that is, deactivating somatic or high-impact germline alteration in DNA repair genes); (4) alterations in *TP53* (that is, *TP53* altered); and (5) presence of the *TMPRSS2–ERG* fusion (that is, *TMPRSS2*–*ERG* fusion positive; Supplementary Fig. 1).

Patients are randomized to either the control arm, or one of the investigational biomarker-driven treatment arms (Fig. 1a). For the control arm patients, the treating physician selects a standard-of-care treatment. Participating physicians, both in the control and investigational treatment arms, are blinded to the biomarker results. ProBio uses outcome-adaptive randomization, which adapts over time to increase allocation to more promising treatments^35^. Adaptation takes into account information on the biomarker subgroup combinations and the accumulated evidence during the trial on the efficacy of the treatment arms. At disease progression^54^, patients in the investigational arms can be re-randomized based on a new ctDNA analysis, whereas control arm patients can receive a new line of physician’s choice treatment within ProBio, allowing each patient to contribute with multiple data points for outcome analyses.

ProBio’s analytic framework compares different investigational treatment arms against the physician’s choice control arm, and across the investigational arms within the prespecified biomarker signatures. The control group enables the evaluation of biomarker-guided treatment decisions versus physician’s choice strategies. Randomized comparisons across investigational treatment arms assess treatment class efficacy. Stratified comparisons by biomarker status allow for interaction analysis to assess treatment-predictive potential by comparing treatment efficacy in patients with and without a specific biomarker. All comparisons use concurrently randomized patients.

An independent data safety and monitoring committee reviews data biannually together with unblinded study statisticians and provides recommendations for early termination based on graduation or futility stopping rules, that is, probability of superiority > 85% for graduation, and probability of superiority < 15% for futility on a minimum of 25 evaluated biomarker signature–treatment combinations. Additionally, the probability of superiority within all biomarker subgroup combinations belonging to the evaluated biomarker signature needs to be >70% or <50% for graduation or futility, respectively (see details on stopping rules for graduation and futility in Supplementary Information). Upon termination recommendation and data lock, a minimum follow-up of 4 months is ensured for all patients before final analyses. The trial protocol adheres to SPIRIT 2013 (ref. ^55^), was approved by ethics boards in Sweden, Belgium, Norway and Switzerland, and was conducted in accordance with the Declaration of Helsinki and Good Clinical Practice guidelines. The trial protocol is available as Supplementary Information. Reporting adhered to the Consolidated Standards of Reporting Trials (CONSORT) guidelines. We designed the trial, oversaw data collection by consortium members and take responsibility for data accuracy and trial fidelity. Only authors contributed to manuscript writing. Funding organizations (as detailed in Acknowledgements and Supplementary Information) were not involved in protocol development, data analysis or manuscript preparation.

### Patients

Eligible patients had progressive mCRPC and were initiating first-line or second-line systemic therapy for mCRPC. The type of progressive disease at trial entry was recorded per Prostate Cancer Clinical Trials Working Group recommendations^54^, encompassing PSA only, bone only (with or without nodal disease), nodal disease only and visceral disease (with or without involvement of other sites). Recent routine laboratory (that is, hematology, biochemistry, enzymes and oncological markers) and conventional imaging (that is, computerized tomography or magnetic resonance imaging, and bone scintigraphy scans) results were mandatory for all participants. Prior ADT intensification with for example docetaxel or an ARPI for metastatic hormone-sensitive or non-metastatic castration-resistant disease was allowed, and continuous ADT was required. All patients had adequate performance score, bone marrow, renal and hepatic function to receive any treatment available in the ProBio platform. Initially, the study considered patients initiating first-line or second-line systemic treatment for mCRPC as eligible. However, after a protocol amendment in December 2020, enrollment was limited to patients starting first-line systemic treatment for mCRPC. Additionally, sequencing or rechallenging with ARPIs was not allowed from then on. This decision was primarily driven by ethical considerations and clinical efficacy evidence, with the known cross-resistance between ARPIs^41^ in contrast to taxane sequencing (for example, cabazitaxel after docetaxel) after prior ARPI exposure^42^. However, it should be noted that any type of systemic therapy or ADT intensification before mCRPC was still allowed and that this has remained unchanged throughout the trial. Participants were excluded if their biomarker signature could not be determined due to undetectable ctDNA, technical failure or detection of microsatellite instability or a hypermutator genotype. Full eligibility criteria are provided in the protocol.

### Biomarker signature definitions

The initial predefined biomarker signatures are defined as tumor properties or mutations in certain genes or pathways encompassing *AR*, *TP53*, homologous recombination deficiency genes and the *TMPRSS2–ERG* fusion (Supplementary Fig. 6). Only clonal alterations in *TP53*, *TMPRSS2–ERG* and homologous recombination deficiency genes qualified to categorize a patient as biomarker positive, whereas any hotspot SNV or GSR in the *AR* gene, regardless of clonality, qualified to classify a study participant as *AR* (SNV/GSR) positive.

Detailed information on bioinformatic analysis and somatic and germline variant assessment is provided in the Supplementary Information. The biomarker signature *AR* (SNV/GSR) negative and *TP53* wild type is defined by absence of relevant GSRs or hotspot single-nucleotide variation in *AR*, and without any relevant structural variation, mutation or homozygous deletion in *TP53*. The *TMPRSS2–ERG* fusion biomarker signature is defined by the detection of a gene fusion by structural rearrangements or by deletion through copy number alteration analysis. The *TP53*-altered biomarker signature is defined as the detection of high-impact structural variation affecting one or more exons, hotspot or high-impact point mutation, or a homozygous deletion in the *TP53* gene. Homologous recombination deficiency is defined by the detection of relevant somatic or germline structural variation affecting one or more exons, hotspot or high-impact point mutation not known to be benign, or a homozygous deletion in *ATM*, *ATR*, *BARD1*, *BRCA1*, *BRCA2*, *BRIP1*, *CDK12*, *CHEK2*, *FANCA*, *MRE11A*, *NBN*, *PALB2*, *RAD50*, *RAD51*, *RAD51B*, *RAD51C* or *RAD51D*.

### Treatments

ProBio initially randomized patients to either the control (that is, physician’s choice) or an investigational treatment arm, including ARPIs (either 1,000 mg of abiraterone acetate or 160 mg of enzalutamide daily), taxanes (either docetaxel at a dosage of 75 mg or cabazitaxel at a dosage of 20–25 mg per square meter of body-surface area intravenously every 3 weeks), or carboplatin (area under the curve 4–5, every 3 weeks). Conditions for use were in accordance with the summary of product characteristics and local guidelines. Following protocol amendment, randomization was also opened to niraparib in combination with abiraterone acetate. Here, we compare the results of ARPIs and taxanes.

### Randomization

The absence or presence of the four selected biomarkers (that is, *AR*, *TP53*, homologous recombination deficiency genes and *TMPRSS2*–*ERG* gene fusion) generated 16 distinct biomarker subgroup combinations. Each patient was exclusively assigned to one subgroup. Randomization was stratified on these subgroups, leading to varying randomization probabilities across different subgroup levels. While early termination was based on the evaluation of therapy classes within biomarker signatures, treatment arms were also compared against the control arm within specific biomarker subgroup combinations. Probabilities of superiority derived in the latter comparisons are utilized to update the initial randomization probabilities in each biomarker subgroup combination. We ensured that the randomization probabilities for the control arm, within each subgroup, are equal to or higher than the maximum randomization probabilities in the experimental arms. Consequently, randomization between a graduating therapy arm and physician’s choice will mimic a 1:1 randomization scheme, which also has the advantage of mitigating the impact of potential temporal changes in the patient characteristics on the study outcomes.

Additionally, randomization takes into account prior systemic therapy exposure and avoids sequencing or rechallenging with second-generation ARPIs (for example, no randomization to enzalutamide when the patient was previously treated with abiraterone acetate). However, sequencing with taxanes was allowed (that is, cabazitaxel after prior therapy with docetaxel). To facilitate this, randomization probabilities for non-permitted treatment sequences were set to zero.

### Outcomes

The primary endpoint was the time to NLCB, defined as the date and specific reason a therapy was ultimately discontinued^54^. This endpoint focuses on determining when a treatment should be discontinued when the patient is NLCB rather than strictly at the first evidence of progression. A patient was deemed NLCB according to the physician’s judgment by taking into account the composite evaluation of PSA, conventional radiology and clinical assessment using the following progression definitions. Biochemical progression after a decline from baseline, was defined as the time from the start of therapy to the first PSA increase that is ≥ 25% and ≥ 2 ng ml^−1^ above the nadir. If there was no decline from baseline, PSA progression was defined as a ≥ 25% increase and a ≥ 2 ng ml^−1^ increase from baseline beyond 12 weeks. Radiologic progression was defined as the time from random assignment to the date when the first site of disease was found to progress, based on a manifestation-specific definition of progression, or death, whichever occurs first. Nodal and visceral disease was assessed by computed tomography. Bone disease was assessed by Tc99 bone scan with at least two new lesions on the first posttreatment scan to deem a patient progressive. Radiologic evaluation occurred at fixed intervals (that is, every 8 weeks for the first 24 weeks, followed by every 12 weeks) or triggered by clinical and/or biochemical progression as determined by the treating physician. For overall trial conduct, a local radiologic response assessment was used for the primary endpoint definitions. Clinical progression was defined based on the clinical judgment of various factors, including but not limited to AEs or disease-related complications, clinical deterioration, presence of clinically meaningful pain and an increase in analgesic consumption. Overall survival and SAE/AE rates were secondary endpoints. Overall survival was defined as the time from the initial study randomization to death from any cause. Information regarding all-cause mortality was updated biannually using electronic health records. Details on outcome definitions are provided in the protocol.

### Statistical analysis

ProBio has been designed as a screening device to identify patient subgroups more likely to benefit from a therapy class. We set thresholds based on the posterior probability of superiority for treatment–biomarker combinations to exit the platform trial for an early indication of superiority (probability of superiority > 85%) or for futility (probability of superiority < 15%). These thresholds were calibrated through simulation studies to control type I error and ensure sufficient power^35^. The estimated type I error rate was less than 10% for each therapy when concurrently evaluating a maximum of five arms within five biomarker signatures and less than 5% for each biomarker signature–treatment combination. Power ranged between 65% and 83% depending on the simulation scenario. Each biomarker signature–treatment class combination allowed a maximum of 150 randomized patients.

We utilized a Bayesian Weibull accelerated failure time model^56^ estimated on all data (that is, first and second randomization) for the time to NLCB and on first randomization data for overall survival. Using these fitted models, the posterior distribution of the treatment effect was calculated as the relative impact on the expected survival time, referred to as the STR. An STR above 1 implies longer expected survival, while an STR below 1 suggests shorter expected survival (for example, an STR of 1.2 is straightforwardly interpreted as a 20% increase in survival time, while an STR of 0.8 means a 20% decrease). We compared ARPIs and taxanes to the physician’s choice group, as well as against each other using taxanes as a reference. We complemented the results for the time-to-event endpoints by providing the posterior survival curves, the corresponding Kaplan–Meier estimates and the estimated posterior median survival times. For the (log) STR, we adopted normal vague priors with mean 0 and standard deviation 0.5. Posterior distribution results were summarized by medians and 90% CrI, along with the posterior probability of superiority. Result robustness was assessed through prespecified subgroup sensitivity analyses, specifically focusing on patients starting first-line systemic therapy for metastatic castration-resistant disease, patients starting first-line systemic therapy for metastatic castration-resistant disease after prior ADT monotherapy (that is, patients naive for therapy intensification in metastatic hormone-sensitive or non-metastatic castration-resistant disease) and patients without prior exposure to the drug class under evaluation during ProBio (that is, no treatment class rechallenge). Within the investigational arms, the latter ‘no treatment class rechallenge’ sensitivity analysis thus encompassed a comparison of patients that were naive to the randomized drug class.

The treatment-predictive value of the biomarker signatures was assessed through interaction analyses comparing the ARPI to the taxane arm. The differential treatment effect was quantified by examining the posterior distribution of the interaction coefficient, which represents the multiplicative effect of a treatment in the biomarker-positive patients compared to the effect of the treatment in the biomarker-negative patients.

All patients receiving ARPIs or taxanes, including those in the control arm, were included in the safety population. In this analysis, the occurrence of AEs and SAEs were compared. All analyses were performed in R (version 4.2.2), with additional details being provided in the Supplementary Information.

### Reporting summary

Further information on research design is available in the Nature Portfolio Reporting Summary linked to this article.

## Online content

Any methods, additional references, Nature Portfolio reporting summaries, source data, extended data, supplementary information, acknowledgements, peer review information; details of author contributions and competing interests; and statements of data and code availability are available at 10.1038/s41591-024-03204-2.

## Supplementary information

Supplementary Information Supplementary Methods, Figs. 1–6 and Tables 1–4; lists of the ProBio investigators, sites, coworkers and staff contributing in ProBio, funding organizations and industry collaborators supporting ProBio; and the ProBio (NCT03903835, v4.1) trial protocol.

Reporting Summary

## Data Availability

All data relevant for the interpretation of our findings reported here are provided in the Article or the Supplementary Information. The data supporting the findings of this trial can be accessed under the following conditions: Requests for data access should be directed to the corresponding author. Access to the sequencing data requires approval from the Swedish Ethical Review Authority and an agreement with the data protection and legal unit at Karolinska Institutet. Data providing information on individual outcomes or genotypes are classified as personal registry information under Swedish law (Personal Data Act), thus prohibiting submission to a public repository. The data can be used for retrospective auxiliary research questions upon ethical committee approval. Researchers must provide a study-specific protocol conforming to local guidelines. Requests will be processed within 1–2 months upon submission of a complete and compliant auxiliary research-specific study protocol. For further details or to initiate a request, please contact the corresponding author.

## Code availability

Code for the statistical analysis is available via GitHub at https://github.com/alecri/arpi_all/.
