# Author Correction: Androgen receptor pathway inhibitors and taxanes in metastatic prostate cancer: an outcome-adaptive randomized platform trial

**DOI:** 10.1038/s41591-024-03268-0

**Published:** 2024-08-28

**Authors:** Bram De Laere, Alessio Crippa, Andrea Discacciati, Berit Larsson, Maria Persson, Susanne Johansson, Sanne D’hondt, Rebecka Bergström, Venkatesh Chellappa, Markus Mayrhofer, Mahsan Banijamali, Anastasijia Kotsalaynen, Céline Schelstraete, Jan Pieter Vanwelkenhuyzen, Marie Hjälm-Eriksson, Linn Pettersson, Anders Ullén, Nicolaas Lumen, Gunilla Enblad, Camilla Thellenberg Karlsson, Elin Jänes, Johan Sandzén, Peter Schatteman, Maria Nyre Vigmostad, Martha Olsson, Christophe Ghysel, Brieuc Sautois, Wendy De Roock, Siska Van Bruwaene, Mats Anden, Ingrida Verbiene, Daan De Maeseneer, Els Everaert, Jochen Darras, Bjørg Y. Aksnessether, Daisy Luyten, Michiel Strijbos, Ashkan Mortezavi, Jan Oldenburg, Piet Ost, Martin Eklund, Henrik Grönberg, Johan Lindberg

**Affiliations:** 1 Department of Medical Epidemiology and Biostatistics, Karolinska Institutet, Stockholm, Sweden; 2 Department of Human Structure and Repair, Ghent University, Ghent, Belgium; 3 Health, Innovation and Research Institute (Clinical Trial Unit), University Hospital Ghent, Ghent, Belgium; 4 National Bioinformatics Infrastructure Sweden, Science for Life Laboratory, Department of Cell and Molecular Biology, Uppsala University, Uppsala, Sweden; 5 Department of Urology, University Hospital Ghent, Ghent, Belgium; 6 Department of Oncology, Capio Saint Göran’s Hospital, Stockholm, Sweden; 7 Department of Oncology, Länssjukhuset Ryhov, Jönköping, Sweden; 8 Department of Oncology, Karolinska University Hospital, Stockholm, Sweden; 9 Department of Oncology, Uppsala University Hospital, Uppsala, Sweden; 10 Department of Oncology, University Hospital of Umeå, Umeå, Sweden; 11 Department of Oncology, Sundsvalls sjukhus, Sundsvall, Sweden; 12 Department of Oncology, Centralsjukhuset Karlstad, Karlstad, Sweden; 13 Department of Urology, Onze Lieve Vrouwziekenhuis, Aalst, Belgium; 14 Department of Oncology, Stavanger University Hospital, Stavanger, Norway; 15 Department of Oncology, Centrallasarettet Växjö, Växjö, Sweden; 16 Department of Urology, AZ Sint Jan Brugge AV, Bruges, Belgium; 17 Department of Oncology, CHU de Liège - site Sart Tilman, Liège, Belgium; 18 Department of Oncology, Ziekenhuis Oost- Limburg, Genk, Belgium; 19 Department of Urology, AZ Groeninge, Kortrijk, Belgium; 20 Department of Oncology, Länssjukhuset i Kalmar, Kalmar, Sweden; 21 Department of Oncology, Falu lasarett, Falu, Sweden; 22 Department of Oncology, AZ Sint-Lucas, Bruges, Belgium; 23 Department of Oncology, Vitaz campus Sint-Niklaas Lodewijk, Sint-Niklaas, Belgium; 24 Department of Urology, AZ Damiaan, Oostende, Belgium; 25 Department of Oncology, Ålesund Hospital, Ålesund, Norway; 26 Department of Oncology, Virga Jessa, Hasselt, Belgium; 27 Department of Oncology, GZA Sint-Augustinus, Antwerpen, Belgium; 28 Department of Urology, Universitätsspital Basel, Basel, Switzerland; 29 Department of Urology, Universitätsspital Zürich, Zürich, Switzerland; 30 Department of Oncology, Akershus University Hospital, Nordbyhagen, Norway; 31 Department of Radiation Oncology, GZA Sint-Augustinus, Antwerpen, Belgium; 32 Prostatacancer Centrum, Capio S:t Görans Sjukhus, Stockholm, Sweden; 33 Department of Medical Epidemiology and Biostatistics, Science for Life Laboratory, Karolinska Institutet, Stockholm, Sweden

**Keywords:** Predictive markers, Tumour biomarkers, Randomized controlled trials, Prostate cancer

Correction to: *Nature Medicine* 10.1038/s41591-024-03204-2, published online 20 August 2024.

In the version of the article initially published, Rebecka Bergström’s name appeared incorrectly (as R. Bergström) and has now been amended in the HTML and PDF versions of the article.

