# Supplementary material for: Androgen receptor pathway inhibitors and taxanes in metastatic prostate cancer: an outcome-adaptive randomized platform trial
Source: Nat Med. 2024 Aug 20;30(11):3291–302. doi: 10.1038/s41591-024-03204-2 (PMC11564108; doi:10.1038/s41591-024-03204-2)
Supplement: Supplementary file 1 — Supplementary Methods, Figs. 1–6 and Tables 1–4; lists of the ProBio investigators, sites, coworkers and staff contributing in ProBio, funding organizations and industry collaborators supporting ProBio; and the ProBio (NCT03903835, v4.1) trial protocol. [file 41591_2024_3204_MOESM1_ESM.pdf]

nature medicine

Supplementary information https://doi.org/10.1038/s41591-024-03204-2

Androgen receptor pathway inhibitors and taxanes in metastatic prostate cancer: an outcome-adaptive randomized platform trial

In the format provided by the authors and unedited

Table of Contents:

Sample processing, bioinformatic analysis and variant assessment. 2

Additional details on statistical analysis 3

Main analysis: efficacy of therapy arms. 3

Interaction analysis: differential treatment effect. 5

Stopping Rules for graduation and futility 6

Supplementary Figures and Tables 7

Supplementary Figure S1. Biomarker signatures and subgroup combinations. 7

Supplementary Figure S2. Adaptation of randomization probabilities over time for disclosed treatment arms. 8

Supplementary Figure S3. Probability distributions of survival time ratio in selected biomarker signatures. 9

Supplementary Figure S4. Posterior survival curves and Kaplan-Meier estimates by therapy arm in selected biomarker signatures. 10

Supplementary Figure S5. Differential treatment effects according to biomarker signature status (positive vs negative). 12

Supplementary Figure S6. The ProBio gene panel and biomarker subgroup assignments. 14

Supplementary Table S1. Treatment received in the investigational and control arms 15

Supplementary Table S2. The type(s) of progressive disease which ultimately resulted in the discontinuation of the treatment and the no longer clinically benefitting endpoint. 16

Supplementary Table S3. Sensitivity analysis for the time to no longer clinically benefitting and overall survival in the five prespecified biomarker signatures. 17

Supplementary Table S4. Differential treatment effects in the time to no longer clinically benefitting for AR pathway inhibitors versus taxanes, stratified by pre-specified biomarker signature status. 18

List of ProBio investigators 19

List of sites, co-workers, and staff contributing in ProBio 23

List of funding organizations and industry collaborators supporting ProBio 27

References in Supplementary Appendix: 28

ProBio (NCT03903835, v4.1) trial protocol 29

Sample processing, bioinformatic analysis and variant assessment.

Briefly, plasma was enriched from 2-3×10 ml Streck BCT-collected blood (Streck, Catalog# 230470) using a double spin protocol, and stored at − 80°C prior to cell-free DNA extraction. Upon plasma aspiration, leftover Streck-BCT buffy coats were stored for germline DNA extraction. Cell-free and germline DNA isolations were performed using the QiaSymphony (Qiagen, Catalog# 9001301) and NucleoSpin Blood (Macherey Nagel, Catalog# 740951.50) technology, respectively. Upon isolation, quality control, and shearing of germline DNA, 20-200 ng of cell-free DNA and 150 ng of germline DNA were used to prepare sequencing libraries using Kapa DNA HypePrep (Roche, Catalog# KK8503). Cell-free and germline DNA libraries were subjected to hybridization-based capture with the ProBio panel (Supplementary Figure S6). The ProBio panel (oligos ordered from Twist Biosciences) was designed to enrich target regions in the human genome known to harbor somatic- or germline driver alterations in prostate cancer. Previously reported somatic- and germline alteration types determined how individual genes were targeted (e.g. for structural variation or copy-number alterations). The panel design was adapted to maximize the fraction of patients carrying relevant alterations in the pre-specified biomarker signatures, e.g. for homologous recombination deficiency, by including genes that are rarely altered but that collectively significantly increase the number of biomarker signature positive patients. Paired-end sequencing, 90- and 15 million read-pairs for cfDNA- and germline DNA, was performed on a NovaSeq instrument (Illumina, Catalog# 20012850).

Sequencing data underwent processing using an in-house bioinformatics pipeline, AutoSeq, which integrates publicly available- and in-house developed tools. Briefly, following mapping with BWA MEM (v.0.7.17-r1188) [1], realignment was performed (GATK, v4.1.2.0 [2]) and small variants were called using Mutect2 (v.4.1.2.0) [2 3], Strelka (v2.9.10) [4], VarDict (v.1.8.3)

[5] and VarScan2 (v.2.4.4) [6]. Results were merged with SomaticSeq (v3.7.3) [7] and annotated with the Ensembl Variant Effect Predictor (v110.1) [8]. Variants called by ≥2 callers and with impact high or moderate were kept. Calling of small germline variants was performed with GATK HaplotypeCaller (v4.1.2.0) [2]. Structural variants were called with an in-house developed algorithm (SVcaller, v0.1) [9]. Copy-number alterations were identified using CNVkit (v.0.9.11) [10] and in-house developed software (Jumble, v0.1, https://github.com/ClinSeq/jumble). Purity and ploidy analysis was performed using PureCN. (v1.12.2) [11]. All variants and variant types were manually inspected in a user interface, complemented with the integrated genomics viewer (IGV, v2.16.2) [12] to remove any variant with artifacts [13]. Somatic- and germline alterations were subsequently assessed according to pre-specified criteria (Supplementary Figure S6) to assign each patient to a specific biomarker subgroup combination (Supplementary Figure S1).

Additional details on statistical analysis

Main analysis: efficacy of therapy arms.

We utilized Bayesian accelerated failure time models, assuming a Weibull distribution for the survival outcomes T_e, where e = 1 represents time to no longer clinically benefitting survival and e = 2 represents overall survival. The Weibull distribution is characterized by two parameters: the shape parameter γ_e and the scale parameter μ_e. In the survival models, we assumed a common γ_e while the natural logarithm of μ_e is modeled as a linear function of the therapy arms:

$$log(\mu_{ei}) = \beta_{0e} + \beta_{1e}X_{1i} + \beta_{2e}X_{2i}$$

where X_{1i} and X_{2i} are indicator variables representing the randomized allocation of the i-th patient to AR Pathway Inhibitors ($X_{1i} = 1$) or Taxanes ($X_{2i} = 1$), respectively.

We estimated the survival models separately in the five prespecified biomarker signatures $S_s, s = 1, \cdots, 5$. The treatment effects were measured using Survival Time Ratio (STR), which was calculated by exponentiating the regression coefficients. The STR represents the relative increase (if STR > 1) or decrease (otherwise) in expected survival time when comparing each arm to the reference group. A value of STR = 1 indicates no differences in efficacy between the groups. To determine the probability of superiority, we calculated the posterior probability that the STR was greater than 1.

We assumed the following prior distribution: $\gamma_e \sim Exp(1)$, $\beta_{0e} \sim Normal(b_{0e}, 0.5)$ (with $b_{01} = 3$ and with $b_{02} = 4$), and $\beta_{je} \sim Normal(0, 0.5)$ (j = 1, 2), which translates into an 83% probability that the a-priori STR falls between 0.5 and 2, and into a 50% initial probability of superiority.

For our point estimates, we used the medians of the posterior distribution, providing a summary measure for the treatment effects. To capture the uncertainty in the estimates, we calculated 90% Credible Intervals (CrI) using the 5th and 95th quantiles of the posterior distribution, which serve as lower and upper limits, respectively. To derive the posterior distributions, we employed Hamiltonian Monte Carlo algorithms implemented in the probabilistic programming language Stan, accessed through the rstanarm package in R version 4.2.2.

We created tables displaying the STR and the probability of superiority for the experimental arms compared to the control group ($exp(\beta_{1e})$ and $exp(\beta_{2e})$), and for ARPI compared to Taxanes ($\frac{exp(\beta_{1e})}{exp(\beta_{2e})}$) for both survival endpoints and across biomarker signatures. We also included the number of events and randomized patients in each comparison to provide additional context. Furthermore, we illustrated the observed results by plotting the posterior

distribution of the STR. This graph allowed for a clearer understanding of the range of possible effect sizes. To provide a more comprehensive understanding of the results, we translated the effect size based on the posterior distribution of the regression coefficients into posterior survival curves, further complemented by the non-parametric Kaplan-Meier curves. We also reported information on the estimated median survival times in tables inserted in the survival curves figures.

Interaction analysis: differential treatment effect.

For the time to no longer clinically benefitting (primary endpoint), we examined the extent of differential treatment effects between the experimental therapy arms (AR Pathway Inhibitors versus Taxane) based on the presence or absence of the prespecified biomarker signatures. In this analysis, we excluded the Control group as it consisted of multiple treatments, making it challenging to interpret the results. To compare the efficacy of ARPI versus Taxanes based on the biomarker signatures, we extended the survival model by incorporating an interaction term:

$$log(\mu_{i1}) = \beta_{01} + \beta_{11}X_{1i} + \beta_{21}S_{si} + \beta_{31}X_{1i}S_{si}$$

where S_{si} is an indicator variable that takes a value of 1 for patients who were positive for the biomarker signature S_s, and the interaction term $X_{1i}S_{si}$. This interaction term allowed us to assess whether the treatment effect varied depending on the biomarker status of the patients. To assess the extent of differences in treatment effects, we calculated the Survival Time Ratio (STR) separately for patients who were negative (STR_0) and positive (STR_1) for the biomarker signature S_s. These ratios represent the relative increase or decrease in expected survival time for each group compared to the reference group (Taxanes). Furthermore, we summarized the distribution of the exponential of the coefficient β_{31}, which quantifies the additional

multiplicative effect. This coefficient reflects the extent of difference in treatment effects between patients who are positive versus negative for the biomarker signature S and can be interpreted as the ratio of STR_1 to STR_0.

In the presentation of the results for the interaction analysis, we followed a similar format to the main analysis.

Stopping Rules for graduation and futility

**Graduation**:	$n \geq 25$ for the evaluated treatment-signature combination<br>$\pi_s \geq 85\%$, π_s being the probability of superiority for the evaluated treatment vs the control within the biomarker signature<br>$\pi_j \geq 70\%$, π_j being the probability of superiority for the evaluated treatment vs the control within all the biomarker subgroup combinations belonging to the evaluated signature
**Futility**:	$n \geq 25$ for the evaluated treatment-signature combination<br>$\pi_s \leq 30\%$, π_s being the probability of superiority for the evaluated treatment vs the control within the biomarker signature<br>$\pi_j \leq 50\%$, π_j being the probability of superiority for the evaluated treatment vs the control within all the biomarker subgroup combinations belonging to the evaluated signature
**Max patients**:	$n \geq 150$

Supplementary Figures and Tables

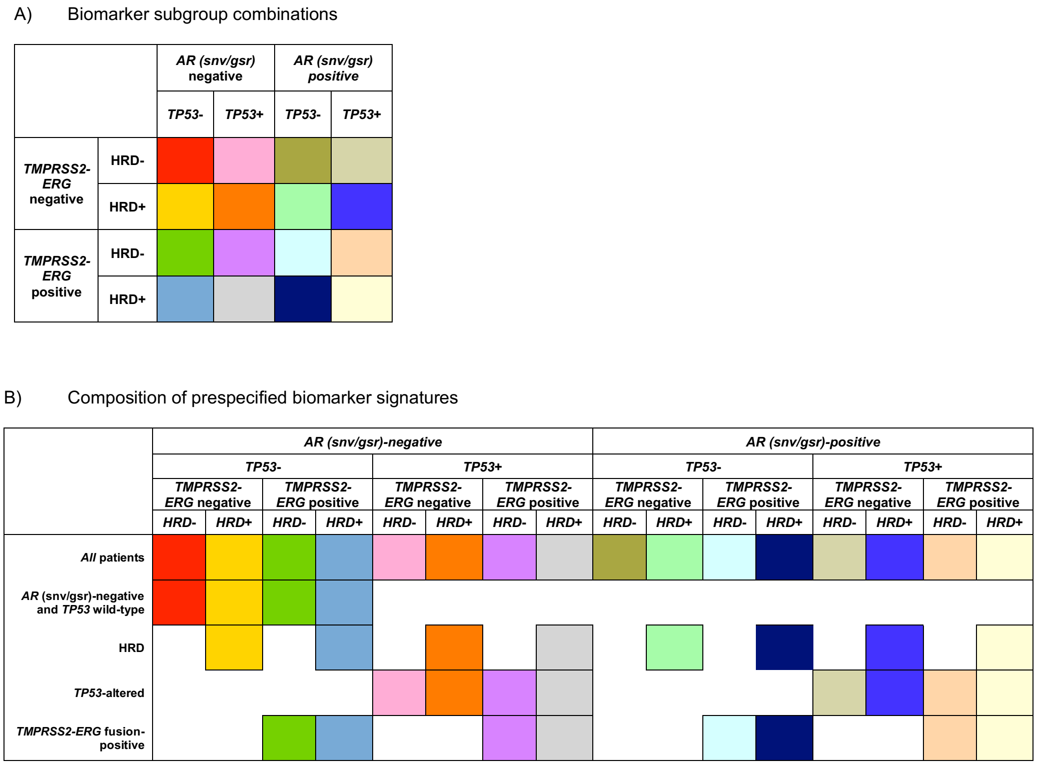

Supplementary Figure S1. Biomarker signatures and subgroup combinations. Genetic biomarkers are assessed pre-randomization by comprehensive genomic profiling of germline and circulating tumor DNA from blood samples. Four biomarkers – androgen receptor (*AR*), homologous recombination deficiency (HRD), *TP53*, and *TMPRSS2-ERG* gene fusion status – are used to classify patients into 16 predefined subgroups as depicted by the different colors in color table A. These 16 subgroups based on biomarker combinations are used for randomization. The efficacy analysis focuses on five specified biomarker signatures: *all* patients (i.e. any biomarker subgroup), *AR* (single nucleotide variant (snv)/genomic structural rearrangement (gsr))-negative and *TP53* wild-type, HRD, *TP53*-altered, and *TMPRSS2-ERG* fusion-positive. Color table B, using the same colors from the 16 predefined subgroups in color table A, illustrates the composition of subgroup combinations within the selected biomarker signatures. Subgroups not included in eligible biomarker signatures are depicted in white.

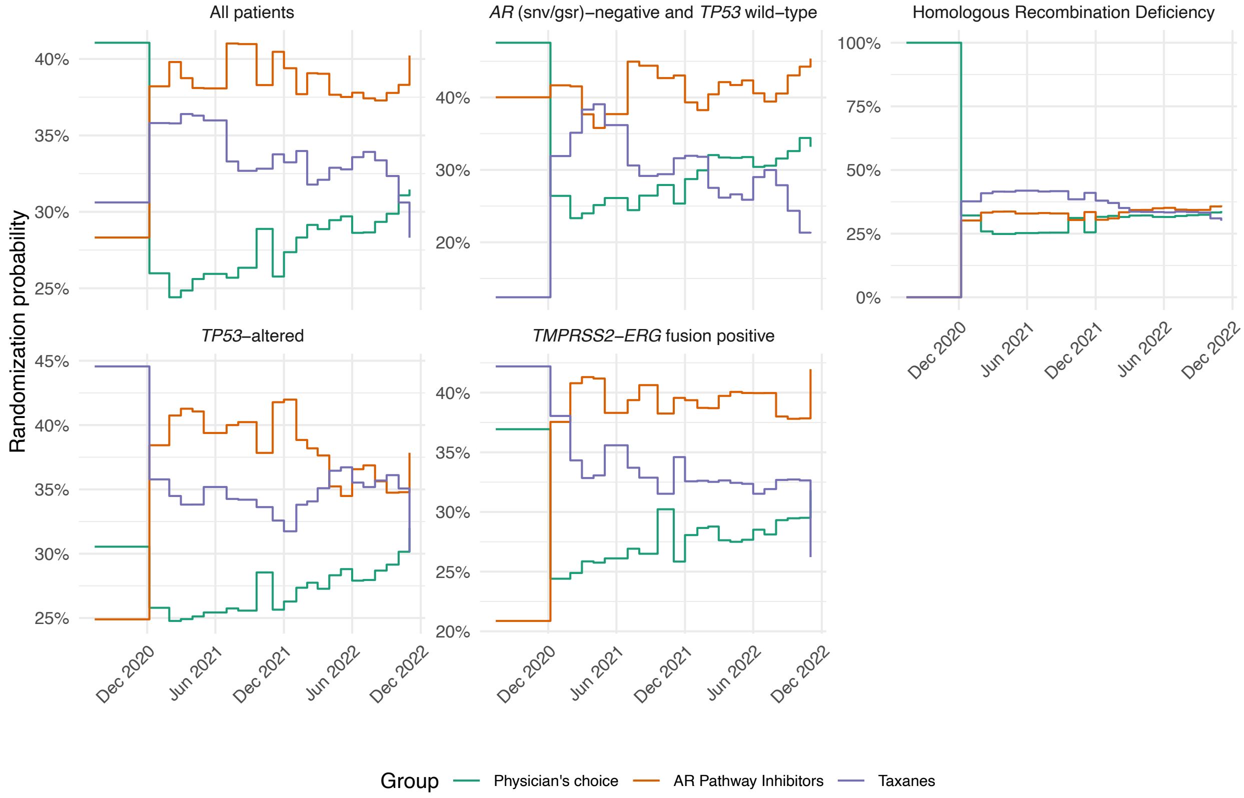

Supplementary Figure S2. Adaptation of randomization probabilities over time for disclosed treatment arms.

Randomization probabilities were fixed until 50 patients had been randomized to experimental arms. These probabilities were monthly updated based on the posterior probabilities of superiority computed on the accumulated data as described in the protocol. Randomization probability for the control group (i.e., physician's choice) was ensured to match the maximum randomization probabilities in the experimental arms, so it also increases with increasing evidence of superiority of AR pathway inhibitors in the selected biomarker signatures. Randomization probabilities are only provided for the disclosed therapies, ensuring that they are re-normalized to a sum of 100%.

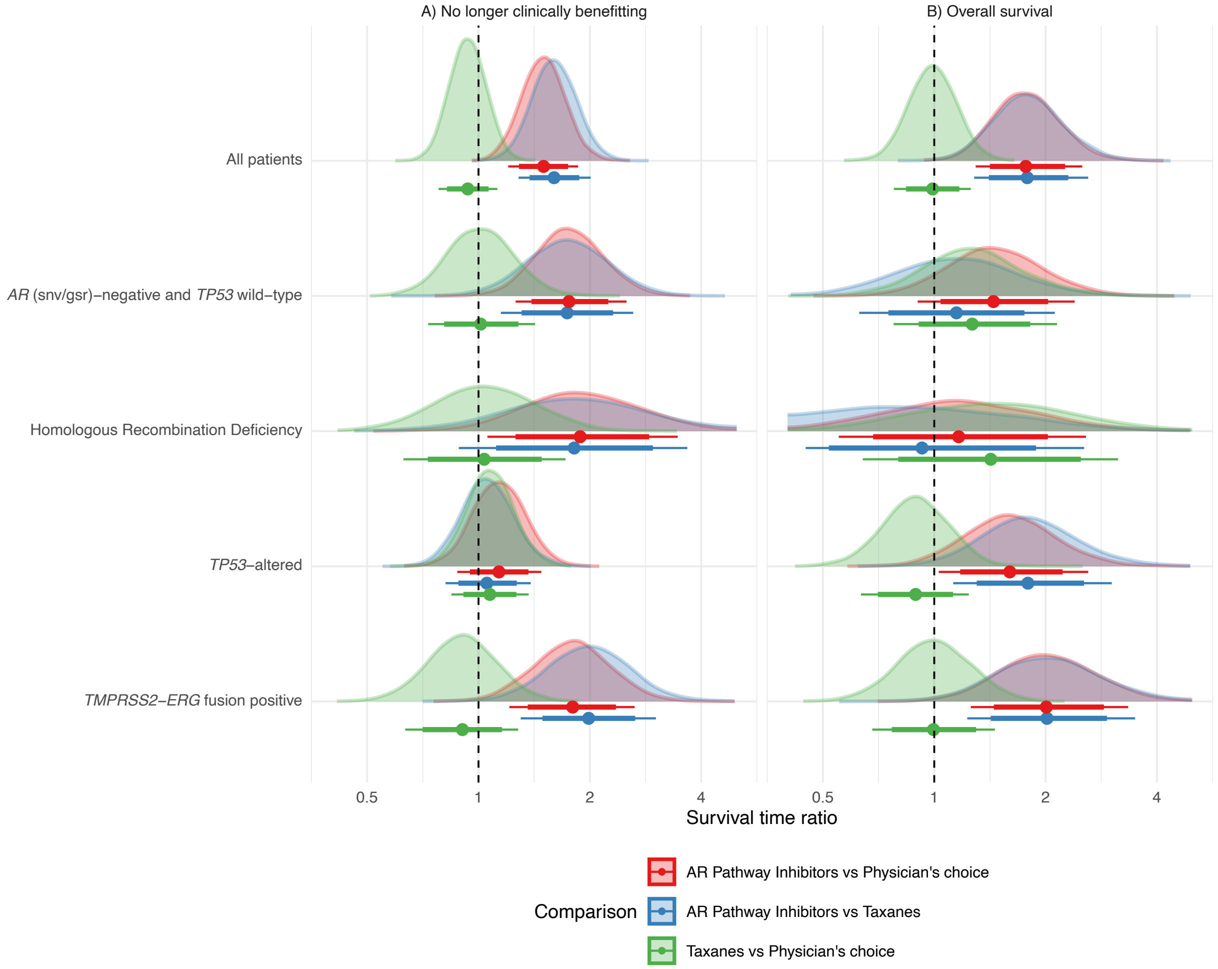

Supplementary Figure S3. Probability distributions of survival time ratio in selected biomarker signatures.

Histograms illustrating the posterior probability distributions of the survival time ratio (STR) for AR pathway inhibitors compared to physician's choice (Control) and taxanes within the five prespecified biomarker signatures. Results are provided for A) the time to no longer clinically benefitting (left), and B) overall survival (right). AR pathway inhibitors achieved the pre-specified threshold for efficacy for the time to no longer clinically benefitting across the '*all* patients' biomarker signature. STR values were estimated using Weibull accelerated failure time survival models. The forest plot below each histogram graph summarizes the posterior densities, with circles representing the median STR with thin and thick lines indicating 75% and 90% credible intervals, respectively. Additional information on number of events and total number of randomizations provided for each comparison is available in Table 2.

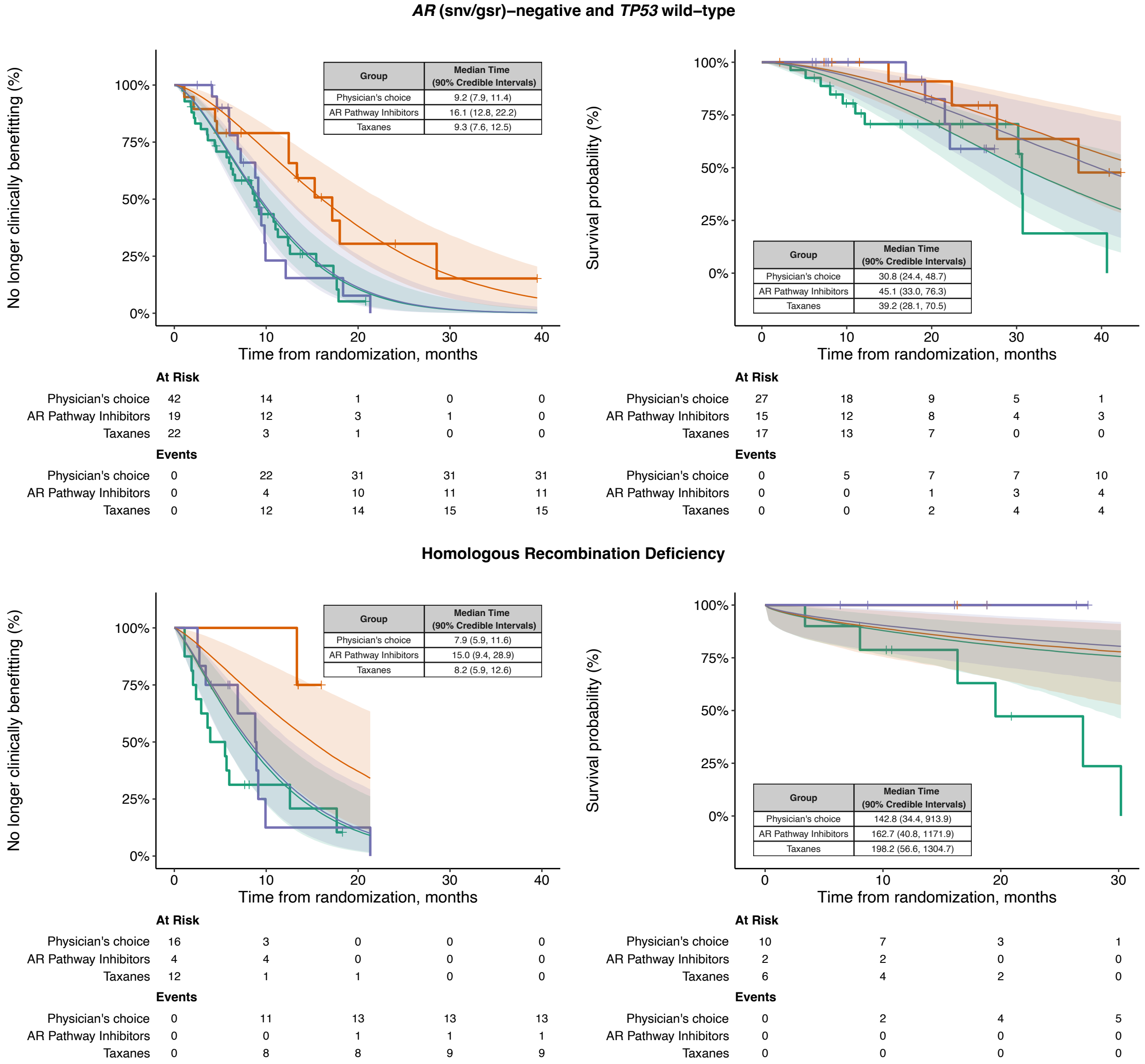

Supplementary Figure S4. Posterior survival curves and Kaplan-Meier estimates by therapy arm in selected biomarker signatures.

Posterior survival curves (smooth) and Kaplan-Meier estimates (step function) for the time to no longer clinically benefitting (left) and overall (right) survival in selected biomarker signatures. Tick marks on Kaplan-Meier curves indicate censored patients. Survival curves were estimated using Weibull accelerated failure time survival models. Smoothed colored lines denote medians of the posterior distribution, while shaded areas represent corresponding 90% credible intervals. Additional information on patients at risk, cumulative events, and median time to no longer clinically benefitting and overall survival times (in months) are provided.

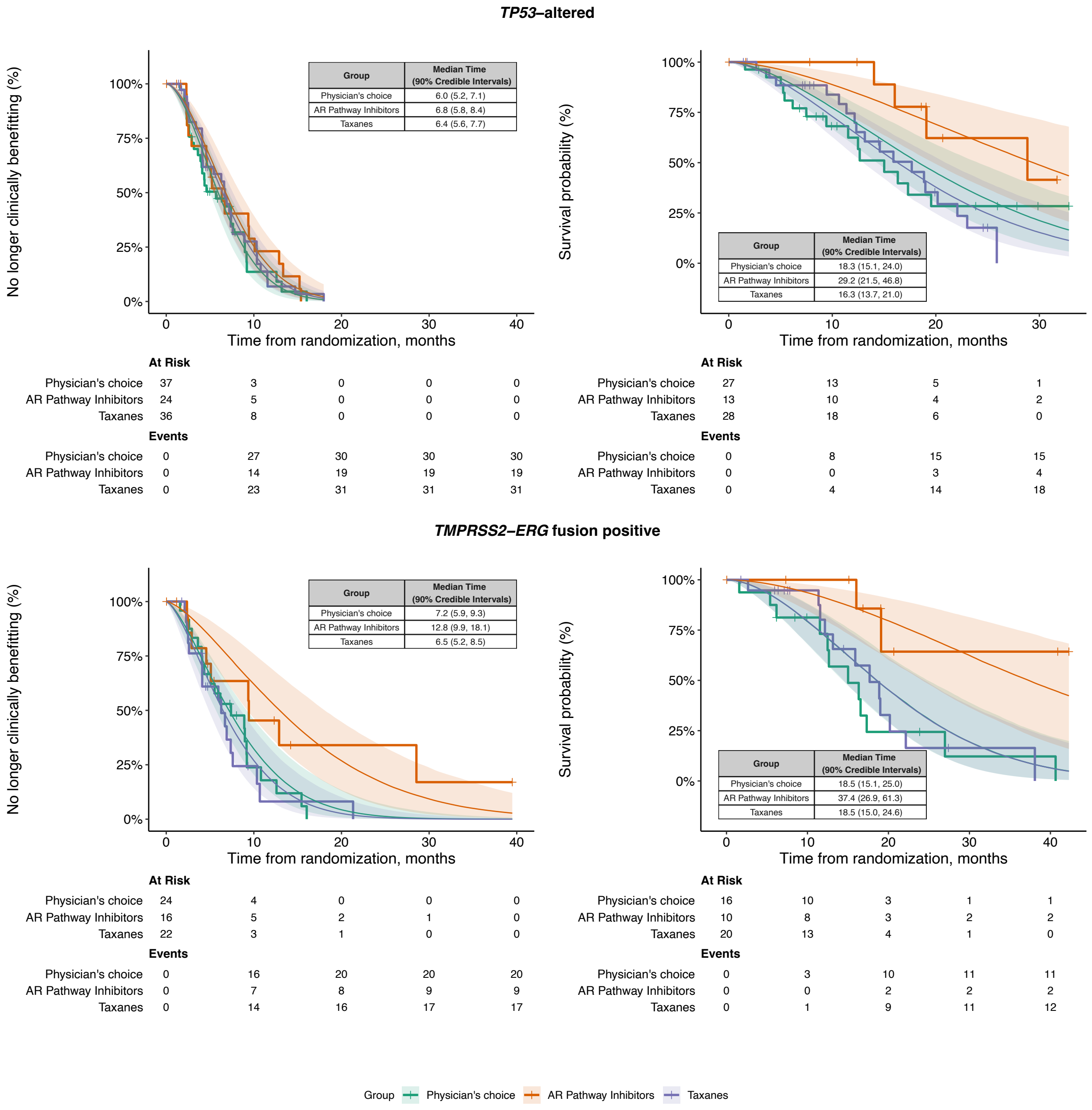

Supplementary Figure S4. Posterior survival curves and Kaplan-Meier estimates by therapy arm in selected biomarker signatures *(continued)*

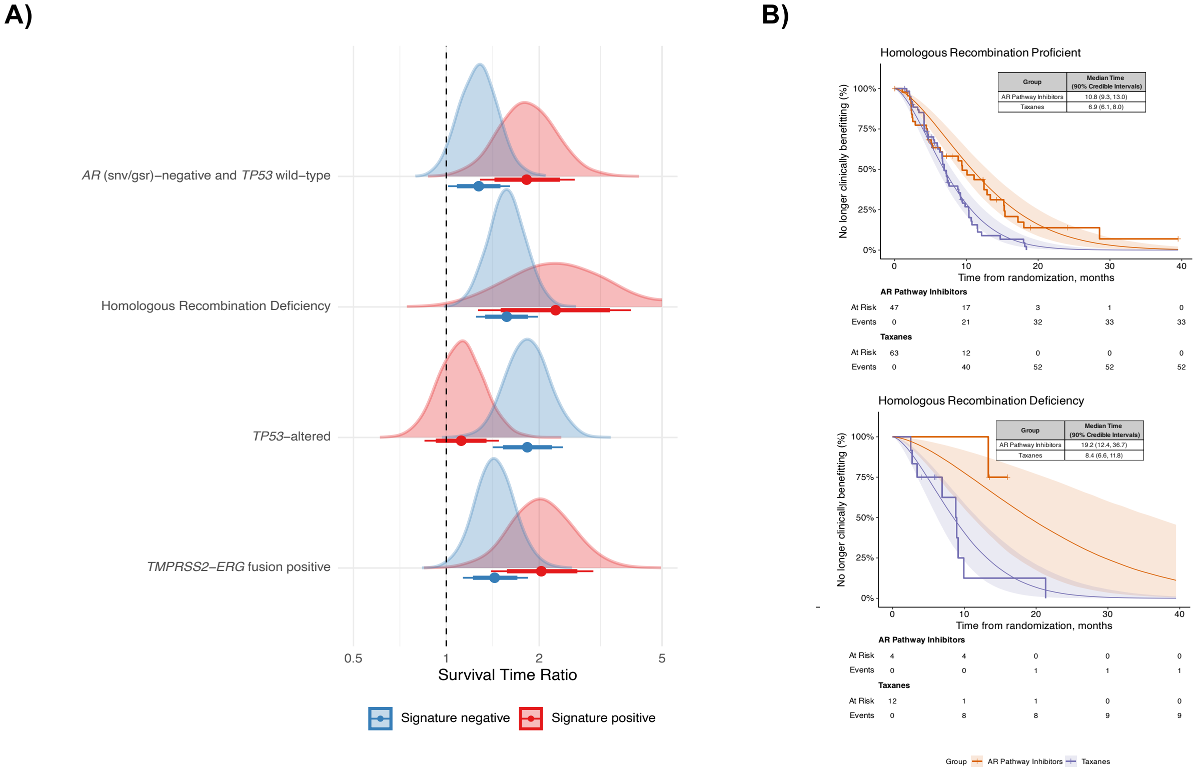

Supplementary Figure S5. Differential treatment effects according to biomarker signature status (positive vs negative).

A) Probability distributions of survival time ratio with respect to biomarker signature status (positive vs negative). Histograms plots displaying posterior probability distributions of the survival time ratio (STR) for time to no longer clinically benefitting. Comparing AR pathway inhibitors to taxanes, the efficacy is assessed with respect to the presence or absence of the predefined biomarker signatures. STR densities for patients with specific biomarker signatures are estimated using a Weibull accelerated failure time survival model with an interaction term, allowing for differential treatment effects. Below each histogram, a forest plot summarizes posterior densities, with circles representing medians and thin and thick lines indicating 75% and 90% confidence intervals, respectively. Additional information on number of events and total number of randomizations provided for each comparison is available in Supplementary Table S4. B) Posterior survival curves and Kaplan-Meier estimates by therapy arm and Homologous Recombination Deficiency (HRD) biomarker signature status (positive vs negative). Posterior survival curves (smooth) and Kaplan-Meier estimates (step function) for time to no longer clinically benefitting with AR pathway inhibitors and taxanes, for patients negative for a specific biomarker signature, i.e. homologous recombination proficient (left), and for patients positive for the respective biomarker signature, i.e. homologous recombination deficient (right). Survival curves were estimated using Weibull accelerated failure time survival models with an interaction term, enabling the assessment of differential treatment effects. The smoothed colored lines in the figure represent medians of the posterior distribution, while shaded areas depict corresponding 90% credible intervals. Additional information on patients at risk, cumulative events, and median time to no longer clinically benefitting survival times (in months) are provided.

A)

Mutations	
All coding exons	51 genes
Hotspots	27 genes
**Pharmacogenetic variants**	
SNPs	4 genes
**Copy-number alterations**	
Tiled SNP for genome-wide CNV	3128 SNPs
Directed analysis to increase sensitivty	20 genes
**Structural variation**	
Genefusions by intronic sequencing	3 genes
Gene-body sequencing (e.g. BRCA1/2)	8 genes
**Microsatellite instability & Hypermutation**	
Microsatellites	63
Hypermutation, entire design footprint	Yes
Associated genes	6
**DNA repair deficiency**	
Associated genes	16
**Total size (Mb)**	
	1.5

B)

Exonic regions targeted to detect small somatic- and germline alterations

AKT1	APC	AR	ARID1A	ARID2	ATM	ATR	BARD1	BRAF	BRCA1	BRCA2	BRIP1	CCND1	CDH1
CDK12	CDK4	CDK6	CDKN1A	CDKN1B	CDKN2A	CHD1	CHEK2	CTNNB1	CUL3	DICER1	DNMT3A	FANCA	FOXA1
FOXO1	HRAS	IDH1	JAK1	KDM6A	KEAP1	KMT2A	KMT2C	KMT2D	KRAS	MED12	MET	MGA	MLH1
MLH3	MRE11A	MSH2	MSH3	MSH6	NBN	NCOR1	NKX3-1	NRAS	PALB2	PIK3CA	PIK3CB	PIK3CD	PIK3R1
PIK3R2	PMS1	PMS2	POLD1	POLE	PTEN	RAD50	RAD51	RAD51B	RAD51C	RAD51D	RB1	RNF43	SETD2
SF3B1	SPEN	SPOP	TP53	U2AF1	XPO1	ZFHX3	ZMYM3						

Intronic and intergenic regions targeted to increase sensitivity to detect copy-number alterations

AR	AR enhancer	ATM	BRCA2	CCND1	CDK12	CDKN2A	CDKN2B	CHD1	CHEK2	FANCA	MYC	NKX3-1	PIK3CA
PIK3R1	PTEN	RB1	TP53	ZBTB16	Intergenic region between TMPRSS2 and ERG								

Intronic regions targeted to detect fusions and structural rearrangements

AR	ATM	BRAF	BRCA1	BRCA2	CDK12	ERG	PTEN	RB1	TMPRSS2	TP53

C)

Genetic biomarker	Gene(s)	Alteration type	Detailed info
AR	AR	Predefined hotspot mutations, regardless of clonality.	L702H<br>V716M<br>W742C<br>W742L<br>H875Y<br>F877L<br>T878A<br>T878S<br>F877L<br>T878A<br>M896T
		High-impact genomic structural rearrangement, regardless of clonality.<br>Copy-number alteration.	*Panel D*
HRD	ATM<br>ATR<br>BARD1<br>BRCA1<br>BRCA2<br>BRIP1<br>CDK12<br>CHEK2<br>FANCA<br>MRE11A<br>NBN<br>PALB2<br>RAD50<br>RAD51<br>RAD51B<br>RAD51C<br>RAD51D	Somatic variants:<br>Clonal high-impact genomic structural rearrangement affecting one or more exons.<br>Clonal hotspot or high-impact point mutation/indel not known to be benign.<br>Homozygous deletion detected by copy-number alteratio analysis.<br><br>Germline variants:<br>High-impact genomic structural rearrangement affecting one or more exons.<br>High-impact point mutation/indel not known to be benign.<br>Deletion of one allele detected by copy-number alteration analysis.	
TP53	TP53	Clonal high-impact genomic structural rearrangement affecting one or more exons.<br>Clonal hotspot or high-impact point mutation/indel not known to be benign.<br>Homozygous deletion detected by copy-number alteratio analysis.	
TMPRSS2-ERG fusion	TMPRSS2<br>ERG	Copy-number alteration.<br>Genomic structural rearrangement.	*Panel E*

D)

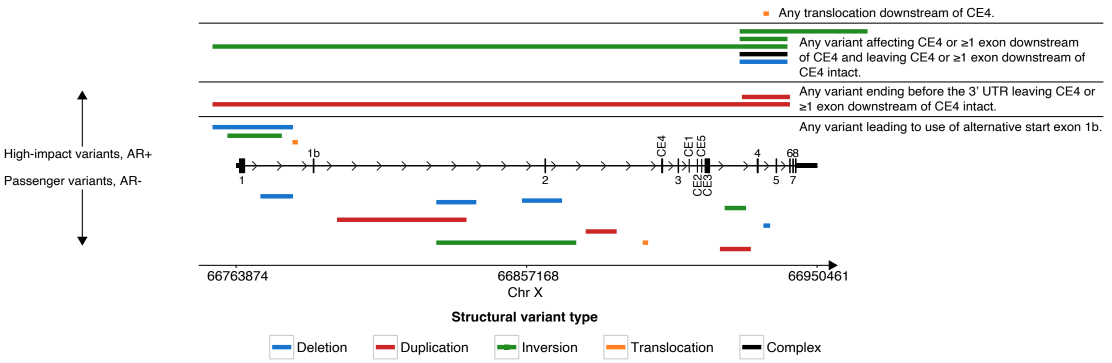

E)

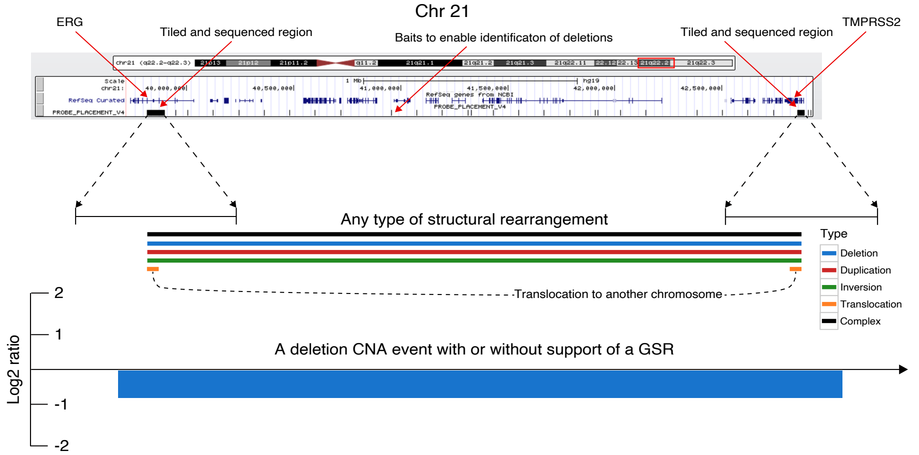

Supplementary Figure S6. The ProBio gene panel and biomarker subgroup assignments.

A) The alteration types interrogated by the ProBio gene panel and the number of genes for each type. B) The genes targeted and the primarily investigated alteration type for each gene. C) The genes and alteration types applied to determine genetic biomarker status are listed. High-impact mutations are defined as frameshift- or nonsense variants. For HRD and *TP53*, only clonal alterations, pragmatically defined as a variant allele frequency equal to or more than 25% of the ctDNA fraction (Mayrhofer et al, 2018), were accounted for. High impact structural variation and copy-number alterations affecting *AR* are described in panel D. The copy-number alterations and genomic structural rearrangements used to call the *TMPRSS2-ERG* gene fusion are described in panel E. D) Classification of structural variation and copy-number alteration in the androgen receptor. The gene body of *AR*, including cryptic exons (CE) present in non-canonical *AR* transcript, is displayed. High-impact genomic structural rearrangements and definitions classifying a patient as androgen receptor alteration positive (AR+) are shown above the *AR* gene track. Examples of genomic structural variants of unknown significance (AR-) are displayed below the *AR* gene track. Due to the presence of repetitive and non-unique regions in the *AR* gene body, only ~80% is possible to enrich and interrogate for genomic structural rearrangements. AR copy number alterations were therefore also analyzed to classify patients as AR+/-. The following copy-number alterations were applied to identify AR+ patients: copy-number alterations affecting exonic sequence downstream of cryptic exon 4 or including cryptic exon 4 with the exonic sequences upstream of cryptic exon 4 intact; copy-number alteration inactivating exon 1 with the alternative first exon 1b intact. E) Capture design and criteria for identifying the *TMPRSS2-ERG* gene fusion. The upper panel display UCSC genome browser tracks of chromosome 21 covering the ~3 Mb distance between *TMPRSS2* and *ERG*. Introns, commonly reported to harbor fusion break points were targeted. Additionally, baits were placed between *TMPRSS2* and *ERG* to enable detection of fusion events through copy-number alteration analysis. Any type of structural rearrangement connecting the target regions in *TMPRSS2* and *ERG* as well as copy-number deletions were applied to identify *TMPRSS2-ERG*+ patients.

Supplementary Table S1. Treatment received in the investigational and control arms

The table provides a descriptive overview of the treatments administered in patients who were randomized to the physician's choice (control group), AR pathway inhibitor, or taxane treatment arms.

	Physician's choice<br>N = 92	AR pathway inhibitors<br>N = 51	Taxanes<br>N = 75
**Treatment received**			
Abiraterone	16 (18%)	23 (46%)	0 (0%)
Enzalutamide	21 (23%)	27 (54%)	0 (0%)
Cabazitaxel	16 (18%)	0 (0%)	34 (45%)
Docetaxel	32 (35%)	0 (0%)	41 (55%)
Radium-223	5 (5.5%)	0 (0%)	0 (0%)
Other	1 (1.1%)	0 (0%)	0 (0%)
Unknown	1	1	0

Supplementary Table S2. The type(s) of progressive disease which ultimately resulted in the discontinuation of the treatment and the no longer clinically benefitting endpoint. All dimensions of progressive disease (i.e. biochemical (i.e. PSA), radiological, and/or clinical progression) were recorded following Prostate Cancer Working Group 3 recommendations, and eventually resulted in the clinical need/decision to terminate or change treatment, deeming a patient no longer clinically benefitting.

	Physician's choice<br>N = 92	AR pathway inhibitors<br>N = 51	Taxanes<br>N = 75
**Number of events**	69 (75%)	34 (66.7%)	61 (82.3%)
**PSA progression**			
No	12 (13%)	2 (3.9%)	5 (6.7%)
Yes	53 (58%)	32 (63%)	55 (73%)
Not evaluated	27 (29%)	17 (33%)	15 (20%)
**Radiological progression**			
No	15 (16%)	9 (18%)	13 (17%)
Yes	50 (54%)	24 (47%)	44 (59%)
Not evaluated	27 (29%)	18 (35%)	18 (24%)
**Clinical progression**			
No	29 (32%)	18 (35%)	31 (41%)
Yes	38 (41%)	17 (33%)	30 (40%)
Not evaluated	25 (27%)	16 (31%)	14 (19%)
**Number of dimensions for progression**			
0	24 (26%)	17 (33%)	15 (20%)
1	16 (17%)	7 (14%)	13 (17%)
2	31 (34%)	15 (29%)	25 (33%)
3	21 (23%)	12 (24%)	22 (29%)
**Type of progression for endpoint**			
PSA, radiological, and clinical	20 (29%)	12 (35%)	22 (36%)
PSA and radiological	18 (26%)	10 (29%)	20 (33%)
PSA and clinical	8 (12%)	3 (8.8%)	5 (8.2%)
Radiological and clinical	5 (7.2%)	2 (5.9%)	0 (0%)
Only radiological	6 (8.7%)	0 (0%)	2 (3.3%)
Only PSA	6 (8.7%)	7 (21%)	8 (13%)
Only clinical	4 (5.8%)	0 (0%)	3 (4.9%)
Death	2 (2.9%)	0 (0%)	0 (0%)
Other	0 (0%)	0 (0%)	1 (1.6%)

Supplementary Table S3. Sensitivity analysis for the time to no longer clinically benefitting and overall survival in the five prespecified biomarker signatures.

Sensitivity analysis on efficacy outcomes for time to no longer clinically benefitting (left) and overall (right) survival within the five predefined biomarker signatures (*'all'* patients, *AR* (snv/gsr)-negative and *TP53* wild-type, Homologous Recombination Deficiency, *TP53*-altered, and *TMPRSS2-ERG* fusion-positive). We contrast the primary findings (i.e. main analysis) with subgroup analyses conducted on distinct patient groups: 1) First-line patients only, 2) First-line patients who were previously treated with ADT monotherapy, and 3) Patients who did not undergo a therapy class re-challenge during ProBio. For each comparison, the posterior median survival time ratio (STR), 90% credible intervals (CrI), and the posterior probability of superiority (PPS) are provided. Additionally, we offer supplementary event/N data for each comparison, delineated separately for the therapy arm under comparison and the reference arm.

Biomarker signature or group	Subgroup	Arm (Events/N)	Reference (Events/N)	PPS	STR (90% CrI)	Arm (Events/N)	Reference (Events/N)	PPS	STR (90% CrI)
**'*All*' patients**	**Main analysis**	ARPI (34/51)	Physician's choice (69/92)	1.00	1.50 (1.20, 1.86)	ARPI (8/31)	Physician's choice (30/64)	1.00	1.77 (1.29, 2.51)
	**First line**	ARPI (17/28)	Physician's choice (34/52)	0.99	1.53 (1.15, 2.05)	ARPI (6/28)	Physician's choice (20/52)	0.98	1.67 (1.13, 2.58)
	**First line post ADT only**	ARPI (7/12)	Physician's choice (16/22)	0.99	1.66 (1.13, 2.42)	ARPI (3/12)	Physician's choice (8/22)	0.87	1.35 (0.87, 2.16)
	**No re-challenge**	ARPI (24/39)	Physician's choice (42/61)	0.99	1.48 (1.15, 1.93)	ARPI (7/30)	Physician's choice (22/53)	0.99	1.64 (1.15, 2.41)
	**Main analysis**	ARPI (34/51)	Taxanes (61/75)	1.00	1.60 (1.28, 2.01)	ARPI (8/31)	Taxanes (29/56)	1.00	1.78 (1.28, 2.61)
	**First line**	ARPI (17/28)	Taxanes (31/42)	1.00	1.81 (1.34, 2.48)	ARPI (6/28)	Taxanes (19/42)	0.99	1.89 (1.24, 3.01)
	**First line post ADT only**	ARPI (7/12)	Taxanes (15/19)	0.99	1.81 (1.21, 2.72)	ARPI (3/12)	Taxanes (6/19)	0.86	1.41 (0.83, 2.37)
	**No re-challenge**	ARPI (24/39)	Taxanes (35/43)	1.00	1.67 (1.26, 2.21)	ARPI (7/30)	Taxanes (16/35)	0.99	1.69 (1.14, 2.61)
	**Main analysis**	Taxanes (61/75)	Physician's choice (69/92)	0.27	0.94 (0.78, 1.12)	Taxanes (29/56)	Physician's choice (30/64)	0.48	0.99 (0.78, 1.26)
	**First line**	Taxanes (31/42)	Physician's choice (34/52)	0.13	0.84 (0.66, 1.08)	Taxanes (19/42)	Physician's choice (20/52)	0.26	0.89 (0.64, 1.21)
	**First line post ADT only**	Taxanes (15/19)	Physician's choice (16/22)	0.33	0.92 (0.66, 1.26)	Taxanes (6/19)	Physician's choice (8/22)	0.44	0.96 (0.65, 1.46)
	**No re-challenge**	Taxanes (35/43)	Physician's choice (42/61)	0.21	0.89 (0.70, 1.13)	Taxanes (16/35)	Physician's choice (22/53)	0.43	0.97 (0.71, 1.33)
***AR* (snv/gsr)-negative and *TP53* wild-type**	**Main analysis**	ARPI (11/19)	Physician's choice (31/42)	1.00	1.76 (1.26, 2.51)	ARPI (4/15)	Physician's choice (11/27)	0.90	1.45 (0.90, 2.40)
	**First line**	ARPI (7/13)	Physician's choice (14/21)	0.94	1.56 (0.99, 2.52)	ARPI (2/13)	Physician's choice (6/21)	0.70	1.26 (0.59, 2.62)
	**First line post ADT only**	ARPI (4/6)	Physician's choice (7/10)	0.91	1.56 (0.91, 2.59)	ARPI (1/6)	Physician's choice (3/10)	0.59	1.11 (0.50, 2.46)
	**No re-challenge**	ARPI (9/17)	Physician's choice (22/29)	0.99	1.73 (1.19, 2.63)	ARPI (3/14)	Physician's choice (9/25)	0.83	1.39 (0.77, 2.44)
	**Main analysis**	ARPI (11/19)	Taxanes (15/22)	0.99	1.74 (1.15, 2.62)	ARPI (4/15)	Taxanes (4/17)	0.64	1.14 (0.61, 2.11)
	**First line**	ARPI (7/13)	Taxanes (10/15)	0.95	1.78 (1.01, 3.10)	ARPI (2/13)	Taxanes (3/15)	0.61	1.17 (0.42, 3.13)
	**First line post ADT only**	ARPI (4/6)	Taxanes (8/10)	0.95	1.80 (1.00, 3.19)	ARPI (1/6)	Taxanes (2/10)	0.55	1.08 (0.37, 3.06)
	**No re-challenge**	ARPI (9/17)	Taxanes (12/16)	0.98	1.80 (1.12, 2.91)	ARPI (3/14)	Taxanes (3/14)	0.64	1.17 (0.54, 2.36)
	**Main analysis**	Taxanes (15/22)	Physician's choice (31/42)	0.53	1.01 (0.73, 1.42)	Taxanes (4/17)	Physician's choice (11/27)	0.79	1.27 (0.78, 2.15)
	**First line**	Taxanes (10/15)	Physician's choice (14/21)	0.31	0.87 (0.56, 1.37)	Taxanes (3/15)	Physician's choice (6/21)	0.57	1.08 (0.53, 2.18)
	**First line post ADT only**	Taxanes (8/10)	Physician's choice (7/10)	0.31	0.87 (0.53, 1.40)	Taxanes (2/10)	Physician's choice (3/10)	0.53	1.03 (0.48, 2.23)
	**No re-challenge**	Taxanes (12/16)	Physician's choice (22/29)	0.43	0.96 (0.66, 1.44)	Taxanes (3/14)	Physician's choice (9/25)	0.70	1.19 (0.67, 2.15)
**Homologous Recombination Deficiency**	**Main analysis**	ARPI (1/4)	Physician's choice (13/16)	0.96	1.89 (1.06, 3.53)	ARPI (0/2)	Physician's choice (6/10)	0.63	1.15 (0.52, 2.57)
	**First line**	ARPI (0/2)	Physician's choice (6/9)	0.76	1.36 (0.65, 2.93)	ARPI (0/2)	Physician's choice (5/9)	0.57	1.09 (0.50, 2.46)
	**First line post ADT only**	ARPI (0/0)	Physician's choice (3/5)	NA	NA (NA, NA)	ARPI (0/0)	Physician's choice (3/5)	NA	NA (NA, NA)
	**No re-challenge**	ARPI (1/4)	Physician's choice (10/13)	0.92	1.69 (0.91, 3.33)	ARPI (0/2)	Physician's choice (6/10)	0.60	1.14 (0.52, 2.51)
	**Main analysis**	ARPI (1/4)	Taxanes (9/12)	0.92	1.83 (0.88, 3.89)	ARPI (0/2)	Taxanes (0/6)	0.39	0.82 (0.27, 2.46)
	**First line**	ARPI (0/2)	Taxanes (3/5)	0.67	1.30 (0.48, 3.47)	ARPI (0/2)	Taxanes (0/5)	0.44	0.90 (0.28, 2.79)
	**First line post ADT only**	ARPI (0/0)	Taxanes (1/1)	NA	NA (NA, NA)	ARPI (0/0)	Taxanes (0/1)	NA	NA (NA, NA)
	**No re-challenge**	ARPI (1/4)	Taxanes (5/6)	0.85	1.61 (0.73, 3.72)	ARPI (0/2)	Taxanes (0/5)	0.40	0.83 (0.28, 2.53)
	**Main analysis**	Taxanes (9/12)	Physician's choice (13/16)	0.55	1.04 (0.62, 1.72)	Taxanes (0/6)	Physician's choice (6/10)	0.75	1.42 (0.62, 3.20)
	**First line**	Taxanes (3/5)	Physician's choice (6/9)	0.55	1.05 (0.51, 2.15)	Taxanes (0/5)	Physician's choice (5/9)	0.66	1.23 (0.54, 2.85)
	**First line post ADT only**	Taxanes (1/1)	Physician's choice (3/5)	NA	NA (NA, NA)	Taxanes (0/1)	Physician's choice (3/5)	NA	NA (NA, NA)
	**No re-challenge**	Taxanes (5/6)	Physician's choice (10/13)	0.56	1.06 (0.58, 1.90)	Taxanes (0/5)	Physician's choice (6/10)	0.73	1.36 (0.60, 3.05)
***TP53*-altered**	**Main analysis**	ARPI (19/24)	Physician's choice (30/37)	0.78	1.14 (0.88, 1.48)	ARPI (4/13)	Physician's choice (15/27)	0.96	1.60 (1.03, 2.61)
	**First line**	ARPI (9/12)	Physician's choice (17/23)	0.84	1.21 (0.88, 1.71)	ARPI (4/12)	Physician's choice (11/23)	0.87	1.41 (0.87, 2.43)
	**First line post ADT only**	ARPI (3/5)	Physician's choice (7/9)	0.74	1.20 (0.76, 2.00)	ARPI (2/5)	Physician's choice (3/9)	0.48	0.98 (0.46, 2.15)
	**No re-challenge**	ARPI (14/18)	Physician's choice (16/23)	0.72	1.12 (0.81, 1.55)	ARPI (4/13)	Physician's choice (10/20)	0.91	1.47 (0.92, 2.46)
	**Main analysis**	ARPI (19/24)	Taxanes (31/36)	0.63	1.05 (0.81, 1.38)	ARPI (4/13)	Taxanes (18/28)	0.98	1.79 (1.13, 3.03)
	**First line**	ARPI (9/12)	Taxanes (16/21)	0.82	1.21 (0.86, 1.75)	ARPI (4/12)	Taxanes (13/21)	0.96	1.80 (1.02, 3.27)
	**First line post ADT only**	ARPI (3/5)	Taxanes (6/8)	0.73	1.22 (0.71, 2.14)	ARPI (2/5)	Taxanes (3/8)	0.53	1.04 (0.36, 2.82)
	**No re-challenge**	ARPI (14/18)	Taxanes (18/21)	0.68	1.10 (0.78, 1.55)	ARPI (4/13)	Taxanes (10/16)	0.94	1.65 (0.96, 2.90)
	**Main analysis**	Taxanes (31/36)	Physician's choice (30/37)	0.69	1.07 (0.84, 1.37)	Taxanes (18/28)	Physician's choice (15/27)	0.28	0.89 (0.63, 1.24)
	**First line**	Taxanes (16/21)	Physician's choice (17/23)	0.50	1.00 (0.75, 1.34)	Taxanes (13/21)	Physician's choice (11/23)	0.18	0.79 (0.52, 1.21)
	**First line post ADT only**	Taxanes (6/8)	Physician's choice (7/9)	0.48	0.99 (0.66, 1.50)	Taxanes (3/8)	Physician's choice (3/9)	0.46	0.95 (0.45, 2.02)
	**No re-challenge**	Taxanes (18/21)	Physician's choice (16/23)	0.55	1.02 (0.75, 1.39)	Taxanes (10/16)	Physician's choice (10/20)	0.32	0.89 (0.58, 1.36)
***TMPRSS2-ERG* fusion positive**	**Main analysis**	ARPI (9/16)	Physician's choice (20/24)	0.99	1.80 (1.21, 2.64)	ARPI (2/10)	Physician's choice (12/16)	0.99	2.01 (1.26, 3.38)
	**First line**	ARPI (4/9)	Physician's choice (10/12)	0.99	2.16 (1.30, 3.48)	ARPI (2/9)	Physician's choice (9/12)	0.95	1.78 (1.01, 3.24)
	**First line post ADT only**	ARPI (2/6)	Physician's choice (6/7)	0.98	2.13 (1.18, 3.76)	ARPI (1/6)	Physician's choice (5/7)	0.88	1.59 (0.83, 3.05)
	**No re-challenge**	ARPI (7/13)	Physician's choice (13/16)	0.99	1.86 (1.18, 2.91)	ARPI (2/10)	Physician's choice (9/13)	0.98	1.86 (1.12, 3.24)
	**Main analysis**	ARPI (9/16)	Taxanes (17/22)	0.99	1.99 (1.30, 3.02)	ARPI (2/10)	Taxanes (12/20)	0.99	2.02 (1.23, 3.55)
	**First line**	ARPI (4/9)	Taxanes (9/13)	0.99	2.24 (1.26, 3.80)	ARPI (2/9)	Taxanes (6/13)	0.89	1.64 (0.81, 3.29)
	**First line post ADT only**	ARPI (2/6)	Taxanes (7/8)	0.98	2.44 (1.26, 4.66)	ARPI (1/6)	Taxanes (4/8)	0.85	1.62 (0.74, 3.52)
	**No re-challenge**	ARPI (7/13)	Taxanes (11/15)	0.98	1.89 (1.15, 3.11)	ARPI (2/10)	Taxanes (8/14)	0.96	1.82 (1.03, 3.35)
	**Main analysis**	Taxanes (17/22)	Physician's choice (20/24)	0.31	0.90 (0.63, 1.28)	Taxanes (12/20)	Physician's choice (12/16)	0.49	0.99 (0.68, 1.46)
	**First line**	Taxanes (9/13)	Physician's choice (10/12)	0.45	0.97 (0.61, 1.51)	Taxanes (6/13)	Physician's choice (9/12)	0.62	1.09 (0.65, 1.83)
	**First line post ADT only**	Taxanes (7/8)	Physician's choice (6/7)	0.33	0.87 (0.51, 1.48)	Taxanes (4/8)	Physician's choice (5/7)	0.47	0.98 (0.54, 1.81)
	**No re-challenge**	Taxanes (11/15)	Physician's choice (13/16)	0.47	0.98 (0.64, 1.51)	Taxanes (8/14)	Physician's choice (9/13)	0.55	1.03 (0.67, 1.59)

Supplementary Table S4. Differential treatment effects in the time to no longer clinically benefitting for AR pathway inhibitors versus taxanes, stratified by pre-specified biomarker signature status.

An interaction analysis was performed using Weibull accelerated failure time models to estimate survival time ratio (STR) when comparing AR pathway inhibitors to Taxanes separately for patients who were positive or negative for a specific biomarker signature. Provided are the number of events and total number of randomizations (Events/N) for each therapy arm. Comparing the AR pathway inhibitors to Taxanes median survival time ratios and corresponding 90% Credible Intervals for each biomarker signature status combination are provided. Forest plot visualizations for the STRs for biomarker signature-positive and -negative groups are available in Supplementary Figure S5A. The differential treatment effect was quantified by means of the STR ratio of biomarker signature-positive over -negative status, which represents the in- or decrease in STR between biomarker-positive and -negative patients.

Biomarker signature	Events/N				STR		
	Biomarker signature negative		Biomarker signature positive		Biomarker signature negative	Biomarker signature positive	
	Taxanes	AR pathway inhibitors	Taxanes	AR pathway inhibitors			STR ratio
AR **(snv/gsr)-negative and** ***TP53*** **wild-type**	46/53	23/32	15/22	11/19	1.27 (1.01, 1.61)	1.82 (1.29, 2.60)	1.44 (1.05, 1.95)
**Homologous Recombination Deficiency**	52/63	33/47	9/12	1/4	1.57 (1.25, 1.98)	2.28 (1.27, 4.26)	1.46 (0.92, 2.34)
TP53**-altered**	30/39	15/27	31/36	19/24	1.83 (1.41, 2.39)	1.12 (0.85, 1.48)	0.61 (0.46, 0.80)
TMPRSS2-ERG **fusion positive**	44/53	25/35	17/22	9/16	1.43 (1.13, 1.84)	2.03 (1.39, 3.00)	1.42 (1.03, 1.97)

List of ProBio investigators

Author name	Academic degree	Affiliations
Bram De Laere	PhD	Department of Human Structure and Repair Ghent University, Ghent, Belgium<br><br>Cancer Research Institute Ghent (CRIG), Ghent, Belgium<br><br>Department of Medical Epidemiology and Biostatistics, Karolinska Institutet, Stockholm, Sweden
Alessio Crippa	PhD	Department of Medical Epidemiology and Biostatistics, Karolinska Institutet, Stockholm, Sweden
Andrea Discacciati	PhD	Department of Medical Epidemiology and Biostatistics, Karolinska Institutet, Stockholm, Sweden
Berit Larsson	MSc	Department of Medical Epidemiology and Biostatistics, Karolinska Institutet, Stockholm, Sweden
Maria Persson	MSc	Department of Medical Epidemiology and Biostatistics, Karolinska Institutet, Stockholm, Sweden
Susanne Johansson	BSc	Department of Medical Epidemiology and Biostatistics, Karolinska Institutet, Stockholm, Sweden
Sanne D'hondt	PhD	Health, Innovation and Research Institute (Clinical Trial Unit), Ghent, Belgium
Rebecka Bergström	MSc	Department of Medical Epidemiology and Biostatistics, Karolinska Institutet, Stockholm, Sweden
Venkatesh Chellappa	MSc	Department of Medical Epidemiology and Biostatistics, Karolinska Institutet, Stockholm, Sweden

Markus Mayrhofer	PhD	National Bioinformatics Infrastructure Sweden, Science for Life Laboratory, Department of Cell and Molecular Biology, Uppsala University, Uppsala, Sweden
Mahsan Banijamali	PhD	Department of Medical Epidemiology and Biostatistics, Karolinska Institutet, Stockholm, Sweden
Anastasijia Kotsalaynen	MSc	Department of Medical Epidemiology and Biostatistics, Karolinska Institutet, Stockholm, Sweden
Céline Schelstraete	BSc	Department of Urology, University Hospital Ghent, Ghent, Belgium
Jan Pieter Vanwelkenhuyzen	MSc	Department of Human Structure and Repair Ghent University, Ghent, Belgium<br><br>Cancer Research Institute Ghent (CRIG), Ghent, Belgium<br><br>Translational Oncogenomics and Bio-informatics, VIB - UGent center for medical biotechnology<br><br>Department of Medical Epidemiology and Biostatistics, Karolinska Institutet, Stockholm, Sweden
Marie Hjälm-Eriksson	MD, PhD	Department of Oncology, Capio Saint Göran's Hospital, Stockholm, Sweden
Linn Pettersson	MD, PhD	Department of Oncology, Länssjukhuset Ryhov, Jönköping, Sweden
Anders Ullén	MD, PhD	Department of Oncology, Karolinska University Hospital, Stockholm, Sweden
Nicolaas Lumen	MD, PhD	Department of Urology, University Hospital Ghent, Ghent, Belgium
Gunilla Enblad	MD, PhD	Department of Oncology, Uppsala University

		Hospital, Uppsala, Sweden
Camilla Thellenberg Karlsson	MD, PhD	Department of Oncology, University Hospital of Umeå, Umeå, Sweden
Elin Jänes	MD, PhD	Department of Oncology, Sundsvalls sjukhus, Sundsvall, Sweden
Johan Sandzén	MD, PhD	Department of Oncology, Centralsjukhuset Karlstad, Karlstad, Sweden
Peter Schatteman	MD	Department of Urology, Onze Lieve Vrouwziekenhuis, Aalst, Belgium
Maria Nyre Vigmostad	MD, PhD	Department of Oncology, Stavanger University Hospital, Stavanger, Norway
Martha Olsson	MD, PhD	Department of Oncology, Centrallasarettet Växjö, Växjö, Sweden
Christophe Ghysel	MD	Department of Urology, AZ Sint Jan Brugge-Oostende AV, Brugge, Belgium
Brieuc Sautois	MD, PhD	Department of Oncology, Le Centre hospitalier universitaire de Liège (CHU de Liège - site Sart Tilman), Liège, Belgium
Wendy De Roock	MD, PhD	Department of Oncology, Ziekenhuis Oost-Limburg, Genk, Belgium
Siska Van Bruwaene	MD, PhD	Department of Urology, AZ Groeninge, Kortrijk, Belgium
Mats Anden	MD, PhD	Department of Oncology, Länssjukhuset i Kalmar, Kalmar, Sweden
Ingrida Verbiene	MD, PhD	Department of Oncology, Falu lasarett, Falu, Sweden
Daan De Maeseneer	MD, PhD	Department of Oncology, AZ Sint-Lucas, Brugge, Belgium

Els Everaert	MD	Department of Oncology, Vitaz campus Sint-Niklaas Lodewijk, Sint-Niklaas, Belgium
Jochen Darras	MD	Department of Urology, AZ Damiaan, Oostende, Belgium
Bjørg Y. Aksnessether	MD, PhD	Department of Oncology, Ålesund Hospital, Ålesund, Norway
Daisy Luyten	MD	Department of Oncology, Virga Jessa, Hasselt, Belgium
Michiel Strijbos	MD, PhD	Department of Oncology, GZA Sint-Augustinus, Antwerpen, Belgium
Ashkan Mortezavi	MD, PhD	Universitätsspital Basel, Basel, Switzerland
Jan Oldenburg	MD, PhD	Akershus University Hospital, Nordbyhagen, Norway
Piet Ost	MD, PhD	Department of Human Structure and Repair Ghent University, Ghent, Belgium<br><br>Department of Radiation Oncology, GZA Sint-Augustinus, Antwerpen, Belgium
Martin Eklund	PhD	Department of Medical Epidemiology and Biostatistics, Karolinska Institutet, Stockholm, Sweden
Henrik Grönberg	MD, PhD	Department of Medical Epidemiology and Biostatistics, Karolinska Institutet, Stockholm, Sweden<br><br>Medicinskt ansvarig Prostatacancer Centrum, Kirurgkliniken, Capio S:t Görans Sjukhus, Stockholm, Sweden
Johan Lindberg	PhD	Department of Medical Epidemiology and Biostatistics, Science for Life Laboratory, Karolinska Institutet, Stockholm, Sweden

List of sites, co-workers, and staff contributing in ProBio

Karolinska Institute

Murugan, Sarath
Carlberg, Konstantin
Maniram, Karthick

Capio St Göran

Ekefjärd, Emma
Grönberg, Henrik
(Hallin, Charlotta)
Hjälm-Eriksson, Marie
Holmsten, Karin
Hovstadius, Malin
Modin, Viktoria
Männikkö, Veera
Nygren, Lillemor
Rasch-Westin, Martina
Sofiadis, Anastasios
Wärnberg, Lena

Länssjukhuset Ryhov

Jakobsson, Terese
Karlsson, Therese I.
Papanatoniou, Dimitrios
Pettersson, Linn
Thelin, Bo
Wennerholm, Sara

Akademiska sjukhuset

Emanuelsson, Mia
Enblad, Gunilla
Hillbom, Sara
Laurell, Anna
Larsson Enhörning, Pamela
Vandeputte, Sophie

Karolinska University Hospital

Castellanos, Enrique Nino
Cohn Cedermark, Gabriella
Corestav, Lisa
Costa Svedman, Fernanda
Fines, Katrin
Gorzov, Petr
Hallgren, Amanda
Hammarlund, Katarina
Jawdat, Faith
Majumder, Khairul
Nord, Carina
Ullen, Anders

University Hospital Ghent

Baeyens, Ruth
Bas, Sabrina
Beulque, Deborah
Bultijnck, Renée
Claeys, Wietse
Clue, Ann
Decruyenaere, Alexander
De Laere, Bram
De Letter, Rita
De Maeseneer, Daan
De Peysseleir, Katelijne
De Stommeleir, Els
Donck, Eva
Fonteyne, Valerie
Lambert, Edouard
Lumen, Nicolaas
Muyllaert, Anne
Ost, Piet
Poppe, Lindsay
Reis Guerreiro, Jorge
Rottey, Sylvie
Schelstraete, Céline
Spiessens, An
Vanderstichele, Nancy
Vanneste, Ben
Van Praet, Charles
Vansteelant, Lore
Vanwelkenhuzen, Jan-Pieter

Norrlands Universitetssjukhus

Martin Hellström
Forsell, Johan
Orrvik Olsson, Birgitta
Söderkvist, Karin
Söderman, Linda
Thellenberg, Camilla
Thomasson, Marcus

Länssjukhuset i Sundsvall

Aziz, Athir
Beckman, Lars
Bilander, Elin
Birgersson, Anna
Eliasson, Carina
Flygare, Petra
Hansén, Maria
Hashim, Hashim
Jonsson, Emma
Jänes, Elin
Lind, Helene
Lundström, Anders
Mårtensson, Carina
Notstam, Kristina
Näslund, Christin
Rhodin, Hanna
Sjöström, Emma
Waller, Elisabet
Wennstig, Anna-Karin
Wiljelöf, Joakim

Centralsjukhuset i Karlstad

Bonde, Kristina
Carlhed, Rickard
Sandzén, Johan
Sätherberg, Eva
Törnkvist, Karin

OLV Ziekenhuis Aalst

Debrouwer ,Annelies
De Groote, Ruben
De Man, Elsie
De Naeyer, Geert
De Waele, Laurien
Dhondt ,Frederik
Ghijsels, Katia
Merckx, Dorien
Schatteman, Peter
Tombeur ,Sofie
Van Varenbergh, Kristel

Stavanger Universitetssykehus

Elve, Inger Benedicte
Galta, Oda
Holta, Kjersti
Nyre Vigmostad, Maria
Svilosen, Åse

Akershus Universitetssykehus

Aas Sviland, Torunn
Lerstad, Lilli Martine
Minh Vu, Khan
Oldenburg, Jan
Syed, Moshan
Tyskø Sletbak, Tone

Växjö Lasarett

Kalin, Erik
Olsson, Martha
Rudendal, Emma
Svensson, Kristina M.
Wendt, Anna
Wiltz, Hans-Jürgen

AZ Sint-Jan Brugge

Brouwers, Barbara
Develter, Thijs
Ghysel, Christophe
Slock, Angelique
Uvin, Pieter
Van Oyen, Els
Vantieghem, Sophie

University Hospital Luik, CHU Liège

Catot, Amandine
Collinge, Astrid
Denis, Chloe
Evrard, Vanina
Freres, Pierre
Gennigens, Christine
Gonne, Elodie
Lecocq, Marie
Lousberg, Laurence
Mahioui, Layla
Marchal, Nathalie
Nicolaers, Laurence
Poncin, Aurelie
Sautois, Brieuc
Schroeder, Helene
Troisfontaine, Florence
Wéra, Odile

Ziekenhuis Oost-Limburg

Cornillie, Jasmien
De Roock, Wendy
Eerlings, Christophe
Tuerlinckx, Charlotte

AZ Groeninge

Beels, Elodie
Billiet, Ignace
Dekeyser, Ingrid
Demets, Kim
Lesage, Karl
Mullie, Nancy
Van Bruwaene, Siska
Vandeputte, Lieselotte
Verleyen, Pieter
Veys, Ralf
Werbrouck, Patrick

Länssjukhuset Kalmar

Andén, Mats
Fust, Lisa
Jung, Helen
Larsson, Emma
Wojtyna, Elzbieta
Vass, Nandor

Falu Lasarett

Hejll, Emma
Robertsson, Maria
Rosen, Marie
Verbiene, Ingrida

AZ Sint Lucas Brugge

Baekelandt, Frederic
De Maeseneer, Daan
Maerten, Tania
Mommerency, Lislot
Pérez, Noé

AZ Nikolaas

Everaert, Els
De Brabander, Isabelle
De Corte, Veerle
Deleu, Ines
Dockx, Yanina
Kruse, Vibeke
Lamot, Caroline
Lybaert, Willem
Reinders, Noortje
Vandenabbeele, Anske
Van de Walle, Mieke
Van Hoyweghen, Luna
Van Remortel, Anneleen
Vunic, Helga

AZ Damiaan

Darras, Jochen
Houthoofdt, Ruben

Lobbesstael, Nele
Mattelaer, Pieter
Ponette, Diederik
Vandenbroucke, Louise

Ålesund sjukehus

Aksnessæther, Bjørg Y.
Nøttveit Haabet, Rita
Indrebø, Gunnar
Kvammen, Øivind
Rudberg Rusten, Ida

Jessa Ziekenhuis

Joosens, Eric
Luyten, Daisy
Meubis, Jeroen
Requile, Annelies
Rummens, Jean-Luc

Stijven, Patrick
Wessels, Tim

GZA Sint-Augustinus

Bouazza, Ousama
Claeys, Kris
Dirix, Luc
Horemans, Jo
Lavaerts, Hilde
Mahieu, Hanne
Mignon, Sasha
Nelissen, Wouter
Ost, Piet
Provè, Annemie
Rutten, Annemie
Strijbos Michiel
Traets, Kathleen
van Cann ,Thomas
Van den Mooter, Tom
Van Extergem, An
Van der Eycken, Jan
Van der Eycken, Sarah
Vrancken, Sophie
Voet, Maxime

List of funding organizations and industry collaborators supporting ProBio

Funding organizations

Sweden

ALF Medicine (Region Stockholm clinical research grant)
- Period: 01/01/2020-31/12/2022
- Project-id: 20190087

Swedish Cancer Society
- Period: 01/01/2022 – 31/12/2024
- Project-id: 21 1610 P

Swedish Research Council
- Period: 01/01/2022-31/12/2025
- Project-id: 2021-00331

Belgium

Stand up to Cancer - Flemish Cancer Society (Kom op tegen Kanker)
- Period: 01/01/2020-31/01/2025
- Project-id: STI.VLK.2020.0006.01

Stand up to Cancer - Flemish Cancer Society (Kom op tegen Kanker)
- Period: 01/03/2022-31/03/2026
- Project-id: STI.VLK.2022.0005.01

Switzerland

Krebsliga beider Basel
- Period: 06/2022 - 06/2024
- Project-id: KLbB-5580-02-2022

Industry collaborators providing support to ProBio

The Janssen Pharmaceutical Companies of Johnson & Johnson

- Type of support: financial & drug
- Investigational ProBio arm: Niraparib in combination with Abiraterone Acetate

AstraZeneca

- Type of support: financial & drug
- Investigational ProBio arm: Capivasertib in combination with Docetaxel

References in Supplementary Appendix:

1. Li H. Aligning sequence reads, clone sequences and assembly contigs with BWA-MEM [Internet]. arXiv [q-bio.GN]. 2013;Available from: http://arxiv.org/abs/1303.3997

2. Van der Auwera GA, O'Connor BD. Genomics in the Cloud: Using Docker, GATK, and WDL in Terra. "O'Reilly Media, Inc."; 2020.

3. Benjamin D, Sato T, Cibulskis K, Getz G, Stewart C, Lichtenstein L. Calling Somatic SNVs and Indels with Mutect2 [Internet]. bioRxiv. 2019 [cited 2023 Oct 2];861054. Available from: https://www.biorxiv.org/content/10.1101/861054

4. Saunders CT, Wong WSW, Swamy S, Becq J, Murray LJ, Cheetham RK. Strelka: accurate somatic small-variant calling from sequenced tumor-normal sample pairs. Bioinformatics 2012;28(14):1811–7.

5. Lai Z, Markovets A, Ahdesmaki M, et al. VarDict: a novel and versatile variant caller for next-generation sequencing in cancer research. Nucleic Acids Res 2016;44(11):e108.

6. Koboldt DC, Zhang Q, Larson DE, et al. VarScan 2: somatic mutation and copy number alteration discovery in cancer by exome sequencing. Genome Res 2012;22(3):568–76.

7. Fang LT, Afshar PT, Chhibber A, et al. An ensemble approach to accurately detect somatic mutations using SomaticSeq. Genome Biol 2015;16(1):197.

8. McLaren W, Gil L, Hunt SE, et al. The Ensembl Variant Effect Predictor. Genome Biol 2016;17(1):122.

9. Mayrhofer M, De Laere B, Whitington T, et al. Cell-free DNA profiling of metastatic prostate cancer reveals microsatellite instability, structural rearrangements and clonal hematopoiesis. Genome Med 2018;10(1):85.

10. Talevich E, Shain AH, Botton T, Bastian BC. CNVkit: Genome-Wide Copy Number Detection and Visualization from Targeted DNA Sequencing. PLoS Comput Biol 2016;12(4):e1004873.

11. Riester M, Singh AP, Brannon AR, et al. PureCN: copy number calling and SNV classification using targeted short read sequencing. Source Code Biol Med 2016;11:13.

12. Robinson JT, Thorvaldsdóttir H, Winckler W, et al. Integrative genomics viewer. Nat Biotechnol 2011;29(1):24–6.

13. Barnell EK, Ronning P, Campbell KM, et al. Standard operating procedure for somatic variant refinement of sequencing data with paired tumor and normal samples. Genet Med 2019;21(4):972–81.

ProBio (NCT03903835, v4.1) trial protocol

PROTOCOL SYNOPSIS
Trial title:
**ProBio:**<br>**An outcome-adaptive and randomised multi-arm biomarker driven study in patients with metastatic prostate cancer**

Coordinating Investigator and Sponsor's representative

Henrik Grönberg
Karolinska Institutet

Trial Title

ProBio: An outcome-adaptive and randomised multi-arm biomarker driven study in patients with metastatic prostate cancer

**Trial code**	**EudraCT No**
ProBio	2018-002350-78<br>**ClinicalTrials.gov No**<br>NCT03903835

Coordinating Investigator

Professor Henrik Grönberg, Department of Medical Epidemiology and Biostatistics, Karolinska Institutet, Sweden

Trial centre(s)

ProBio is an international and multicentre study with patient accrual in

- Sweden
- Belgium
- Norway
- Switzerland

Detailed information on participating sites is provided in the Trial Conduct Supplement *'TRIAL ADMINISTRATION, INVESTIGATORS AND SITES'*.

**Trial period**	**Phase of development**
Estimated date of first patient included Q1 2019<br>Estimated date of last patient completed: Q4 2026	Phase III

Objectives and endpoints

Primary objective and endpoint

**Objective**	**Endpoint**
Evaluation of the clinical effectiveness of treatment class selection based on a biomarker signature derived from circulating tumor DNA (ctDNA) or tumor tissue DNA by improving Progression Free Survival (PFS) compared to standard-of-care (SOC) in patients with metastatic hormone-sensitive and castration-resistant prostate cancer. The goal is to early identify in which biomarker signature a therapy class is superior to SOC, by comparing biomarker signature-therapy class combinations with respect to superiority in PFS to a common control group (SOC) (**Primary analysis**)	Progression-free survival, where progression is defined according to disease stage at trial entry:<br>• For mHSPC: Time to development of castration-resistance (European Association of Urology [EAU] guidelines)<br>• For mCRPC: Time to no longer clinical benefiting (NLCB) (Prostate Cancer Working Group [PCWG3] guidelines)

Secondary objectives and endpoints

**Objective**	**Endpoint**
Evaluating whether treatment class selection based on biomarker signatures can, compared to standard of care, improve the PFS distribution of the experimental arm altogether versus the control group (**key secondary analysis 1**)	Progression-free survival, where progression is defined according to disease stage at trial entry (see endpoint of primary analysis)

To determine whether a certain treatment class is superior for a certain biomarker signature compared to other treatment classes, by comparing experimental arms against each other (efficacy analysis) within any biomarker signature across experimental arms (**key secondary analysis 2**)	Progression-free survival, where progression is defined according to disease stage at trial entry (see endpoint of primary analysis)
Evaluating whether treatment class selection based on biomarker signatures can, compared to standard of care, improve treatment class response rate (RR) after 2-4 months of treatment	Treatment class response rate (RR)<br>● RECIST v1.1 objective response<br>● PSA response<br>● Composite overall response
Evaluating whether treatment class selection based on biomarker signatures can, compared to standard of care, improve time to biochemical progression	PSA-PFS
Evaluating whether treatment class selection based on biomarker signatures can, compared to standard of care, improve time to radiological progression	rPFS
Evaluating whether treatment class selection based on biomarker signatures can, compared to standard of care, improve the time from the initial study randomisation to the 2nd progression or death from any cause	PFS2
Evaluating whether treatment class selection based on biomarker signatures can, compared to standard of care, improve the time from the initial study randomisation to death from any cause	Overall Survival (OS)
Evaluating whether treatment class selection based on biomarker signatures can, compared to standard of care, improve quality of life	Quality of life assessed by<br>● EORTC-QLQ-C30<br>● EQ-5D-5L<br>● BPI-SF
Evaluating whether treatment class selection based on biomarker signatures can, compared to standard of care, improve health economy	Cost effectiveness will be assessed by using the EQ-5D-5L instrument to estimate health utilities. Treatment costs will be based on drug costs and reimbursement data.
Evaluating whether treatment class selection based on biomarker signatures, compared to standard of care, does not increase toxicity (i.e drug safety)	Frequency and severity of adverse events (AE) using Common Terminology Criteria for Adverse Events (CTCAE v5.0)
To identify additional predictive and prognostic biomarkers.	
Identify superior treatment sequencing regimens (i.e. is treatment A followed by treatment B superior to treatment B followed by treatment A given a biomarker signature)	

Exploratory objectives

**Objective**	**Endpoint**

Exploratory studies will be undertaken on biomaterial and information collected	Detailed information on exploratory objectives and endpoints are described in detail in the Trial Conduct Supplement *'AUXILIARY RESEARCH OBJECTIVES'*.

Trial design

ProBio is an outcome-adaptive, multi-arm, open-label, multiple assignment randomised biomarker driven platform trial in patients with metastatic hormone-sensitive and castration-resistant prostate cancer.

Patients will be randomised to control or experimental treatment class arms. Patients in the control arm will receive standard of care following national guidelines and will remain within the control arm throughout the course of the trial. Patients in the experimental arm will be randomised to a treatment class (consisting of one or multiple drugs) based on a biomarker signature. The biomarker signatures are defined as tumour properties or mutations in certain genes/pathways identified in the scientific literature as important in prostate cancer treatment response. The biomarker signatures are identified using a gene panel specifically designed for advanced prostate cancer.

Alterations in the following genes/pathways or combinations thereof constitute the initial biomarker signatures, and are described in detail in Trial conduct supplement *'BIOMARKER SIGNATURES'*:

- Androgen receptor
- DNA-repair deficiency
- TP53
- TMPRSS2-ERG gene fusion
- Other biomarker signatures upon drug availability and protocol amendment, such as:
  - TMB-H/MSI+/CDK12-
  - PI3K pathway alterations

New biomarker signatures can be identified by retrospective analysis and may be implemented in the trial design upon amendment of the protocol.

Patients in the experimental arm can be randomised to the following treatment classes, depending on national guidelines, availability and reimbursement criteria:

For mHSPC

- AR signalling inhibitors (ARSi, see *'SUB-PROTOCOL: STANDARD-OF-CARE IMP'*)
  - Abiraterone acetate plus prednisone
  - Apalutamide
  - Other ARSi upon approval from authorities and protocol amendment
- Taxane-based chemotherapy (see *'SUB-PROTOCOL: STANDARD-OF-CARE IMP'*)
  - Docetaxel
- Other investigational agent(s) sponsored by pharmaceutical company
  - Niraparib plus abiraterone acetate plus prednisone (see *'SUB-PROTOCOL: NIRAPARIB PLUS ABIRATERONE ACETATE PLUS PREDNISONE'*)
- Other agents or drug classes upon sub protocol amendment, e.g.
  - Checkpoint inhibitor
  - PI3K pathway inhibitor
  - PSMA-targeted therapy

For mCRPC

- AR signalling inhibitors (ARSi, see *'SUB-PROTOCOL: STANDARD-OF-CARE IMP'*)
  - Enzalutamide
  - Abiraterone acetate plus prednisone
- Taxane-based chemotherapy (see *'SUB-PROTOCOL: STANDARD-OF-CARE IMP'*)
  - Docetaxel
  - Cabazitaxel
- Platinum-based chemotherapy
  - Carboplatin (see *'SUB-PROTOCOL: CARBOPLATIN'*)
- Other investigational agent(s) sponsored by pharmaceutical company:

    - Niraparib plus abiraterone acetate plus prednisone (see *'SUB-PROTOCOL: NIRAPARIB PLUS ABIRATERONE ACETATE PLUS PREDNISONE'*)
- Other agents or drug classes upon sub protocol amendment, e.g.
    - Checkpoint inhibitor
    - PI3K pathway inhibitor
    - PSMA-targeted therapy

ProBio will use outcome-adaptive randomisation, i.e. the randomisation probabilities will be adapted based on the observed PFS within biomarker signatures. Treatment classes will initially be assigned to patients based on all biomarker signatures for which the treatment classes may be effective. The trial will be analyzed within a Bayesian framework, which allows for calculations of the probability for each treatment class that it is superior to standard of care within a given signature. Each experimental arm will be evaluated for efficacy relative to the control arm with the same biomarker signatures. The randomisation probabilities within the experimental arm are defined in proportion to the probability that each treatment class is superior to standard of care within a given biomarker signature, and will therefore be adapted as data accumulates in the trial and knowledge accumulates for what biomarker signatures and specific treatment classe that are more likely to be effective. Participants and treating physicians will be blinded to the biomarker signature profile of each patient. The biomarker signatures will thus not influence treatment choice among controls (reflecting today's standard of care).

Further, ProBio will use the sequential multiple assignment trial (SMART) concept:

- For patients entering the trial in the mHSPC setting, those patients will remain in the trial once mCRPC is reached, upon which the patient will be re-randomised for the 1st line systemic therapy for mCRPC. Alternatively, and upon request and preference from the patient and treating physician, progressive patients with mHSPC and who have thus reached mCRPC may exit the trial or enter other RCTs, e.g. the MAGNITUDE trial (NCT03748641).
- For patients entering the mCRPC stage of the trial, or upon progression from mHSPC to mCRPC, blood will be drawn for ctDNA profiling. Patients will be randomised based on this biomarker profile. I.e., men progressing from mHSPC to mCRPC within ProBio will essentially re-enter the trial in the mCRPC setting with an updated biomarker profile.
- Patients who are enrolled in the trial and are progressing on 1st line mCRPC therapy will be re-randomised for a 2nd line of mCRPC treatment based on a new biomarker profile. Maximally, a patient can be randomised three times and receive three treatments in the trial after inclusion, i.e. mHSPC → 1st line mCRPC → 2nd line mCRPC.

Trial results will be evaluated regularly by an independent data and safety monitoring board (DSMB). The DSMB will evaluate treatment class-signature combinations with respect to:

Graduation for superiority: A treatment class-biomarker signature combination will be graduated from the trial if it has a probability of superiority compared to standard of care exceeding a pre-specified threshold (85%).

Termination for futility: Treatment class-biomarker signature combinations will be dropped from the trial for futility when success probabilities drop sufficiently low (less than 30% using a minimum of 20 patients assigned to the specific treatment class-biomarker signature combination). Alternatively, if the maximum sample size (500 for mHSPC and 150 for the mCRPC setting) is assigned to a treatment class-biomarker signature combination without graduation for superiority, assignments to that combination will end.

Validation trial: Graduated biomarker signature – treatment class combinations will be validated in a frequentist validation trial within the framework of ProBio. Following graduation for superiority the DSMB will evaluate the available data and in collaboration with the trial investigators, make decisions about: 1) if the biomarker signature needs to be adjusted in the validation arm 2) the validation trial design taking the effect size of the graduated biomarker-signature treatment class combination into account.

ProBio is a platform study covering both the mHSPC and mCRPC stage of the disease. This means that new treatments and/or biomarker signatures can be added to the experimental arms in the future. This will be done after authority approval and protocol amendments.

Number of patients planned

ProBio is a platform trial, and new arms may be added to the platform throughout the course of the trial. Therefore, there is no maximum number of patients planned. Each signature-treatment class combination may receive a maximum of 300 and 150 patients in the mHSPC and the mCRPC setting, respectively.

Diagnosis and main eligibility criteria

Inclusion criteria

- Male patients, aged above 18 years, with histologically confirmed prostate adenocarcinoma, initiating systemic therapy for metastatic disease, encompassing
  - Newly diagnosed (i.e. de novo) metastatic hormone-sensitive prostate cancer (mHSPC)

    or
  - First-line mCRPC, i.e. first evidence of progressive metastatic prostate cancer under castrate levels (<50 ng/dL) of serum testosterone, as defined by the EAU guidelines, encompassing:
    - Biochemical progression: Three consecutive rises in PSA 1 wk apart, resulting in two 50% increases over the nadir, and PSA >2 ng/ml

      and/or
    - Radiologic progression: The appearance of new lesions: either two or more new bone lesions on bone scan or a soft tissue lesion using the Response Evaluation Criteria in Solid Tumours.
- Distant metastatic disease documented by conventional imaging, i.e. positive bone scan or metastatic lesions on CT or MRI. Radiology taken within 6 weeks of inclusion may be used, if older a new scan needs to be taken. With the advent of novel imaging modalities using radionuclides, e.g. ^{68}Ga-PSMA-11 PET/CT, the ProBio trial will allow for future incorporation of these imaging modalities upon availability of validated guidelines, progression criteria and protocol amendment.
- Adequate health, hematologic, hepatic, and renal function, as assessed by the investigator, to receive all available treatments in the trial in each disease state (mHSPC and mCRPC) (i.e. haemoglobin ≥ 100 g/L (blood transfusion not less than 21 days prior to screening), absolute neutrophil count ≥ 1.5 x 10^9/L, platelets ≥100 x 10^9/L and Total bilirubin < 1.5 ULN (patients with Gilberts Syndrome bilirubin < 40 µg/L) and AST and ALT ≤ 1.5 ULN (or ≤ 3 ULN in the presence of liver metastases) and serum creatinine not greater than 1 ULN (if serum creatinine is between 1 and 1.5 ULN, patients may be eligible provided that the calculated GFR is at least 50 ml/min measured directly by 24-hour urine sampling OR using Cockcroft-Gault method)
- Albumin greater than or equal to 28 g/L
- ECOG/WHO performance status 0-2
- Able to understand the patient information and sign written informed consent.
- Agrees to use an effective contraceptive method during and up to 6 months after study drug treatment, and should not donate sperm during this period.

Exclusion criteria

- Other malignancies within 5 years except non-melanoma skin cancer
- Within 6 months of randomisation: myocardial infarction, unstable angina, angioplasty, bypass surgery, stroke, TIA, or congestive heart failure NYHA class III or IV
- Uncontrolled hypertension. Subjects with a history of hypertension are allowed provided blood pressure is controlled by anti-hypertensive treatment
- Upon entering the mHSPC phase of the trial, prior systemic therapy (including ADT) is not allowed. Patients with mCRPC may not enter the trial when they have already received prior systemic therapy (with the exception of standard ADT) for mCRPC.
- Any severe acute or chronic medical condition that places the patient at increased risk of serious toxicity or interferes with the interpretation of study results
- Unable to comply with study procedures
- Current participation in another clinical trial that will be in conflict with the present study, e.g. administration of an investigational therapeutic or invasive surgical procedure within 28 days prior to study enrolment. Imaging-based interventional trials are allowed as long as the conventional imaging intervals within ProBio are preserved.
- Patients who are unlikely to comply with the protocol (e.g. uncooperative attitude, inability to return for subsequent visits) and/or otherwise considered by the Investigator to be unlikely to complete the study
- Any condition or situation which, in the opinion of the investigator, would put the subject at risk, may confound study results, or interfere with the subject's participation in this study
- Any medical condition that would make use of the study treatments contraindicated, according to the SmPC, e.g. significant heart or liver disease. The investigator should check the SmPC and/or IB for the assigned study treatments.
- The determination of a biomarker signature is necessary to randomise patients during ProBio. Patients will therefore be excluded in case of:
  - For patients with mHSPC: failure to detect ctDNA or somatic alterations from the primary tumour biopsies
  - For patients with mCRPC: undetectable levels of ctDNA.

Investigational Medicinal Products (IMPs)

- Androgen receptor signalling inhibitors (ARSi)
  - Enzalutamide
  - Abiraterone acetate
  - Apalutamide
  - Other ARSi upon authority approval and protocol amendment
- Taxane-based chemotherapy
  - Cabazitaxel
  - Docetaxel
- Platinum-based chemotherapy
  - Carboplatin
- Poly ADP Ribose Polymerase inhibitors
  - Niraparib
- Other agents upon protocol amendment

IMP will be used in brand products, doses and dosages according to national labelling and the current SmPC (Summary of Product Characteristics) and/or IB (investigator brochure).

Duration of treatment

The duration of treatment will last until the patient

- For mHSPC
  - develops castration-resistant disease, as defined by EAU guidelines
- For mCRPC
  - no longer has a clinical benefit from the treatment, based on evaluation of the biochemical, clinical and/or radiological progression of the disease or intolerable toxicity, as defined by PCWG3 criteria.

Duration of patients' involvement in the trial

Treatment will continue until disease progression, withdrawal of consent, or the occurrence of unacceptable toxicity. Patients can be reassigned to another treatment within the study based on the SMART (Sequential, Multiple Assignment, Randomised Trial) concept two additional times for a maximum of three randomisations.

Safety assessments

Safety evaluations include adverse events, vital signs measurements, physical examinations, medical imaging, and clinical laboratory tests.

Safety overview will be done periodically after 200 patients have been included and conducted every 3 months. An independent data and safety monitoring board will be responsible for evaluating and follow-up of the safety.

Statistical methods

The statistical analyses are described in detail in the Statistical Analysis Plan (SAP) including a Data Display Plan, which will be finalized and approved before database lock of first dropped or graduated biomarker signature-treatment class combination (for graduation or futility).

All data will be presented using descriptive statistics. Results will be presented in total, by treatment class, and across biomarker signatures. Continuous variables will be summarized using measures of central tendency and variability. Categorical variables will be summarized using absolute and relative frequencies.

We will use Bayesian parametric models to contrast the posterior mean PFS distributions across treatment class arms within biomarker signatures. We will report the differences in the mean PFS survival as measures of treatment effect.

The primary analysis will be on an intention to treat (ITT) basis and drop-outs will be censored at their last observed time point. We will also perform per protocol (PP) secondary analyses, where drop-outs and patients not treated according to the protocol are excluded from the analysis.

Patients will be re-assigned to treatment classes multiple times, permitting the sponsor to learn about the effectiveness of biomarker signature-treatment class combinations several times from each patient, and analyses are therefore

conditioned on randomisation round. The Sequential Multiple Assignment Randomisation Trials (SMART) design concept will also be used to generate hypotheses about the effectiveness of different dynamic treatment regimes, e.g. advantages with respect to progression free and overall survival of different sequences of treatments. Additional analyses will include comparisons of progression free survival in all patients randomised to the biomarker driven treatment class arms to patients in the standard of care arm, as well as comparisons between therapies in the two randomised groups (irrespective of biomarker signature).

MASTER PROTOCOL

Trial title:

ProBio:
An outcome-adaptive and randomised multi-arm biomarker driven study in patients with metastatic prostate cancer

Coordinating Investigator and Sponsor's representative

Henrik Grönberg
Karolinska Institutet

PROTOCOL SIGNATURE PAGE

Trial title:

ProBio: An outcome-adaptive and randomised multi-arm biomarker driven study in patients with metastatic prostate cancer

Sponsor

Professor Henrik Grönberg	Signature	Date

Coordinating Investigator

Professor Henrik Grönberg	Signature	Date

INVESTIGATOR SIGNATURE PAGE

Site Principal Investigator

Trial title:
ProBio: An outcome-adaptive and randomised multi-arm biomarker driven study in patients with metastatic prostate cancer

I agree to the terms of this trial protocol. I will conduct the study in accordance with the procedures specified in the protocol, the ethical principles in the latest version of the Declaration of Helsinki, ICH Good Clinical Practice and applicable regulatory requirements.

Investigator Name		Signature		Date

TABLE OF CONTENTS

PROTOCOL SIGNATURE PAGE 2

INVESTIGATOR SIGNATURE PAGE 3

TABLE OF CONTENTS 4

PREFACE 7

1. BACKGROUND 8
1.1 Metastatic hormone-sensitive prostate cancer 8
1.2 Metastatic castration-resistant prostate cancer 8
1.3 Biomarkers and the advantage of liquid biopsies 9

2. TRIAL OBJECTIVES AND ENDPOINTS 11
2.1 Hypothesis 11
2.2 Primary clinical efficacy objectives and endpoints 12
2.2.1 Motivation for choice of primary endpoint 12
2.2.2 Measurement of progression-free survival (PFS) 13
2.2.2.1 Definition of progression-free survival for patients entering the mHSPC platform of ProBio 13
2.2.2.2 Definition of progression-free survival for patients entering the mCRPC platform of ProBio 14
2.3 Secondary clinical efficacy objectives and endpoints 15
2.3.1 Measurement of treatment response rate (RR) 15
2.3.2 Quality of life objectives 16
2.3.3 Safety and adverse events (AE) objectives 16
2.3.4 Cost-effectiveness objectives 16
2.4 Exploratory objectives 16

3. INVESTIGATIONAL PLAN 17
3.1 Rationale for trial design 17
3.2 Overall trial design 18

4. PATIENT SELECTION 22
4.1 Selection of trial population 22
4.1.1 Inclusion criteria 22
4.1.2 Exclusion criteria 23
4.2 Patients treated with study drug(s) before (re-)randomisation 23
4.3 Removal of trial patients from treatment or assessments 24

5. TREATMENT OF PARTICIPANTS 25
5.1 Standard-of-care androgen deprivation therapy (ADT) as background systemic therapy 25
5.2 Identity of Investigational Medicinal Product (IMP) 25
5.3 Treatment compliance 26

5.4 Prior and concomitant therapy 26
5.5 Treatment procedures during and upon subject (re-)randomisation 27
5.6 Treatment procedures upon intolerance 28

6. PATIENT LEVEL TRIAL ASSESSMENTS 29
6.1 Demographics, baseline characteristics and medication use 29
6.2 Vital signs and physical examination 29
6.3 Patient-reported outcomes 30
6.4 Patient sample collection for routine laboratory measurements and biobanking of liquid biopsies 30

7. STUDY PROCEDURES 31
7.1 Technical pilot study prior to study initiation 31
7.2 Sample handling and liquid/tissue biopsy profiling during ProBio 32
7.3 Procedures during patient study visits 33
7.3.1 Patients entering the mHSPC platform from the ProBio trial 33
7.3.1.1 Visit 1 (Screening - Phase 1) 33
7.3.1.2 Visit 2 (Screening - Phase 2) 34
7.3.1.3 Randomisation 35
7.3.1.4 Visit 3 (treatment start, time = 0 months ± 1 week) 35
7.3.1.5 Visit 4 (time = month 1 ± 1 week) 35
7.3.1.6 Visit [5, 6, 7, 8, etc.] (time = month 3, 6, 9, 12, etc. ± 3 weeks) 36
7.3.2 Schedule of activities in mHSPC 37
7.3.3 Patients entering the mCRPC platform from the ProBio trial 38
7.3.3.1 Visit 1 (Screening) 38
7.3.3.2 Randomisation 39
7.3.3.3 Visit 2 (treatment start, time = month 0 ± 1 week) 39
7.3.3.4 Visit 3 (time = month 1 ± 1 week) 39
7.3.3.5 Visit [4, 5, 6, 7, etc.] (time = month 2, 4, 6, 9, etc. ± 3 weeks) 40
7.3.4 Schedule of activities in mCRPC 41

8. PHARMACOVIGILANCE 42
8.1 Safety assessments 42
8.2 Adverse events 42
8.3 Definitions 42
8.4 Eliciting and recording of AEs 44
8.5 Pre-existing condition 44
8.6 Reporting of SAEs 44
8.7 Follow-up of unresolved AEs 45
8.8 Coding of AEs 45
8.9 Country-specific safety reporting procedures and contact details 45
8.9.1 AE 46
8.9.2 SAE 46
8.9.3 SUSAR 46

9. STATISTICAL CONSIDERATIONS 48
9.1 Biomarker subgroup combinations versus biomarker signatures 49
9.2 Randomisation 50
9.3 Interim analyses and data monitoring 51
9.4 Analytical populations 51
9.5 Statistical analysis plan summary 51
9.6 Determination of sample size 52
9.6.1 Simulations and sample size calculation for the mHSPC platform of the ProBio trial 53
9.6.2 Simulations and sample size calculation for the mCRPC platform of the ProBio trial 54
9.6.3 Simulations web interface 55
9.7 Confirmatory trial 55

10. ETHICS 56
10.1 Ethical and Regulatory review 56
10.2 Ethical conduct of the trial 56
10.3 Patient information and consent 56
10.4 Patient data protection 56
10.5 Ethical considerations in terms of detected genomic alterations 57
10.5.1 Blinding of biomarker signatures 57
10.5.2 MSI 57
10.5.3 Risk vs benefit assessment in the context of Homologous Recombination Repair Deficiency (HRD) 57

11. DATA HANDLING, RECORD KEEPING AND MONITORING 58
11.1 Data Management 58
11.2 Quality Control and Data Quality Assurance 58
11.2.1 Case Report Forms (eCRFs) 59
11.2.2 Monitoring 59
11.2.3 Training of staff 59
11.2.4 Audit and inspections 59
11.2.5 Changes in the approved Trial Protocol 59

12. GENERAL TRIAL MANAGEMENT 60
12.1 Trial time table 60
12.2 Insurance/indemnity 60
12.3 Trial agreements 60
12.4 Criteria for termination of the trial 61
12.5 Record retention 61
12.6 Disclosure and confidentiality 61
12.7 Emergency procedures 61

PREFACE

ProBio is an outcome-adaptive and randomised multi-arm biomarker driven study in patients with metastatic prostate cancer. Given the complexity of the trial design and conduct, the clinical protocol has been built up in a structured way, following the recommendations from the Clinical Trials Facilitation and Coordination Group (CTFG), a working group of the Heads of Medicines Agencies on clinical trials (*https://www.hma.eu/ctfg.html*). This entails that a Master protocol was written encompassing a general description of e.g. study background, endpoints and objectives, investigational plan, patient selection, study procedures, etc. Additionally, given the flexibility of incorporating new treatment arms and/or biomarkers into the trial, several treatment-specific sub protocols have been developed next to trial conduct supplements and appendices (*Figure 1 - ProBio structured protocol design*).

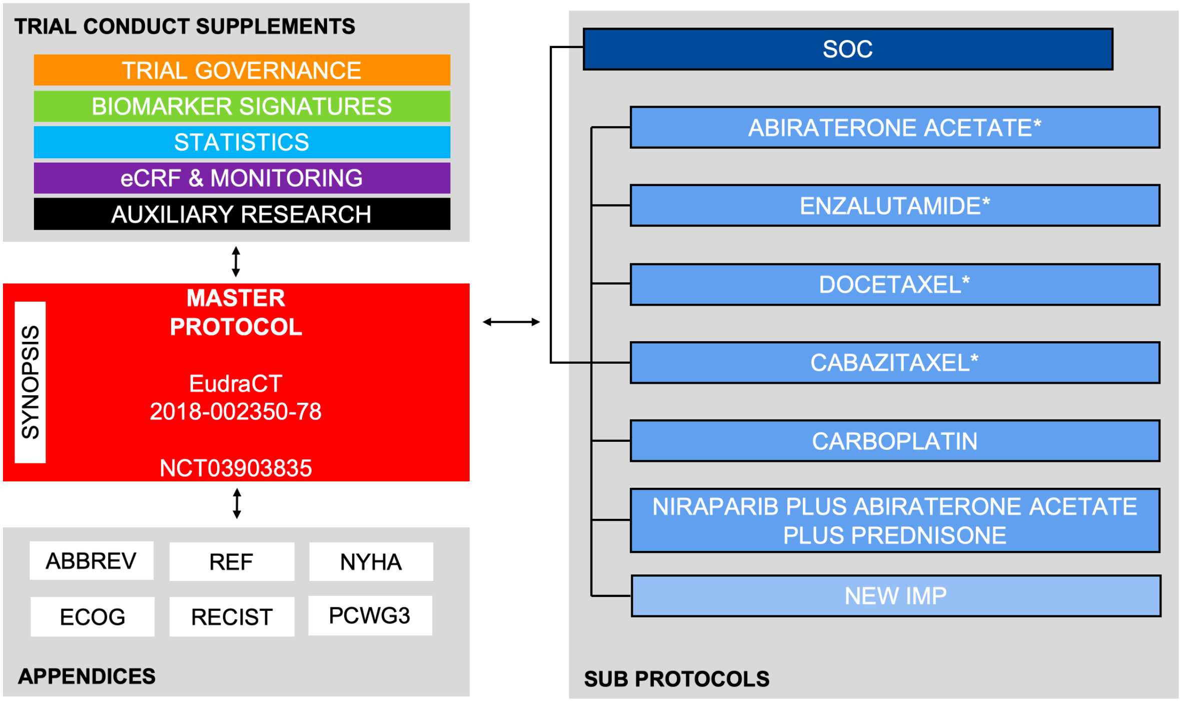

Figure 1 - ProBio structured protocol design. *ProBio has a complex trial design with multiple treatment arms being compared to a shared standard-of-care (SOC) control arm. The structured protocol design is characterised by a master protocol with several sub-protocols, trial conduct supplements and appendices. The trial and structured protocol design allow for extensive adaptations where e.g. arms with new IMPs are being opened and closed or new biomarker signatures are being incorporated during the conduct of the trial via substantial amendments of new/existing sub protocols and trial conduct supplements. eCRF: electronic case report form; ABBREV: abbreviations; ECOG: Eastern Cooperative Oncology Group; REF: references; RECIST: Response Evaluation Criteria in Solid Tumors; NYHA: New York Heart Association; PCWG3: Prostate Cancer Working Group 3; SOC: standard-of-care; IMP: Investigational Medicinal Product. * denotes that abiraterone acetate, enzalutamide, docetaxel and cabazitaxel are approved SOC drugs for prostate cancer and therefore drug-specific information is identical to and described in the SOC sub protocol.*

1. BACKGROUND

Prostate cancer is the most common cancer diagnosed in European men. Every year more than 450,000 men are diagnosed and about 107,000 die of prostate cancer (Ferlay et al. 2018). In men with metastatic prostate cancer (mPC), hormonal treatment (surgical or medical castration) has been standard care for the last 75 years. In recent years, combinations of castration with chemotherapy or androgen receptor signaling inhibitors (ARSi) has demonstrated significant increases in overall survival compared to men receiving only castration, which has changed the standard of care worldwide (Sweeney et al. 2015; James et al. 2017; Fizazi et al. 2017). However, eventually all men will progress and develop metastatic castration-resistant prostate cancer (mCRPC). It is estimated that around 20-30% of all prostate cancer diagnoses will develop mCRPC at some point in their disease trajectory.

1.1 Metastatic hormone-sensitive prostate cancer

For particular subpopulations of men with metastatic hormone-sensitive prostate cancer, the combination of ADT with either AR signaling inhibitors, e.g. abiraterone acetate (Fizazi et al. 2017; James et al. 2017), or docetaxel chemotherapy (James et al. 2016; Sweeney et al. 2015) offers significant survival advantages (*Table 1 - Endpoint results from RCTs in metastatic hormone-sensitive prostate cancer*) and is now incorporated into the ASCO guidelines (Morris et al. 2018). Although the optimal patient selection has not yet been established, the guidelines recommend the association of abiraterone acetate or docetaxel for men with hormone-sensitive metastatic prostate cancer, with local radiotherapy of the prostate in case of *de novo* low-volume disease (C. C. Parker et al. 2018). Comparative data of the combination of ADT with abiraterone acetate versus docetaxel is limited, but a subset analysis of STAMPEDE (Sydes et al. 2018) and two meta-analyses (Wallis et al. 2018) demonstrated similar survival benefits, which was also observed in a network meta-analysis of <70 year-old patients with ECOG <2, Gleason score <8, or visceral metastases (Feyerabend et al. 2018). Besides combining ADT with abiraterone acetate or docetaxel, the combination with antiandrogens (i.e. combined androgen blockade) enzalutamide and apalutamide have demonstrated their antitumoral effect in men with mHSPC during two randomised trials (ENZAMET [Australian and New Zealand Urogenital and Prostate Cancer Trials Group (ANZUP) 1304] and TITAN) (I. D. Davis et al. 2019; Chi et al. 2019). Currently, no treatment predictive biomarker exists that could aid therapy or drug class (e.g. ADT + ARSi or ADT + taxane) selection in men with mHSPC.

Table 1 - Endpoint results from RCTs in metastatic hormone-sensitive prostate cancer. *rPFS: radiographic progression-free survival. OS: overall survival, NR: not reached*

		PFS				OS			
**Trial**	**Therapy**	**median (mo)**	**HR**	**95%CI**	**type**	**median (mo)**	**HR**	**95%CI**	**Ref**
**GETUG-15**	Docetaxel	22.9	0.69	0.55 - 0.87	rPFS	62.1	0.88	0.68 - 1.14	Gravis et al, *Eur Urol*, 2016
**CHAARTED**	Docetaxel	33	0.62	0.51 - 0.75	Clinical	57.6	0.72	0.59 - 0.89	Kyriakopoulos et al, *J Clin Oncol*, 2018
**STAMPEDE (C)**	Docetaxel	37	0.69	0.59 - 0.81	PFS	59.1	0.81	0.69 - 0.95	Clarke et al, *Ann of Onc*, 2019
**STAMPEDE (G)**	Abiraterone	NR, 3y: 75% *	0.45	0.37 - 0.54	FFS	NR, 3y: 83% *	0.61	0.49 - 0.79	Hoyle et al, *Eur Urol*, 2019; *James et al, *N Engl J Med*, 2017
**LATITUDE**	Abiraterone	33.3	0.31	0.27 - 0.36	PSA	53.3	0.66	0.56 - 0.78	Fizazi et al, *Lancet Onc*, 2019
**ARCHES**	Enzalutamide	NR, 2y: 73%	0.39	0.30 - 0.50	rPFS	NR	0.81	0.53 - 1.25	Armstrong et al, *J Clin Oncol*, 2019
**ENZAMET**	Enzalutamide	NR, 3y: 68%	0.40	0.33 - 0.49	Clinical	NR, 3y: 80%	0.67	0.52 - 0.86	Davis et al, *N Engl J Med*, 2019
**TITAN**	Apalutamide	NR, 2y: 68.2%	0.48	0.39 - 0.60	rPFS	NR, 2y: 82.4%	0.67	0.51 - 0.89	Chi et al, *N Engl J Med*, 2019

1.2 Metastatic castration-resistant prostate cancer

Although ADT is initially sufficient in almost all patients with advanced disease to control the disease, the cancer cells will achieve the ability to progress under castrate-levels of circulating androgens, which is referred to as metastatic castration-resistant prostate cancer (mCRPC). Nonetheless, mCRPC

is usually amenable to treatment with novel ARSi. Clinical management of mCRPC typically encompasses the sequential use of different systemic agents, including novel hormonal therapies, chemotherapy, and other approaches, e.g. such as radionuclide- and immuno-therapy. All of which aiming towards a prolongation of survival while maintaining quality of life. However data on optimal sequencing of these options is limited (Khalaf et al. 2019; de Wit et al. 2019).

Besides docetaxel (Taxotere), four new drugs have been approved for the treatment of mCRPC and are routinely used in European health care centres. The drugs are enzalutamide (Xtandi), abiraterone acetate (Zytiga), cabazitaxel (Jevtana) and Ra-223 (Xofigo), and all lead to a moderate increase in overall survival. Although these drugs are definitely beneficial for many patients they carry three serious disadvantages (*Table 2 - Endpoint results from RCTs in metastatic castration-resistant prostate cancer*). Firstly, these drugs are all very expensive. With prices ranging between EUR 3,000-5,000 per month they constitute a major cost burden for cancer care. Secondly, the response rates to these drugs are relatively low, which leads to suboptimal treatment and unnecessary side effects (Johann Sebastian de Bono et al. 2010; Berruti, Pia, and Terzolo 2011; Scher et al. 2012; Ryan et al. 2013; C. Parker et al. 2013; Beer et al. 2014). Thirdly, there are no predictive treatment markers available in clinical care today, which leads to ineffective trial-and-error in treatment decisions. This raises the question which systemic therapy or therapy class (e.g. ARSi or taxane) needs to be initiated in which patients with mCRPC.

Table 2 - Endpoint results from RCTs in metastatic castration-resistant prostate cancer. *rPFS: radiographic progression-free survival. OS: overall survival*

		PFS				OS			
**Trial**	**Therapy**	**median (mo)**	**HR**	**95%CI**	**type**	**median (mo)**	**HR**	**95%CI**	**Ref**
**TROPIC**	Cabazitaxel	2.8	0.74	0.64 - 0.86	PFS	15.1	0.70	0.59 - 0.83	De Bono et al, *Lancet Onc*, 2010
**ALSYMPCA**	Radium-223	3.6	0.64	0.54 - 0.77	PSA	14.9	0.70	0.58 - 0.83	Parker et al, *N Engl J Med*, 2013
**COU-AA-301**	Abiraterone post docetaxel	5.6	0.66	0.58 - 0.76	rPFS	15.8	0.74	0.64 - 0.86	Fizazi et al, *Lancet Onc*, 2012
**COU-AA-302**	Abiraterone pre docetaxel	16.5	0.53	0.45 - 0.62	rPFS	34.7	0.81	0.70 - 0.93	Ryan et al, *Lancet Onc*, 2015
**AFFIRM**	Enzalutamid post docetaxel	8.3	0.40	0.35 - 0.47	rPFS	18.4	0.63	0.53 - 0.75	Cabot et al, *N Engl J Med*, 2012
**PREVAIL**	Enzalutamide pre docetaxel	20.0	0.32	0.28 - 0.36	rPFS	35.3	0.77	0.67 - 0.88	Beer et al, *Eur Urol*, 2017

1.3 Biomarkers and the advantage of liquid biopsies

In advanced prostate cancer, several predictive and prognostic biomarkers have been suggested for abiraterone acetate and enzalutamide. The AR-V7 splice variant demonstrated initial promising results as a negative response biomarker (Antonarakis et al. 2014). However, the initial finding of clear-cut association could not be validated in follow-up studies (Antonarakis et al. 2017; Armstrong, Halabi, et al. 2019) and is currently a subject of intense debate (Steinestel et al. 2017; De Laere et al. 2019; Plymate, Sharp, and de Bono 2018). Amplification and mutations in the androgen receptor (AR) have also been associated with poor treatment response (Romanel et al. 2015; Annala et al. 2018). However, clinical applicability remains uncertain as individual mutations in AR have different mechanisms of action (Lallous et al. 2016) and amplifications were not significantly associated to PFS in a recent report (Annala et al. 2018). Intra-AR structural rearrangements, detectable by liquid biopsies (De Laere et al. 2017), are associated with resistance to endocrine therapy by the generation of non-canonical AR transcripts facilitating ligand independent growth (Henzler et al. 2016)). Our group recapitulated this association of intra-AR structural variation with PFS in context of ARSi (De Laere et al. 2018). Apart from the AR signaling pathway, PTEN and TP53 have also been associated with poor response (Annala et al. 2018; Hussain, Daignault-Newton, et al. 2018; Maughan et al. 2018; Ferraldeschi et al. 2015). The evidence for PTEN remains obscure as the association was done without interrogation of TP53 (Ferraldeschi et al. 2015). Multiple other studies have assessed both genes simultaneously and found only TP53 to be significantly associated with abiraterone acetate and enzalutamide outcome in multivariate analysis (Annala et al. 2018; Hussain, Daignault-Newton, et al. 2018; Maughan et al. 2018).

Comprehensive liquid biopsy profiling recently revealed that TP53 outperformed other AR biomarkers in multivariate analysis (De Laere et al. 2018) which need to be comprehensively profiled and simultaneously taken into account in order to identify patients with poor prognosis starting ARSi therapy. In addition, WNT and RB1 alterations have been suggested to infer *a priori* resistance to ARSi, however, current literature consist of small sets of somatically altered patients without incorporating prognostic routine biomarkers in multivariate analyses (Abida et al. 2019; Chen et al. 2019). Genes in the HRR pathways were also suggested to be associated with poor response (Annala et al. 2018) but independent efforts came to opposite conclusions (Hussain, Daignault-Newton, et al. 2018; Antonarakis et al. 2018) or did not find any difference in PFS (Mateo et al. 2018).

Poly (ADP-ribose) polymerase (PARP) inhibitors prevent the PARP enzymes to repair single-strand nicks in the DNA. During replication, double strand breaks accumulate which cannot be efficiently repaired in tumors with homologous recombination deficiency (Farmer et al. 2005; Bryant et al. 2005). In mPC, mutations in Homologous Recombination Repair (HRR)-associated genes (e.g. BRCA1, BRCA2, ATM), termed HRR deficiency (HRD), were predictive for response and survival to the PARP-inhibitor olaparib in a pivotal study of 50 men with mCRPC (TOPARP-A) (Mateo et al. 2015). However, contradicting results were reported in a retrospective study of 390 mCRPC patients investigating carriers of pathogenic germline variants in HRR genes (Mateo et al. 2018). Heterogeneity exist in the mutational signature patterns associated with inactivation of different HRR genes, which may potentially explain the heterogeneity in response to PARP inhibition (Polak et al. 2017). Recently, TOPARP-B and PROfound validated the antitumor activity of the PARP inhibitor Olaparib in men with HRD, however, the treatment effect was primarily observed in BRCA complex-associated genes (BRCA1/2 and PALB2) and not in other HRD-associated genes (Mateo et al. 2019; J. de Bono et al. 2020).

During 2017 FDA approved the immunomodulator Pembrolizumab in patients with microsatellite instable (MSI) or mismatch repair deficient tumors in all cancers irrespectively of primary site (Food, Administration, and Others 2017). Response rates >50% in solid tumors of various origins support the decision (Le et al. 2017). Three to four percent of prostate cancers have the MSI phenotype. Although immunotherapy did not demonstrate increased survival in unselected mCRPC (Kwon et al. 2014; Beer, Kwon, et al. 2017), subsets of patients hypermutated, MSI+ or CDK12 deficient have reported partial- or complete responses (Abida et al. 2018; Le et al. 2017; Cabel et al. 2017; Zehir et al. 2017; Wu et al. 2018; Lee et al. 2018).

No consistent treatment predictive markers exist for Cabazitaxel. Recent data suggest that patients with alterations in HRR genes treated with Ra-223 have an increased progression-free survival (Isaacsson Velho et al. 2018). The GETUG 12 and 15 trials investigated the effect of adding docetaxel to hormonal therapy in high-risk localized and treatment naïve metastatic cancer, respectively. Retrospective analysis suggests the improvement in PFS is confined to the cancers harboring the TMPRSS2-ERG gene fusion (Rajpar et al. 2017), however contradictory data exist and the prognostic and predictive properties of the TMPRSS2-ERG gene fusion require further evaluation (Rescigno et al. 2018; Galletti et al. 2014). In addition, treating PTEN-deficient cancers with the AKT1-inhibitor ipatasertib demonstrated promising results (J. S. de Bono, De Giorgi, and Rodrigues 2019). Principally, multiple biomarkers have demonstrated promising initial results but the common denominator for all is the lack of testing in randomised clinical trials.

The lack of predictive biomarkers in metastatic prostate cancer is partly due to the difficulty of obtaining metastatic tissue for molecular analysis as the majority metastasize to the bone. A range of success rates, with or without direct image-guidance, has been reported (Spritzer et al. 2013; Lorente et al. 2016; Holmes et al. 2017; Sailer et al. 2017) and is correlated to tumor burden (Lorente et al. 2016). Liquid biopsies might offer an alternative to sampling of metastatic tissue, which for prostate cancer has demonstrated a high concordance in direct comparisons (Wyatt et al. 2017; Razavi et al.

2019). The vast majority of mutations detected in tissue were also present in ctDNA. In addition to the higher success-rate of ctDNA, the following advantages render ctDNA assessments highly attractive: 1) has faster turnover 2) cost less 2) has no side-effects 3) allows for longitudinal monitoring 4) provides information from multiple metastatic sites (Goodall et al. 2017; Quigley et al. 2017).

To enable biomarker driven clinical trials we have established a hybridisation capture gene panel, tailored for metastatic prostate cancer. Pilot data from 208 cases and 269 treatment baseline blood draws identify ctDNA in 85.1% of the study participants (Mayrhofer et al. 2018). Although detection was possible for the majority of men, the fraction of tumor DNA in plasma or tissue determines what types of somatic alterations that can be identified. Men with homozygous deletion of BRCA2 is associated with response to carboplatin or PARP inhibition (Cheng et al. 2016; Mateo et al. 2019). To call all classes of somatic alterations, including homozygous deletions, >0.2 ctDNA fraction is required which was possible for 57% of mHSPC, 33% of first line mCPRC and approximately 50% of second and third line mCRPC in our pilot data. Therefore, and for mHSPC patients not exposed to the evolutionary pressure of treatment causing inter-metastatic lesion heterogeneity (Gundem et al. 2015) tumor profiling of the diagnostic biopsies is a viable option if ctDNA fraction is <0.2. For mCRPC, sampling of metastasis tissue is not feasible in a clinically relevant time-frame in a multicenter trial as ProBio. A mCRPC metastatic tumor sequencing effort of 150 patients reported tumor suppressor biallelic inactivation in all cases where one high-impact variant was detected (Robinson et al. 2015). To test the hypothesis that one clonal high-impact variant may infer tumor suppressor deficiency when ctDNA fraction is too low to detect e.g. loss of heterozygosity, the non-repetitive regions were captured and sequenced for the entire gene body of TP53, PTEN and RB1. Seventy-one samples from 59 men had a ctDNA fraction ≥0.2, where all classes of somatic variation are detectable. Biallelic inactivation occurred in 47.5%, 20.3% and 44.1% of patients in PTEN, RB1 and TP53, respectively. Only one patient harboured a clonal variant in TP53 without the presence of a second hit. In concordance to previous studies on mCRPC tissue (Robinson et al. 2015), high impact mutations associated with HRD were detected in 18.0% of patients and microsatellite instability in 3.81% of eligible samples (≥10% ctDNA fraction). AR-alterations, amplifications, mutations and intra-AR structural rearrangements occurred in 45.8% of the samples and 50.3% of the patients, which is in line with reports investigating amplifications and mutations (Romanel et al. 2015; Wyatt et al. 2016).

In summary, the combination of new expensive drugs with moderate effects and the lack of predictive treatment markers will lead to an unsustainable situation (Mailankody and Prasad 2015). The need for systematic evaluation of treatment predictive markers in metastatic prostate cancer is evident and urgent (Barbieri et al. 2017). With the development of ctDNA and NGS technology we now have a powerful tool to assess molecular biomarkers in advanced prostate cancer.

2. TRIAL OBJECTIVES AND ENDPOINTS

2.1 Hypothesis

The proposed hypothesis is that therapy class (e.g. ARSi, PARPi, taxane- or platinum-based chemotherapy, etc.) decisions based on biomarker signatures identified by sequencing ctDNA and/or diagnostic biopsies (e.g. in case of low/undetectable circulating tumour burden in patients with *de novo* mHSPC) significantly will increase the progression free survival (PFS) in patients with metastatic hormone-sensitive (mHSPC) and castration-resistant (mCRPC) prostate cancer compared to current clinical standard of care (SOC). The goal is to identify in which biomarker signature a therapy class is superior to SOC. Those hypotheses will be tested in a large multicentre randomised controlled trial with an outcome-adaptive, multi-arm, biomarker-driven design in male patients, aged above 18 years,

with histologically confirmed prostate adenocarcinoma, initiating systemic therapy for metastatic disease, encompassing:

1. newly diagnosed, i.e. de novo, metastatic hormone-sensitive prostate cancer (mHSPC)
2. first-line metastatic castration-resistant prostate cancer (mCRPC), i.e. at first evidence of progressive disease under castrate levels (<50 ng/dl) of serum testosterone, as defined by the EAU guidelines.
3. re-randomised mCRPC patients starting 2nd line systemic therapy after randomisation to and progression on 1st line therapy for mCRPC.

Specifically, we aim to investigate if treatment decisions based on a biomarker signature identified by sequencing circulating or, in case of *de novo* mHSPC with low/undetectable ctDNA, diagnostic biopsy-derived tumour DNA:

- improves PFS (primary endpoint), where progression is defined according to disease stage at trial entry
- improves PSA-PFS
- improves radiographic PFS
- improves treatment response rate (RR) after 3-4 months of treatment
- improves PFS2
- improves overall survival
- improves quality of life
- improves health economy
- does not increase toxicity

compared to current standard of care (i.e. treatment without relating the treatment decision to the information about the cancer's biomarker), and thus demonstrating the clinical utility of the pre-specified biomarker signature as a predictive biomarker for systemic therapy for advanced prostate cancer.

2.2 Primary clinical efficacy objectives and endpoints

To determine whether therapy class choice based on a biomarker signature can improve PFS compared to standard of care in male patients with mHSPC and mCRPC, where standard of care is defined as clinician-patient treatment decision without access to information on the biomarker profile. For that purpose, we aim at identifying biomarker signature-therapy class combinations that are superior to the control group (SOC) in terms of PFS. Progression free survival (PFS) is defined according to disease stage at trial entry, using:

- For mHSPC: Time to development of castration-resistance according to EAU guidelines (see *'2.2.2.1 - Definition of progression-free survival for patients entering the mHSPC platform of ProBio'*)
- For mCRPC: Time to no longer clinical benefit (NLCB) according to PCWG3 guidelines (see *'2.2.2.2 - Definition of progression-free survival for patients entering the mCRPC platform of ProBio'*)

2.2.1 Motivation for choice of primary endpoint

The goal of ProBio is to learn as rapidly as possible which therapy classes are effective for which biomarker signatures. The primary endpoint should therefore be as fast as possible to evaluate and

robustly associated with the end goal of improved overall survival. Intermediate endpoints that translate to improved overall survival are traditionally challenging in advanced prostate cancer treatment trials. Metastatic prostate cancer is characterized by the dominance of bone metastases, which traditionally have been difficult to be objectively measured with radiographic imaging. Additionally, metastatic prostate cancer patients are also prone to the flare phenomenon, in which an increase of radionuclide uptake occurs due to healing lesions. This was most recently shown in a post hoc, retrospective secondary analysis of PREVAIL and AFFIRM (i.e. enzalutamide-treated mCRPC before and after docetaxel, respectively). In taxane-naïve men with mCRPC the detection of a new unconfirmed lesions on follow-up bone scans may represent pseudoprogression and was indicative of a favorable treatment response to enzalutamide (Armstrong, Al-Adhami, et al. 2019).

PCWG3 guidelines recommend using time-to-event endpoints. PREVAIL demonstrated strong associations between radiographic progression free survival (rPFS) and overall survival among men with mCRPC (Rathkopf et al. 2018). The regulatory approval criteria from the US FDA and European Medicines Agency require the demonstration of clinical benefit: an improvement in how a patient feels or functions or how long he or she survives. With the exception of the relief of pain, all the approvals for mHSPC and mCRPC have been based on the prevent/delay end points that reflect unequivocal benefit to a patient: prolongation of life or a reduction in pain (primarily due to skeletal-related events). Survival endpoints (overall and prostate cancer specific survival) and side effect endpoints are therefore necessary and constitute the most solid proof of benefit but take too long time to evaluate to be ideal as primary endpoints in ProBio (since ProBio is not a confirmatory trial). Nonetheless, upon long-term follow up, OS analyses will be performed as a secondary endpoint.

Additionally, since the ProBio trial has an interconnection between the mHSPC and mCRPC stage of the disease, and will also re-randomise patients who have entered ProBio in the mCRPC stage of the disease, the investigators will also evaluate PFS2. PFS2 is defined as the time from the initial study randomisation to the 2nd progression or death from any cause, and its usage is highly encouraged by the European Medicines Agency. Most recently the surrogacy of PFS2 for OS has been suggested in a retrospective analysis of published data from solid tumour clinical studies, demonstrating a strong positive correlation between both time-to-event endpoints (Mainwaring et al. 2019). These findings warrant further investigation and evaluation as secondary endpoint during the ProBio trial. Besides biochemical (i.e PSA) and clinical evaluation of the patient for our primary time-to-event endpoint PFS, also radiographic evaluation will take place at fixed intervals to determine disease progression. For overall trial conduct a local radiological response assessment will be used for the primary endpoint definitions, with all RECIST 1.1 evaluable responses/progressions to be confirmed by central review.

2.2.2 Measurement of progression-free survival (PFS)

2.2.2.1 Definition of progression-free survival for patients entering the mHSPC platform of ProBio

Previous RCTs in metastatic hormone-sensitive prostate cancer testing different combination therapy modalities (*Table 1 - Endpoint results from RCTs in metastatic hormone-sensitive prostate cancer*) have employed different progression criteria, such as PSA-PFS, clinical PFS, failure-free survival, radiographic PFS, time to pain progression, etc. which is in part due to the patient populations the different trials enrolled.

In the mHSPC setting the ProBio trial will target patients with newly diagnosed metastatic prostate cancer, and will use the following time-to-event definitions for PFS:

Time to development of castration-resistance, as defined by EAU guidelines (Cornford et al. 2017), i.e. whilst having castration levels of serum testosterone (<50ng/dL or 1.7 nmol/L) the time from randomisation to one of the following types of progression:

- Biochemical progression: Three consecutive rises in PSA, 1 week apart, resulting in two 50% increases over the nadir, and PSA >2 ng/ml

or

- Radiologic progression: The appearance of new lesions, either:

    - two or more new bone lesions on bone scan

      or

    - a soft tissue lesion using RECIST v1.1. (see *'APPENDIX 4: RESPONSE EVALUATION CRITERIA IN SOLID TUMORS'*).

  Radiologic progression will be evaluated by conventional imaging (i.e. bone scan and CT/MRI) or novel radionuclide imaging modalities if used consistently, at fixed intervals (i.e. every 6 months) or triggered by indication of clinical and/or biochemical progression (at the decision of the treating physician). For overall trial conduct a local radiological response assessment will be used for the primary endpoint definitions, with all RECIST 1.1 evaluable responses/progressions to be confirmed by central review.

2.2.2.2 Definition of progression-free survival for patients entering the mCRPC platform of ProBio

In the mCRPC setting the ProBio trial will target patients that are progressive upon enrolment in the mHSPC setting, or that have developed mCRPC (according to EAU guidelines) in SOC and were previously not enrolled in ProBio and need to initiate 1st line systemic therapy for mCRPC. Patients with mCRPC will be monitored up to 2 lines of systemic therapy, and will be evaluated by following time-to-event definitions for PFS:

Time to no longer clinical benefit (NLCB), as defined by Prostate Cancer Working Group (PCWG) 3 (see *'APPENDIX 5: PROSTATE CANCER WORKING GROUP 3 (PCWG3) CRITERIA'*), i.e. a new time-to-event measure that focuses on determining when a treatment should be discontinued when the patient is NLCB rather than strictly at the first evidence of progression. It is defined as the date and the specific reason(s) a therapy was ultimately discontinued, by evaluating the following types of progression:

- Biochemical progression: PCWG3 retains PCWG2 criteria, encompassing:

    - After decline from baseline: Time from start of therapy to first PSA increase that is ≥ 25% and ≥ 2 ng/mL above the nadir

    - No decline from baseline: PSA progression ≥ 25% increase and ≥ 2 ng/mL increase from baseline beyond 12 weeks

- Radiologic progression, defined as the time from random assignment to the date when the first site of disease is found to progress (using a manifestation-specific definition of progression), or death, whichever occurs first, encompassing:

    - CT for nodal/visceral disease: RECIST v1.1 (see *'APPENDIX 4: RESPONSE EVALUATION CRITERIA IN SOLID TUMORS'*)

    - Tc99 bone scan: At least two new lesions on first post-treatment scan, with at least two additional lesions on the next scan (2+2 rule)

  Radiologic progression will be evaluated by conventional imaging (i.e. bone scan and CT/MRI) or novel radionuclide imaging modalities if used consistently, at fixed intervals (i.e. every 8 weeks the first 24 weeks, followed by every 12 weeks) or triggered by indication of clinical and/or biochemical progression (at the decision of the treating physician). For overall trial conduct a local radiological response assessment will be used for the primary endpoint definitions, with all RECIST v1.1 evaluable responses/progressions to be confirmed by central review.

- Clinical progression, encompassing, but not limited to, the clinical judgment of:
    - AE or disease-related complication, clinical deterioration, presence of clinically meaningful pain, increase in analgesic consumption, etc.

2.3 Secondary clinical efficacy objectives and endpoints

To determine whether therapy class choices based on a biomarker signature can:

- improve PSA-PFS (according to PCWG3 criteria, see *'APPENDIX 5: PROSTATE CANCER WORKING GROUP 3 (PCWG3) CRITERIA'*)
- improve radiographic PFS (according to PCWG3 criteria, see *'APPENDIX 5: PROSTATE CANCER WORKING GROUP 3 (PCWG3) CRITERIA'*)
- improve treatment response rate (RR) after 3-4 months of treatment
- improve PFS2, defined as the time from the initial study randomisation to the 2nd progression or death from any cause
- improve overall survival, defined as the time from the initial study randomisation to death from any cause
- improve quality of life
- improve health economy
- does not increase toxicity (i.e. drug safety)
- To identify additional predictive and prognostic biomarkers
- Identify a certain therapy class that is superior for a certain biomarker signature compared to other therapy classes (efficacy)
- Identify superior treatment sequencing regimens (i.e. is treatment A followed by treatment B superior to treatment B followed by treatment A given a biomarker signature)

2.3.1 Measurement of treatment response rate (RR)

Response rates (RR) at 3-4 months on therapy, maximum RR within 3-4 months or the maximal change (rise or fall) at any time, in both mHSPC and mCRPC will be evaluated by the established international standards of:

- the Prostate Cancer Working Group version 3 (PCWG3) (see *'APPENDIX 5: PROSTATE CANCER WORKING GROUP 3 (PCWG3) CRITERIA'*)

and

- RECIST 1.1 criteria for soft tissue metastases (e.g. lung, liver and lymph nodes) (see *'APPENDIX 4: RESPONSE EVALUATION CRITERIA IN SOLID TUMORS'*).

Overall, RR will encompass, besides RECIST 1.1 for objective responses, also a decrease in PSA of 30% or more (PSA30), 50% or more (PSA50) or 90% or more (PSA90) from baseline. Additionally, for both PSA and response assessment by imaging, the proportion of patients who have not progressed at fixed time intervals (6, 12 and 24 months) will be reported. Finally, an overall confirmed response, defined as a composite of any of the following outcomes: radiological objective response (as assessed by RECIST 1.1) and/or a decrease in PSA of 50% or more (PSA50) from baseline will be evaluated. Any biochemical response needs to be confirmed by a second consecutive assessment at least 4 weeks later.

2.3.2 Quality of life objectives

Quality of life will be assessed using the following instruments, and is described in *'6.3 Patient-reported outcomes'*:

- EQ-5D-5L
- EORTC QLQ-C30
- BPI-SF

2.3.3 Safety and adverse events (AE) objectives

Common Terminology Criteria for Adverse Events (CTCAE, v5.0) will be used to record adverse events. Frequency and severity of adverse events (AE) will be evaluated, as described in *'8. PHARMACOVIGILANCE'.*

2.3.4 Cost-effectiveness objectives

Cost effectiveness will be assessed by using the EQ-5D-5L instrument to estimate health utilities. Treatment costs will be based on drug costs and reimbursement data.

2.4 Exploratory objectives

The following exploratory objectives will be addressed:

- Can ctDNA fraction dynamics replace radiographic and biochemical response evaluation for therapy response assessment?
- Can ctDNA fraction bursts predict therapy response?
- Retrospective analysis of the gene panel profile to identify new biomarker signature-treatment associations.
- Analysis of cell-free DNA methylomes
- Analysis of cell-free RNA and/or thrombocyte-derived RNA
- Prospective DNA analysis of CTCs

- RNA analysis of CTCs
- Prospective evaluation of clinical validity of PSMA-PET/CT-scan in 1st line mCRPC (CUTR-01 observational cohort study, PI: B. Sautois, CHU Liège, Belgium)
- Development of a new patient-reported outcome measure (PROM) instrument to evaluate the quality-of-life (QoL) of patients with advanced prostate cancer
- Quantitative analysis of androgen receptor perturbations using a blood-based liquid biopsy as a treatment-predictive biomarker for men with metastatic castration- resistant prostate cancer.

For more details on exploratory objectives, please see Trial Conduct Supplement *'AUXILIARY RESEARCH OBJECTIVES'*.

3. INVESTIGATIONAL PLAN

3.1 Rationale for trial design

Studies of patients with metastatic prostate cancer suggest that some have significant benefit from specific drugs while others appear to derive much less value. Because prostate cancer is a genetically and clinically heterogeneous disease, the ability to identify markers that predict early responders to treatment and long-term survival would markedly improve the prostate cancer treatment paradigm. At present, the upfront use of combination therapy, such as ADT plus docetaxel or abiraterone acetate plus prednisone, are considered as the new standard-of-care for the treatment of mHSPC, and will in the near future be expanded with anti-androgens such as apalutamide and enzalutamide. This raises the question which combination regimen needs to be initiated in which patient with mHSPC.

Additionally, it is unknown which treatment sequence will result in the longest overall survival once the patient progresses from the hormone-sensitive towards the castration-resistant stage of the disease. Post hoc subgroup analyses have looked into clinical characteristics (e.g. disease burden and prior or concurrent therapy exposure) to infer which patients derived the most benefit from the combination therapy in mHSPC. Nonetheless, these trials were not designed to infer which systemic therapy class (i.e. AR signalling inhibitor (ARSi) versus taxane-based chemotherapy) should be prescribed to which mHSPC patient. Although the available systemic therapies have improved the clinical outcome for patients with mHSPC and mCRPC, a personalised therapy selection using molecular biomarkers is lacking. The ProBio trial (EudraCT: 2018-002350-78, NCT03903835) is currently testing the concept of biomarker signature-driven therapy selection in the mCRPC setting. However, as multiple systemic agents to treat mCRPC are becoming available in the mHSPC setting, predictive biomarkers and patient selection in this patient population will experience the same necessity as for the treatment of mCRPC.

Hence, the ProBio trial will investigate both stages (i.e. mHSPC and mCRPC) of the disease in an interconnected manner, aiming to answer three key questions:

1. Which mHSPC patient population, defined by a biomarker signature, derives more benefit from chemohormonal versus ADT plus an additional ARSi (i.e. abiraterone acetate plus prednisone, enzalutamide or apalutamide) versus other experimental treatment modalities (e.g. combination therapy with niraparib plus abiraterone acetate plus prednisone)?

2. Upon development of mCRPC and during two lines of systemic therapy, which biomarker signature-therapy class combination results in the longest PFS?

3. Which treatment sequence going from de novo mHSPC towards 3rd line mCRPC results in the longest OS benefit?

The infrastructure of the ProBio trial enables us to take an important step toward systematically assigning pharmaceutical drugs and rapidly learning about the impact of these drugs or therapy classes on participants based on specific biomarker characteristics (signatures) of their tumors. It is critical that we shorten the knowledge turns for evaluating drugs and get the most promising drugs to those most likely to benefit. Assessing biomarker profile-therapy class combinations in the mHSPC and mCRPC setting, with progression-free survival as endpoint, will provide the proper time horizon for evaluation. The classic method of randomised trials evaluating one agent at a time for a set number of individuals is inefficient and will not allow to rapidly learn for whom drugs are most effective. To address this problem, we will use an outcome adaptive randomised trial based on biomarker signatures where multiple drugs can be evaluated simultaneously on a backbone of molecularly profiled participants.

Using Bayesian methods of adaptive randomisation, treatments or therapy classes will be assigned to participants who have higher probability of efficacy. Therefore, therapy classes which show the appropriate beneficial changes within a specific biomarker signature will be preferentially assigned within that signature and move through the trial more rapidly. Therapy class-biomarker signature combinations that do not show the likelihood of improved PFS rate will be dropped from the trial. The Bayesian predictive probability for each therapy class-biomarker signature combination of being superior to treatment of patients with the same signature in the control arm will be calculated for each possible signature. Therapy class-biomarker signature combinations will be dropped from the trial for futility when this probability drops sufficiently low. Therapy class-biomarker signature combinations will be graduated at an interim point should this probability reach a sufficient level. Those therapy class-biomarker signature combinations with high Bayesian predictive probability of being more effective than current clinical practice will graduate. The rationale for the ProBio platform study design is to learn from the data that accumulates in the trial and quickly generate solid hypotheses in a prospective way. To generate practice changing evidence, we will subsequently validate the promising therapy class-biomarker signature combinations in a confirmatory trial. When a therapy class graduates from the platform for a biomarker signature it will no longer be available in the active arms of the associated signature and will enter in a side trial nested within the ProBio platform. The control group for the biomarker signatures belonging to the graduating biomarker signature will be divided in two halves using fixed randomisation, the first receiving SOC and the other the graduating therapy class. Hence, the patients in the SOC arm will at this stage act as a comparator both for the therapy class in the confirmatory trial, and for the remaining active arms in the platform study. The confirmatory trial will be analyzed in a frequentist manner, and allow for early stopping (see *‘9. GENERAL STATISTICAL CONSIDERATIONS’* and Trial Conduct Supplement *‘STATISTICAL CONSIDERATIONS’*).

3.2 Overall trial design

ProBio is an outcome-adaptive, multi-arm, open-label, multiple assignment randomised biomarker driven platform trial in patients with metastatic hormone-sensitive and castration-resistant prostate cancer (*Figure 2 - The ProBio study design*). Importantly, the ProBio trial has an interconnection between the mHSPC and mCRPC platforms. Patients entering the trial in the mHSPC phase will be monitored throughout the course of their ADT combination therapy and up to 2 lines in the mCRPC setting, which will provide important insights in the disease trajectory and the overall life history of the advanced prostate cancer (Hong et al. 2015; Carreira et al. 2014). Alternatively, and upon request

and preference from the patient and treating physician, progressive patients with mHSPC and who have thus reached mCRPC may exit the trial or enter other RCTs, e.g. the MAGNITUDE trial (NCT03748641). Additionally, patients recently diagnosed with metastatic castration-resistant disease post prior systemic therapy for mHSPC or non-metastatic CRPC (nmCRPC) outside ProBio can immediately enter the mCRPC phase of ProBio.

Besides answering the clinical-relevant question which treatment sequence maximises OS in which patient subpopulation, the novel design will also result in an accurate assessment of the genetic landscape,the evolutionary behaviour and dynamics throughout the course of the disease, as it is influenced by therapeutic interventions. By clinically monitoring advanced prostate cancer tumours from diagnosis to relapse, and synchronously tracking the genetic trajectory in function of therapy, we believe this study can define the clinical impact of (sub)clonal heterogeneity and validate the stratification of patients based on molecular subgroups, bringing a more personalized medicine to this rapidly expanding patient population.

Patients will be randomised to control or experimental treatment arms. Patients in the control arm will receive standard of care following national guidelines. Importantly, once randomised to the control arm, allocated patients will remain in the control arm throughout subsequent re-randomisations during the course of trial. Patients in the experimental arm will be randomised to a drug within a therapy class based on a biomarker signature. Studied drug classes and investigational compounds are described in detail in chapter *'5. TREATMENT OF PARTICIPANTS'.*

The biomarker signature is defined as tumour properties or mutations in certain genes/pathways identified in the scientific literature as important in prostate cancer treatment response. The biomarker signatures are identified using a hybridisation capture gene panel specifically designed for advanced prostate cancer. Biomarker signatures tested during ProBio (i.e. both current and future) are described in detail in Trial conduct supplement *'BIOMARKER SIGNATURES'*, and encompass following genes/pathways or combinations thereof:

- Androgen receptor
- Homologous recombination repair deficiency (HRD)
- TP53
- TMPRSS2-ERG gene fusion
- Other biomarker signatures upon drug availability and protocol amendment, such as:
    - TMB-H/MSI+/CDK12-
    - PI3K pathway alterations

ProBio will use outcome-adaptive randomisation, adapting the randomisation based on progression-free survival within combinations of therapy classes and biomarker signatures. Treatments will initially be assigned to all biomarker signatures where they might be effective. The trial will be analyzed within a Bayesian framework, which allows for calculations of the probability for each therapy class that it is superior to standard of care within a given signature. Each experimental arm will be evaluated relative to patients in the control arm with the same biomarker signature. Participants and treating physicians will be blinded to the biomarker signature in the control arm. This information will thus not influence treatment choice among controls (reflecting today's standard of care).

Further, ProBio will use the sequential multiple assignments trial (SMART) concept, where each patient who progresses within the trial will re-enter the trial and be re-assigned to another therapy class based on the patient's current biomarker signature. The randomisation probabilities within the experimental arm are defined in proportion to the probability that each therapy class is superior to standard of care

for a given biomarker signature, and therefore change as data accumulates in the trial and knowledge accumulates for what biomarker signature specific therapy classes that are more likely to be effective. Trial results will be evaluated at a minimum biannually by an independent data and safety monitoring board (DSMB), or when is necessary due to decisions of graduating or dropping arms, or due to safety reasons. The DSMB will evaluate therapy class-biomarker signature combinations with respect to

Graduation for superiority: a therapy class-biomarker signature combination from the trial will be graduated if it has a Bayesian probability of superiority exceeding a pre-specified threshold.

Termination for futility: therapy class-biomarker signature combinations will be dropped from the trial for futility when success probabilities drop sufficiently low.

Alternatively, if the maximum sample size of 300 and 150 patients in the mHSPC and the mCRPC setting, respectively, is assigned to a therapy class-biomarker signature combination without graduation for superiority, assignments to that combination will end.
ProBio is a platform study. This means that new treatments and biomarker profiles can be added to the experimental arm in the future. This will be done after protocol amendments.

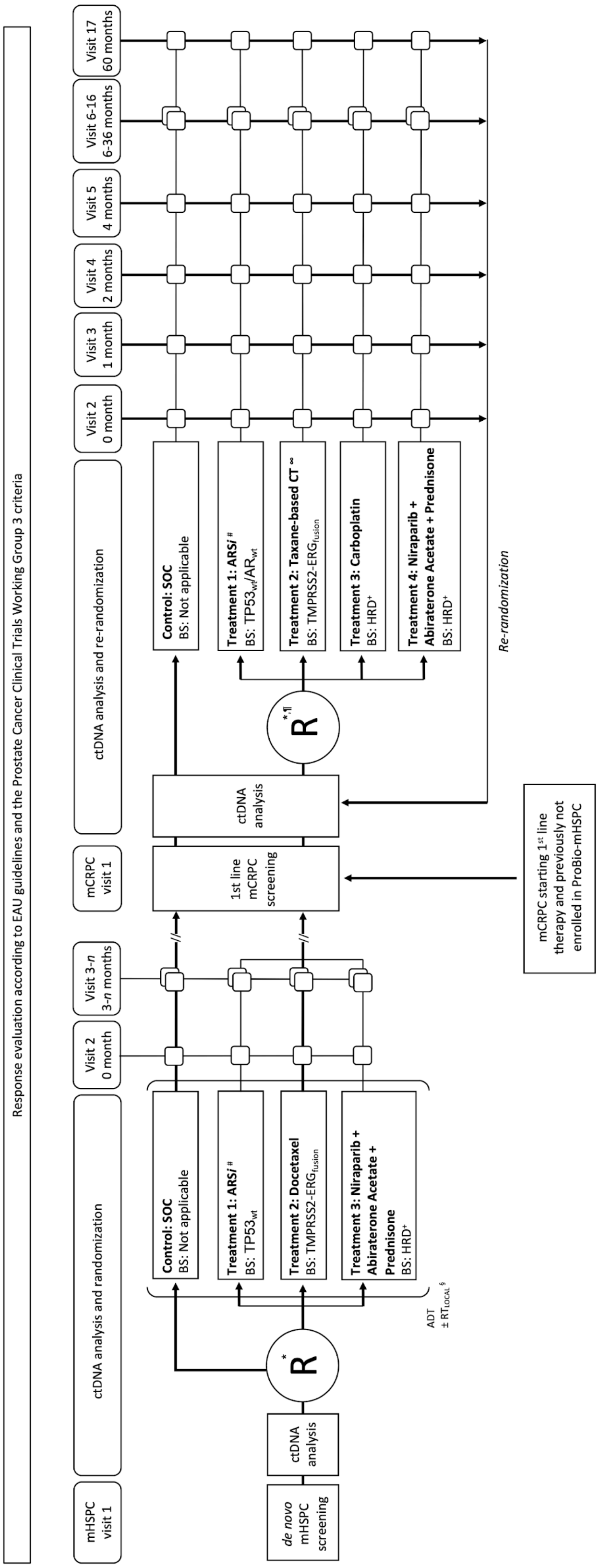

Figure 2 - The ProBio study design: *Bridging and tracking the disease trajectory from mHSPC to mCRPC. mHSPC: metastatic hormone-sensitive prostate cancer. mCRPC: metastatic castration-resistant prostate cancer. ctDNA: circulating tumour DNA. SOC: standard-of-care. * adaptive randomisation, § EBRT to the primary tumour in low-volume disease, ¶ not allowing a taxane-taxane or ARSi-ARSi sequence when patients are rapidly progressing from mHSPC to mCRPC, or going from first to second-line systemic therapy for mCRPC, # AR signalling inhibitor (ARSi) based on availability and reimbursement (e.g. abiraterone acetate plus prednisone, enzalutamide or apalutamide),∞ taxane-based chemotherapy (CT) based on availability and reimbursement (i.e. docetaxel or cabazitaxel).*

4. PATIENT SELECTION

4.1 Selection of trial population

The target patient population in the ProBio trial is focused on patients with metastatic hormone-sensitive (mHSPC) and metastatic castration-resistant (mCRPC) prostate cancer starting a new line of systemic therapy. Besides the enrolment of men with *de novo* mHSPC, who need to initiate systemic therapy, the study also allows to enrol progressive mHSPC patients who need to initiate first-line systemic therapy for mCRPC. Patients in the mCRPC setting are not eligible if they already have received systemic therapy for mCRPC, prior to study inclusion, thus warranting exclusive enrollment of mCRPC patients that need to initiate 1st line systemic therapy for castration-resistant disease. The patients will be selected from oncology or urology clinics. Each clinic will keep a log of all patients screened and included. The reason for screening failure should be stated for all patients screened but not included. The reason for withdrawal should be stated for all patients included but not completed.

4.1.1 Inclusion criteria

1. Male patients, aged above 18 years, with histologically confirmed prostate adenocarcinoma, initiating systemic therapy for metastatic disease, encompassing:

    - Metastatic hormone-sensitive prostate cancer (mHSPC)
      or
    - First-line mCRPC, i.e. first evidence of progressive metastatic prostate cancer under castrate levels (<50 ng/dL or 1.7 nmol/L) of serum testosterone, as defined by the EAU guidelines, encompassing:
        - Biochemical progression: Three consecutive rises in PSA 1 wk apart, resulting in two 50% increases over the nadir, and PSA >2 ng/ml and/or
        - Radiologic progression: The appearance of new lesions: either two or more new bone lesions on bone scan or a soft tissue lesion using the Response Evaluation Criteria in Solid Tumours
2. Distant metastatic disease documented by conventional imaging, i.e. positive bone scan or metastatic lesions on CT or MRI. Radiology taken within 6 weeks of screening may be used, if older a new scan needs to be taken. With the advent of novel imaging modalities using radionuclides, e.g. ^{68}Ga-PSMA-11 PET/CT, the ProBio trial will allow for future incorporation of these imaging modalities upon availability of validated guidelines, progression criteria and protocol amendment. Until then novel imaging cannot be used for inclusion and response/progression endpoints.
3. Adequate health, hematologic, hepatic, and renal function, as assessed by the investigator, to receive all available treatments in the trial in each disease state (mHSPC and mCRPC), i.e.
    - Haemoglobin ≥ 100 g/L (blood transfusion not less than 21 days prior to screening)
    - Absolute neutrophil count ≥ 1.5 x 10^9/L
    - Platelets ≥100 x 10^9/L
    - Total bilirubin < 1.5 ULN (patients with Gilbert's Syndrome bilirubin < 40 μg/L)
    - AST and ALT ≤ 1.5 ULN (or ≤ 3 ULN in the presence of liver metastases)
    - Serum creatinine not greater than 1 ULN (if serum creatinine is between 1 and 1.5 ULN, patients may be eligible provided that the calculated GFR is at least 50 ml/min measured directly by 24-hour urine sampling OR using Cockcroft-Gault method)

4. Albumin greater than or equal to 28 g/L
5. ECOG/WHO performance score 0-2

6. Agrees to use an effective contraceptive method during and up to 6 months after study drug treatment, and should not donate sperm during this period.
7. Able to understand the patient information and sign written informed consent.

4.1.2 Exclusion criteria

1. Other malignancies within 5 years except non-melanoma skin cancer
2. Within 6 months of randomisation: myocardial infarction, unstable angina, angioplasty, bypass surgery, stroke, TIA, or congestive heart failure NYHA class III or IV
3. Uncontrolled hypertension. Subjects with a history of hypertension are allowed provided blood pressure is controlled by anti-hypertensive treatment
4. Upon entering the mHSPC phase of the trial, prior systemic therapy (including ADT) is not allowed. Patients with mCRPC may not enter the trial when they have already received prior systemic therapy (with the exception of standard ADT) for mCRPC.
5. Any severe acute or chronic medical condition that places the patient at increased risk of serious toxicity or interferes with the interpretation of study results
6. Unable to comply with study procedures
7. Current participation in another clinical trial that will be in conflict with the present study, e.g. administration of an investigational therapeutic or invasive surgical procedure within 28 days prior to study enrolment. Imaging-based interventional trials are allowed as long as the conventional imaging intervals within ProBio are preserved.
8. Patients who are unlikely to comply with the protocol (e.g. uncooperative attitude, inability to return for subsequent visits) and/or otherwise considered by the Investigator to be unlikely to complete the study
9. Any condition or situation which, in the opinion of the investigator, would put the subject at risk, may confound study results, or interfere with the subject's participation in this study
10. Any medical condition that would make use of the study treatments contraindicated, according to the SmPC, e.g. significant heart or liver disease. The investigator should check the SmPC and/or IB for the assigned study treatments.
11. The determination of a biomarker signature is necessary to randomise patients during ProBio. Patients will therefore be excluded in case of:
    a. For patients with mHSPC: failure to detect ctDNA or somatic alterations from the primary tumour biopsies
    b. For patients with mCRPC: undetectable levels of ctDNA.

4.2 Patients treated with study drug(s) before (re-)randomisation

Patients treated earlier for mHSPC prior to study inclusion in the mCRPC platform of the trial, with any study drug cannot be randomised to receive the same drug in monotherapy, with exception of docetaxel rechallenge (*vide infra*). In terms of the systemic agents that will be studied, the investigators will avoid potential cross-resistance by e.g. not allowing a taxane-taxane or ARSi-ARSi sequence when patients are rapidly progressing from mHSPC to mCRPC, or going from first to second-line systemic therapy for mCRPC, when the inferred biomarker signature would not allow for it, or when it is deemed unfeasible based on clinical judgment of the treating physician.

Although the TROPIC trial showed activity of cabazitaxel in docetaxel-refractory mCRPC, and the CARD trial demonstrated the superiority of cabazitaxel in 3rd line mCRPC (encompassing predominately patients coming from an ADT-only regimen in mHSPC) post docetaxel and an ARSi (de Wit et al. 2019), potential cross-resistance between taxanes can occur (Lombard et al. 2017; van Soest et al. 2013). In the context of upfront chemohormonal therapy for mHSPC it was reported how abiraterone acetate

plus prednisone and enzalutamide had comparable therapeutic efficacy in 1st line mCRPC, whereas re-challenging with docetaxel resulted in low response rates (Francini et al. 2018; Lavaud et al. 2018). Nonetheless, retrospective data in mCRPC may indicate that docetaxel rechallenge may be effective in patients who initially responded to docetaxel and maintained a progression-free interval (PFI) of ≥6 months (Assi et al, 2020). During ProBio a more conservative PFI of ≥12 months will be employed to allow for docetaxel rechallenge. Importantly, besides a docetaxel rechallenge, the ProBio investigators will only allow a docetaxel-cabazitaxel sequence (de Wit et al. 2019), since data on the alternative sequence is lacking.

Although some activity for enzalutamide post abiraterone acetate plus prednisone in 'long-term' responders in the mCRPC setting has been observed (Johann S. de Bono et al. 2018), the post-hoc analysis of COU-AA-302 (i.e. abiraterone acetate plus prednisone in chemo-naive mCRPC) demonstrated limited benefit of ARSi after abiraterone acetate plus prednisone (Smith et al. 2017), which was most recently confirmed in phase II cross-over study with abiraterone acetate plus prednisone and enzalutamide (Khalaf et al. 2019). A similar observation has been made for enzalutamide, followed by or combined with abiraterone acetate plus prednisone (Attard et al. 2018). These treatment sequence insights were somewhat reflected in the treatment choices in LATITUDE patients who progressed on ADT plus abiraterone acetate plus prednisone, where docetaxel was the most common therapy prescribed once the patients reached the mCRPC state (Fizazi et al. 2019a). During the ProBio trial, ARSi-ARSi sequences (in the context of 2nd and new generation ARSi) will be avoided. However, the combination therapy abiraterone plus niraparib will be allowed after any other systemic therapy (with exception of abiraterone monotherapy).

Next, the ProBio mHSPC platform will initially utilise abiraterone acetate plus prednisone and apalutamide as only ARSi, based on current availability and reimbursement. Protocol amendments will be made once other ARSi, such as enzalutamide and darolutamide, become available. However, for the antiandrogens we expect similar clinical benefits, based on very comparable chemical structures (Higano 2019), and reported outcome data in both the mHSPC (TITAN (Chi et al. 2019), ENZAMET (I. D. Davis et al. 2019)) and non-metastatic CRPC (PROSPER (Hussain, Fizazi, et al. 2018), SPARTAN (Smith et al. 2018)) setting. Therefore, ProBio will investigate patient allocation based on drug or therapy class (e.g. ARSi versus taxane-based chemotherapy) in context of different biomarker signatures.

Additionally, we allow for external beam radiotherapy (EBRT) to the primary tumour in case of low metastatic burden as this is nowadays considered standard-of-care. Low-volume disease can be defined per CHAARTED definition (i.e. ≠ ≥4 bone metastases with one or more outside the vertebral bodies or pelvis, or visceral metastases, or both) (C. C. Parker et al. 2018) per STOPCAP M1 definition (i.e. <5 or ≥5 bone metastases) (Burdett et al. 2019), or according to the STAMPEDE M1|RT comparison definition (i.e. patients with only lymph node metastases or < 4 bone metastases (± lymph nodes), regardless of location (Ali et al. 2019). The incorporation of which definition will be defined by the SOC at that time, and will be updated upon protocol amendment.

4.3 Removal of trial patients from treatment or assessments

A patient should be withdrawn from the trial treatment if, in the opinion of the Investigator, it is medically necessary, or if it is the expressed wish of the patient. Patients are free to discontinue their participation in the trial at any time. The electronic case report form (eCRF) should be completed as far as possible and data entered will be verified by the monitor. This former will be achieved by completing the 'Unscheduled Event' and 'Treatment discontinuation' forms in the SMART-TRIAL eCRF system (see trial conduct supplement *'SMART-TRIAL eCRF STANDARD OPERATING PROCEDURES'*).

A withdrawn patient is not allowed to re-enter into the trial. A withdrawn patient will not be replaced. The criteria for withdrawal are the following:

- Patient wishes to discontinue
- Unacceptable AE/toxicity, including laboratory values
- Non-compliance with the study protocol
- Patient refuses to cooperate
- Other medical reasons
- Unblinding of biomarker profile

5. TREATMENT OF PARTICIPANTS

5.1 Standard-of-care androgen deprivation therapy (ADT) as background systemic therapy

Upon blood collection during the trial screening phase of the mHSPC platform, or during any timepoint in the mCRPC platform of ProBio, the patient will continuously receive androgen deprivation therapy (ADT) according to standard-of-care. The choice of the prescribed GnRHa, to achieve castrate levels of testosterone (≤50 ng/dL), is at the discretion of the investigator and dosing should be consistent with the prescribing information. All GnRHa therapies should be recorded in the patient's medication section in the SMART-TRIAL eCRF system. Subjects are permitted to undergo a bilateral orchiectomy instead of ADT with a GnRHa during the study.

5.2 Identity of Investigational Medicinal Product (IMP)

The Investigator must ensure that the IMP will only be used in accordance with the IMP-specific subprotocol, which provides information on the background (using SmPC/IB), Distribution, Packaging, Dosage and Safety Assessments. Patients in the experimental arms can be randomised to the following therapy classes and IMPs, depending on national guidelines, availability and reimbursement criteria:

For mHSPC:

- AR signalling inhibitors (ARSi, see *'SUB-PROTOCOL: STANDARD-OF-CARE IMP'*)
  - Abiraterone acetate plus prednisone
  - Apalutamide
  - Other ARSi upon approval from authorities and protocol amendment

- Taxane-based chemotherapy (see *'SUB-PROTOCOL: STANDARD-OF-CARE IMP'*)
  - Docetaxel

- Other investigational agent(s) sponsored by pharmaceutical company
  - Niraparib plus abiraterone acetate plus prednisone (prednisone will be used as concomitant medication; see *'SUB-PROTOCOL: NIRAPARIB PLUS ABIRATERONE ACETATE PLUS PREDNISONE'*)

- Other agents or drug classes upon sub protocol amendment, e.g.
  - Checkpoint inhibitor
  - PI3K pathway inhibitor
  - PSMA-targeted therapy

For mCRPC:

- AR signalling inhibitors (ARSi, see *'SUB-PROTOCOL: STANDARD-OF-CARE IMP'*)
    - Enzalutamide
    - Abiraterone acetate plus prednisone (or other glucocorticoid at the discretion of treating physician)

- Taxane-based chemotherapy (see *'SUB-PROTOCOL: STANDARD-OF-CARE IMP'*)
    - Docetaxel
    - Cabazitaxel
- Platinum-based chemotherapy
    - Carboplatin (see *'SUB-PROTOCOL: CARBOPLATIN'*)

- Other investigational agent(s) sponsored by pharmaceutical company:
    - Niraparib plus abiraterone acetate plus prednisone (prednisone will be used as concomitant medication; see *'SUB-PROTOCOL: NIRAPARIB PLUS ABIRATERONE ACETATE PLUS PREDNISONE'*)

- Other agents or drug classes upon sub protocol amendment, e.g.
    - Checkpoint inhibitor
    - PI3K pathway inhibitor
    - PSMA-targeted therapy

5.3 Treatment compliance

Country-specific drug accountability procedures and logs will be used at a site and pharmacy level to register expiry dates, lot and batch numbers of administered IMP, dates of dispensation, preparation and administration, dates of returned medication, and the number of remaining pills upon return. Additionally, the treatment compliance will be assessed by the investigator, treating physician and/or trial nurse at each study visit by asking if the patients received and used the treatment as prescribed.

5.4 Prior and concomitant therapy

Other cancer therapy that is considered necessary for the patient's welfare may be given at the discretion of the Investigator. Such an example is the administration of Bone Health Agents (e.g. zoledronic acid or denosumab) or performing external radiotherapy for uncontrolled pain from bone metastases, or external radiotherapy of the prostate in case of low-volume de novo mHSPC.
Detailed conditions for the use of the study treatments and contraindications, special warnings and precautions for use are described in accordance with the marketing authorization in the Summary of Product Characteristics (SmPC) for the study treatments. All concomitant therapy, in the categories of steroids (e.g. prednisone in context of abiraterone acetate), bone health agents (e.g. denosumab) and analgesics, that used during the trial period must be recorded in the patients' Medication section in eCRF system (see trial conduct supplement *'SMART-TRIAL eCRF STANDARD OPERATING PROCEDURES'*). No other drug under investigation may be used concomitantly with the trial medication.

5.5 Treatment procedures during and upon subject (re-)randomisation

ProBio patients can follow different pathways within the trial, which not only depends on their biomarker subgroup combinations, but also on the timing of randomisation and in which arm the patient was initially randomised to. An overview of the most relevant pathways for a ProBio patient is provided in *Figure 3 - Treatment procedures*. Patients are excluded if their biomarker signature cannot be inferred (e.g. due to undetectable ctDNA or technical failure). Patients will be randomized either to the control group (SOC) or one of the experimental biomarker signature-therapy combination arms. As patients might be unfit or unwilling to continue the trial, both the patient and treating physician might choose to discontinue the patient and exit the trial. Upon progressive disease, patients initially randomised to one of the biomarker signature-therapy combination arms will be rerandomized to another biomarker signature-therapy combination arm after a new liquid biopsy profile was generated. During the 12-15 day turnaround time to generate a new liquid biopsy profile it is at the discretion of the treating physician to completely stop or continue the prior systemic therapy on which the patient is currently progressive. In contrast, patients initially randomised to the control group will remain in their arm upon progression (unless graduated biomarker signatures-therapy combinations are available) and will immediately receive a new line of physicians' choice SOC therapy. In the later stage of the trial, if the biomarker subgroup combination of a new patient belongs to one of the graduating biomarker signatures, the patient will enter the confirmatory trial with fixed randomization to the control (SOC) or the graduating active treatment. Finally, upon progressive disease after the second or third randomization, depending if the patient entered ProBio in the mCRPC or mHSPC setting respectively, all patients will discontinue and exit the ProBio trial for the PFS endpoints. Long-term follow for overall survival (OS) analysis will take place via the national cancer registries by biannual communication with the coordinating and/or site principal investigators.

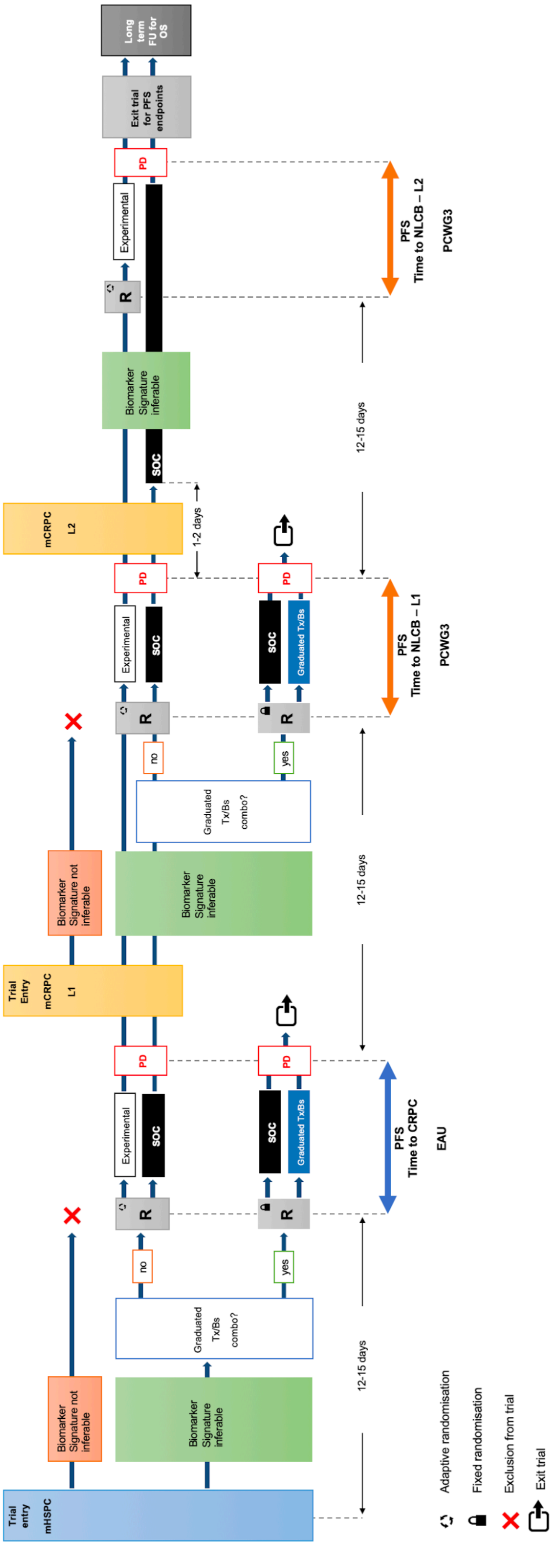

Figure 3 - Treatment procedures. *The patient pathways in context of absence or presence of graduated therapy-biomarker signature combinations (Tx/Bs combo) with consideration of initial randomisation arm during re-randomisation. Patients may enter the metastatic hormone-sensitive prostate cancer (mHSPC) phase of the ProBio trial, and will be followed till metastatic castration-resistant prostate cancer (mCRPC) onset, and during 2 lines of systemic therapy in mCRPC. Alternatively, patients previously treated within the SOC outside of ProBio and that have developed mCRPC can immediately enter the mCRPC phase of the ProBio trial, and will be followed during 2 lines of systemic therapy in mCRPC. SOC: Standard-of-care; PD: progressive disease; R: randomisation; L1: first line; L2: second line; PFS: Progression-free survival; OS: overall survival; EAU: European Association of Urology; NLCB: no longer clinically benefitting; PCWG3: Prostate Cancer Clinical Trials Working Group 3*

5.6 Treatment procedures upon intolerance

In case of intolerance, whilst responding (at the discretion of the treating physician), to one of the IMPs throughout the course of the trial, the patient will be followed up with surveillance, not necessarily requiring a new line of systemic therapy. IMP-specific procedures upon intolerance are described in the drug-specific subprotocols, SmPC and IBs. In general, the initially initiated therapy is paused or stopped, which is registered in the patient's medication form in the SMART-TRIAL eCRF system (see trial conduct supplement *'SMART-TRIAL eCRF STANDARD OPERATING PROCEDURES'*). Initiating a new line of systemic therapy upon re-randomisation will only take place upon documented and registered progressive disease of the prior line of systemic therapy.

6. PATIENT LEVEL TRIAL ASSESSMENTS

6.1 Demographics, baseline characteristics and medication use

Following parameters and information must be documented in the SMART-TRIAL eCRF system during the Screening and Baseline Assessment procedures, as well as during data event visits throughout the course of trial:

- Demographics
- Physical characteristics (weight, height, blood pressure and heart rate) and ECOG performance status
- All relevant medical, surgical and histopathological history since prostate cancer diagnosis
- Sites, extent and sizes of metastases
- Blood hematology and biochemistry
- Patient Reported Outcome (PRO) measures:
    - EQ-5D-5L
    - EORTC QLQ-C30
    - BPI-SF
- Medication use
    - Antitumor agents
        - Investigational Medicinal Product (IMP)
        - Androgen deprivation therapy
    - Concomitant medication
        - Bone health agents
        - Glucocorticoid regimens
        - Analgesics

6.2 Vital signs and physical examination

A physical examination, encompassing measurements of heart rate, blood pressure and weight, together with an evaluation of the patient's performance status (see *'APPENDIX 2: ECOG PERFORMANCE STATUS'*) will be measured at each study visit. Physical examinations will be directed at detecting adverse events. Physical examinations should be performed by the same evaluator throughout the study, whenever possible. Any clinically significant change in physical findings noted during the study should be reported as an adverse event. Any clinically significant abnormalities persisting at the end of the study will be followed by the investigator until resolution or until reaching a clinically stable endpoint.

6.3 Patient-reported outcomes

Quality of life will be assessed using following instruments:

The EORTC QLQ-C30 is a questionnaire developed to assess the quality of life of cancer patients. It is a copyrighted instrument, which has been translated and validated in over 100 languages and is used in more than 3,000 studies worldwide. Presently QLQ-C30 Version 3.0 is the most recent version and will be used.

The EQ-5D-5L (EuroQol Group, www.euroqol.org) is a validated tool that measures mobility, self-care, usual activities, pain, discomfort, and anxiety/depression. The EQ-5D quality of life instrument consists of a 5-item questionnaire and a visual analogue scale ranging from 0 (worst imaginable health state) to 100 (best imaginable health state) that integrates many aspects of the subject's disease process into a single assessment. Cost effectiveness will be assessed by using the EQ-5D-5L instrument to estimate health utilities. Treatment costs will be based on drug costs and reimbursement data.
The Brief Pain Inventory - Short Form (BPI-SF) is a 9-item self-administered questionnaire used to evaluate the severity of a patient's pain and the impact of this pain on the patient's daily functioning. The patient is asked to rate their worst, least, average, and current pain intensity, list current treatments and their perceived effectiveness, and rate the degree that pain interferes with general activity, mood, walking ability, normal work, relations with other persons, sleep, and enjoyment of life on a 10 point scale. The brevity of the BPI-SF makes it suitable for settings in which pain is assessed on a frequent basis (e.g. in a randomised control trial).

6.4 Patient sample collection for routine laboratory measurements and biobanking of liquid biopsies

The handling and analysis of the blood samples will be made according to the local routines at the site.

- Sample collections for routine clinical laboratory analysis:

    - One 2-4 ml EDTA blood tube will be drawn to analyze
        - Haemoglobin
        - CBC: complete blood count profile
            - platelet count
            - red blood cells
            - total white blood cells
            - absolute neutrophil counts
            - absolute lymphocyte counts
            - absolute monocyte counts

    - One 2-4 ml lithium heparin blood tube will be drawn to analyse
        - Testosterone
        - PSA
        - Creatinine
        - Albumin
        - Bilirubin
        - lactate dehydrogenase (LDH)
        - alkaline phosphatase (ALP)
        - liver enzymes:
            - alanine aminotransferase (ALT/SGPT)
            - aspartate aminotransferase (AST/SGOT)

- <u>Sample collections for liquid and/or tissue biopsy biobanking for downstream molecular profiling:</u>

  - Genomic profiling of cell-free DNA and germline DNA: Blood will be collected in 2 x 10 ml preservative tubes such as Streck Cell-Free DNA BCT tubes. The Streck tubes will be sent with regular mail to the to the country's centralised Biobank (i.e. University Hospital or University from the national PI) for harvesting of plasma, extraction of cell-free DNA and extraction of germline DNA from the remaining white blood cell pellet.

  - 3 x 10 ml blood for exploratory objectives will be collected in either preservative tubes such as Streck cell-free DNA BCT, EDTA tubes or selected tubes tailored for the exploratory objectives. Blood will be stored in regional biobanks or participating sites may choose to ship blood for biobanking to the country's centralised Biobank (i.e. University Hospital or University from the national PI).

  - Other specific samples can be collected in accordance with local/regional standard of care procedures. 25-50 mL of first-catch urine will be collected in designated collection cups for exploratory retrospective biomarkers studies. Urine will be stored in regional biobanks or participating sites may choose to ship urine for biobanking at the country's centralised Biobank (i.e. University Hospital or University from the national PI).

  - Diagnostic prostate biopsies from men with de novo mHSPC. 4 sections x 10 µm section thickness, >2 mm total length of cancer. Biopsies with <50% need to be macro dissected.

7. STUDY PROCEDURES

7.1 Technical pilot study prior to study initiation

To make sure that the handling of blood samples taken at the participating centers and further sent for molecular profiling is working as expected and with high quality, a technical pilot study may be performed before the actual start of the ProBio study at the center. Therefore, 3-5 patients at participating centers with metastatic prostate cancer will be asked to donate extra blood during routine blood sampling for disease follow-up at the center. The blood samples collected in the technical pilot study will not be used in the main study and will be destroyed after analysis. A nurse from each site will talk to a patient and inform about the technical pilot study and if the patient agrees to participate in the technical pilot study he will sign the written informed consent before any study related procedures are performed. Blood will be drawn according to study procedures. 2 x 10 ml preservative tubes such as Streck Cell-Free DNA BCT tubes will be used for prospective molecular profiling. In the technical pilot, we will evaluate:

1. Sample logistics including time to arrival at the country's centralised Biobank (i.e. University Hospital or University from the national PI).
2. Sample quality after shipment to the country's centralised Biobank (i.e. University Hospital or University from the national PI).
3. That molecular profiling is finalized within the expected time frame (2 weeks)

7.2 Sample handling and liquid/tissue biopsy profiling during ProBio

Patients will consent to the study, allowing ProBio Investigators to draw blood and collect urine samples at any of the participating hospitals, according to study procedures. In the *de novo* mHSPC setting of ProBio the investigators will also collect tumor tissue sections from the archival diagnostic biopsies to complement the ctDNA analysis to infer the biomarker signatures. Hence, tumour-marked sections of diagnostics biopsies will be requested from the departments of Pathology for all patients entering the mHSPC platform of the trial. In terms of sample handling and processing, the ProBio trial allows for different scenarios:

1. Transport of whole blood samples, urine and tissue sections to Karolinska Institutet
2. Local blood pre-processing and transport of plasma and blood cell pellets to Karolinska Institutet
3. Local blood pre-processing and extractions, with transport of extracted cfDNA and gDNA to Karolinska Institutet
4. Local blood pre-processing, extractions and NGS, followed by data transfer, analysis and variant curation at the Karolinska Institutet
5. Local blood pre-processing, extractions, NGS, and data analysis with variant curation at the Karolinska Institutet
6. Local blood pre-processing, extractions, NGS, data analysis and variant curation

All sites will be provided with dedicated ProBio boxes for transport of patient samples. Patient samples ready for transport will be deposited at a specific location in the participating hospital, after which a sample pick-up will be ordered online with a dedicated courier service company per participating country (*Table 3 - Country-specific biobanking infrastructure and national logistics services*). Notification per email of every pick-up per site will take place, dependent on national services and agreements with the transport company.

Table 3 - Country-specific biobanking infrastructure and national logistics services

**Country**	**Central biobank**	**Logistics Service**
Sweden	KI Biobank, Karolinska Institutet, Stockholm	World Courier (Sweden) AB<br>Söderbyvägen 1A<br>SE-195 25 Arlandastad<br>jkrohne@worldcourier.se
Belgium	Hiruz Biobank, University Hospital Gent<br>Corneel Heymanslaan 10, 9000 Gent<br>hiruz.biobank@UZGent.be	Inter Healthcare Transport (IHCT)<br>Motstraat 40<br>B-2800 Mechelen<br>09/216.20.63<br>ihct@hendriks.be
Norway	Akershus University Hospital Research Biobank	World Courier (Sweden) AB<br>Söderbyvägen 1A<br>SE-195 25 Arlandastad<br>jkrohne@worldcourier.se
Switzerland	Department of Urology<br>University Hospital Basel<br>Spitalstrasse 32<br>4031 Basel<br>Switzerland	To be completed upon protocol RA/EC submission

Upon collection of patient samples, the courier will transport all samples to the country's centralised Biobank (i.e. University Hospital or University from the national PI) for storage and preparation of

plasma, blood cell controls and other analytes depending on the auxiliary research projects. In countries without biobanking infrastructure, blood samples will be sent individually or batchwise directly to Karolinska Institutets biobank (Stockholm, Sweden) for processing. Sample processing for downstream molecular characterization encompasses the generation of plasma and WBC from blood samples collected in preservative tubes such as Streck cell-free DNA BCT tubes (i.e. 2 x 10 mL). Next, cfDNA and germline DNA will be extracted followed by standard quality control steps (i.e. e.g. yield and integrity measurements). All extraction and standard quality control procedures will occur either at the country's centralised Biobank or at Karolinska Institutets biobank. Countries with centralized processing, will ship prepared cfDNA and germline DNA samples to the Department of Medical Epidemiology and Biostatistics (MEB) at Karolinska Institutet (Stockholm, Sweden). Prospective molecular profiling will be performed on extracted cell-free DNA and germline DNA. A detailed description of the molecular profiling and biomarker signature definitions is provided in Trial conduct supplement *'BIOMARKER SIGNATURES'*.

7.3 Procedures during patient study visits

Patients at participating centers with metastatic hormone-sensitive (mHSPC) and castration-resistant prostate cancer (mCRPC) will be screened for participation. Patients fulfilling the inclusion and exclusion criteria and willing to participate will be included. A nurse from each site will call/talk to potential study participants to inform about the study. To be able to monitor screening performance at each site, the nurse will also ask for consent to save basic data on these pre-screened participants. Depending on which stage of the disease the patient enters the trial, different schedules of activities, i.e. study or data event visits, are applied as described below. Besides the described study visits, the patient will continue to have his routine visits for e.g. routine blood analysis, from which the results will also be collected via the SMART-TRIAL eCRF system (see trial conduct supplement *'SMART-TRIAL eCRF STANDARD OPERATING PROCEDURES"*).

7.3.1 Patients entering the mHSPC platform from the ProBio trial

7.3.1.1 Visit 1 (Screening - Phase 1)

Firstly, men with suspicion of metastatic prostate cancer will be invited to donate blood for biomarker research and to potentially participate in the ProBio trial. The Investigator will explain to the patient the nature and aim of the study, its procedures, possible side effects, requirements and restrictions. The purpose of this phase in the screening procedure is to identify patients that are eligible for the study, draw blood for routine clinical laboratory analysis and biobanking prior to initiating androgen deprivation therapy (ADT).

The following procedures will be performed during visit 1:

- Obtain signed written informed consent for participation in the study
- Record results from latest routine clinical laboratory analysis
    - Routine blood analysis results taken within 2 weeks of inclusion may be used, if older a new blood collection for routine analysis needs to be performed.
- Collect 2 x 10 ml blood for prospective ctDNA analyses in preservative tubes such as Streck cell-free DNA BCT tubes and 3 x 10 ml blood for exploratory objectives in either preservative tubes such as Streck cell-free DNA BCT, EDTA tubes or selected tubes tailored for the exploratory objectives. Blood will be stored in regional biobanks or participating sites may choose to ship blood for biobanking to the country's centralised Biobank (i.e. University Hospital or University from the national PI).

- Collect 25-50 mL first-catch urine in designated collection cups for exploratory objectives. Urine will be stored in regional biobanks or participating sites may choose to ship urine for biobanking at the country's centralised Biobank (i.e. University Hospital or University from the national PI).
- Collect tissue sections from all diagnostic prostate cancer core biopsies
- Order and perform conventional imaging.
- Upon blood collection, androgen deprivation therapy (ADT) may be started
    - The choice of the prescribed GnRHa, to achieve castrate levels of testosterone (≤50 ng/dL), is at the discretion of the investigator and dosing should be consistent with the prescribing information. All GnRHa therapies should be recorded in the patient's medication section in the SMART-TRIAL eCRF system. Subjects are permitted to undergo a bilateral orchiectomy instead of ADT with a GnRHa during the study.
    - ADT may be postponed to Visit 2, if imaging results are considered essential by the treating physician

7.3.1.2 Visit 2 (Screening - Phase 2)

After diagnosis and staging is done and if metastatic disease is detected by imaging, the patient will be enrolled in the study. The patient's biobanked plasma, tissue and germline DNA samples prior to ADT are retrieved from the biobank and processed for biomarker signature analysis and clinical characteristics and data are collected.

The following procedures will be performed during visit 2:

- Check that the patient fulfils all inclusion criteria and none of the exclusion criteria
- Complete SMART-TRIAL eCRF Inclusion & Baseline Assessment Data events, recording among others:
    - Patient demographic information
    - ECOG status
    - Patient medical and tumor characteristics
    - Patient concomitant medication
    - Results from latest CT/MR scans (thorax and abdomen) and bone scintigraphy.
- Collect 2 x 10 ml blood for future ctDNA analyses in in preservative tubes such as Streck cell-free DNA BCT tubes and 3 x 10 ml blood for exploratory objectives in either in preservative tubes such as Streck cell-free DNA BCT, EDTA tubes or selected tubes tailored for the exploratory objectives. Blood will be stored in regional biobanks or participating sites may choose to ship blood for biobanking to the country's centralised Biobank (i.e. University Hospital or University from the national PI).
- Collect 25-50 mL first-catch urine in designated collection cups for exploratory objectives. Urine will be stored in regional biobanks or participating sites may choose to ship urine for biobanking at the country's centralised Biobank (i.e. University Hospital or University from the national PI).
- Record Patient Reported Outcome (PRO) measures:
    - EQ-5D-5L
    - EORTC QLQ-C30
    - BPI-SF
- Complete SMART-TRIAL eCRF Data event

7.3.1.3 Randomisation

Patients who fulfil all inclusion criteria, none of the exclusion criteria, and where all data is available for randomisation (i.e. ctDNA and/or tissue biopsy analysis completed, biomarker signature determined, results from CT/MR scans (thorax and abdomen) and bone scintigraphy available, PSA-value, etc.) will be randomised to either control arm (standard of care) or one of the experimental arms.

7.3.1.4 Visit 3 (treatment start, time = 0 months ± 1 week)

The combination treatment (i.e. ADT (which was already initiated during visit [1] or [2]) + investigational agent) period begins as soon as possible after randomisation.

The following procedures will be performed during the visit 3:

- Inform patient on treatment choice
    - In case of randomisation to a non-approved therapy for prostate cancer (i.e. non SOC) , a Drug-Specific patient information document and ICF will be provided to the patient
- Record patient concomitant medication
- Collect blood for routine clinical laboratory analysis
- Collect 2 x 10 ml blood for future ctDNA analyses in in preservative tubes such as Streck cell-free DNA BCT tubes and 3 x 10 ml blood for exploratory objectives in either in preservative tubes such as Streck cell-free DNA BCT, EDTA tubes or selected tubes tailored for the exploratory objectives. Blood will be stored in regional biobanks or participating sites may choose to ship blood for biobanking to the country's centralised Biobank (i.e. University Hospital or University from the national PI).
- Collect 25-50 mL first-catch urine in designated collection cups for exploratory objectives. Urine will be stored in regional biobanks or participating sites may choose to ship urine for biobanking at the country's centralised Biobank (i.e. University Hospital or University from the national PI).
- Record patient Patient Reported Outcome (PRO) measures:
    - EQ-5D-5L
    - EORTC QLQ-C30
    - BPI-SF
- Start combination treatment
- Complete SMART-TRIAL eCRF Data event

7.3.1.5 Visit 4 (time = month 1 ± 1 week)

Data collection for exploratory analyses.

<u>The following procedures will be performed 1 week before visits [4]:</u>

- Collect blood for routine clinical laboratory analysis

<u>The following procedures will be performed during the visit 4:</u>

- Collect 2 x 10 ml blood for future ctDNA analyses in in preservative tubes such as Streck cell-free DNA BCT tubes and 3 x 10 ml blood for exploratory objectives in either in

preservative tubes such as Streck cell-free DNA BCT, EDTA tubes or selected tubes tailored for the exploratory objectives. Blood will be stored in regional biobanks or participating sites may choose to ship blood for biobanking to the country's centralised Biobank (i.e. University Hospital or University from the national PI).
- Collect 25-50 mL first-catch urine in designated collection cups for exploratory objectives. Urine will be stored in regional biobanks or participating sites may choose to ship urine for biobanking at the country's centralised Biobank (i.e. University Hospital or University from the national PI).
- Record patient concomitant medication
- Record patient Patient Reported Outcome (PRO) measures:
    - EQ-5D-5L
    - EORTC QLQ-C30
    - BPI-SF
- Assess patient AEs
- Check patient compliance to medications, by asking the patient
- Response evaluation
    - Conduct and record biochemical/clinical response evaluation
- Complete SMART-TRIAL eCRF Data event

7.3.1.6 Visit [5, 6, 7, 8, etc.] (time = month 3, 6, 9, 12, etc. ± 3 weeks)

Response evaluation. During the treatment period, the patient will be assessed by the treating physician every 3 months until progressive disease, with conventional imaging being performed every 6 months.

<u>The following procedures will be performed 1 week before every 3-month visit [5, 6, 7, 8, etc.]</u>

- Collect blood for routine clinical laboratory analysis

<u>The following procedures will be performed 1 week before before every 6-month visit [6, 8, 10 ,12, 14, etc.]:</u>

- Perform conventional imaging every 6 months, i.e. at month 6, 12, 18, etc.

<u>The following procedures will be performed during every 3-month visit [5, 6, 7, 8, etc.] until progressive disease:</u>

- Collect 2 x 10 ml blood for future ctDNA analyses in in preservative tubes such as Streck cell-free DNA BCT tubes and 3 x 10 ml blood for exploratory objectives in either in preservative tubes such as Streck cell-free DNA BCT, EDTA tubes or selected tubes tailored for the exploratory objectives. Blood will be stored in regional biobanks or participating sites may choose to ship blood for biobanking to the country's centralised Biobank (i.e. University Hospital or University from the national PI).
- Collect 25-50 mL first-catch urine in designated collection cups for exploratory objectives. Urine will be stored in regional biobanks or participating sites may choose to ship urine for biobanking at the country's centralised Biobank (i.e. University Hospital or University from the national PI).
- Record patient concomitant medication
- Record Patient Reported Outcome (PRO) measures:
    - EQ-5D-5L
    - EORTC QLQ-C30
    - BPI-SF

- Assess patient AEs
- Check patient compliance to medications
- Response evaluation.
    - Conduct and record response evaluation based on clinical benefit (physician assessment), PSA and radiology (CT/bone scintigraphy)
    - Record response evaluation as Progressive disease, Stable Disease or Response. If progressive disease is present, the study drug is stopped, and evaluation is done to decide if the patient can be re-randomised towards the mCRPC platform of the ProBio trial. If stable disease or treatment response is present, the treatment continues on study drug.
- Complete SMART-TRIAL eCRF Data event

7.3.2 Schedule of activities in mHSPC

Activities	Screening		Randomisation	Follow up during Treatment Period till mCRPC development (EAU guidelines)														
Patient visits	1	2		3*	4	5	6	7	8	9	10	11	12	13	14	15	16	
Time from treatment start (months)	-1	-0.5		0	1	3	6	9	12	15	18	21	24	27	30	33	36	Every 3 months
Vist windows (weeks)	0	±1		±1	±1	±3	±3	±3	±3	±3	±3	±3	±3	±3	±3	±3	±3	±3
Inclusion/exclusion criteria		X																
Informed consent	X																	
Medical and disease history		X																
Conventional imaging (Bone scan/CT or MRI) ¶	X						X		X		X		X		X		X	Every 6 months
Routine blood-based biomarkers (e.g., CBC, PSA, LDH, ALP, etc.) ¶	X	X		X	X	X	X	X	X	X	X	X	X	X	X	X	X	Every 3 months
Blood/urine liquid biopsy	X	X		X	X	X	X	X	X	X	X	X	X	X	X	X	X	Every 3 months
Collection tissue specimens from diagnostic prostate core biopsies	X																	
Patient-reported outcome measures (PROM)		X		X	X	X	X	X	X	X	X	X	X	X	X	X	X	Every 3 months
Randomisation			X															
Inform treatment choice (standard-of-care or experimental)				X														
Start ADT	X																	
Start Combination Therapy				X														
Response evaluation					X	X	X	X	X	X	X	X	X	X	X	X	X	Every 3 months
Drug compliance					X	X	X	X	X	X	X	X	X	X	X	X	X	Every 3 months
AE assessment					X	X	X	X	X	X	X	X	X	X	X	X	X	Every 3 months

Figure 4 - Schedule of activities in mHSPC. *(*) Visit 3 will take place when results of the biomarker signature analysis and new baseline assessment is available. ¶ denotes that upon treatment start (0M) all imaging and routine blood analyses need to be performed 1 week prior to scheduled follow-up study visit, ensuring all information is available for treatment response evaluation at study data event visit.*

7.3.3 Patients entering the mCRPC platform from the ProBio trial

7.3.3.1 Visit 1 (Screening)

The purpose of visit 1 is to select patients that are eligible for the study, obtain informed consent, draw blood for routine clinical laboratory analysis, genomic analysis, biobanking and record patient information. These eligible patients may enter the mCRPC platform from ProBio directly when starting 1st line systemic therapy (regardless of prior disease history), or may enter upon reaching mCRPC after being treated in the mHSPC phase from the ProBio trial. Thus, for the latter population a new ICF procedure is not needed, given that the patient already provided informed consent at ProBio trial entry in the mHSPC setting of the study.

The Investigator will explain to the patient the nature and aim of the study, its procedures, possible side effects, requirements and restrictions. The patient will sign the written informed consent before any study related procedures are performed.

<u>The following procedures will be performed during visit 1:</u>

- Obtain signed written informed consent for participation in the study if patients are directly entering ProBio in the mCRPC setting (i.e. patients who were treated for their mHSPC disease outside of the ProBio study).
- Check that the patients fulfil all inclusion criteria and none of the exclusion criteria
- Record results from latest routine clinical laboratory analysis
  - Routine blood analysis results taken within 2 weeks of inclusion may be used, if older a new blood collection for routine analysis needs to be performed.
- Collect 2 x 10 ml blood for prospective ctDNA analyses in in preservative tubes such as Streck cell-free DNA BCT tubes and 3 x 10 ml blood for exploratory objectives in either in preservative tubes such as Streck cell-free DNA BCT, EDTA tubes or selected tubes tailored for the exploratory objectives. Blood will be stored in regional biobanks or participating sites may choose to ship blood for biobanking to the country’s centralised Biobank (i.e. University Hospital or University from the national PI).
- Collect 25-50 mL first-catch urine in designated collection cups for exploratory objectives. Urine will be stored in regional biobanks or participating sites may choose to ship urine for biobanking at the country’s centralised Biobank (i.e. University Hospital or University from the national PI).
- Complete SMART-TRIAL eCRF Inclusion & Baseline Assessment Data events, recording among others:
  - Patient demographic information
  - ECOG status
  - Patient medical and surgical history
  - Patient prior and concomitant medication
  - Results from latest CT/MR scans (thorax and abdomen) and bone scintigraphy.
    - Radiology taken within 6 weeks of inclusion may be used, if older a new scan needs to be taken.
- Record Patient Reported Outcome (PRO) measures:
  - EQ-5D-5L
  - EORTC QLQ-C30
  - BPI-SF

7.3.3.2 Randomisation

Patients who fulfil all inclusion criteria, none of the exclusion criteria, and where all data is available for randomisation (ctDNA analysis completed, results from CT/MR scans (thorax and abdomen) and bone scintigraphy available, PSA-value, etc.) will be randomised to either standard of care or one of the experimental arms.

7.3.3.3 Visit 2 (treatment start, time = month 0 ± 1 week)

The treatment period begins as soon as possible after randomisation.

The following procedures will be performed during the visit 2:

- Inform patient on treatment choice
  - In case of randomisation to a non-approved therapy for prostate cancer (i.e. non SOC), a Drug-Specific patient information document and ICF will be provided to the patient
- Record patient concomitant medication
- Collect blood for routine clinical laboratory analysis
- Collect 2 x 10 ml blood for future ctDNA analyses in preservative tubes such as Streck cell-free DNA BCT tubes and 3 x 10 ml blood for exploratory objectives in either preservative tubes such as Streck cell-free DNA BCT, EDTA tubes or selected tubes tailored for the exploratory objectives. Blood will be stored in regional biobanks or participating sites may choose to ship blood for biobanking to the country's centralised Biobank (i.e. University Hospital or University from the national PI).
- Collect 25-50 mL first-catch urine in designated collection cups for exploratory objectives. Urine will be stored in regional biobanks or participating sites may choose to ship urine for biobanking at the country's centralised Biobank (i.e. University Hospital or University from the national PI).
- Complete SMART-TRIAL eCRF Month 0 (0M) Data event
- Record patient Patient Reported Outcome (PRO) measures:
  - EQ-5D-5L
  - EORTC QLQ-C30
  - BPI-SF
- Start new systemic treatment for mCRPC
- Complete SMART-TRIAL eCRF Data event

7.3.3.4 Visit 3 (time = month 1 ± 1 week)

Data collection for exploratory analyses.

The following procedures will be performed during the visit 3:

- Collect blood for routine clinical laboratory analysis
- Collect 2 x 10 ml blood for future ctDNA analyses in preservative tubes such as Streck cell-free DNA BCT tubes and 3 x 10 ml blood for exploratory objectives in either in preservative tubes such as Streck cell-free DNA BCT, EDTA tubes or selected tubes tailored for the exploratory objectives. Blood will be stored in regional biobanks or participating sites may choose to ship blood for biobanking to the country's centralised Biobank (i.e. University Hospital or University from the national PI).

- Collect 25-50 mL first-catch urine in designated collection cups for exploratory objectives. Urine will be stored in regional biobanks or participating sites may choose to ship urine for biobanking at the country's centralised Biobank (i.e. University Hospital or University from the national PI).
- Record patient concomitant medication
- Record patient Patient Reported Outcome (PRO) measures:
    - EQ-5D-5L
    - EORTC QLQ-C30
    - BPI-SF
- Assess patient AEs
- Check patient compliance to medications, by asking the patient
- Response evaluation.
    - Conduct and record biochemical/clinical response evaluation
- Complete SMART-TRIAL eCRF Data event

7.3.3.5 Visit [4, 5, 6, 7, etc.] (time = month 2, 4, 6, 9, etc. ± 3 weeks)

Response evaluation. During the first 24 weeks of treatment start, the patient will be assessed by treating physician every 8 weeks (i.e. month 2, 4, and 6). After 24 weeks, the patient will be assessed by treating physician every 3 months (i.e. month 9, 12, etc.) until progressive disease, i.e. time to no longer clinical benefit.

<u>The following procedures will be performed 1 week before every scheduled data event visits</u>:

- Perform conventional imaging
- Collect blood for routine clinical laboratory analysis

<u>The following procedures will be performed during the visit:</u>

- Collect 2 x 10 ml blood for future ctDNA analysis in preservative tubes such as Streck cell-free DNA BCT tubes and 3 x 10 ml blood for exploratory objectives in either preservative tubes such as Streck cell-free DNA BCT, EDTA tubes or selected tubes tailored for the exploratory objectives. Blood will be stored in regional biobanks or participating sites may choose to ship blood for biobanking to the country's centralised Biobank (i.e. University Hospital or University from the national PI).
- Collect 25-50 mL first-catch urine in designated collection cups for exploratory objectives. Urine will be stored in regional biobanks or participating sites may choose to ship urine for biobanking at the country's centralised Biobank (i.e. University Hospital or University from the national PI).
- Record patient concomitant medication
- Record Patient Reported Outcome (PRO) measures:
    - EQ-5D-5L
    - EORTC QLQ-C30
    - BPI-SF
- Assess patient AEs
- Check patient compliance to medications
- Response evaluation.
    - Conduct and record response evaluation based on clinical benefit (physician assessment), PSA and radiology (CT/bone scintigraphy)
    - Record response evaluation as Progressive disease, Stable Disease or Response. If progressive disease is present, the study drug is stopped, and evaluation is done

to decide if the patient can be re-randomised. If stable disease or treatment response is present, the treatment continues on study drug.

- Complete SMART-TRIAL eCRF Data event

Upon progressive disease and two randomisation cycles within ProBio, the patient will be followed using registers and/or 6-monthly follow-up inquiries towards the treating physician in terms of latest treatment status (if still on study therapy after month 36) and survival status (i.e. deceased/alive).

7.3.4 Schedule of activities in mCRPC

Activities	Screening	Randomisation	Follow up during Treatment Period till no longer clinically benefiting (PCWG3 guidelines)															
**Patient visits**	1		2*	3	4	5	6	7	8	9	10	11	12	13	14	15	16	
**Time from treatment start (months)**	**-0.5**		**0**	**1**	**2**	**4**	**6**	**9**	**12**	**15**	**18**	**21**	**24**	**27**	**30**	**33**	**36**	**Every 3 months**
**Vist windows (weeks)**	0		±1	±1	±1	±1	±3	±3	±3	±3	±3	±3	±3	±3	±3	±3	±3	±3
**Inclusion/exclusion criteria**	X																	
**Informed consent**	X																	
**Medical and disease history**	X																	
**Conventional imaging (Bone scan/CT or MRI)** ¶	X				X	X	X	X	X	X	X	X	X	X	X	X	X	**Every 3 months**
**Routine blood-based biomarkers (e.g., CBC, PSA, LDH, ALP, etc.)** ¶	X		X	X	X	X	X	X	X	X	X	X	X	X	X	X	X	**Every 3 months**
**Blood/urine liquid biopsy**	X		X	X	X	X	X	X	X	X	X	X	X	X	X	X	X	**Every 3 months**
**Patient-reported outcome measures (PROM)**	X		X	X	X	X	X	X	X	X	X	X	X	X	X	X	X	**Every 3 months**
**Randomisation**		X																
**Inform treatment choice (standard-of-care or experimental)**			X															
**Start systemic Therapy**			X															
**Response evaluation**				X	X	X	X	X	X	X	X	X	X	X	X	X	X	**Every 3 months**
**Drug compliance**				X	X	X	X	X	X	X	X	X	X	X	X	X	X	**Every 3 months**
**AE assessment**				X	X	X	X	X	X	X	X	X	X	X	X	X	X	**Every 3 months**

Figure 5 - Schedule activities in mCRPC. *(*) Visit 2 will take place when results of the biomarker signature analysis and baseline assessment is available. ¶ denotes that upon treatment start (0M) all imaging and routine blood analyses need to be performed 1 week prior to scheduled follow-up study visit, ensuring all information is available for treatment response evaluation at study data event visit.*

8. PHARMACOVIGILANCE

A detailed description in terms of treatment-specific pharmacovigilance, adverse events of special interest and active monitoring are described in the subprotocols for approved and investigational agents for prostate cancer. In general the definitions and procedures described below are applicable for all ProBio treatment arms. For niraparib plus abiraterone acetate plus prednisone there are also special situations, AE of special interest and product quality measures for which specific procedures are described in *'SUB-PROTOCOL: NIRAPARIB PLUS ABIRATERONE ACETATE PLUS PREDNISONE'*.

8.1 Safety assessments

This study has been designated as an interventional study with investigational medicinal products (IMP). As such, the safety assessment will include evaluation of AEs.

8.2 Adverse events

The evaluation period for safety will start at the time a signed and dated informed consent form is obtained to at least 30 days after the last dose of study treatment. Adverse events will be reported by the patient for the duration of the study. Adverse events including laboratory adverse events will be graded and summarized according to the Common Terminology Criteria for Adverse Events (CTCAE) v5.0.

Any clinically significant abnormalities persisting at the end of the study or during early withdrawal will be followed by the investigator until resolution or until a clinically stable endpoint is reached.

Any adverse event not listed in the CTCAE will be graded as follows:
Grade 1, Mild: Awareness of symptoms that are easily tolerated, causing minimal discomfort and not interfering with everyday activities.
Grade 2, Moderate: Sufficient discomfort is present to cause interference with normal activity.
Grade 3, Severe: Extreme distress, causing significant impairment of functioning or incapacitation. Prevents normal everyday activities.
Grade 4, Life-threatening: Urgent intervention indicated.
Grade 5, Death.

8.3 Definitions

Adverse Event:

Any untoward medical event in a patient administered a pharmaceutical product which does not necessarily have to have a causal relationship with the treatment. An adverse event can be any unfavorable and unintended sign (including abnormal finding or lack of expected pharmacological action), symptom, or disease temporarily associated with the use of a medicinal (investigational or non-investigational) product, whether or not related to that (investigational or non-investigational) product.
This includes any occurrence that is new in onset or aggravated in severity from the baseline condition, or abnormal results of any diagnostic procedures, including laboratory test abnormalities.

Adverse Reaction:

An adverse reaction is defined as all untoward and unintended responses to an investigational medicinal product related to any dose administered.

Serious Adverse Event:

Any adverse event occurring that results in any of the following outcomes:

- death;
- a life-threatening adverse drug experience;
- in-patient hospitalization or prolongation of existing hospitalization;
- persistent or significant disability/incapacity;
- congenital anomaly/birth defect;
- a suspected transmission of any infectious agent via administration of a medicinal product
- is medically significant*

*Any untoward medical occurrence that is considered medically significant. Medical and scientific judgment should be exercised in deciding whether expedited reporting is appropriate in other situations, such as important medical events that may not result in death, be life-threatening or require hospitalization but may be considered a serious adverse drug experience when, based on appropriate medical judgement, that may jeopardize the patient or subject and may require medical or surgical intervention to prevent one of the other outcomes listed in the bulleted list above. Examples of such medical events include allergic bronchospasm requiring intensive treatment in an emergency room or at home, blood dyscrasias or convulsions that do not result in hospitalization, or development of drug dependency or drug abuse or malignancy

Suspected Unexpected Serious Adverse Reaction (SUSAR)

A SUSAR is a **s**uspected **u**nexpected **s**erious **a**dverse **r**eaction, potentially causally related to the investigational medicinal product, and will be reported to the appropriate Independent Ethics Committee (IEC) and Competent Authority (CA).

Unlisted (Unexpected) Adverse Reaction/Reference Safety Information

An adverse reaction is considered unlisted when the nature or severity is not consistent with the applicable product reference safety information. For an investigational product, the expectedness of an adverse reaction will be determined by whether or not it is listed in the SmPC.

Hospitalization:

For reports of hospitalization, it is the sign, symptom or diagnosis which led to hospitalization that is the serious event for which details must be provided. Any event requiring hospitalization or prolongation of hospitalization that occurs during the study must be reported as a serious adverse event, except hospitalizations for the following:

- Hospitalizations not intended to treat an acute illness or adverse event (e.g., social reasons such as pending placement in long-term care facility)
- Surgery or procedure planned before entry into the study. [Note: Hospitalizations that were planned before the signing of ICF and where the underlying condition for which the hospitalization was planned has not worsened will not be considered serious adverse events. Any adverse event that results in a prolongation of the originally planned hospitalization is to be reported as a new serious adverse event

8.4 Eliciting and recording of AEs

The method for collecting and recording AEs will be spontaneous reporting and open question e.g. "How have you felt since previous visit" during the treatment and follow-up period.
All AEs, serious and non-serious, and pre-existing conditions, should be recorded in the eCRFs. If no AE has occurred during the trial period, this should also be recorded.
The following evaluations are to be done by the Investigator in connection with the AE:

Description of the AE
- If possible, the Investigator uses the diagnosis for description of the event. If not the diagnosis is decided, report the primary sign or symptom as the AE or SAE term

Seriousness
- Yes or No, see definition of a SAE for seriousness, as of above.

Duration of the AE (start date- end date)

Action taken due to the AE
- None
- IMP stopped
- Dosage changed

Causality rating with investigational medicinal product

Not related: Indicates that the AE is definitely not related to the study drug.

Related:
- The AE could have been produced by the patient's clinical status or the medicinal product.
- The AE follows a reasonable temporal sequence from the time of administration of the medicinal product, and cannot be reasonably explained by the known characteristics of the patient's clinical status.

Outcome of the AE
- Recovered/Resolved
- Recovering/Resolving
- Not recovered/Not resolved
- Recovered/Resolved with sequelae
- Fatal
- Unknown

8.5 Pre-existing condition

Events available at the first administration of IMP are part of baseline information (pre-existing conditions). These events will be considered as AE, only if they worsen after first administration of IMP. Elective procedures or surgery planned before first administration of IMP is not considered as an AE/SAE.

8.6 Reporting of SAEs

All SAEs must be reported by the Investigator using the SMART-TRIAL AE module within 24 hours from the Investigator's knowledge of the event to the Study Sponsor or other members of the staff at the Sponsor, regardless of the time that may have elapsed from the time the event occurred to when the Investigator first learns of it. For the niraparib plus abiraterone acetate plus prednisone therapy arm there are additional reporting procedures, as described in *'SUB-PROTOCOL: NIRAPARIB PLUS ABIRATERONE ACETATE PLUS PREDNISONE'*.

The initial SMART-TRIAL SAE report should contain as a minimum the following information:

- patient identification
- treatment specification
- diagnosis or symptoms
- time specification for the SAE
- name of the original reporter

A follow up SAE report form must also be completed, signed by the Investigator and submitted to the national Sponsor no later than five working days after the initial information was received. Dependent on the country of enrollment, different procedures may be employed, as described in '*Country-specific AE requirements and procedures*'.

Apart from the information above, this follow-up report should also contain the following information:

- assessment of intensity
- assessment of causality

No distinction should be made between the tested investigational product and the reference/comparator product regarding reporting of SAEs. Only SAEs that are both unexpected and assessed as related to IMP(s), i.e. SUSARs, are subject to expedited reporting to the appropriate IEC and CA, by the Sponsor.

8.7 Follow-up of unresolved AEs

If a patient is withdrawn due to an AE, or if an AE persists at the end of the trial treatment period, this should be followed up until the condition has ceased or until the patient is under professional medical care and a potential causality between IMP and the AE has been penetrated. An outcome assessment should be performed when an AE persists.

8.8 Coding of AEs

All AEs will be coded according to Medical Dictionary for Regulatory Activities (MedDRA). The coding will be performed by the Sponsor. AEs will be classified into standardized medical terminology from the verbatim description (Investigator term) using MedDRA. AEs will be presented by preferred term nested within System Organ Class. Verbatim description and all MedDRA level terms, including the lower level terms, for all AEs will be contained in the data listings of the clinical study report for this protocol.

8.9 Country-specific safety reporting procedures and contact details

In general, the definitions and procedures described below are applicable for all ProBio treatment arms. For niraparib plus abiraterone acetate plus prednisone there are additional situations that

require reporting to the Sponsor, as described in *'SUB-PROTOCOL: NIRAPARIB PLUS ABIRATERONE ACETATE PLUS PREDNISONE'*.

8.9.1 AE

In accordance with the rules of the protocol, investigators shall report to sponsor any adverse events or abnormal laboratory readings which are regarded in the protocol as critical to the assessment of the safety of the medicinal product. The evaluation period for safety will start at the time a signed and dated informed consent form is obtained to at least 30 days after the last dose of study treatment. Adverse events will be reported for the duration of the study. Adverse events including laboratory adverse events will be graded and summarized according to the CTCAE criteria. Any clinically significant abnormalities persisting at the end of the study or during early withdrawal will be followed by the investigator until resolution or until a clinically stable endpoint is reached. All suspected new risks and relevant new aspects of known adverse reactions that require safety-related measures, i.e. so called safety signals, must be reported to the Sponsor within 24 hours. The Sponsor-Investigator must report the safety signals within 7 days to the Ethics Committee via an appointed clinical trials unit, and (if required depending on national legislation) to the competent authorities, using the country-specific contact details (Table 4). The Sponsor-Investigator must immediately inform all participating Investigators about all safety signals. The other in the trial involved Ethics Committees will be informed about safety signals via the Sponsor-Investigator.

8.9.2 SAE

All serious adverse events (initial and follow up information) occurring during this study (and occurring within a period of 12 weeks following the last intake of study medication) must be reported by the local Principal Investigator within 24 hours after becoming aware of the SAE using the country-specific contact details (Table 4). It is the responsibility of the local PI to report the local SAE's to the local EC. This reporting is done by using the appropriate SAE form. The Sponsor-Investigator will re-evaluate the SAE and return the form to the site. SAEs resulting in death are reported to the local Ethics Committee, which may be performed by an appointed clinical trial unit, within 7 days. Subsequently, the other in the trial involved Ethics Committees will receive the SAEs resulting in death via the Sponsor-Investigator within 7 days. An annual safety report is submitted once a year during the clinical trial to the competent authorities by the Coordinating Investigator/Sponsor regarding the safety of the subjects and a list of all suspected serious side effects that have arisen during the year.

8.9.3 SUSAR

In case the Coordinating Investigator/Sponsor, in consultation with the nationally appointed Clinical Trial Unit, decides that the SAE is a SUSAR (Suspected Unexpected Serious Adverse Reaction), the appointed Clinical Trial Unit will report the SUSAR to the competent authorities within the timelines as defined in national legislation, using the country-specific contact details (Table 4). The Coordinating Investigator/Sponsor reports the SUSAR to all local PI's. In case of a life-threatening SUSAR the entire reporting process must be completed within 7 calendar days. In case of a non life-threatening SUSAR the reporting process must be completed within 15 calendar days. In case the (SU)SAR occurs at a local participating site, the local PI or study team should also contact the local Ethics Committee and the Coordinating Investigator, depending on the national legislation.

Table 4 - General (A) and country-specific (A & B) contact details for pharmacovigilance reporting

A

**General Contact information**	**Sweden**	**Belgium**	**Norway**	**Switzerland**
Coordinating Investigator	henrik.gronberg@ki.se	piet.ost@ugent.be	jan.oldenburg@medisin.uio.no	Ashkan@Mortezavi.com
	+46 (0)70-341 13 56	+32 (0)93322411	0047 95094528	
Clinical trial unit	Karolinska Trial Alliance	HIRUZ Clinical Trial Unit (CTU) of the University Hospital Ghent	Karolinska Trial Alliance (SUSAR reporting)	
	kta.karolinska@sll.se	hiruz.ctu@uzgent.be	kta.karolinska@sll.se	
Project Manager Sponsor	ProBioPV@meb.ki.se			
	+46 (0)8-524 825 76 | +46 (0)70-263 52 97			
Trial Sponsor representative	henrik.gronberg@ki.se			
	+46 (0)70-341 13 56			

B

	**Sweden**	**Belgium**	**Norway**	**Switzerland**
**(S)AE reporting**	Project manager of the Sponsor	HIRUZ CTU of the University Hospital Ghent<br>The local/central ethics committee<br>The National Coordinating Investigator<br>Project manager of the Sponsor	The National Coordinating Investigator<br>Project manager of the Sponsor	National Sponsor-Investigator<br>BASEC<br>The local/central ethics committee<br>Project manager of the Sponsor
**SUSAR reporting**	Karolinska Trial Alliance (KTA)<br>Medical Products Agency (Läkemedelsverket)<br>Project manager of the Sponsor	HIRUZ CTU of the University Hospital Ghent<br>The local/central ethics committee<br>Federal Agency for Medicines and Health Products<br>The National Coordinating Investigator	Karolinska Trial Alliance (KTA)<br>Medical Products Agency (SLV)<br>Project manager of the Sponsor	The local/central ethics committee<br>BASEC<br>Swissmedic<br>Project manager of the Sponsor

		Project manager of the Sponsor		

9. STATISTICAL CONSIDERATIONS

The goal of ProBio is to evaluate the clinical effectiveness of treatment class selection based on a biomarker signature derived from circulating tumor DNA (ctDNA) or tumor tissue DNA by improving Progression Free Survival (PFS) compared to standard-of-care (SOC) in patients with metastatic hormone-sensitive and castration-resistant prostate cancer. The goal is to early identify in which biomarker signature a therapy class is superior to SOC. The level to which this goal is reached will be evaluated on three different levels:

- Primary analysis: By comparing biomarker signature-therapy class combinations with respect to superiority in PFS to a common control group (SOC) (Figure 6A). By doing that, it will be possible to demonstrate the clinical utility of the selected biomarker signatures for guiding systemic therapy in mPC patients.

- Key secondary analysis 1: By evaluating the PFS distribution of the experimental arm altogether versus the control group (Figure 6B).

- Key secondary analysis 2: By comparing experimental arms against each other (efficacy analysis) within any biomarker signature across experimental arms (Figure 6C).

A detailed description of the statistical analysis plan (SAP) and data display plan (DDP) is provided in the Trial Conduct Supplement *'STATISTICAL ANALYSIS PLAN'*). The SAP and DDP will be finalized and approved before database lock of the first dropped or graduated signature-therapy class combination.

All data will be presented using descriptive statistics. Results will be presented in total, by therapy class, and across biomarker signatures. Continuous variables will be summarized using measures of central tendency and variability. Categorical variables will be summarized using absolute and relative frequencies.

Statistical models will be used to assess the primary and several of the secondary outcomes (see Statistical analysis plan summary below). In particular, for time-to-event outcomes we will use Bayesian survival models to contrast treatments within biomarker signatures. For binary outcomes, we will instead employ Bayesian logistic regression models.

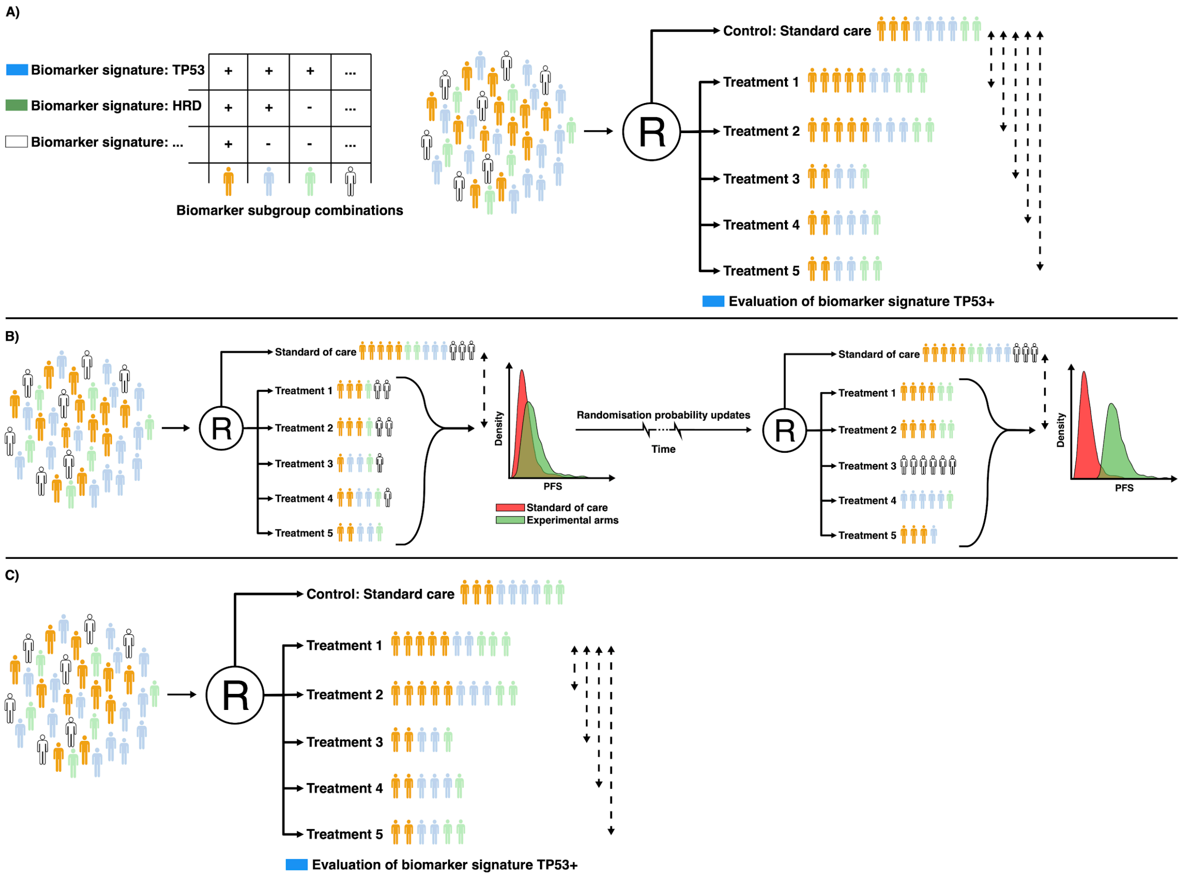

Figure 6 - Comparative analyses within ProBio. A) Comparison of biomarker signature-therapy class combinations with respect to a common control group (SOC). B) Evaluation of PFS distribution of all experimental arms versus the control group. C) Comparison of the experimental arms against each other (efficacy analysis) within any biomarker signature.

9.1 Biomarker subgroup combinations versus biomarker signatures

In ProBio, we perform genomic profiling of the cancer for each enrolled patient. *Table 5* shows the possible combinations, so called biomarker subgroup combinations, and their estimated prevalence in mCRPC based on data from (Mayrhofer et al. 2018). As seen in *Table 5*, the prevalence of some combinations are very low, in fact it is for some subgroup combinations too low for meaningful inference. Instead, we study the effect of therapy classes within sets of biomarker subgroup combinations, denoted "biomarker signatures" (*Table 6 - Biomarker signatures used in ProBio*). Each signature thus has higher prevalence than the subgroup combinations, making inference possible on the signature level.

Table 5 - Biomarker subgroup combinations used in ProBio and estimates of their prevalence.

ARA	DRD	TP53	TEfus	Percentage
-	-	-	-	32.4
-	-	-	+	6.7
-	-	+	-	17.1
-	-	+	+	11.4
-	+	-	-	6.7
-	+	-	+	4.8
-	+	+	-	1.9
-	+	+	+	1.0
+	-	-	-	4.8
+	-	-	+	3.8
+	-	+	-	1.0
+	-	+	+	3.8
+	+	-	-	2.9
+	+	-	+	1.0
+	+	+	-	1.0

Table 6 - Biomarker signatures used in ProBio. *The plus and minus signs in the table header shows the biomarker subgroup combination from Table 5. Each row of the table shows (with an 'X') which subgroup combinations that are included in different signatures. Note that some signatures are partially overlapping; in fact, the signature 'all' contains all other signatures and thus contains all patients in the trial*

signatures	----	---+	--+-	--++	-+--	-+-+	-++-	-+++	+---	+--+	+-+-	+-++	++--	++-+	+++-	prev
all	X	X	X	X	X	X	X	X	X	X	X	X	X	X	X	1.00
TP53- & AR-	X	X			X	X										0.50
TP53+			X	X			X	X			X	X			X	0.37
DRD+					X	X	X	X					X	X	X	0.19
TEfus+		X		X		X		X		X		X		X		0.32

9.2 Randomisation

Randomisation between assignment to the control arm or the biomarker driven arms will depend upon the patient's biomarker subgroup combination (i.e. the combination of the selected binary biomarkers shown in *Table 5*) and treatment history, and will therefore occur after the results from the ctDNA and/or tissue profiling (for patients with mHSPC) is obtained. Patients with mCRPC with too little tumor burden to permit ctDNA profiling will be excluded from the ProBio study and will not be randomised.

Randomisation to the control arm vs. to the biomarker driven arms will be done within each biomarker subgroup combination. Importantly, once patients are randomized to the control arm (i.e. standard-of-care), these patients will remain in the control arm throughout the course of the trial and subsequent randomisations (to permit analyses of overall survival). The randomisation algorithm is designed so that the probability of being randomised to the control group is equal to the largest of the probabilities of receiving an active treatment (this is true for any biomarker subgroup combination). The randomisation probabilities within the biomarker driven arm are proportional to the probabilities of superiority, which are constantly updated using the accumulating outcome data. Assignments to therapy in the biomarker driven arms will thus be done using the current information about the efficacy of the various regimens in the biomarker subgroup combination.

Information from previous studies may be incorporated in the randomisation at study onset if reliable such data exists. Specifically, patients with an intact androgen receptor (AR) and without TP53 mutations will initially have increased chance of being randomised to therapy class ARSi, patients with the TMPRSS2-ERG gene fusion will have increased chance of receiving taxane-based chemotherapy and HRR deficient patients will have an increase chance of receiving carboplatin or the combination therapy with niraparib plus abiraterone acetate plus prednisone. The advantages of using outcome-adaptive randomisation are that it improves on average the outcomes of study participants treated within the trial, increases the available information on treatment effects and adverse effects for the most effective treatments, shortens the evaluation time for the best therapy class – biomarker signature combinations, and allows less promising therapy class – biomarker signature combinations to fail early (thus benefiting both patients in the trial and the operating characteristics of the study).

9.3 Interim analyses and data monitoring

This is a Bayesian outcome-adaptive trial and both safety- and efficacy endpoints will be continuously evaluated. The accumulated outcome data will be analyzed monthly to evaluate treatments and to update the randomisation probabilities. If any of the stopping rules is met (graduation or futility), we will provide detailed results to the DSMB that will decide about early stopping a specific arm in the trial. Early stopping is defined by study treatment arms reaching pre-specified criteria (see Section *'9.6 Determination of sample size'*). Upon graduation of a treatment-signature combination (based on the primary analysis), we will also evaluate the key secondary analyses (1 and 2). Upon dropping a treatment-signature combination for futility, we will evaluate also the key secondary analysis 2 (but not 1).

9.4 Analytical populations

The Full Analysis Population (FAP) consists of all patients who were randomised. Patients withdrawn after randomisation and with follow-up data will also be included (according to an intention to treat analysis).

The Per Protocol Population (PP) consists of all patients in the FAP and complied with the assigned treatment, and had no significant protocol violations.

The analyses will be performed on both the FAP (primary) and PP populations.

Men randomised to the control group will receive SOC and their received treatment will be recorded. Additional comparison on the efficacy of alternative treatments vs each other will be also performed using the FAP population.

The Safety Population which consists of all patients who were included into the study and met all inclusion criteria and no exclusion criteria and had at least one exposure/dose to the study interventions.

9.5 Statistical analysis plan summary

We will use Bayesian parametric models to contrast the posterior mean PFS distributions across treatment arms within biomarker signatures. We will report the differences in the mean PFS survival as measures of therapy class effect. In addition, we will present functions of interest for survival outcomes, such as survival curves, hazard functions, and rates of time to progression. Uncertainty will be presented by reporting credible intervals. More details are available in the SAP.

The primary analysis will be on an intention to treat (ITT) basis. Drop-outs will be censored at their last observed time point (i.e. available data on the primary endpoint from men who drop out after relapse and re-randomisation will be used in the primary analysis). We will also perform per protocol (PP) secondary analyses, where drop-outs and non-compliers (e.g. men randomised to a specific therapy class but received another treatment) are removed from the analysis data. Men will be randomised multiple times, permitting us to learn about the effectiveness of the biomarker signature-therapy class combinations several times from each participant, and analyses are therefore conditioned on line of treatment. The Sequential Multiple Assignment Randomization Trials (SMART) design concept will also be used to generate hypotheses about the effectiveness of different dynamic treatment regimes, e.g. advantages with respect to progression free and overall survival of different sequences of treatments. Additional analyses will include comparisons of the response rate after 2 months of treatment in the mCRPC setting and 4 months in the mHSPC setting in all men randomised to the biomarker driven arms to men in the standard of care arm, as well as comparisons between therapies in the two randomised groups (irrespective of biomarker signature).

For the ProBio primary analysis (i.e. to assess whether treatment choice based on biomarker signatures is superior to standard of care) can be thought of as testing the effectiveness of the intervention. In the simulations, this was done by calculating the probability of superiority as the mean of the posterior superiority probabilities across the simulation repetitions for each active therapy class. Note however that the comparison against standard of care within each biomarker signature means that some men get the same therapy class on the experimental arm and the controls, since the therapy class selected by the physicians in the control arm may be the same as what the selection is based on the biomarkers. Depending on the prevalence of the treatment, the fraction of patients getting the same therapy class in the experimental and control arm can be relatively high, which dilutes the difference between the arms and therefore reduces power. It is therefore also interesting to assess the differences in PFS between different therapy classes within the same biomarker signature. This can be thought of as the efficacy of the intervention. To address the question of efficacy, we also computed the probability that each therapy class was superior as compared to others within each biomarker signature (this analysis was performed within the experimental arm to leverage the randomisation of different therapy classes).

Data from QLQ questionnaires will be standardized to scales of 0–100 for each function and symptom. Comparisons of QL scores across treatment groups will be made using regression models. Differences in toxicity events will be assessed using methods for categorical data, such as contingency tables.

AEs will be summarized by presenting the absolute and relative frequencies of AEs by treatment-signature combination. The incidence of AEs will be based on the numbers and percentages of patients with AEs. Although a MedDRA term may be reported more than once for a subject, that subject will be counted only one time in the incidence count for that MedDRA term.

9.6 Determination of sample size

Throughout the trial, decisions will continuously be made about each therapy class-biomarker signature combination. That is, it must be decided whether each therapy class-biomarker signature combination will i) "graduate" (demonstrate superiority), ii) be terminated for futility, iii) keep enrolling patients. Therapy class-biomarker signature combinations may be stopped for futility or superiority:

- Therapy class-biomarker signature combinations will be dropped from the trial for futility when probabilities of superiority drop sufficiently low (less than 30% using a minimum number

of patients assigned to the specific therapy class-biomarker signature combination in the biomarker driven arm).

- If the maximum sample size of 150 in mCRPC and 300 in mHSPC participants assigned to a therapy class-signature combination is reached, assignments to that combination will end.
- Graduation will be done on a biomarker signature combination from the trial if it has a Bayesian probability of superiority greater than a pre-specified threshold (85%) based on a minimum number of patients as well as a high probability of superiority within the biomarker subtype combinations. If a therapy class reaches a threshold for graduation, the DSMB will review the findings and make a recommendation to the principal investigators for final approval.

In a trial assessing the effectiveness of several therapy classes in the presence of multiple biomarker signatures it is important to control the false-positive rate (i.e. the error of graduating a treatment-signature combination when it in fact is not effective) due to the multiple test being performed. On the other hand, it is not advisable to over reduce the false-positive rate as it increases the type II errors and thus reduces the power of detecting effective therapy classes. As there are no closed form solutions to characterize the error and power for such complex trial designs as used in ProBio, we investigated the operating characteristics of the trial via extensive simulation studies.

The simulations were performed separately for the two phases of the trial under a wide range of scenarios, that required the specifications of several inputs. We have selected plausible values for the required inputs based either on inhouse available data (De Laere et al. 2018) or summary tables reported in scientific articles (Gravis et al. 2016; Kyriakopoulos et al. 2018; Clarke et al. 2019; Hoyle et al. 2019; James et al. 2017; Fizazi et al. 2019b; Armstrong, Szmulewitz, et al. 2019; Ian D. Davis et al. 2019; Chi et al. 2019; C. Parker et al. 2013; Fizazi et al. 2012; Ryan et al. 2015; Scher et al. 2012; Beer, Armstrong, et al. 2017). We varied the treatment effects in those scenarios in order to cover a wide range of possibilities, from pessimistic cases in which no treatment is effective in any biomarker signature, to more ideal cases where some of the treatments are effective for a subset (or all) of the biomarker signatures. For each scenario, we randomised participants in a fictitious simulated trial and recorded their outcomes.

The results of the simulation design included the average number of randomised participants within each combination of therapy class and biomarker signature, whether therapy classes-biomarker signatures graduated, and the average duration of the time in which a certain therapy class remained under investigation in a specific biomarker signature. We calculated the power as the percentage of times we graduated a therapy class for the true biomarker signature (for example graduated a therapy class for the HRD+ signature when the therapy class benefited only HRD+ patients). The false-positive rate was calculated as the percentage of times that a therapy class graduated only for a signature that did not benefit from the treatment (for example, a therapy class graduate for the TEfus+ signature but not for the HRD+ signature, when the therapy class benefited only HRD+ patients). It should be noted that these are very stringent definitions of errors, since we only count a graduation as correct if it happens for exactly the right biomarker signature-therapy class combination. It is also possible to be partially right, for example graduating a therapy class for a biomarker signature which is a sub- or superset of the exact right signature. Partially correct graduations and futility stops are counted as errors in the simulations, in order to get as conservative an estimation of type 1 and 2 error rates as possible.

9.6.1 Simulations and sample size calculation for the mHSPC platform of the ProBio trial

We simulated the PFS times from a Weibull survival model with a median PFS time of 32 months. More effective treatment classes were instead generated from a Weibull survival model with median PFS

times ranging from 50 to 60 months, which approximately corresponds to a hazard ratio in the range of 0.55 and 0.45, respectively. The control group representing standard-of-care is based on a mix of three available treatment classes: 35% ADT+Hormone therapies, 35% ADT+Taxel therapy, and 30% other therapies. We set 300 patients to have maximum sample size within each biomarker signature-treatment class combination, while assuming an enrollment rate of 30 patients/month. The maximum enrolling and follow-up time was 5 years.

The average number of enrolled patients in effective biomarker signature-treatment class combinations varied between 80 and 301 depending on the simulation scenario. For example, an average of 106 patients were enrolled in the simulation scenario where ADT+Niraparib had a median PFS time of 60 months in the HRD+ signature, whose prevalence has been estimated around 30%. Treatment classes evaluated in more prevalent signatures should require generally less patients for graduation. On the other hand, as we considered treatment also present in the control group, this diluted the comparison and led to a higher average number of enrolled participants as high as 224 for ADT+Hormone therapies in the TP53- signature or 146 for ADT+Taxel therapies in the TEfus+ signature. The average time in which effective signature-treatment class combinations remained in the trial range from 41 (e.g. ADT+Niraparib in the HRD+ signature) to 46 months (e.g. ADT+Taxel therapies in the TEfus+ signature) depending on the assumed simulation scenario.

We have designed the simulation scenarios to control the false-positive rate for single treatment classes to be lower than 15%, and around than 35% when considering treatment classes altogether in the multiple biomarker signatures. The overall error rate ranges from 30.6% in the more pessimistic scenario to 7.4% in more ideal scenarios, while treatment class-specific error rates were in the range of 1%-9%. We have adopted a stringent definition of error, i.e. graduating a treatment class-signature that was not effective (see above). On the other hand, as biomarker signatures overlap, some effective biomarker subgroup combinations still contribute also to ineffective biomarker signatures. The overall power, instead, ranged from 71% to 84% depending on which treatment class, the assumed PFS time distribution, and the prevalence of the biomarker signature. The main focus of ProBio is the effectiveness of treatment decisions based on the biomarker signature rather than the efficacy of (many already approved) treatment class-signature combinations. Therefore, the figures for power have to be related to the scope of the trial. On the other hand, we can also compare the efficacy of alternative treatment class-signature combinations. For example, in the scenario where several treatment classes are more effective in different signatures, we were able to contrast the PFS time distribution across the treatment class-signature combinations within the experimental arm. Thus, ProBio provides two levels on which to learn about which signature-treatment class combinations that results in increased PFS: the effectiveness level and the efficacy level. Even if a signature-treatment class combination fails to demonstrate superiority compared to standard of care (effectiveness) due to the fact that the controls to a large extent get the same treatment class (because physicians select the correct treatment for the patients without knowledge of the biomarkers), there is still the possibility to demonstrate that a specific treatment class is superior to other treatment classes for a given signature (efficacy).

9.6.2 Simulations and sample size calculation for the mCRPC platform of the ProBio trial

In each scenario we simulated the PFS time from a Weibull survival model with a mean PFS time 6.3 months. A more effective therapy class was instead generated from a Weibull survival model with a higher mean PFS time, ranging from 16.3 to 19.1 months, which corresponds to a hazard ratio of approximately 0.3 to 0.4 for the active treatment as compared to the control group. Based on data from current clinical practice, the control group is a mix of the available treatment classes. In particular, patients in the control group receive Hormone therapies 40% of the times, Taxel therapies 35% of the times, and other therapies 25% of the times. We fixed a maximum of 150 patients enrolled in each combination of treatment class-biomarker signature, and that the accrual rate was 30 patients/month.

The maximum enrolling and follow-up time was 3 years. In terms of sample sizes in the simulations, the average number of participants in a treatment class-biomarker signature combination ranged from 49 to 91 depending on the scenario assumed. Specifically, during the initial start of the ProBio study (evaluation of drugs already approved for CRPC that have effect against large patient groups where there is a good prior knowledge about what biomarker signature they are active against), based on the simulations we estimate that 35 patients need to be recruited to graduate the ARSi arms. These calculations assume a prevalence of 40% for CRPC patients with intact androgen receptor and a doubling of the PFS in the biomarker driven arms. The average time in which effective biomarker signature-treatment class combinations remained in the trial range from 11 (e.g. Hormones therapies in the TP53- & AR- signature) to 25 months (e.g. Niraparib in the HDR+ signature) depending on the assumed simulation scenario.

Similar to the setting of mHSPC patients, we have designed the simulation for controlling the false error rate to be in the order of 35% when considering all the treatment classes together, and less than 15% for individual treatment class. The overall error rate ranged from the 37% (most pessimistic setting) to 8%. We have also adopted here the stricter definition of error, where effective subgroup combinations also contribute to ineffective biomarker signatures. Figures for power calculations resemble those from the mHSPC setting, ranging from 65% to 83% depending on the graduating treatment class, the assumed PFS time distribution, and the prevalence of the biomarker signature.

9.6.3 Simulations web interface

We have performed an extensive range of simulation scenarios and it is impractical to summarize them all in a document. We have therefore implemented a web interface to explore the possible evolution of the study in one instance of the simulations for each of the considered scenarios. The interactive webpage is available at the following link: http://alecri.github.io/downloads/sim_probio_v5/simulation_report.html . We present the main results separately for the two study populations in the platform trials.

9.7 Confirmatory trial

The false-positive rate is the major inferential stumbling block in a study investigating the benefits of many therapy classes in the presence of many biomarker profiles, and false-positives must thus be controlled. Given the multiplicity of the available therapy class-signature combinations, it is important to control the type I error rate in the trial. Therefore, therapy classes that are performing well in one of the biomarker signatures and meet the requirements for graduation will be included in a seamless confirmatory trial. The confirmatory trial will be conducted within the ProBio platform. The graduated therapy class will no longer be available in the experimental arm for the associated biomarker signature of graduation. The patients who would have been randomised to the graduated therapy class biomarker signature combination will be instead assigned to the control group. A proportion of the control group will still work as comparator for the remaining therapy classes under investigation while the other part will constitute the side trial. A fixed proportion of patients will be randomised (1:1 ratio) to either the graduated therapy class or current clinical practice, with a maximum of 100 patients in total. The accumulating data in the validation trial will be assessed at specific time points to allow for early stopping using an O'Brien-Fleming alpha spending function. We will implement discrete sequential boundaries in order to maintain the type I error at desirable levels. The main goal of the ProBio platform is to quickly generate highly plausible hypotheses, which will then be confirmed or disproved in the validation trial. We may therefore allow a less stringent type I error for the platform design; the type I error in ProBio is around 10% but can reach 35% under unlikely, but possible, circumstances. Therefore, we will design the validation trial with an α-level of 15%, so that the maximum overall type I error is given by the product of the two (due to independence between the cohorts), which will be around 5.25% (thus roughly corresponding to an α in the range of 0.05, typically

used in frequentist trials). More detailed characteristics on the confirmatory trial will be provided in the statistical analysis plan.

10. ETHICS

10.1 Ethical and Regulatory review

Necessary approvals of the Trial Protocol, the Patient Information and Informed Consent Form (ICF) must be obtained before enrolment of any patient into the trial. Furthermore, it is the responsibility of the Sponsor to keep the applicable Independent Ethics Committee (IEC) informed of any Suspected Unexpected Serious Adverse Reactions (SUSARs) and any substantial amendments to the protocol during the trial period. The written approval from the IEC, including a trial identification and the date of approval, will be filed at the Sponsor representative and at the trial site(s) together with a list of the IEC members, their titles or occupation, and their institutional affiliations.

10.2 Ethical conduct of the trial

The trial will be performed in accordance with the recommendations guiding physicians in biomedical research involving human patients that were adopted in 1964 by the 18th World Medical Assembly, in Helsinki, Finland, with later revisions.

10.3 Patient information and consent

It is the responsibility of the Investigator to give each patient (or the patient's legal representative), adequate verbal and written information regarding the objectives and the procedures of the trial as well as any risks or inconvenience involved before including the patient in the trial. The patient (or the patient's legal representative) should be informed that by signing the ICF he/she authorizes monitor(s), auditor(s), the IEC and the Regulatory Authorities (RA) to have direct access to the patient's medical records for verification of clinical trial procedures. The patient must be informed about the right to withdraw from the trial at any time. The patient should be allowed sufficient time for consideration of the proposal. It is the responsibility of the Investigator to obtain signed informed consent from all patients before including them in the trial. The ICF must be signed and dated before any trial-specific procedures are performed, including screening procedures. The signed ICFs must be filed by the Investigator for possible future audits and/or inspections. The final version of the Patient Information and ICF is submitted to the Ethics Committee(s) and concerned RA and must not be changed without permission from the Sponsor and the local IEC. Within the ProBio trial, one single patient information document and ICF is used, which may be either presented to the patient during inclusion in the mHSPC or mCRPC phase of ProBio (dependent in which phase of the disease the patient is being enrolled). Additionally, in case of randomisation to a non-approved therapy for prostate cancer (i.e. non SOC), a Drug-Specific patient information document will be provided to the patient.

10.4 Patient data protection

The Investigator must file a patient identification list which includes sufficient information to link records, i.e. the Case Report Form (CRF) and clinical records. This list should be preserved for possible future inspections/audits but should not be made available to the Sponsor except for monitoring or auditing purposes. The patients will be informed that the data will be stored and analyzed by

computer, that national regulations for the handling of computerized data will be followed and that identification of individual patient data will only be possible for the Investigator. The patients will be informed about the possibility of inspections/audits of relevant parts of the clinical records by representatives of the Sponsor, and/or RA. Authorization to direct access to the patient's clinical records, as described above, is given by signing the ICF.

10.5 Ethical considerations in terms of detected genomic alterations

10.5.1 Blinding of biomarker signatures

Patients and treating physicians will be blinded to biomarker signature, both in the experimental- and control arms, but not to study drugs. One exception is if the genomic analysis identifies a germline mutation in high penetrance genes, that are associated with hereditary cancer syndrome, encompassing but not limited to *APC, BRCA1, BRCA2, CDH1, CDK4, CDKN2A, DICER1, MET, MLH1, MSH2, MSH6, PMS2, MSH3, PALB2, POLD1, POLE, PTEN, RB1* and *TP53*. This information might be important for not only the patients but also his relatives. Upon detection of any pathogenic or clinically relevant germline events, the mutational status of the gene(s) will be disclosed immediately to the treating physician, who is then responsible to initiate a genetic consult, upon discussion and consent from the patient. In case of detection of somatic alterations in the *BRCA1/2* genes, the mutational status will be disclosed upon 2nd progression in the mCRPC setting. An independent DSMB, Data and Safety Monitoring Board, will be responsible for evaluating and follow-up of the safety. If results from other ongoing studies makes it unethical to continue, the blinding might be broken.

10.5.2 MSI

Patients with high tumor mutational burden (TMB-H) and microsatellite instability (MSI+) are rare events in advanced prostate cancer with high-impact alterations in multiple potential driver genes making it difficult to identify the key driving genomic alterations. In the absence of a checkpoint inhibitor or other immunotherapy treatment arm this currently excludes inclusion of MSI+ as a biomarker signature in the current phase of the study. TMB-H/MSI+ cases will be monitored in the standard of care arm (i.e observational) and will not be used as controls for the experimental arms. An TMB-H/MSI+ status at screening will be disclosed immediately to the treating physician.

However, upon sponsoring or availability of a immunomodulator (i.e. checkpoint inhibitor) as investigational medical product (IMP) within the ProBio trial, the investigators aim to test the hypothesis that metastatic prostate cancer patients with TMB-H/MSI+/CDK12- will demonstrate robust durable responses to checkpoint inhibition, as described in detail in Trial conduct supplement *'BIOMARKER SIGNATURES'*.

10.5.3 Risk vs benefit assessment in the context of Homologous Recombination Repair Deficiency (HRD)

Because of the outcome-adaptive design, patients randomised to the experimental arms will have a higher probability to get an effective treatment. Men with somatic- or germline alterations in genes associated with HRD will experience a higher randomisation probability towards the carboplatin and/or niraparib plus abiraterone acetate plus prednisone arms. This decision was taken by the investigators due to: 1) the lack of overall survival benefit in the only phase-III clinical trial published on molecularly unselected mPC patients; 2) the strong association between durable responses to platinum-based chemotherapy or PARP inhibition and HRD in prostate cancer and cancers of other histological origins.

Patients in the experimental arm will be re-randomised after first disease progression and will have a higher probability to receive an effective treatment. The side effects of drugs with an indication for treatment of advanced prostate cancer are well known and similar in both study arms. For carboplatin, the risks and side-effects are expected to not succeed the established levels for ovarian cancer and small-cell lung cancer which are the two currently approved indications (according to the current SmPC), although carboplatin is routinely used for treatment of other cancers e.g. bladder cancer. The safety profile of any study drug is documented for the registered products, in the Summary of Product Characteristics (SmPC).

The genomic panel can identify germline mutations that are associated with a hereditary cancer syndrome, e.g. *BRCA1/2* and *MLH1, PMS2, MSH2/6*. This information might be important for not only the patients but also his relatives. Upon detection of any pathogenic or clinically relevant germline events, the mutational status of the gene(s) will be disclosed immediately to the treating physician, who is then responsible to initiate a genetic consult, upon discussion and consent from the patient. In case of detection of somatic alterations in the *BRCA1/2* genes, the mutational status will be disclosed upon progressive disease after the 2nd randomisation in the mCRPC setting.

An independent DSMB, Data and Safety Monitoring Board, will be responsible for evaluating and follow-up of the safety.

11. DATA HANDLING, RECORD KEEPING AND MONITORING

11.1 Data Management

This study will collect and report clinical trial data using the CE certified and GDPR compliant SMART-TRIAL eCRF system (Medei ApS, Denmark). Due to the complex nature of the statistical design, a randomisation engine was developed in the R statistical programming language (version 3.4). In order to perform the required calculations for decision making and to perform randomisation, all data relevant to the statistical modelling will be de-identified and flow from the SMART-TRIAL eCRF system to the randomisation engine; results will be returned to the SMART-TRIAL eCRF system. All application users will be trained to use the system and will comply with the instructions in the protocol-specific “User Manual” provided by the KI/SMART-TRIAL team as well as applicable regulatory requirements. De-identified study data will be available in the SMART-TRIAL eCRF system analysis portal for approved users, as outlined in the ProBio Data Access and Publication Guidelines. Participant eligibility will be systematically assessed at each of the participating ProBio study sites using the ‘Inclusion’ form in the SMART-TRIAL eCRF system. Sites will provide detailed information to all relevant treating physicians on the conduct of the trial to optimize physician participation.

11.2 Quality Control and Data Quality Assurance

A quality control (QC) of data will be performed to ensure that data entry and verification have been performed correctly in accordance to pre-defined instructions. The QC will be performed as described in the Data Management Plan. The QC will be performed before data is declared clean and the study database is closed.

11.2.1 Case Report Forms (eCRFs)

The Monitor(s) will review and verify the data collected in the eCRFs against the source documents during monitoring visits. The Monitor(s) will address the discrepancies found in the data and will ensure that corrections to the data are properly made and documented by the site personnel. All corrections will be documented in an audit trail.

11.2.2 Monitoring

The trial sites will be visited by an appointed Monitor per country periodically at times agreed with the Site Principal Investigator. It is the function of the Monitor to ascertain that all aspects of the protocol are compliant with and that the conduct of the trial conforms to applicable regulatory requirements and established rules for Good Clinical Practice (GCP).

11.2.3 Training of staff

All Investigators and staff carrying out observations of primary or other major efficacy variables involved in the trial should provide a curriculum vitae. The Investigator will keep a list of all personnel involved in the trial together with their function and trial related duties delegated. He/she will ensure that appropriate trial related training is given to all of these staff, and that any new information of relevance to the performance of this trial is forwarded to the staff involved.
Before inclusion of patients the Monitor will perform a trial initiation visit to inform and train relevant trial staff.

11.2.4 Audit and inspections

The trial site may be subject to quality assurance audit by the Sponsor or someone appointed for this task by the Sponsor. A regulatory authority may request to make an inspection of the trial site. The procedures of such a visit would be similar to those of a monitoring visit, and data already checked by the Monitor may be checked again. The Investigator is required to inform the Sponsor immediately of an inspection requested by a regulatory authority. The Investigator and other relevant personnel must be available during the audit/inspection and must devote sufficient time.

11.2.5 Changes in the approved Trial Protocol

Trial procedures must not be changed without the mutual agreement of the Investigator and the Sponsor. Any substantial change to the approved Final Trial Protocol will be documented in a written and numbered Protocol Amendment. Any proposed substantial change to the Final Trial Protocol must be discussed with and approved by the Sponsor before submitted to IEC and Regulatory Authority for approval, according to applicable national regulations.

12. GENERAL TRIAL MANAGEMENT

12.1 Trial time table

First patient in: Q1 2019
Last patient in: Q4 2025
Last follow-up: Q4 2029

12.2 Insurance/indemnity

Country	Insurance
Sweden	Swedish Patient Insurance and Läkemedelsförsäkringen
Belgium	GHENT UNIVERSITY HOSPITAL has taken out a Clinical Trial Insurance certificate with Allianz Global Corporate & Specialty SE Belgium branche, Uitbreidingstraat 86, 2600 Berchem, Belgium, ; Tel: +32 33 04 16 00<br><br>INSURANCE ID: BEL000862
Norway	Legemiddelansvarsforeningen (LAF) 2020 on behalf of investigator Jan Oldenburg<br><br>Reference for 2021: #9904313/1
Switzerland	To be completed upon protocol amendment

The Sponsor agrees to indemnify (legal and financial coverage) and hold the Investigator free of harm from any claim, whether based on legal principles or on generally accepted liability standards within the pharmaceutical industry, made against him by reason of personal injury, including death, to any person arising out of or connected with the performance of the trial to the extent that the injury is not caused by:

- failure by the Investigator to adhere to the terms of the Protocol
- failure by the Investigator to comply with any applicable governmental regulations;
- malpractice, negligence or willful malfeasance by the Investigator.

The Investigator agrees to notify the Sponsor whenever he/she becomes aware of a claim or action, and to co-operate with and to authorize the Sponsor to carry out sole management of such claim or action. The Sponsor does not provide the randomised study treatment and does therefore not need any product liability insurance.

12.3 Trial agreements

The Principal Investigator at the investigational site must comply with all the terms, conditions, and obligations of the Clinical Trial Agreement (CTA) for this trial.

12.4 Criteria for termination of the trial

The Sponsor reserves the right to discontinue the trial at any time but intends only to exercise this right for valid scientific or administrative reasons. After such a decision, the Investigator must call in all participating and inform the patients as well as perform relevant assessments, preferably according to the scheme for the final assessments. All delivered and unused trial products and other trial materials must be returned and all CRFs completed as far as possible. Investigator(s) will be reimbursed for reasonable expenses incurred, in the event this becomes necessary.

Reporting of results and publication policy
A clinical trial report, in compliance with ICH E3; Guideline for Industry, will be prepared by the Sponsor describing the conduct of the trial, the statistical analysis performed and the obtained results.
The data generated by this trial are considered confidential information and the property of the Sponsor. Said confidential information may be published only in collaboration with participating personnel from the Sponsor. The Sponsor reserves the right to review and comment on the proposed publication prior to being submitted and/or published.

12.5 Record retention

The Investigator must arrange for retention at the investigational site of a list of the patients and their identifying code, patient files and other trial documents. The archiving period must be adapted to regulations in force but should not be shorter than ten years after the termination of the trial and the presentation of the final report.
It is the responsibility of the Sponsor to inform the Investigator/institution as to when these documents no longer need to be retained.

12.6 Disclosure and confidentiality

All unpublished information concerning the IMP and research carried out by ProBio study group, including patent applications, manufacturing processes, basic scientific data, etc., is considered confidential and the sole property of the ProBio study group. Disclosure to third parties must be limited to those undertaking legitimate peer review of the scientific and ethical aspects of the trial and to those participating, including the recipients of drugs, so that customary medical care and informed consent can be achieved.

12.7 Emergency procedures

The Investigator is responsible for ensuring that there are procedures and expertise available to cope with medical emergencies during the trial. In case of an overdose, the patients will be taken care of according to standard of care. Emergency contact details are listed in the trial conduct supplement *‘TRIAL ADMINISTRATION, INVESTIGATORS AND SITES’*.

SUB-PROTOCOL: STANDARD-OF-CARE IMP

ProBio

Trial title:

ProBio:
An outcome-adaptive and randomised multi-arm biomarker driven study in patients with metastatic prostate cancer

Coordinating Investigator and Sponsor's representative

Henrik Grönberg
Karolinska Institutet

TABLE OF CONTENTS

TABLE OF CONTENTS 2

1. BACKGROUND 3

2. STUDY POPULATION 3

3. PACKAGING, LABELING AND STORAGE 3

4. DOSES AND TREATMENT REGIMENS 4

5. PHARMACOVIGILANCE 4

1. BACKGROUND

During ProBio, following approved SOC compounds (both in the SOC control and experimental arms) will be tested:

For mHSPC:

- AR signalling inhibitors (ARSi)
  - Abiraterone Acetate
  - Apalutamide

- Taxane-based chemotherapy
  - Docetaxel

- Other treatments upon approval from authorities and protocol amendment

For mCRPC:

- AR signalling inhibitors (ARSi)
  - Enzalutamide
  - Abiraterone Acetate

- Taxane-based chemotherapy
  - Docetaxel
  - Cabazitaxel

- Radionuclide-based therapy
  - Radium-223 (only allowed in the SOC control arm)

- Other treatments upon approval from authorities and protocol amendment

Detailed background information for these study treatments is described in the Summary of Product Characteristics (SmPC).

2. STUDY POPULATION

During ProBio, SOC compounds will be tested in male patients, aged above 18 years, with histologically confirmed prostate adenocarcinoma, initiating systemic therapy for metastatic disease, encompassing both newly diagnosed (i.e. *de novo*) metastatic hormone-sensitive prostate cancer (mHSPC) and metastatic castration-resistant prostate cancer (mCRPC):

3. PACKAGING, LABELING AND STORAGE

Enzalutamide, abiraterone, cabazitaxel, docetaxel and apalutamide are currently approved as standard-of-care (SOC) or are available via medical need programs for metastatic prostate cancer and will not be provided by the sponsor. The patients are given prescriptions by the investigators for the registered pharmaceutical drugs available with the above substances. All approved formulations and strengths of randomised treatments according to the marketing authorizations will be used in this study. Detailed conditions for the use of the study treatments and contraindications, special warnings and precautions for use are described in accordance with the marketing authorization in the Summary of Product Characteristics (SmPC) for the study treatments.

4. DOSES AND TREATMENT REGIMENS

Detailed conditions for the use of the study treatments including dose and dosages are described in accordance with the marketing authorization in the SmPC for the study treatments.

5. PHARMACOVIGILANCE

Abiraterone Acetate, Enzalutamide, Apalutamide, Docetaxel and Cabazitaxel have been approved for the treatment of metastatic prostate cancer. Known toxicities, the expectedness of an adverse event and the active monitoring of AEs of special interest will be determined by whether or not it is listed in the SmPC for the study treatment.

SUB-PROTOCOL: CARBOPLATIN

Trial title:

ProBio:
An outcome-adaptive and randomised multi-arm biomarker driven study in patients with metastatic prostate cancer

Coordinating Investigator and Sponsor's representative

Henrik Grönberg
Karolinska Institutet

TABLE OF CONTENTS

TABLE OF CONTENTS 2

1. BACKGROUND 3

1.1 Introduction 3

1.2 Association with DNA-repair deficiency 3

1.3 Clinical trials in metastatic prostate cancer 4

1.4 Retrospective studies supporting an association between DNA repair deficiency in metastatic prostate cancer and response to platinum-based chemotherapy 5

1.5 Summary carboplatin 5

2. STUDY POPULATION 6

3. PACKAGING, LABELING AND STORAGE 6

4. DOSES AND TREATMENT REGIMENS 6

4.1 Treatment regimens 6

4.2 Dose modifications and management of toxicity 6

5. PHARMACOVIGILANCE 6

1. BACKGROUND

1.1 Introduction

A failed cell-cycle experiment back in the 1960s, using platinum electrodes, led to the discovery of the cisplatin molecule and insight into its intracellular biological effect. Platinum complexes become activated in the intracellular milieu, which results in the formation of DNA adducts that interfere with replication and trigger DNA-damage response and cell death. Administration of cisplatin into preclinical models in 1968 demonstrated promising results which were followed by clinical trials in the early 70s where platinum-based chemotherapy was introduced for treating cancer (Kelland 2007). Today, platinum-based compounds still remain the therapeutic cornerstone for a multitude of cancers in various stages e.g. locally advanced and metastatic ovarian- and bladder cancer (McGuire et al. 1996; Neoadjuvant Chemotherapy plus Cystect...).

1.2 Association with DNA-repair deficiency

The inherent connection between platinum-based compound ́s mechanism of action and DNA damage was further strengthened by reports demonstrating that cancers, initiated by pathogenic germline mutations in DNA-repair genes such as *BRCA1* and *BRCA2*, demonstrated increased sensitivity to platinum-based chemotherapy. A population-based, case-control study of 1001 women in ovarian cancer revealed that 85.1% mutation carriers remained disease free after surgery and platinum chemotherapy at six months compared to 68.3% of noncarriers (Alsop et al. 2012). In a phase 3 clinical trial on 376 women with advanced triple-negative breast cancer, the objective response rate in mutations carriers was 68% for carboplatin- vs. 33% for docetaxel treatment (Tutt et al. 2018). *BRCA1* and *BRCA2* are critical for efficient repair of double strand breaks through homologous recombination. Pennington and colleges performed retrospective sequencing of *BRCA1*, *BRCA2* and additionally 11 genes in the homologous recombination pathway (*ATM, BARD1, BRIP1, CHEK1, CHEK2, FAM175A, MRE11A, NBN, PALB2, RAD51C, RAD51D*) for germline and somatic alterations in 390 ovarian carcinomas (Pennington et al. 2014). Alterations were strongly associated to platinum sensitivity after surgery (p= 0.0002) and overall survival (p= 0.0006). Median overall survival was 66, 59 and 41 months for patients with germline, somatic or no alteration in the aforementioned genes. However, other DNA-repair pathways are also associated with response to platinum. The nucleotide excision repair pathway is responsible for removing bulky DNA adducts caused by UV-light, radiation or other mutagens. In bladder cancer, exome sequencing was performed retrospectively on 50 patients with muscle-invasive carcinoma treated with neoadjuvant cisplatin-based chemotherapy followed by surgery (Van Allen et al. 2014). Somatic alterations in the *ERCC2* gene was associated with a complete pathological response to neoadjuvant cisplatin chemotherapy (9/9 cases). The association was subsequently verified in an independent cohort (Liu et al. 2016). Furthermore, Plimack et al screened retrospectively 287 cancer related genes in two cohorts with muscle-invasive bladder cancer treated with cisplatin before surgery (Plimack et al. 2015). The discovery cohort contained 34 patients and the validation contained 24 patients. The purpose was to identify biomarkers predicting a pathological response to neoadjuvant cisplatin therapy. Genomic alterations in two genes, associated with DNA repair (*ATM*, *FANCC*), together with the tumor suppressor *RB1* were associated with a response and

clinical benefit in both the discovery- (87% of responding patients carried one or more alteration and 0% of non-responders) and validation cohort (64% of responding patients carried one or more alteration and 15% of non-responders).

1.3 Clinical trials in metastatic prostate cancer

The activity of platinum-based chemotherapy in metastatic prostate cancer has been investigated in several clinical trials. In 2007, a single-arm phase II study investigated carboplatin + docetaxel in men with metastatic castrate resistant prostate cancer (mCRPC) refractory to docetaxel (Ross et al. 2008). Out of 34 enrolled patients, 18% experienced a 50% reduction in PSA. 56% experienced grade 3 leukopenia and 1 episode of febrile neutropenia. Median progression-free survival and overall survival was 3 and 12.4 months, respectively. In 2009, a phase III trial assessed the efficacy and side-effects of satraplatin (oral platinum analogue) in mCRPC patients progressing on chemotherapy. 950 patients were randomised 2:1 to oral satraplatin + prednisone vs. placebo + prednisone (Sternberg et al. 2009). There was a significant reduction in time to progression (HR 0.64; 95% CI, 0.51 to 0.79; $P < 0.001$) with a small median difference (11.1 vs 9.7). However, the difference in progression-free survival was elongated in the tail suggesting more pronounced response-difference in <40% of the study participants. Although PSA responses was more frequently observed in the satraplatin vs. placebo arm (25.4 vs. 12.4, P < 0.001), the primary endpoint of increased overall survival was not met. Treatment emerged adverse events (TEAE) occurred more frequently in the satraplatin arm (one or more TEAE: satraplatin arm 91.7%; placebo arm 82.1%. P < 0.001) where hematologic toxicities were the main dose-limiting toxicity for the satraplatin arm. Serious adverse events occurred more frequently in the satraplatin arm (8.7% vs 2.9%, p < 0.001). Grade 3-4 serious adverse events occurred in 6.2% in the satraplatin arm vs. 2.2% with placebo. In 2012, a single arm phase II study investigated paclitaxel in combination with carboplatin as salvage treatment in 38 patients with mCRPC, where 24 had received multiple chemotherapy regimens (Kentepozidis et al. 2012). Ten patients (26.3%) experienced a 50% reduction in PSA. Median progression-free survival and overall survival was 3.6 and 9.9 months, respectively. Grade 3 and 4 neutropenia was observed in three (7.9 %) and nine (23.7 %) patients, respectively. Today, there are three international studies (Clinicaltrials.gov) on the same patient population (CRPC with mutations in the DNA repair genes) that use Carboplatin as single dose in the same way we propose to do:

- Trial Evaluating the Efficacy of CARBOPLATIN in Metastatic Prostate Cancer With Gene Alterations in the Homologous Recombination Pathway (PRO-CARBO)- NCT03652493
- The BARCODE 2 Study - The Use of Genetic Profiling to Guide Prostate Cancer Treatment (BARCODE2) - NCT02955082
- Carboplatin in Castration-resistant Prostate Cancer (PRO-PLAT) - NCT02311764.

In addition, there are a number of case reports about patients well responding to carboplatin monotherapy (Cheng et al. 2016; Beltran et al. 2015; Zafeiriou et al. 2019). The natural combination with Docetaxel clearly gives much more side effects and we believe that this more toxic combination will have limited applicability.In the event that patients do not respond as anticipated to Carboplatin, there is a built- in safety mechanisms in the trial design, which means that the treatment arm will be

dropped from the trial for futility when success probabilities drop sufficiently low using a minimum of 20 patients.

1.4 Retrospective studies supporting an association between DNA repair deficiency in metastatic prostate cancer and response to platinum-based chemotherapy

As summarized here, somatic- or germline alterations in DNA-repair genes are strongly associated with increased sensitivity to platinum compounds in breast-, ovarian- and bladder cancer. It is therefore noteworthy that the fraction of men with ≥50% PSA response from the two phase-II trials (Ross et al. 2008; Kentepozidis et al. 2012) or PSA response in the phase-III trial (Sternberg et al. 2009) is close to the proportion of mCRPC patients reported to harbor DNA-repair deficiency (Robinson et al. 2015). Support for this connection has been reported in the literature. Pomeranz et al. performed a retrospective analysis of a single-institution mCRPC cohort where BRCA2 germline variants were investigated for response to carboplatin (Pomerantz et al. 2017). Eight out of 141 men carried pathogenic germline variants that were also treated with carboplatin. Six experienced >50% PSA decline within 12 weeks compared to 23/133 (17%) of non-carriers (P < .001). Cheng et al. profiled three exceptional mCRPC platinum chemotherapy responders defined as patients who experience complete or partial response longer than 6 months when the expected response to therapy is <20% (Cheng et al. 2016). All three all presented with BRCA2 biallelic inactivation. Similarly, Zafeiriou et al investigated three cases with durable responses to platinum-based chemotherapy. The first patient had mutational signatures in the tumor genome indicative of homologous recombination deficiency, the second harbored a pathogenic germline variant in BRCA2 and the third presented with a pathogenic germline variant in ATM (Zafeiriou et al. 2019). Finally, a mCRPC patient receiving palliative cisplatin-docetaxel chemotherapy was enrolled in a trial with the goal to increase the understanding how whole-exome sequencing could affect treatment selection in patients with metastatic disease (Beltran et al. 2015). The patient experienced an exceptional response without evidence of recurrent disease after two years of follow up. *FANCA* biallelic inactivation was detected in this patient together with high mutation burden and pronounced chromosomal instability indicating defective DNA-repair.

1.5 Summary carboplatin

The investigators aim to test the hypothesis that DNA-repair deficient metastatic prostate cancer patients will demonstrate robust durable responses to platinum-based chemotherapy. This hypothesis is supported by:

1. A strong association between DNA-repair deficiency and response to platinum-based chemotherapy as demonstrated by multiple clinical trials and retrospective studies from advanced cancers of different tissue origins.
2. The fraction of mCRPC patients responding in clinical trials being close to the proportion reported to harbor DNA-repair deficiency.
3. Retrospective studies that support the association between DNA-repair deficiency and response to platinum-based chemotherapy in mCRPC.

2. STUDY POPULATION

During ProBio, carboplatin therapy will be tested in male patients, aged above 18 years, with histologically confirmed prostate adenocarcinoma, initiating systemic therapy for metastatic castration-resistant prostate cancer (mCRPC).

3. PACKAGING, LABELING AND STORAGE

Carboplatin is approved for advanced ovarian cancer and small-cell lung cancer. Carboplatin will be used in commercial packaging, the hospital pharmacy will procure the commercial packages. Additional labelling will be done by the hospital pharmacy in line with regulatory requirements, and then distributed. The formulations and strength will be given according to EMA product information recommendations for ovarian cancer and small-cell lung cancer. Detailed conditions for the use of the study treatments and contraindications, special warnings and precautions for use are described in the SmPC.

4. DOSES AND TREATMENT REGIMENS

4.1 Treatment regimens

Carboplatin will be administered every 3rd week with an AUC = 4 - 5 (according to clinical judgment of the treating physician) with a dose calculated according to the Carboplatin AUC Dose calculation (Calvert formula):

Dose (mg) = Target AUC (mg/ml x min) x [GFR ml/min + 25].

Treating physicians are encouraged to schedule a minimum of 6 cycles, and if toxicity is tolerable and manageable continue carboplatin up to 9 cycles.

4.2 Dose modifications and management of toxicity

Carboplatin dose reduction instructions are described in the SmPC.

5. PHARMACOVIGILANCE

For carboplatin, the expectedness of an adverse event will be determined by whether or not it is listed in the SmPC. For the investigational therapy of carboplatin there are AEs of special interest. Adverse events of special interest are events that the ProBio trial is actively monitoring as a result of a previously identified signal (even if non-serious), as described in the literature. For Carboplatin, the adverse events of special interest are:

- Thrombocytopenia
- Neutropenia
- Anemia
- Nausea
- Kidney function test abnormalities
- Liver function test (LFT) abnormalities
- Decreased electrolyte level
- Pain
- Asthenia

SUB-PROTOCOL: NIRAPARIB PLUS ABIRATERONE ACETATE PLUS PREDNISONE

Trial title:

ProBio:
An outcome-adaptive and randomised multi-arm biomarker driven study in patients with metastatic prostate cancer

Coordinating Investigator and Sponsor's representative

Henrik Grönberg
Karolinska Institutet

TABLE OF CONTENTS

TABLE OF CONTENTS 2

1. BACKGROUND 3

1.1 Introduction 3

1.2 Clinical trials with niraparib in metastatic prostate cancer 4

1.3 Retrospective studies supporting an association between homologous recombination repair deficiency in metastatic prostate cancer and response to PARP inhibition 4

1.4 Summary niraparib plus abiraterone acetate plus prednisone 5

2. STUDY POPULATION 5

3. PACKAGING, LABELING AND STORAGE 5

4.1 Treatment regimens 6

4.2 Dose modifications and management of toxicity 7

4.3 Non-hematologic toxicities 8

4.3.1 Hepatic toxicities 8

4.3.2 Hypokalemia 11

4.3.3 Hypertension 12

4.3.4 Posterior Reversible Encephalopathy Syndrome (PRES) 13

4.4 Hematologic toxicities 14

5. PHARMACOVIGILANCE 17

5.1 Safety and Product Quality Data for the combination therapy niraparib plus abiraterone acetate 17

5.1.1 Adverse Events of Special Interest (AESI) 17

5.1.2 Special Situations 18

5.1.3 Pregnancy 18

5.1.4 Product quality complaints 18

5.2 Transmission Methods and Procedures for Reporting Safety Data and Product Quality Complaints (PQCs) to Janssen 19

5.2.1 Safety Reporting 19

5.2.2 PQC Reporting 21

5.2.3 Contact information for transmission of Safety Data and Product Quality Complaints to Study Sponsor 21

1. BACKGROUND

1.1 Introduction

There is accumulating data that men with metastatic prostate cancer and a homologous recombination repair (HRR) deficiency (HRD), e.g. *BRCA2* inactivation, may experiences better responses and outcomes when treated with a poly-adenosine diphosphate ribose polymerase (PARP) inhibitor (Mateo et al. 2019). PARP inhibition is synthetically lethal in homologous recombination repair-deficient cells. The repair of single strand breaks in the DNA is among others mediated by PARP1 and serves as a platform to recruit other DNA repair proteins, which add poly (ADP-ribose) units to the target proteins by a process called PARylation. Inhibiting PARylation by blocking the PARP1 catalytic activity results in replication fork collapse and dsDNA breaks, which in the context of homologous recombination repair deficiency remain unrepaired, leading to cell death (Sonnenblick et al. 2015). Importantly, PARP-associated DNA repair pathways are also closely connected to androgen receptor (AR) signalling. AR drives the expression of several homologous recombination factors. AR and PARP exist in a feed-forward loop, wherein AR drives the mRNA expression of PARP. In turn, PARP enzymatic activity is required for maximal AR transcriptional function (Schiewer et al. 2012). Pharmacological targeting of PARP results in diminished AR occupancy on chromatin. Thus, understanding this interplay can offer strategies for PARP inhibition beyond biallelic HRD patients.

Multiple PARP inhibitors have been developed, e.g. olaparib, rucaparib and niraparib. Niraparib (Janssen-Cilag NV Pharmaceuticals) is an orally available, highly selective poly (adenosine diphosphate [ADP]-ribose) polymerase (PARP) inhibitor, with potent activity against PARP-1 and PARP-2 DNA-repair polymerases and is approved as maintenance therapy for adult patients with recurrent epithelial ovarian, fallopian tube, or primary peritoneal cancer who are in a complete or partial response to platinum-based chemotherapy (see*: INVESTIGATOR'S BROCHURE NIRAPARIB, version number 11 and version number 11 addendum 1; and INVESTIGATOR'S BROCHURE CJNJ-67652000 (niraparib/abiraterone acetate fixed-dose combination), version number 1*). Most recently, niraparib received a Breakthrough Therapy Designation, based on data from the GALAHAD study (vide infra) (OncologyPRO n.d.). The ProBio trial will evaluate niraparib in combination with abiraterone acetate plus prednisone for subjects with newly diagnosed metastatic hormone-sensitive (mHSPC) and castration-resistant prostate cancer (mCRPC). The mHSPC population encompasses subjects who have not been treated with niraparib and/or abiraterone acetate in the hormone-sensitive setting. In the clinical setting, phase 1b (Study 64091742PCR1001) data demonstrated that the combination of niraparib with abiraterone acetate plus prednisone showed no drug-drug interactions or overlapping toxicities. Additionally, recent data demonstrated the efficacy of combining AR-targeted therapy and a PARP inhibitor in patients with metastatic prostate cancer. Patients treated with the combination of olaparib and abiraterone acetate plus prednisone had improved rPFS compared with those treated with abiraterone acetate plus prednisone alone, in both patients with and without HRD (Clarke et al. 2018).

1.2 Clinical trials with niraparib in metastatic prostate cancer

The activity of PARP inhibition using niraparib in metastatic prostate cancer is currently being investigated in several clinical trials. GALAHAD (NCT02854436) is an ongoing, multicenter, open-label phase 2 study, evaluating the efficacy and safety of niraparib monotherapy (300 mg daily) in patients with mCRPC and HRR anomalies (i.e. having biallelic alterations in BRCA1/2 (BRCA), ATM, FANCA, PALB2, CHEK2, BRIP1, or HDAC2 by plasma or tissue NGS analysis) who previously received ≥1 line of taxane-based chemotherapy and after ≥1 line of AR-targeted therapy. A prespecified interim analysis revealed objective response rate (ORR) and composite response rate (CRR, i.e. CTC conversion or ≥50% PSA decline) of 41% and 63%, respectively, in patients with bi-allelic BRCA alterations treated with niraparib (Smith et al. 2019). Radiographic progression-free survival (rPFS) and OS was 8.2 and 12.6 months, respectively. In patients with non-BRCA biallelic HRD, ORR was 9% and CRR was 17%. The two non-BRCA responders both had bi-allelic alterations in FANCA. The median rPFS and OS for the bi-allelic non-BRCA HRD patients was 5.3 and 14.0 months, respectively. Thus, GALAHAD supports that niraparib has high clinical activity in patients with bi-allelic HRD.

Niraparib is also being explored in various ongoing combination studies:

- QUEST (NCT03431350) is a phase 1b-2 study of niraparib in combination with JNJ63723283, an investigational, intravenous, monoclonal antibody against programmed cell death receptor-1 (PD-1), for the treatment of mCRPC in patients who previously received at least 4 weeks of 1-2 lines of novel AR-targeted therapy for prostate cancer.

- BEDIVERE (NCT02924766) is a phase 1b study in combination with apalutamide or abiraterone acetate plus prednisone in patients with mCRPC who may or may not have HRR anomalies who previously received ≥1 line of taxane-based chemotherapy and after ≥1 line of AR-targeted therapy.

- MAGNITUDE (NCT03748641) is a phase 3 study of niraparib in combination with abiraterone acetate plus prednisone compared to abiraterone acetate plus prednisone as first-line therapy of mCRPC in patients with or without HRR gene defects.

1.3 Retrospective studies supporting an association between homologous recombination repair deficiency in metastatic prostate cancer and response to PARP inhibition

The landmark TOPARP-A trial (phase II, NCT01682772) demonstrated an ORR in 16/49 olaparib-treated patients who no longer responded to standard treatments, of whom 12 benefitted of olaparib for more than 6 months (Mateo et al. 2015). Next generation sequencing of tumour samples identified 16 patients with somatic homozygous deletions of both *BRCA1* and *FANCA*, somatic frameshift mutations in *PALB2*, heterozygous *PALB2* deletions, and biallelic aberrations in *HDAC2*; of whom 14 responded to olaparib. The follow-up phase II TOPARP-B trial (NCT01682772) confirmed olaparib activity in HRR-deficient (i.e. germline or somatic; mono- or bi-allelic) and heavily pre-treated mCRPC with ORR of 54% and 37% in the 400 and 300 mg cohorts, respectively (Mateo et al. 2019). Highest sensitivity was observed in *BRCA1/2* aberrant tumors, but with confirmed responses in other HRD genotypes. At a median follow up of 17.6 months, median progression-free survival was 5.4 months.

The PROfound trial is the first randomised, open-label, targeted therapy Phase III trial to deliver positive results for biomarker-selected targeted treatment in patients with mCRPC (NCT02987543) (OncologyPRO n.d.). However, the control-arm was designed with a treatment sequence, known to have limited clinical activity, i.e. ARSi following progression on ARSi (Khalaf et al. 2019), which may have artificially inflated the survival difference to Olaparib. Olaparib improved radiographic progression-free survival (rPFS) compared with enzalutamide or abiraterone acetate plus prednisone in men with heavily pretreated and poor prognosis mCRPC (i.e. 2/3 had prior taxanes, and 30-40% had extensive visceral disease) who have a homologous recombination repair (HRR) mutation and have progressed on prior treatment with new hormonal anticancer treatments. Among the *BRCA1/2* and *ATM* cohort olaparib doubled radiographic progression-free survival (rPFS) from 3.55 months in the abiraterone acetate plus prednisone/enzalutamide arm to 7.39 months for olaparib (hazard ratio (HR) 0.35, 95% confidence interval (CI) 0.25-0.47).

1.4 Summary niraparib plus abiraterone acetate plus prednisone

The investigators aim to test the hypothesis that HRR deficient metastatic prostate cancer patients will demonstrate robust durable responses to combination therapy with niraparib and abiraterone acetate plus prednisone.

2. STUDY POPULATION

During ProBio, the combination therapy with niraparib and abiraterone acetate plus prednisone will be tested in male patients, aged above 18 years, with histologically confirmed prostate adenocarcinoma, initiating systemic therapy for metastatic disease, encompassing both newly diagnosed (i.e. *de novo*) metastatic hormone-sensitive prostate cancer (mHSPC) and metastatic castration-resistant prostate cancer (mCRPC).

3. PACKAGING, LABELING AND STORAGE

Niraparib and abiraterone acetate will be provided by the pharmaceutical collaborator, i.e. Janssen. IMP supply and labeling activities will be performed by the third party vendor Clinical Supplies Management Europe sa (CSM, a CliniGen company). Sufficient study drugs for each treatment cycle will be distributed on the first day of each cycle. Detailed conditions for the use of the study treatment niraparib, either as single agent or as a fixed dose combination (FDC) with abiraterone acetate, and contraindications, special warnings and precautions for use are described in the investigator's brochures (IB) (*INVESTIGATOR'S BROCHURE NIRAPARIB, Version Number 11 and version number 11 addendum 1; and INVESTIGATOR'S BROCHURE CJNJ-67652000 (niraparib/abiraterone acetate fixed-dose combination), version number 1*). Detailed conditions for the use of the study treatment abiraterone acetate and contraindications, special warnings and precautions for use are described in the SmPC.

4. DOSES AND TREATMENT REGIMENS

4.1 Treatment regimens

All subjects will receive abiraterone acetate, which will be administered at a total dose of 1000 mg per day by oral administration. Together with abiraterone acetate, concomitant prednisone will also be administered as 5 mg tablets for oral administration. The total daily dose prednisone is dependent on the disease stage of the patient:

Prednisone

- For mHSPC: 5mg/day
- For mCRPC: 10 mg/day

All subjects will receive niraparib, which will be administered at a total dose of 200 mg per day by oral administration. This dose was selected based on data from the ongoing Phase 1b Study 64091742PCR1001 evaluating the recommended Phase 2 dose for the combination of niraparib and abiraterone acetate. In context of dose modifications (see *'4.2 Dose modifications and management of toxicity'*), a one dosage level reduction equals to 100 mg niraparib.

The ProBio trial will incorporate niraparib and abiraterone acetate as single agents or as a fixed dose combination (FDC). Thus, following single drug and FDC formulations will be employed during ProBio:

Single agents of niraparib and abiraterone acetate

- Niraparib: 2 x 100 mg/day
- Abiraterone acetate 4 x 250 mg/day

Fixed dose combinations (FDC) of niraparib and abiraterone acetate

2 formulations will be made available:

- 100 mg niraparib - 500 mg abiraterone acetate, 2 tablets / day
- 50 mg niraparib - 500 mg abiraterone acetate, 2 tablets / day

Study drugs will be administered on an outpatient basis and a treatment cycle is defined as 28 days. Subjects will begin taking study drugs after Visit 3 (mHSPC) or after Visit 2 (mCRPC), i.e. Treatment initiation visit after randomisation. Subjects should take their daily dose of study drugs and no food or liquids should be consumed for at least 2 hours before and for at least 1 hour after dosing. The study drugs should be swallowed whole with water. Study drugs should be administered together, except for prednisone, which may be taken at any time.

Next to the experimental treatment niraparib plus abiraterone acetate plus prednisone, all subjects will receive background androgen deprivation therapy (ADT), as described in *'5.1 Standard-of-care androgen deprivation therapy (ADT) as background systemic therapy'* in the ProBio Clinical Study Master Protocol version 4.1.

4.2 Dose modifications and management of toxicity

Niraparib dose reductions instructions, for both as a single agent and fixed dose combinations (FDC), in context of specific adverse events are described in detail in following chapters.

In general, when single agents are used, dose interruptions/modifications should be managed as follows:

- Grade 1 or Grade 2 toxicities should be managed symptomatically without requiring dose adjustments or dose interruptions; however, closer monitoring (laboratory or clinic visits) should be considered
- The dose of prednisone can remain unchanged with dose modifications of niraparib or abiraterone acetate

Alternatively, when fixed dose combinations (FDC) are used, the patient can be switched to the 50 mg niraparib - 500 mg abiraterone acetate formulation. In case abiraterone acetate dose reduction would be needed whilst the patient is treated with a FDC, a switch to the single agents needs to be made.

In general, management of toxicities using the FDC should be performed as described in 'Table 1 - General Dose Reduction Guidelines for Participants on the FDC Treatment Regimen', and dose interruptions/modifications should be managed as follows:

- The dose of prednisone can remain unchanged with dose modifications of niraparib and/or abiraterone acetate (AA).
- If either niraparib or AA is permanently discontinued due to toxicity, the other agent may be continued as a single agent. Prednisone should be discontinued (with a taper if clinically indicated) if AA is permanently discontinued.

Table 1 - General Dose Reduction Guidelines for Participants on the FDC Treatment Regimen

Dose Required	Dose of Niraparib	Dose of AA	Instruction
Full dose	200 mg	1000 mg	Participants will receive regular-strength FDC tablets, if available. Otherwise they will receive a single-agent combination of niraparib and AA
Reduced niraparib	100 mg	1000 mg	Participants will receive low-strength FDC, if available. Otherwise they will receive a single agent combination of niraparib and AA.
Reduced AA	200 mg (or 100 mg)	250-750 mg	Participants will receive a single agent combination of niraparib and AA.
AA only		500-1000 mg	Participants will receive AA.
AA = abiraterone acetate; FDC = fixed-dose combination			

Any dose/dosage adjustments should be overseen by medically qualified study-site personnel (principal or sub-investigator unless an immediate safety risk appears to be present). All dose interruptions and reductions (including missed dose) and the reason for the interruption/reduction are to be recorded in the study medication eCRF form. Note that cycle days are fixed based on the Cycle 1 Day 1 (i.e. Treatment initiation visit after randomisation) date and will not change due to dose interruptions or delays. Management of toxicities should be performed in general as described in *Table 1, and* as detailed in Sections *'4.3 Non-hematological toxicities'* and *'4.4 Hematological toxicities'*. Once the dose of the study drug is reduced, any re-escalation to a full starting dose must be discussed in advance with the Study Sponsor via ProBioPV@meb.ki.se (with the exception of dose reduction for liver function test (LFT)-related toxicity, for which no dose re-escalation will be permitted, as discussed in section *'4.3.1 Hepatic toxicities'*).

If either study drug is permanently discontinued due to toxicity, the other study drug (niraparib or abiraterone acetate) may be continued. Prednisone should be discontinued (with a taper if clinically indicated) if abiraterone acetate is permanently discontinued.

4.3 Non-hematologic toxicities

For subjects who develop drug-related Grade 3 or higher toxicities, treatment should be withheld unless appropriately managed per institutional standard. Treatment with study drug must not be reinitiated until symptoms of the toxicity have resolved to Grade 1 or baseline. If the toxicity cannot be definitively attributed to either niraparib or abiraterone acetate only, then both niraparib or abiraterone acetate should be interrupted. If study drugs are to be restarted, then abiraterone acetate should be restarted first. If there is continued resolution of the AE to baseline/Grade 1, then niraparib may be restarted at least 7 days after restarting abiraterone acetate. If dose reduction is used for AE management, then please note the following:

- Only 1 dose-level reduction will be permitted for niraparib (from 200mg to 100mg)
- For abiraterone acetate, up to 2 dose-level reductions are permitted. At each dose-level reduction, the dose will be reduced by 1 tablet (250mg) of abiraterone acetate, e.g. 4 to 3 tablets or 3 to 2 tablets.

4.3.1 Hepatic toxicities

Niraparib/AA as Single Agents

Hepatic toxicities are a known potential side effect of abiraterone acetate plus prednisone and niraparib, but are more common with abiraterone acetate plus prednisone treatment. *'Table 2 - Dose modification criteria for AST/ALT/Bilirubin abnormalities for niraparib and abiraterone acetate plus prednisone'* provides dose recommendations for subjects who develop liver function test (LFT) abnormalities during treatment with niraparib and abiraterone acetate plus prednisone. During dose interruptions, LFTs should be monitored at least weekly until Grade 1 or baseline. For subjects being retreated, serum transaminases should be monitored at a minimum of every 2 weeks for 3 months and monthly for the next 3 months.

Table 2 - Dose modification criteria for Hepatic toxicities (i.e. AST/ALT/Bilirubin abnormalities) for niraparib and abiraterone acetate (AA) plus prednisone

Toxicity Grade	Dose of Niraparib	Dose of AA	Dose of Prednisone
Grade 1 -2	No Change	No Change	No Change
Grade 3	Interrupt and return to baseline. Then, resume at previous dose at least 7 days after AA has been started without AST/ALT/bilirubin abnormalities and only after discussion and agreement with Study Sponsor. AST/ALT/bilirubin must be confirmed Grade 1 or baseline before restarting.	Interrupt until return to baseline or to AST or ALT ≤3 x ULN and total bilirubin ≤1.5 x ULN, resume at 750 mg (3 tablets) only after discussion and agreement with Study Sponsor.	No Change
Recurrence Grade 3	Interrupt and return to baseline. Then, resume at 100 mg (1 capsule) at least 7 days after AA has been started without AST/ALT/bilirubin abnormalities and only after discussion and agreement with Study Sponsor.	Interrupt until return to baseline or to AST or ALT ≤3 x ULN and total bilirubin ≤1.5 x ULN, resume at 500 mg (2 tablets) only after discussion and agreement with Study Sponsor.	No Change
Grade 4	Must be interrupted and discussed with Study Sponsor	Must be interrupted and discussed with Study Sponsor.<br><br>If ALT is ≥20 x ULN, discontinue and do not re-treat with AA.	No Change or consider tapering if AA discontinued
AA=abiraterone acetate; AE=adverse event; ALT=alanine transferase; AST=aspartate transferase; FDC=fixed-dose combination; LFT=liver function test; LLN=lower limit of normal; ULN=upper limit of normal; WNL=within normal limits			

If clinical symptoms or signs suggestive of hepatotoxicity develop, serum transaminases should be measured immediately. If a subject develops severe hepatotoxicity (ALT ≥ 20 x ULN) anytime while receiving abiraterone acetate, subjects should be discontinued from treatment and retreatment with abiraterone acetate should not be attempted. Re-escalation of abiraterone acetate or niraparib is not permitted if the dose reduction was due to elevated LFTs. Subjects who develop a concurrent elevation of ALT > 3 x ULN and a total bilirubin > 2 x ULN in the absence of biliary obstruction or other causes responsible for the concurrent elevation should be permanently discontinued from treatment with study drugs.

Niraparib/AA as Fixed Dose Combination

Hepatic toxicities are a known potential side effect of the treatment with niraparib/AA FDC, which cannot be used in subjects with severe hepatic impairment. Whereas for patients with a pre-existing mild hepatic impairment no dosage adjustment is necessary, the usage of niraparib/AA FDC for patients with pre-existing moderate hepatic impairment, should only be considered if the benefit clearly outweighs the possible risk. As the starting dose of AA for subjects with moderate hepatic

impairment should be reduced to 250 mg once daily, niraparib/AA FDC should not be used and should be replaced with 200 mg niraparib and 250 mg AA once daily as single agents.

Serum transaminase and bilirubin levels should be measured prior to starting niraparib/AA FDC, every 2 weeks for the first 3 months of treatment, and monthly thereafter or per protocol. If clinical symptoms or signs suggestive of hepatotoxicity develop, serum transaminases, in particular serum ALT, should be measured immediately. During dose interruptions, LFTs should be monitored (local laboratory) at least weekly. For grade ≥2 LFT abnormalities, LFTs should be monitored at least weekly until grade 1 or baseline. Re-treatment with niraparib/AA FDC may take place only after the return of liver function tests to the patient's baseline and at a reduced dose level. For participants being retreated, serum transaminases should be monitored at a minimum of every 2 weeks for 3 months and monthly for the next 3 months and then follow SoA. If hepatotoxicity recurs at the reduced dose level, discontinue treatment with niraparib/AA FDC. Participants who develop a concurrent elevation of ALT >3 x ULN and a total bilirubin >2 x ULN in the absence of biliary obstruction or other causes responsible for the concurrent elevation should be permanently discontinued from treatment with study medications. If at any time the patient develops severe hepatotoxicity (ALT 20 x ULN), niraparib/AA FDC should be discontinued, and patients should not be retreated. *'Table 3 - Dose modification criteria for AST/ALT/Bilirubin abnormalities for Participants on the FDC Treatment Regimen'* provides dose recommendations for subjects who develop liver function test (LFT) abnormalities during treatment with the FDC of niraparib and abiraterone acetate plus prednisone.

Table 3 - Dose modification criteria for Hepatic toxicities (i.e. AST/ALT/Bilirubin abnormalities) for participants on the FDC treatment regimen

**Adverse Event**	**Grade**	**Action**
↑AST, ALT, bilirubin	1-2	No change in Niraparib or AA
↑AST, ALT, bilirubin	3	**Niraparib:** Interrupt and return to baseline or grade 1. Then, resume single agent niraparib at previous dose at least 7 days after AA has been started without AST/ALT/bilirubin abnormalities and only after discussion and agreement with Study Sponsor. AST/ALT/bilirubin must be confirmed grade 1 or baseline before restarting.<br>**AA:** Interrupt and repeat liver function tests once a week until return to baseline or to AST or ALT ≤3 x ULN and total bilirubin ≤2.0 x ULN, resume at 500 mg only after discussion and agreement with Study Sponsor.
↑AST, ALT, bilirubin	Recurrence Grade 3	**Niraparib**: Interrupt and return to baseline or grade 1. Then, resume at 100 mg of niraparib at least 7 days after AA has been started without AST/ALT/bilirubin abnormalities and only after discussion and agreement with Study Sponsor.<br>**AA**: Interrupt and repeat liver function tests once a week until return to baseline or to AST or ALT ≤3 x ULN and total bilirubin ≤2.0 x ULN, resume at 500 mg only after discussion and agreement with Study Sponsor.
↑AST, ALT, bilirubin	4	**Niraparib**: Must be interrupted and discussed with Study Sponsor.<br>**AA**: Must be interrupted and discussed with Study Sponsor. If ALT is ≥20 x ULN, discontinue and do not re-treat with AA.
AA=abiraterone acetate; AE=adverse event; ALT=alanine transferase; AST=aspartate transferase; FDC=fixed-dose combination; LFT=liver function test; LLN=lower limit of normal; ULN=upper limit of normal; WNL=within normal limits		

4.3.2 Hypokalemia

Niraparib/AA as Single Agents

For subjects who develop hypokalemia on study treatment, maintenance of the subject's potassium level at 4.0 nM or higher should be considered. If hypokalemia persists despite optimal potassium supplementation and adequate oral intake, the dose of prednisone may be increased by 5 mg/day and documented in the study medication eCRF form. The increased dose of prednisone (or prednisolone) must be obtained by locally from an open-label source (e.g. prescription provided by investigator) and documented as a concomitant medication. For Grade ≥3 hypokalemia, abiraterone acetate must be interrupted, and appropriate medical management instituted (e.g. obtain ECG and provide potassium supplement). Treatment with abiraterone acetate should not be reinitiated until hypokalemia has resolved to Grade 1 or baseline. Hypokalemia is also an AE associated with niraparib. If Hypokalemia does not resolve with an abiraterone acetate dose reduction, consider a one dose level reduction for niraparib. For Grade 3-4 hypokalemia events, consider hospitalization of the subject.

Niraparib/AA as Fixed Dose Combination

Before starting niraparib/AA FDC, pre-existing hypokalemia must be corrected. *'Table 4 - Dose modification criteria in context of hypokalemia for Participants on the FDC Treatment Regimen'* provides dose recommendations for subjects who develop hypokalemia during treatment with the niraparib/AA FDC.

Table 4 - Dose modification criteria in context of hypokalemia for participants on the FDC treatment regimen

Adverse Event	Grade	Action
Hypokalemia (<LLN -3 mmol/L to symptomatic with ≤LLN - 3 mmol/L)	1 - 2	Maintenance of the participant's potassium level at ≥4.0 mmol should be considered.<br><br>If hypokalemia persists despite optimal potassium supplementation and adequate oral intake, the dose of prednisone may be increased by 5 mg/day and documented in the study medication electronic case report form.
Hypokalemia (<3.0 mmol/L)	3 - 4	AA must be interrupted, and appropriate medical management instituted (eg, obtain electrocardiogram and provide potassium supplement).<br><br>Treatment with AA should not be reinitiated until hypokalemia has resolved to grade 1 or baseline.<br><br>Hypokalemia is also an AE associated with niraparib – if hypokalemia does not resolve with an AA dose reduction, consider niraparib dose a one dose level reduction.<br><br>For grade 3-4 hypokalemia events, consider hospitalization of the participant.
AA=abiraterone acetate; AE=adverse event; LLN=lower limit of normal		

4.3.3 Hypertension

Niraparib/AA as Single Agents
For subjects who develop hypertension Grade 1-2, closely monitor participants´s BP and provide medical intervention for BP. No change to study drug regimen is needed. For Grade 3, niraparib, abiraterone acetate and prednisone must be interrupted until BP returns to baseline or Grade 1, and appropriate medical management instituted (more intensive monitoring of BP and consider additional anti-hypertensive medications).

For re-occurrence of Grade 3, doses of niraparib and abiraterone acetate must be reduced to 10 mg niraparib and 500 mg abiraterone acetate, and continue intensive BP monitoring. For Grade 4, discontinue all study medication and urgent intervention is required.

Niraparib/AA as Fixed Dose Combination
Niraparib/AA FDC should be used with caution in patients with a history of cardiovascular disease, or whose underlying medical conditions might be compromised by increases in blood pressure, hypokalemia, or fluid retention (eg, heart failure, recent myocardial infarction, or ventricular arrhythmia). Before starting niraparib/AA FDC, pre-existing hypertension must be controlled.

During treatment with niraparib/AA FDC, blood pressure and heart rate should be monitored at least weekly for the first two months, then monthly for the first year and periodically thereafter. Serum potassium, and fluid retention should be monitored at least monthly or per protocol. Hypertension should be medically managed with antihypertensive medicines as well as dose adjustment of niraparib/AA FDC, if necessary.

Niraparib/AA FDC should be discontinued in case of hypertensive crisis or if medically significant hypertension cannot be adequately controlled with antihypertensive therapy. *'Table 5 - Dose modification criteria in context of hypertension for Participants on the FDC Treatment Regimen'* provides dose recommendations for subjects who develop hypokalemia during treatment with the FDC of niraparib and abiraterone acetate plus prednisone.

Table 5 - Dose modification criteria in context of hypertension for participants on the FDC treatment regimen

Adverse Event	Grade	Action
Systolic BP up to 140-159 mm Hg or diastolic BP up to 90-99 mm Hg if previously WNL	1-2	Closely monitor participant's BP.<br><br>Medical intervention for BP (monotherapy).<br><br>No change to study drug regimen.
Systolic BP ≥160 mm Hg or diastolic BP ≥100 mm	3	More intensive monitoring of BP; consider adding additional anti-hypertensive medications to the participant's regimen.<br><br>Hold niraparib, AA, and prednisone until BP returns to baseline or grade 1.
Systolic BP ≥160 mm Hg or diastolic BP ≥100 mm despite a multiple drug regimen	Re-occurrence of Grade 3	Dose reduce so that new dose = 100 mg niraparib and 500 mg AA – discuss with Study Sponsor.<br><br>Continue intensive BP monitoring.
Life threatening consequences (eg, malignant hypertension, transient or permanent neurologic deficit, hypertensive crisis)	4	Discontinue all study medication.<br><br>Urgent intervention required.
AA=abiraterone acetate; AE=adverse event; LLN=lower limit of normal; PBP=blood pressure; WNL=within normal limits		

4.3.4 Posterior Reversible Encephalopathy Syndrome (PRES)

Niraparib/AA as Single Agents or Fixed Dose Combination

PRES is a rare neurological disorder that can present with the following signs and symptoms including seizures, headache, altered mental status, visual disturbance, or cortical blindness, with or without associated hypertension. A diagnosis of PRES requires confirmation by brain imaging, preferably magnetic resonance imaging. There have been rare reports of the development of signs and symptoms that are consistent with PRES in patients treated with niraparib. In patients developing Posterior Reversible Encephalopathy Syndrome (PRES), treatment of specific symptoms including control of hypertension is recommended, along with discontinuation of niraparib/AA FDC.

4.4 Hematologic toxicities

Niraparib/AA as Single Agents

Hematologic toxicities are not a known side effect of abiraterone acetate and treatment with abiraterone acetate-prednisone should generally be continued during niraparib dose interruptions/modifications. Niraparib dose interruption/modification criteria for platelet and neutrophil count abnormalities are described in *'Table 6 - Niraparib dose modification criteria in context of decreased platelet and neutrophil count abnormalities for participants on the single agents treatment regimen'*. For the management of anemia, supportive measures such as blood transfusions may be performed as deemed necessary by the investigator per institutional standard-of-care. For Grade ≥ 3 anemia, niraparib should be interrupted until resolution to Grade < 3. The site should contact the Study Sponsor for discussion and consider discontinuation of niraparib if:

- Hematologic toxicity has not recovered to Grade 1 or baseline after prolonged period of dose interruption
- A diagnosis of MDS/AML is confirmed by a hematologist

Table 6 - Niraparib dose modification criteria in context of decreased platelet, hemoglobin, and neutrophil counts abnormalities for participants on the single agents treatment regimen

AE Grade	Action
1	No change, consider weekly monitoring.
2	At least weekly monitoring and consider interrupting niraparib until ≤Grade 1 or baseline and then resume at same dose with recommendation of weekly monitoring for 28 days after restart.
≥3	Interrupt niraparib until ≤Grade 1 or baseline, then:<br>- Resume at 200 mg or 1 dose-level reduction (at the discretion of the investigator).<br>- If participant was already on reduced dose at 100 mg, (because of non-hematologic toxicity), discuss with Study Sponsor prior to resuming treatment.<br><br>Weekly monitoring is required until resolution to Grade 1 or baseline.<br><br>Weekly monitoring is recommended for 28 days after restarting dose.
Second Occurrence Grade ≥3	Interrupt niraparib until ≤Grade 1 or baseline and restart at 1 dose-level reduction.<br><br>Weekly monitoring is required until resolution to grade 1 or baseline. Weekly monitoring is recommended for 28 days after restarting dose.<br><br>If participant was on 100 mg (because of non-hematologic toxicity), discuss with Study Sponsor prior to resuming treatment.
Third Occurrence Grade ≥3	Permanently discontinue niraparib if hemoglobin, neutrophils, or platelets do not return to grade 1 or baseline within 28 days of dose interruption, and already on niraparib 100 mg dose.

**Table 6 - Niraparib dose modification criteria in context of decreased platelet, hemoglobin, and neutrophil counts abnormalities for participants on the single agents treatment regimen - continued**
**Notes:**<br>For participants with a platelet count ≤10,000 cells/µL, prophylactic platelet transfusion per guidelines may be considered. For participants taking anti-coagulant or anti-platelet therapy, consider the risk/benefit of interrupting these drugs or prophylactic transfusion at an alternative threshold such as ≤20,000 cells/µL.<br><br>Weekly monitoring and/or interruption are not required if at baseline grade, eg, a participant with a baseline hemoglobin of 9.1 g/dL (grade 2 anemia) does not need to be monitored weekly for grade 1 or 2 anemia.<br><br>If a participant requires platelet transfusion or has neutropenic fever or neutropenia requiring granulocytecolony stimulating factor for a grade ≥3 AE deemed to be related to niraparib toxicity, interrupt study medication and restart at 1 dose-level reduction after resolution to grade 1 or baseline. Continue AA at single agent product at same dose. If the participant was previously dose-reduced for the same hematologic toxicity, discontinue niraparib.

Niraparib/AA as Fixed Dose Combination

Hematological ADRs (thrombocytopenia, anemia, neutropenia, and pancytopenia), including clinical diagnoses and/or laboratory findings, have been reported in patients treated with niraparib. These ADRs generally occurred early during treatment, decreased over time, and were managed with laboratory monitoring and dose modifications. Niraparib/AA FDC should be discontinued if a patient develops severe persistent hematological toxicity that does not resolve within 28 days following interruption.

Niraparib/AA FDC dose interruption/modification criteria for platelet and neutrophil count abnormalities are described in *'Table 7 - Niraparib dose modification criteria in context of decreased platelet and neutrophil count abnormalities for participants on the FDC regimen'*.

Table 7 - Niraparib/AA FDC dose modification criteria in context of decreased platelet, hemoglobin, and neutrophil counts abnormalities for participants on the FDC treatment regimen

AE Grade	Action
1	No change, consider weekly monitoring.
2	At least weekly monitoring and consider interrupting niraparib until ≤Grade 1 or baseline and then resume at same dose with recommendation of weekly monitoring for 28 days after restart.
≥3	Interrupt niraparib until ≤grade 1 or baseline, then:<br>- Resume at 200 mg or 1 dose-level reduction (at the discretion of the investigator).<br>- If participant was already on reduced dose at 100 mg, (because of non-hematologic toxicity), discuss with Study Sponsor prior to resuming treatment.<br><br>Weekly monitoring is required until resolution to grade 1 or baseline.<br><br>Weekly monitoring is recommended for 28 days after restarting dose.
Second Occurrence Grade ≥3	Interrupt niraparib until ≤grade 1 or baseline and restart at 1 dose-level reduction.<br><br>Weekly monitoring is required until resolution to grade 1 or baseline. Weekly monitoring is recommended for 28 days after restarting dose.<br><br>If participant was on 100 mg (because of non-hematologic toxicity), discuss with Study Sponsor prior to resuming treatment.
Third Occurrence Grade ≥3	Permanently discontinue niraparib if hemoglobin, neutrophils, or platelets do not return to grade 1 or baseline within 28 days of dose interruption, and already on niraparib 100 mg dose.

Notes:

For participants with a platelet count ≤10,000 cells/µL, prophylactic platelet transfusion per guidelines may be considered. For participants taking anti-coagulant or anti-platelet therapy, consider the risk/benefit of interrupting these drugs or prophylactic transfusion at an alternative threshold such as ≤20,000 cells/µL.

Weekly monitoring and/or interruption are not required if at baseline grade, eg, a participant with a baseline hemoglobin of 9.1 g/dL (grade 2 anemia) does not need to be monitored weekly for grade 1 or 2 anemia.

If a participant requires platelet transfusion or has neutropenic fever or neutropenia requiring granulocytecolony stimulating factor for a grade ≥3 AE deemed to be related to niraparib toxicity, interrupt study medication and restart at 1 dose-level reduction after resolution to grade 1 or baseline. Continue AA at single agent product at same dose. If the participant was previously dose-reduced for the same hematologic toxicity, discontinue niraparib.

For the management of anemia, supportive measures such as blood transfusions may be performed as deemed necessary by the investigator per institutional standard-of-care. For grade ≥3 anemia, niraparib should be interrupted until resolution to grade <3.

The site should contact the Study Sponsor for discussion and consider discontinuation of FDC and switch to single agent AA if:

- Hematologic toxicity has not recovered to grade 1 or baseline after prolonged period of dose interruption
- A diagnosis of MDS/AML is confirmed by a hematologist.

One dosage level reduction = 100 mg niraparib.

5. PHARMACOVIGILANCE

5.1 Safety and Product Quality Data for the combination therapy niraparib plus abiraterone acetate

While niraparib is an investigational agent in metastatic prostate cancer, it has been approved for the treatment of ovarian cancer. Known toxicities include gastrointestinal events, haematological events and hypertension, which all are managed by appropriate medical interventions. For Niraparib, the expectedness of an adverse event will be determined by whether or not it is listed in the Investigator's Brochure (*INVESTIGATOR'S BROCHURE NIRAPARIB, Edition Version 11 and and version number 11 addendum 1; and INVESTIGATOR'S BROCHURE CJNJ-67652000 (niraparib/abiraterone acetate fixed-dose combination), version number 1*). Detailed conditions for the use of the study treatment abiraterone acetate and contraindications, special warnings and precautions for use are described in the SmPC.

5.1.1 Adverse Events of Special Interest (AESI)

For the investigational combination therapy of niraparib with abiraterone acetate plus prednisone there are AEs of special interest that Janssen is actively monitoring as a result of a previously identified signal (even if non-serious). The adverse events of special interest (AESI) are:

Niraparib:
- Anemia
- Thrombocytopenia
- Neutropenia
- MDS/AML

Abiraterone Acetate:
- Hypertension
- Hypokalemia
- Fluid retention/edema
- Hepatotoxicity
- Cataract
- Osteoporosis (including fracture)
- Rhabdomyolysis/myopathy
- Allergic alveolitis
- CYP2D drug interactions and food effect

Additionally, any serious adverse event (SAE) or adverse event of special interest (AESI) should be recorded in the eCRF Adverse Event Report Form and be reported to the Study Sponsor within 24 hours of becoming aware of the event. The Study Sponsor will report to Janssen within 24 hours after becoming aware of the event.

5.1.2 Special Situations

Additionally, they are safety events that may not meet the definition of an adverse event; however, the parties agree that for reporting purposes, they are deemed to be adverse events, and be reported to the Study Sponsor within 24 hours of becoming aware of the event. The Study Sponsor will report to Janssen within 24 hours after becoming aware of the event. Following special situations must be reported to Janssen with or without an associated serious adverse event (SAE):

- Drug exposure during pregnancy (paternal, maternal)
- Suspected transmission of any infectious agent via administration of a Janssen medicinal product
- Overdose of Janssen Product(s) Under Study
- Suspected abuse/misuse of Janssen Product(s) Under Study
- Inadvertent or accidental exposure to Janssen Product(s) Under Study
- Any failure of expected pharmacological action (i.e., lack of effect) of Janssen Product(s) Under Study
- Medication error (includes potential, intercepted or actual) involving a Janssen product (with or without patient exposure to the Janssen Product(s) Under Study, e.g., name confusion)
- Unexpected therapeutic or clinical benefit from use of Janssen Product(s) Under Study

5.1.3 Pregnancy

Niraparib has the potential to cause teratogenicity or embryo-fetal death because niraparib is genotoxic and targets actively dividing cells in animals and patients (eg, bone marrow). In animal studies, niraparib showed effects on sperm (reduced sperm count, spermatids, and germ cells in epididymites and testes). Based on animal studies, niraparib may impair fertility in males of reproductive potential. Abiraterone acetate was not noted to be genotoxic in vitro studies; however, abiraterone acetate is contraindicated in women who are or may become pregnant.Initial reports of pregnancy in partners of male subjects included in the study should be reported to the Study Sponsor within 24 hours of becoming aware of the event. The Study Sponsor will report to Janssen within 24 hours after their knowledge of the event, using the Serious Adverse Event Form. Abnormal pregnancy outcomes (eg, spontaneous abortion, fetal death, stillbirth, congenital anomalies, ectopic pregnancy) are considered SAEs and must be reported using the Serious Adverse Event Form. Depending on local legislation this may require prior consent of the partner. Follow-up information regarding the outcome of the pregnancy and any postnatal sequelae in the infant will be required. Pregnant partners of male subjects should also be appraised of the potential risks to the foetus.

5.1.4 Product quality complaints

Product Quality Complaint (PQC) for the combination therapy niraparib plus abiraterone acetate is defined as any suspicion of a product defect related to a potential quality issue during manufacturing, packaging, release testing, stability monitoring, dose preparation, storage or distribution of the product, or delivery system. Not all PQCs involve a subject. Lot and batch

numbers are of high significance and need to be collected whenever available. Examples of PQC include but not limited to:

- Mislabelling or misbranding
- Information concerning microbial contamination, including a suspected transmission of any infectious agent by a product
- Any significant chemical, physical, or other changes that indicate deterioration in the distributed product
- Any foreign matter reported to be in the product
- Mixed product, e.g., two drugs are mixed-up in the packaging process
- Incorrect tablet sequence (e.g., oral contraceptive tablets)
- Insecure closure with serious medical consequences, e.g., cytotoxics, child-resistant containers, potent drugs
- Suspected counterfeit or tampered product
- Potential Dosing Device Malfunction

5.2 Transmission Methods and Procedures for Reporting Safety Data and Product Quality Complaints (PQCs) to Janssen

5.2.1 Safety Reporting

All Adverse Events, Adverse Events of Special Interest, and special situations, whether serious or non-serious, related or not related, following exposure to a Janssen medicinal product (Niraparib plus Abiraterone Acetate) are to be documented by the investigator and recorded in the eCRF and in the subject's source records. Investigators must record in the eCRF their opinion concerning the relationship of the adverse event to the combination therapy niraparib plus abiraterone acetate. All (serious and non-serious) adverse events reported should be followed-up in accordance with clinical practice.

All SAEs, Adverse Events of Special Interest and Special Reporting Situations that have not resolved by the end of the study, or that have not resolved upon discontinuation of the subject's participation in the study, must be followed until any of the following occurs:

- The event resolves
- The event stabilizes
- The event returns to baseline, if a baseline value/status is available
- The event can be attributed to agents other than the study drug or to factors unrelated to study conduct
- It becomes unlikely that any additional information can be obtained (subject or health care practitioner refusal to provide additional information, lost to follow-up after demonstration of due diligence with follow-up efforts)

The institution and the site principal investigator will transmit all Serious Adverse Events, Adverse Events of Special Interest and Special Reporting Situations following exposure to the Janssen medicinal

product (Niraparib, Abiraterone acetate) in a valid Individual Case Study Report (ICSR) in accordance with Janssen requirements (*vide infra*), in English, within 24-hours of becoming aware of the event(s) to Study Sponsor (see Contact information for transmission of Safety Data and Product Quality Complaints related to niraparib plus abiraterone plus prednisone). The Study Sponsor has the obligation to report to Janssen within 24-hours after becoming aware of this information.

The ICSR must contain the four minimum criteria required to meet regulatory reporting requirements:

- an identifiable subject (but not disclosing personal information such as the subject's name, initials or address)
- an identifiable reporter (investigational site)
- a Janssen medicinal product
- an adverse event, outcome, or certain special situations

The minimum information required is:

- suspected Janssen medicinal product (doses, indication)
- date of therapy (start and end date, if available)
- batch or lot number, if available
- subject details (subject ID and country)
- gender
- age at (S)AE onset
- reporter ID
- adverse event detail (AE verbatim in English), onset date, relatedness, causality, action taken, outcome, (if available)
- Protocol ID

All follow-up information for Serious Adverse Events, Adverse Events of Special Interest and Special Reporting Situations that are not resolved at the end of the study or by the time of patient withdrawal must be reported directly by the site principal investigator, within 24 hours becoming aware using the Serious Adverse Event Report. All available clinical information relevant to the evaluation of a related SAE, Adverse Events of Special Interest, Serious Adverse Drug Reaction (ADR) or Special Reporting Situation is required. The Study Sponsor has the obligation to report to Janssen within 24 hours after becoming aware of this information.

The site principal investigator and Study Sponsor are responsible for ensuring that these cases are complete and if not are promptly followed-up. A safety report is not considered complete until all clinical details needed to interpret the case are received. Reporting of follow-up information should follow the same timeline as initial reports.

Copies of any and all relevant extraordinary (not including routine initial or follow-up ICSR submission) correspondences with regulatory authorities and ethics committees regarding any and all serious adverse events, irrespective of association with the combination therapy niraparib plus abiraterone acetate under study, are to be provided by the Study Sponsor to Janssen within 24 hours of such report or correspondence being sent to applicable health authorities.

5.2.2 PQC Reporting

A PQC may have an impact on the safety and efficacy of the product. Timely, accurate, and complete reporting and analysis of PQC information from studies are crucial for the protection of patients, investigators, and the company, and are mandated by regulatory agencies worldwide. Janssen has established procedures in conformity with regulatory requirements worldwide to ensure appropriate reporting of PQC information. Lot and/or Batch numbers or any report on the failure of expected pharmacological action (i.e., lack of effect) shall be collected. The product should be quarantined immediately and if possible, a picture of the particular Lot/Batch should be obtained.

All initial PQCs involving the combination therapy niraparib plus abiraterone acetate under study must be reported to Study Sponsor by the study principal investigator within 24 hours after being made aware of the event. The Study Sponsor will report to CSM study-specific manager within 24 hours after becoming aware of this information. The CSM study-specific manager will provide additional information forms to be completed.

If the defect for the combination therapy niraparib plus abiraterone acetate under study is combined with either a serious adverse event or non-serious adverse event, the study principal investigator must report the SAE related to the PQC to Study Sponsor according to the Serious Adverse Event reporting timelines. The Study Sponsor will report to Janssen within 24 hours after becoming aware of the event. A sample of the suspected product should be maintained for further investigation if requested by Janssen.

5.2.3 Contact information for transmission of Safety Data and Product Quality Complaints to Study Sponsor

The transmission of safety data (SAE, AESI) and product quality complaints from individual sites within participating countries need to be performed to the Study Sponsor (i.e. Karolinska Institute), who subsequently has the responsibility to inform the respective country pharmacovigilance (PV) divisions of Janssen (*Figure 1 - Transmission procedures from recruiting sites to study sponsor and from study sponsor to Janssen*).

Contact details study sponsor:

Study Sponsor Point of contacts	Contact details
Coordinating Investigator	email: henrik.gronberg@ki.se<br>Tel: +46 (0)70-341 13 56
Project Manager | Pharmacovigilance	e-mail: ProBioPV@meb.ki.se<br>Tel: +46 (0)8-524 825 76 | +46 (0)70-263 52 97

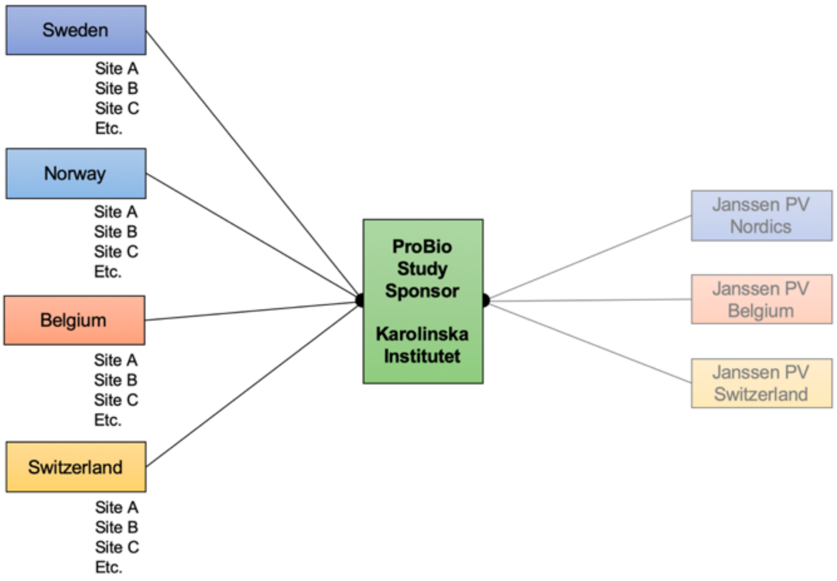

Figure 1 - Transmission procedures from recruiting sites to study sponsor and from study sponsor to Janssen

TRIAL ADMINISTRATION, INVESTIGATORS AND SITES

Trial title:

ProBio:
An outcome-adaptive and randomised multi-arm biomarker driven study in patients with metastatic prostate cancer

Coordinating Investigator and Sponsor's representative

Henrik Grönberg
Karolinska Institutet

TABLE OF CONTENTS

TABLE OF CONTENTS 2

1. TRIAL ADMINISTRATIVE STRUCTURE 3

2. INTERNATIONAL PRINCIPAL INVESTIGATORS 5

3. PARTICIPATING SITES AND SITE PRINCIPAL INVESTIGATORS 6

3.1 SWEDEN 6

3.2 BELGIUM 7

3.3 NORWAY 8

3.4 SWITZERLAND 9

4. MONITORING 10

5. DATA SAFETY AND MONITORING BOARD (DSMB) 11

6. SCIENTIFIC ADVISORY BOARD (SAB) 12

7. EMERGENCY CONTACTS 13

1. TRIAL ADMINISTRATIVE STRUCTURE

This is an investigator-initiated study where Professor Henrik Grönberg will be the Sponsor representative and the general Coordinating Investigator. The Sponsor is Karolinska Institute. The study will be administered by the ProBio Study Group, which includes the members listed in the table below. Departments of oncology and urology in Sweden, Belgium, Norway and Switzerland with a high volume of metastatic prostate cancer patients, will be asked to participate in the study.

NAME	ROLE	TITLE	ADDRESS	PHONE	EMAIL
Henrik Grönberg	Sponsor<br><br>Coordinating investigator	MD, PhD, Professor	Department of Medical Epidemiology and Biostatistics, Karolinska Institutet, Box 281, 17177 Stockholm<br><br>Capio S:t Görans Sjukhus<br>112 81 Stockholm	08-524 824 23	henrik.gronberg@ki.se
Tobias Nordström	Sponsor Urology	MD, PhD	Danderyds Hospital Entrévägen 2, 182 57 Danderyd, Sweden		tobias.nordstrom@ki.se
Martin Eklund	Sponsor Statistics	PhD	Department of Medical Epidemiology and Biostatistics, Karolinska Institutet, Box 281, 17177 Stockholm	08-524 823 72	martin.eklund@ki.se
Johan Lindberg	Sponsor Genomics, unblinded.	PhD	Department of Medical Epidemiology and Biostatistics, Karolinska Institutet, Box 281, 17177 Stockholm	08-524 885 01	johan.lindberg@ki.se
Mark Divers	Biobank for research samples		Karolinska Institutet<br>Box 281<br>171 77 Stockholm	08-524 823 01	Mark.divers@ki.se

Berit Larsson	Project manager ProBio study	MSc	Department of Medical Epidemiology and Biostatistics, Karolinska Institutet, Box 281, 17177 Stockholm	08-524 825 76	berit.larsson@ki.se
Bram De Laere	ProBio unblinded researcher<br><br>Liaison Sweden – Belgium	PhD	Department of Human Structure and Repair Ghent University B-9000 Ghent, C. Heymanslaan 10, Belgium<br><br>Department of Medical Epidemiology and Biostatistics Karolinska Institute S-171 77 Stockholm, Nobels väg 12 A, Sweden	+32496058111	bram.de.laere@ki.se<br>bramdelaere@gmail.com<br>bramdlae.DeLaere@ugent.be
Alessio Crippa	ProBio unblinded statistician	PhD	Karolinska Institutet, Stockholm		alessio.crippa@ki.se
Andrea Discacciati	ProBio unblinded statistician	PhD	Karolinska Institutet, Stockholm		andrea.discacciati@ki.se
Sven Burman	ProBio project manager	M.Sc.	Department of Medical Epidemiology and Biostatistics, Karolinska Institutet, Box 281, 17177 Stockholm	0733544094	sven.burman@ki.se

2. INTERNATIONAL PRINCIPAL INVESTIGATORS

Sponsor and general Coordinating Investigator

Professor Henrik Grönberg, MD, PhD		Signature		Date

Coordinating Investigator Sweden

Professor Henrik Grönberg, MD, PhD		Signature		Date

Coordinating Investigator Belgium

Professor Piet Ost, MD, PhD		Signature		Date

Coordinating Investigator Norway

Professor Jan Oldenburg, MD, PhD		Signature		Date

Coordinating Investigator Switzerland

Ashkan Mortezavi, MD, PhD		Signature		Date

3. PARTICIPATING SITES AND SITE PRINCIPAL INVESTIGATORS

3.1 SWEDEN

SITE (ID)	LOCATION	SITE PI	CONTACT	STATUS	TIV	FPI
Capio S:t Görans Sjukhus (SG)	Stockholm	Marie Hjälm-Eriksson	Marie.Hjalm-Eriksson@capiostgoran.se	Recruiting	2019-01-08	2019-01-29
Karolinska Universitetssjukhuset (KS)	Stockholm	Anders Ullén	Anders.Ullen@ki.se	Recruiting	2019-05-16	2019-06-19
Akademiska Sjukhuset (AK)	Uppsala	Gunilla Enblad	gunilla.enblad@igp.uu.se	Recruiting	2019-06-17	2019-06-18
Norrlands Universitetssjukhus (NU)	Umeå	Camilla Thellenberg Karlsson	camilla.thellenberg@umu.se	Recruiting	2019-05-03	2019-10-02
Länssjukhuset i Sundsvall (SU)	Sundsvall	Elin Jänes	Elin.janes@rvn.se	Recruiting	2019-06-12	2019-06-20
Centrallasarettet i Växjö (VX)	Växjö	Martha Olsson	Martha.olsson@kronoberg.se	Recruiting	2019-11-27	2020-08-06
Universitetssjukhuset i Linköping	Linköping	Nils Elander	nils.elander@regionostergotland.se	Pending	2021-02-01	
Skånes Universitetssjukhus	Lund			Pending		

Länssjukhuset i Kalmar	Kalmar	Mats Andén	mats.anden@regionkalmar.se	Pending	2020-12-10	
Länssjukhuset Ryhov	Jönköping	Linn Pettersson	linn.pettersson@rjl.se	Recruiting	2020-03-13	2020-09-14
Sahlgrenska Universitetssjukhuset	Göteborg	Ingela Franck Lissbrandt		Pending		
Centralsjukhuset	Karlstad	Johan Sandzén	johan.sandzen@regionvarmland.se	Recruiting	2020-11-09	2020-11-10

3.2 BELGIUM

SITE (ID)	LOCATION	SITE PI	CONTACT	STATUS	TIV	FPI
University Hospital Ghent (UZG)	Gent	Piet Ost	Piet.ost@ugent.be	Recruiting	2020-05-20	2020-06-29
AZ Sint-Jan AV (AZSJ)	Brugge	Christophe Ghysel	christophe.ghysel@azsintjan.be	Recruiting	2020-06-03	2020-08-13
University Hospital Leuven (UZL)	Leuven	Wouter Everaerts	wouter.everaerts@uzleuven.be	Pending	Pending	Pending
AZ Nikolaas (NIKO)	Sint-Niklaas	Els Everaert	els.everaert@aznikolaas.be	Recruiting	2020-10-06	2021-03-03
Jessaziekenhuis (JESS)	Hasselt	Daisy Luyten	daisy.luyten@jessazh.be	Recruiting	2020-10-19	Pending
Ziekenhuis Oost-Limburg (ZOLG)	Genk	Wendy De Roock	Wendy.DeRoock@zol.be	Recruiting	2020-11-17	Pending
AZ Groeninge (AZGR)	Kortrijk	Siska Van Bruwaene	SISKA.VANBRUWAENE@azgroeninge.be	Recruiting	2020-09-28	2020-10-06

AZ Sint-Lucas (AZSLB)	Brugge	Daan De Maeseneer	daan.demaeseneer@stlucas.be	Recruiting	2020-10-05	2021-01-18
University Hospital Luik (CHUL)	Luik	Brieuc Sautois	brieuc.sautois@chuliege.be	Recruiting	2020-10-05	2021-02-18
AZ Damiaan (DAMO)	Oostende	Jochen Darras	jdarras@azdamiaan.be	Recruiting	2020-10-12	Pending
AZ Sint Lucas (SLG)	Gent	Luc Merckx	Luc.Merckx@AZSTLUCAS.BE	Pending	Pending	Pending
OLV Ziekenhuis Aalst (OLVA)	Aalst	Peter Schatteman	peter.schatteman@olvz-aalst.be	Recruiting	2020-10-12	2021-03-02
AZ Jan Palfijn Ziekenhuis (JAPA)	Gent	Ines Samyn	Ines.Samyn@janpalfijngent.be	Pending	Pending	Pending

3.3 NORWAY

SITE (ID)	LOCATION	SITE PI	CONTACT	STATUS	TIV	FPI
Akershus Universitetssykehus	Lørenskog	Jan Oldenburg	oldenburg.jan@gmail.com	pending		
Haukeland Universitetssykehus	Bergen	Christian Ekanger	christian.ekanger@helse-bergen.no	pending		
Oslo Universitetssykehus	Oslo	Wolfgang Lilleby	wll@ous-hf.no	pending		
Stavanger Universitetssykehus	Stavanger	Maria Nyre Vigmostad	maria.nyre.vigmostad@sus.no	pending		
Tromsø Universitetssykehus	Tromsø	Hege Haugnes	hege.sagstuen.haugnes@uit.no	pending		
Trondheim Universitetssykehus	Trondheim	Torgrim Tandstad	Torgrim.Tandstad@stolav.no	pending		

Drammen sykehus	Drammen	Arne Berg	arber@vestreviken.no	pending		
Kreftsenter Kristiansand	Kristiansand	Christoph Müller	Christoph.Muller@sshf.no	pending		
Østfold Sykehus	Sarpsborg	Andreas Stensvold	Andreas.Stensvold@so-hf.no	pending		
Ålesund sykehus	Ålesund	Gunnar Indrebø	gunnar.indrebo@helse-mr.no	pending		
Levanger sykehus	Levanger	Oluf Dimitri Røe	oluf.roe@ntnu.no	pending		

3.4 SWITZERLAND

SITE (ID)	LOCATION	SITE PI	CONTACT	STATUS	TIV	FPI
University Hospital Basel	Basel	Ashkan Mortezavi	Ashkan@Mortezavi.com	Pending		

4. MONITORING

The trial sites will be visited by an appointed Monitor per country (*Table 1 - Country-specific trial monitor contact details*), periodically at times agreed with the Site Principal Investigator. It is the function of the Monitor to ascertain that all aspects of the protocol are compliant with and that the conduct of the trial conforms to applicable regulatory requirements and established rules for Good Clinical Practice (GCP). Country-specific Trial Monitor Plans will be available before start of study will be provided in the Trial Conduct Supplement '*MONITORING PLANS*'.

Preferably at the time of each monitoring visit, the Monitor will:

- review the completed eCRFs to ascertain that items have been completed and that the data provided are accurate and obtained in the manner specified in the protocol.
- verify that the data in the eCRF is consistent with the clinical records or other relevant record (Source Data Verification) and that trial results are recorded completely and correctly.
- verify compliance with the procedures for reporting of SAEs, product accountability and record keeping.

For this purpose the national Monitors must be given direct access to clinical records, original laboratory data, etc., as far as these relate to the trial and without jeopardizing patient integrity. The Investigator and other relevant personnel should be available during the monitoring visit and should devote sufficient time.

Table 1 - Country-specific trial monitor contact details

COUNTRY	NAME	ADDRESS	TELEPHONE	EMAIL
Sweden	Maria Persson	Karolinska Trial Alliance Sabbatsbergs sjukhus Olivecronas väg 15, 11361 Stockholm	08-517 71643<br>072-599 12 59	maria.h.persson@sll.se<br><br>hanna.lin.karlsson@sll.se
Belgium	Leen Geets<br><br>Sanne D'hondt	Health, Innovation and Research Institute, University Hospital Ghent, Belgium	+32 9 332 09 39	Leen.Geets@uzgent.be<br><br>Sanne.Dhondt@UZGENT.be
Norway	TBD			
Switzerland	TBD			

5. DATA SAFETY AND MONITORING BOARD (DSMB)

An independent Safety Data Monitoring Board will be responsible for evaluation of safety. The committee/board will consist of independent physicians and researchers. Please see *'Table 2 - Data and Safety Monitoring Board Charter'* for further contact details. A Data and Safety Monitoring Board (DSMB) will be formed to ensure participant safety in this clinical trial. DSMB members will also have additional responsibility for assurance that the trial is conducted to a high standard, and they may be involved in conduct and interpretation of data analyses for efficacy in addition to their primary responsibility for participant safety. The responsibilities of this group include reviewing quantitative recruitment and compliance progress for the study and recommending modifications of the trial protocol and/or administrative structure in the event these goals are not met. The committee will also review tabulated aggregate toxicity and endpoint data. The committee will submit written recommendations on the progress of the study to the Coordinating Investigator and study team. The Coordinating Investigator will ensure that relevant information will be disseminated to the participating Principal Investigators at each site. The DSMB will include a panel of experts recruited from outside of the institutions involved in this study. The DSMB will meet regularly during the study.

Table 2 - Data and Safety Monitoring Board Charter

NAME	ADDRESS	TELEPHONE	EMAIL
Chris Parker	Consultant Clinical Oncologist at the Academic urology unit<br>Royal Marsden Hospital<br>Sutton<br>Surrey<br>SM2 5 PT	Phone: +44-2086613425<br><br>Mobile: +44-7909525392	Chris.Parker@icr.ac.uk
Peter Iversen	Professor of Urology, Copenhagen Prostate Cancer Center, Rigshospitalet. Department of Clinical		Peter.Iversen@regionh.dk
Per Karlsson	Professor of Oncology and Chief Physician<br>Chairman department of Oncology<br>Sahlgrenska Academy / Sahlgrenska University Hospital<br>Gothenburg Sweden	+46-31-3428503	per.karlsson@oncology.gu.se
Jason Connor	CEO of Confluence Stat, LLC	+1-412-860-3113	Jason@confluencestat.com
Ahmet Zehir	Director Clinical Bioinformatics<br>Memorial Sloan Kettering Cancer Center, New York, New York	+1-646-888-3550	zehira@mskcc.org

6. SCIENTIFIC ADVISORY BOARD (SAB)

To be completed upon SAB assembly and protocol amendment

7. EMERGENCY CONTACTS

In case of a medical emergency, contact country sponsor representatives:

SWEDEN
Professor Henrik Grönberg
Mobile: +46 703411356 or
Project Manager Berit Larsson
Mobile: +46 702635297

BELGIUM
Professor Piet Ost
Mobile: +32 484 15 54 31 or
Project Manager
Mobile: to be completed upon protocol amendment

NORWAY
Professor Jan Oldenburg
Mobile: +47 95094528 or
Project Manager
Mobile: to be completed upon protocol amendment

SWITZERLAND
Ashkan Mortezavi
Mobile: +41 76 575 93 00 or
Project Manager
Mobile: to be completed upon protocol amendment

BIOMARKER SIGNATURES
Trial title:
**ProBio:**<br>**An outcome-adaptive and randomised multi-arm biomarker driven study in patients with metastatic prostate cancer**

Coordinating Investigator and Sponsor's representative

Henrik Grönberg
Karolinska Institutet

TABLE OF CONTENTS

TABLE OF CONTENTS 2

1. MOLECULAR GENOMIC PROFILING OF LIQUID AND TISSUE BIOPSIES TO INFER BIOMARKER SIGNATURES 3

2. PRESPECIFIED BIOMARKER SIGNATURES USED FOR RANDOMISATION 4

2.1 TP53 wild-type and AR wild type (TP53wt/ARwt) 5

2.2 TMPRSS2-ERG gene fusion (T-Efusion) 6

2.3 TP53-alterations (TP53m) 6

2.4 Homologous Recombination Deficiency (HRD) 7

2.4.1 Ad hoc subgroup analysis in context of the HRD biomarker signature 8

3. FUTURE PRESPECIFIED BIOMARKER SIGNATURES FOR RANDOMISATION UPON AVAILABILITY OF NEW IMP 9

3.1 Tumor mutational burden high, microsatellite instability or CDK12 inactivation (TMB-H/MSI+/CDK12-) 9

3.2 PI3K perturbed 10

1. MOLECULAR GENOMIC PROFILING OF LIQUID AND TISSUE BIOPSIES TO INFER BIOMARKER SIGNATURES

Extracted DNA from tissue, plasma and whole blood will be subjected to library preparation, in-solution based hybridization capture and sequencing. Depending on the tumor burden, as predicted by metastasis info (e.g. type and how many) together with routine blood laboratory analysis (e.g. LDH, ALP, PSA, cfDNA conc), either a focused biomarker signature design or a more comprehensive design will be applied (Figure 1). Bioinformatic processing will be performed at MEB (Stockholm, Sweden) using the AutoSeq pipeline (Clinseq Team), as previously applied in peer-reviewed publications (Mayrhofer et al. 2018; De Laere et al. 2019; De Laere et al. 2017), with the purpose to identify somatic- and germline alterations. Finally, the identified alterations will be semi-automatically curated using standard operating procedures and applied to categorize patients based on their biomarker signatures.

A)

		Comprehensive design	Biomarker signature design
**Mutations**			
	All coding exons	51 genes	16 genes
	Hotspots	27 genes	12 genes
**Pharmacogenetic variants**			
	SNPs	4 genes	0 genes
**Copy-number alterations**			
	Tiled SNP for genome-wide CNV	3128 SNPs	0 SNPs
	Directed analysis to increase sensitivty	20 genes	1 gene
**Structural variation**			
	Genefusions by intronic sequencing	3 genes	2 genes
	Gene-body sequencing (e.g. BRCA1/2)	8 genes	1 gene
**Microsatellite instability & Hypermutation**			
	Microsatellites	63	0
	Hypermutation, entire design footprint	Yes	No
	Associated genes	6	0
**DNA repair deficiency**			
	Associated genes	16	4
**Total size (Mb)**			
		1.5	0.3

B)

AKT1	BRCA2	CDKN2B	FOXO1	MED12	NBN	PMS1	RB1	ZBTB16
APC	BRIP1	CHD1	HRAS	MET	NCOR1	PMS2	RNF43	ZFHX3
AR	CCND1	CHEK2	IDH1	MGA	NKX3-1	POLD1	SETD2	ZMYM3
ARID1A	CDH1	CTNNB1	JAK1	MLH1	NRAS	POLE	SF3B1	
ARID2	CDK12	CUL3	KDM6A	MLH3	PALB2	PTEN	SPEN	
ATM	CDK4	DICER1	KEAP1	MRE11A	PIK3CA	RAD50	SPOP	
ATR	CDK6	DNMT3A	KMT2A	MSH2	PIK3CB	RAD51	TMPRSS2	
BARD1	CDKN1A	ERG	KMT2C	MSH3	PIK3CD	RAD51B	TP53	
BRAF	CDKN1B	FANCA	KMT2D	MSH6	PIK3R1	RAD51C	U2AF1	
BRCA1	CDKN2A	FOXA1	KRAS	MYC	PIK3R2	RAD51D	XPO1	

Figure 1 - ProBio assay overview. *A) Categorization of genomic alterations detected by the comprehensive- and biomarker signature designs. B) Green: Genes covered by both ProBio designs; Black: genes only covered by the comprehensive design.*

2. PRESPECIFIED BIOMARKER SIGNATURES USED FOR RANDOMISATION

The initial pre-defined biomarker signatures are defined as tumour properties or mutations in certain genes/pathways identified as potentially important in prostate cancer treatment response. These encompass the androgen receptor (*AR*), *TP53*, DNA repair deficiency (DRD), the *TMPRSS2-ERG* fusion and are described in detail in this document. However, depending on the stage of the disease at trial entry (i.e. *de novo* mHSPC and mCRPC), the AR biomarker will be approached differently. AR perturbations are uncommon (<2%) in mHSPC and emerge later during the course of the disease due to the selection pressure of androgen deprivation treatment (Stopsack et al. 2020; Mateo et al. 2020; Kohli et al. 2020). Thus the biomarker signature TP53 wild-type will be applied in the mHSPC setting whereas the TP53 wild-type/AR wild-type will be investigated for mCRPC. An overview of the utilised molecular biomarkers in the mHSPC and mCRPC setting of ProBio is depicted in *Table 1*.

Only clonal somatic alterations in DRD-genes and TP53 will be applied in ProBio to define the biomarker signatures. Somatic alterations are defined as clonal if the variant allele frequency (VAF) is ≥25% times the ctDNA fraction with a correction factor for structural variants, which are captured with poorer efficiency relatively to mutations or indels (Mayrhofer et al. 2018). The TMPRSS2-ERG gene fusion is an early event in localized prostate cancer development, even detected in cancer precursor lesions (Park et al. 2010). As metastatic prostate (mPC) cancer is a monoclonal disease (Liu et al. 2009), a patient will be designated as TMPRSS2-ERG gene fusion positive regardless of the VAF of the fusion event. Similarly, high-level AR amplifications are common in mCRPC with no current rationale how to interpret the VAF of hotspot mutations or intra-AR genomic structural rearrangements (GSR) (Annala et al. 2018; De Laere et al. 2019). Therefore, patients will be regarded as AR mutation positive or AR GSR positive regardless of the VAF.

Table 1 - Composition of initial biomarker signatures depending on the stage of the disease at trial entry

Stage	AR	TP53	DRDm	TE-fusion
mHSPC		x	x	x
mCRPC	x	x	x	x

2.1 TP53 wild-type and AR wild type ($TP53_{wt}/AR_{wt}$)

TP53 mutations and AR alterations have been associated with poor response to abiraterone or enzalutamide treatment (Romanel et al. 2015; Wyatt et al. 2016; Annala et al. 2018; De Laere et al. 2019; Hussain et al. 2018). However, amplifications of the whole AR locus were not associated with treatment response in two recent reports (Annala et al. 2018; De Laere et al. 2019). Collectively the data suggests that patients with TP53 mutations and/or hotspot AR mutations and/or intra-AR structural rearrangements represent an ARSi poor prognosis group of patients. The $TP53_{wt}/AR_{wt}$ biomarker signature will therefore be investigated in ProBio with a strong hypothesis that $TP53_{wt}/AR_{wt}$ patients will present longer responses to ARSi treatment. An exception will be made for patients progressing on abiraterone presenting with wild-type AR or exclusively mutations associated with abiraterone resistance (Lallous et al. 2016).

Definitions AR_{wt}:

- without any of the following somatic events:
  - High-impact structural variation or copy-number alteration affecting exonic sequence downstream of cryptic exon 4 or including cryptic exon 4 with the exonic sequences upstream of cryptic exon 4 intact *(Figure 2 - Androgen Receptor (AR) gene structure).*
  - High-impact structural variation or copy-number alteration inactivating exon 1 with the alternative first exon 1b intact.
  - Hotspot mutation associated with poor response to enzalutamide or abiraterone treatment, respectively.

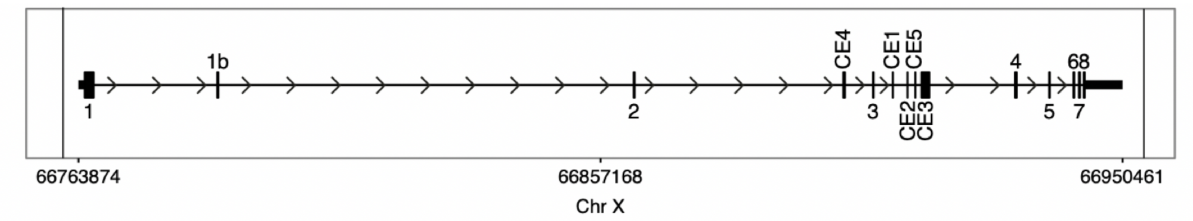

Figure 2 - Androgen Receptor (AR) gene structure (hg19), 5' to 3'-end

Definitions $TP53_{wt}$:

- without any of the following somatic events:
  - High-impact clonal structural variation affecting one or more exons.
  - Hotspot or high-impact clonal point mutation not known to be benign.
  - Homozygous deletion.

Prevalence:
50 % of mCRPC patients constitute the $TP53_{wt}/AR_{wt}$ biomarker signature (Mayrhofer et al. 2018).

2.2 TMPRSS2-ERG gene fusion (T-E_{fusion})

The TMPRSS2-ERG gene-fusion occurs in 40-50% of prostate cancer (Robinson et al. 2015; Cancer Genome Atlas Research Network ...). The addition of abiraterone (Fizazi et al. 2017; James et al. 2017), or docetaxel chemotherapy (James et al. 2016; Sweeney et al. 2015) to first line mPC treatment prolongs survival and have become standard of care world wide. Retrospective analysis in the GETUG 12 and 15 trials suggest that improvement in progression free survival is confined to the cancers harboring the TMPRSS2-ERG gene fusion (Rajpar et al. 2017), however contradictory data exist (Rescigno et al. 2018; Galletti et al. 2014). The impact of the TMPRSS2-ERG gene-fusion on chemotherapy and other treatment responses will therefore be investigated in ProBio as prospective data is needed to solidify its negative, positive or neutral association with outcomes.

Definitions T-E_{fusion}:
- detection of at least one of the following somatic events:
  - Gene fusion by structural rearrangements.
  - Gene fusion by deletion detected through copy-number alteration analysis.

Prevalence:
The TMPRSS2-ERG gene fusion was detected in 32 % of patients in in-house generated pilot data (Mayrhofer et al. 2018).

2.3 TP53-alterations ($TP53_m$)

$TP53_m$ occurs in 8% of localized prostate cancer (Cancer Genome Atlas Research Network ...) and is overrepresented in metastatic disease (Hussain et al. 2018; Mayrhofer et al. 2018; Robinson et al. 2015; Wang et al. 2017). Alterations in TP53 are associated with poor response to ARSi (Annala et al. 2018; De Laere et al. 2019). The hypothesis in ProBio is that TP53 altered mPC constitutes a poor prognosis category of patients regardless of therapy.

Definitions:
- $TP53_m$, detection of at least one of the following somatic events:
  - Clonal high-impact structural variation affecting one or more exons.
  - Clonal missense or high-impact point mutation.
  - Homozygous deletion.

Prevalence:
The $TP53_m$ biomarker signature occurs in 37 % of patients in in-house generated pilot data (Mayrhofer et al. 2018).

2.4 Homologous Recombination Deficiency (HRD)

Metastatic prostate cancer with inactivation of genes associated with with DNA repair deficiency (DRD) is sensitive to PARP inhibition (Mateo et al. 2015; Mateo et al. 2019), crosslinking chemotherapy (Pomerantz et al. 2017; Cheng et al. 2016; Beltran et al. 2015) or radium-223 (Isaacsson Velho et al. 2019). Contradicting associations between DRD and abiraterone/enzalutamide treatment were recently published (Annala et al. 2018; Hussain et al. 2018; Clarke et al. 2018; Antonarakis et al. 2018; Mateo et al. 2018). In addition, it is currently not known if inactivation of all genes associated with DRD are equally relevant in the context of various treatments. There is heterogeneity in the mutational pattern phenotype, which is associated with their different roles in the DNA-repair machinery. Therefore, it is hypothesized that DRD genes, not causing the mutational imprint of HRD, will not be sensitive to e.g. PARP inhibition (Polak et al. 2017) as recently demonstrated in the TOPARP-B trial (Mateo et al. 2019). Intriguingly, potential synergy effects of ARSi and PARP inhibition may sensitise a broader group of DRD genes (Clarke et al. 2018). This relationship awaits evaluation in prospective randomised clinical trials. In ProBio we have the hypothesis that patients with the biomarker signature HRD, encompassing a broad group of DRD genes, will demonstrate significantly prolonged responses to 1) ARSi and PARP inhibition and 2) carboplatin.

Definitions HRD:

- detection of at least one of the following somatic events in either of ATM, ATR, BARD1, BRCA1, BRCA2, BRIP1, CDK12, CHEK2, FANCA, MRE11A, NBN, PALB2, RAD50, RAD51, RAD51B, RAD51C, and RAD51D:
    - Clonal high-impact structural variation affecting one or more exons.
    - Clonal hotspot or high-impact point mutation not known to be benign.
    - Homozygous deletion.

- detection of at least one of the following germline events in either of ATM, ATR, BARD1, BRCA1, BRCA2, BRIP1, CDK12, CHEK2, FANCA, MRE11A, NBN, PALB2, RAD50, RAD51, RAD51B, RAD51C, and RAD51D:
    - High-impact structural variation affecting one or more exons.
    - High-impact point mutation not known to be benign.
    - Deletion of one allele detected by copy-number alteration analysis.

Prevalence:

Approximately 19% of CRPC harbor high-impact clonal somatic/germline alterations or homozygous deletions in genes associated with DRD in our pilot data (Mayrhofer et al. 2018).

For the HRD biomarker signature pre-specified subgroup analyses have been defined and described in *'2.4.1 Ad hoc subgroup analysis in context of the DRDm biomarker signature'*.

2.4.1 Ad hoc subgroup analysis in context of the HRD biomarker signature

Recent publications or conference abstracts (*Table 2 - Response assessment results from clinical trials with PARP inhibitors*) demonstrate that HRD genes not part of the BRCA complex are not associated with response PARP inhibition (Mateo et al. 2019; Abida et al. 2020; OncologyPRO).

Table 2 - Response assessment results from clinical trials with PARP inhibitors in metastatic castration-resistant prostate cancer, stratified by DRD genes.

		Study	TOPARP-B	TRITON2	PROfound
		Status	Published	Published	Published
		Treatment	Olaparib (PARP)	Rucaparib (PARP)	Olaparib (PARP)
		Disease and line or treatment	mCRPC, ≥2 line, ≥1 line of taxanes	mCRPC, progressed on second line	mCRPC ≥2 line
		Patient selections	Carries of DRD alterations	Carries of non-BRCA AND BRCA1/2 DRD alterations	Carries of DRD alterations
		Nbr patients	98	78	778
		Design	phase 2 trial	phase 2 trial	Randomized phase 3 trial. Controversial control arm
		Design	Randomized 1:1 to 300 or 400 mg dose	Purpose: safety and efficacy of rucaparib	Cohort A: BRCA1/2 + ATM, cohort B: Other DRD genes
		Response	Composite of radiology, PSA50 or CTC conversion	Recist, PCWG3 or PSA50	
		Biomaterial	Biopsies and germline DNA	Tissue/Plasma and germline	Tissue
		Profiling	NGS at ICR, London	Mix of in-house and commercial NGS assays	Foundation medicine
Response assessments	Composite response	BRCA1/2	25/30 (83·3%; 65·3–94·4)		Significant increase in PFS, only responses in BRCA2 (Ntot BRCA2 altered = 141 men). Ntot BRCA1 = 13
		ATM	7/19 (36·8%; 16·3–61·6)		Ntotal = 86 men, no diff to standard of care
		CDK12	5/20 (25·0%; 8·7–49·1)		Ntotal = 89 men, no diff to standard of care
		PALB2	4/7 (57·1%; 18·4–90·1)		NA
		CHEK2			Ntotal = 13 men, no diff to standard of care
		Other	4/20 (20·0%; 5·7–43·7)		Responses seen in 7/10 men with RAD51B/L alterations (belongs to BRCA complex)
	Recist	BRCA1/2	11/21 (52·4%; 29·8–74·3)	27/62 (43.5%)	
		ATM	1/12 (8·3%; 0·2–38·5)	2/19 [10.5%]	
		CDK12	0/18 (0·0%; 0–18·5†)	0/10 [0%]	
		PALB2	2/6 (33·3%; 4·3–77·7)	2/2 [100%]	
		CHEK2		1/9 [11.1%]	
		Other	0/17 (0·0%; 0–19·5†)		
	PSA 50	BRCA1/2	23/30 (76·7%; 57·7–90·1)	63/115 (54.8%)	
		ATM	1/19 (5·3%; 0·1–26·0)	2/49 [4.1%]	
		CDK12	0/20 (0·0%; 0–16·8†)	1/15 [6.7%]	
		PALB2	4/6 (66·7%; 22·3–95·7)	2/2 [100%]	
		CHEK2		2/12 [16.7%]	
		Other	2/17 (11·8%; 1·5–36·4)	1/4 FANCA, 1/2 BRIP1, 1 RAD51B	
	CTC convers	BRCA1/2	17/22 (77·3%; 54·6–92·2)		
		ATM	5/10 (50·0%; 18·7–81·3)		
		CDK12	5/12 (41·7%; 15·2–72·3)		
		PALB2	0/2 (0–84·2†)		
		Other	3/11 (27·3%; 6·0–61·0)		

However, ARSi treatment has been suggested to sensitise non-BRCA complex mCPRC to PARP inhibitors (N. Clarke et al., 2018), therefore the HRD biomarker signature will initially include all four categories of DRD genes (BRCA complex, sensing, signalling and other) (Polak et al. 2017). Nonetheless, if the effect of ARSi-introduced BRCAness is negligible and to avoid randomising to ineffective treatment, and to investigate the predictive role of individual genes on platinum sensitivity (Mota et al. 2020), the following subgroup analyses has been pre-specified:

1. **Genes associated with BRCA complex (strand invasion/repair) and non-BRCA complex, as defined below, will be continuously evaluated separately.**

- BRCA complex (strand invasion/repair)
  - BARD1, BRCA1, BRCA2, BRIP1, PALB2, RAD51, RAD51B, RAD51C, and RAD51D
  - FANCA (unclear mechanism, shown to interact with PARP1 and BRCA1)

- Non-BRCA complex
  - Sensing DNA breaks
    - ATR, MRE11A, RAD50, NBN
  - Signaling in DNA repair pathway

        - ATM, CHEK2
    - Other DRD genes
        - CDK12

2. Selected HRD-associated genes compliant with the MAGNITUDE (NCT03748641) and AMPLITUDE (NCT04497844) RCTs will be continuously evaluated separately.

- BRCA1, BRCA2, BRIP1, CDK12, CHEK2, FANCA, PALB2, RAD51B and RAD54L
    - RAD54L is not covered by the ProBio panel (see *'Figure 1 - ProBio assay comprehensive design overview'*) and will be incorporated in future updates of the ProBio panel design and upon protocol amendment.

Evaluation will be done on a monthly basis, simultaneously as updating the randomisation probabilities. Any indication of differential response in the experimental arms between BRCA complex and non-BRCA complex genes vs. the control arm will be presented to the DSMB for advice if the HRD biomarker signature should be kept intact or split in two- or more biomarker signatures.

3. FUTURE PRESPECIFIED BIOMARKER SIGNATURES FOR RANDOMISATION UPON AVAILABILITY OF NEW IMP

3.1 Tumor mutational burden high, microsatellite instability or CDK12 inactivation (TMB-H/MSI+/CDK12-)

Immunomodulation by targeting programmed cell death protein 1 (PD-1), programmed death-ligand 1 (PD-L1), or cytotoxic T-lymphocyte-associated protein 4 (CTLA-4) has demonstrated promising results in multiple advanced cancers. Several molecular biomarkers have been proposed with predictive potential, encompassing, but not limited to, microsatellite instability (MSI+) (Le et al. 2017; Asaoka et al. 2015; Abida et al. 2018), mismatch-repair deficiency (MMR) (Chung et al. 2019) , CDK12 inactivation (CDK12-) (Wu et al. 2018), high tumor mutation burden (TMB-H) and perturbation of DNA polymerase epsilon (*POLE*) and delta 1 (*POLD1*) proof-reading genes (Lee et al. 2018). The common denominator for these molecular biomarkers is an increase in neoantigens on the cell surface which facilitates a strong immune response towards the cancer cells in the context of check-point inhibitor therapy. We demonstrated for the first time that the MSI+ phenotype and an elevated tumor burden may be detected directly by ctDNA profiling (Mayrhofer et al. 2018), later replicated by others (Willis et al. 2019). Approximately 10 % of metastatic prostate cancers harbor somatic alterations associated with an increased load of neoantigens on the cell-surface associated with response to immunotherapy (Wu et al. 2018).

Definitions TMB-H/MSI+/CDK12-:

- TMB-H, detection of:
    - ≥20 mutations per megabase of sequence
- MSI$_+$, detection in patients with ≥0.10 ctDNA fraction:
    - ≥0.10 unstable microsatellites

- CDK12-, detection of:
    - clonal biallelic inactivation of CDK12
    - clonal monoallelic inactivation of CDK12 and an observed increase in tandem focal duplications

Prevalence:
The TMB-H/MSI+/CDK12- biomarker signature constitutes approximately 10% of mCRPC cases (Wu et al. 2018).

3.2 PI3K perturbed

Dysregulation of the phosphatidylinositol 3-kinase (PI3K) pathway is prevalent in metastatic prostate cancer; with ca. 40% of tumours having an inactivation of the *PTEN* tumour suppressor gene. Other mechanisms of PI3K pathway activation include among other deletions of the regulatory unit *PIK3R1,* as well as activating hotspot mutations or high level amplifications in oncogenes, such as *PIK3CA, PIK3CB* and *AKT1* (Chung et al. 2019). Clinical effectiveness of PI3K inhibitors in advanced breast cancer has been demonstrated in patients harbouring hotspot *PIK3CA* mutations (André et al. 2019). In prostate cancer, pan-AKT inhibition has demonstrated promising results in phase 2 (de Bono et al. 2019) and phase 3 (OncologyPRO) clinical trials in mCRPC patients with PTEN loss.

In addition, it is currently not known if activation or inactivation of all genes associated with the PI3K pathway are equally relevant in the context of various treatments. Recently the potential of activating AKT1/PIK3CA mutations as biomarkers for PI3K pathway inhibitor sensitivity in mCRPC has been reported (Herberts et al. 2020). This warrants prospective evaluation testing PI3K pathway inhibitors in this population. In ProBio we have the hypothesis that patients with a perturbed PI3K signalling pathway will demonstrate significantly prolonged responses to 1) ARSi and AKTi inhibition or 2) taxane-based chemotherapy and AKTi inhibition, dependent on which systemic drug classes the patient previously received.

Definitions PI3K perturbed:

- detection of at least one of the following somatic events in PTEN or PIK3R1:
    - Clonal high-impact structural variation affecting one or more exons.
    - Clonal hotspot or high-impact loss-of-function point or frameshift mutation not known to be benign.
    - Homozygous deletion.

- detection of at least one of the following somatic events in PIK3CA, PIK3CB or AKT1:
    - Clonal activating hotspot mutation
    - Focal amplification

Prevalence:
Approximately 40% of CRPC harbor high-impact clonal somatic alterations or homozygous deletions in genes associated with the PI3K pathway in our pilot data (Mayrhofer et al. 2018).

SMART-TRIAL eCRF STANDARD OPERATING PROCEDURES

Trial title:

ProBio:
An outcome-adaptive and randomised multi-arm biomarker driven study in patients with metastatic prostate cancer

Coordinating Investigator and Sponsor's representative

Henrik Grönberg
Karolinska Institutet

TABLE OF CONTENTS

TABLE OF CONTENTS 2
1. PURPOSE **Error! Bookmark not defined.**
2. PROCESS 3
2.1 Side menu 3
2.2 Subjects 3
2.2.1 Enrolment 3
2.2.2 Discontinue subjects and re-enrol subjects 4
2.2.2.1 Discontinuation 4
2.2.2.2 Re-enrolment 6
2.2.3 Sign off subjects 7
2.2.4 Exclude subjects 7
2.3 Study Overview and Data input 7
2.3.1 Main Site Overview 7
2.3.2 AE/SAE and Medication notification 9
2.3.3 Locking Data Events – Freeze Answers 9
2.3.4 Collecting data – Subjects (Not applicable in ProBio) 10
2.3.5 Collecting data – Healthcare Professionals 10
2.3.6 Jumping between forms in a visit event 10
2.3.7 Question Comments during visit events 11
2.3.8 Remove selected answer 11
2.3.9 Mark mandatory question answer as not available 11
2.3.10 Review subject/visit forms data 11
2.3.11 Change/edit answers/data 12
2.3.12 Overview of subjects and subject detailed information 12
2.4 Medication and medication accounting 14
2.4.1 Register IMP dose and concomitant medication 14
2.4.2 IMP accounting (not used in ProBio in protocol version 3.0) 15
2.5 Discontinued events 16
2.6 Unscheduled events 16
2.7 Adverse Events / Serious Adverse Events and Serious Adverse Reaction 17
2.7.1 Instructions for reporting AE/SAE and SAR 19
3. OVERVIEW OF SMART-TRIAL DATA EVENTS FORMS DURING PROBIO 20
3.1 Data events during ProBio-mHSPC 21
3.2 Data events during ProBio-mCRPC 22
3.2.1 Data event: Inclusion (I) 22
3.2.2 Data event: Baseline Assessment (BA) 23
3.2.3 Data event: 0 Month (0M) – Treatment start 23
3.2.4 Data event: 1 Month follow-up (1M) 23
3.2.5 Data event during first 24 weeks: every 8 weeks, i.e. 2M, 4M & 6M 23
3.2.6 Data event after the first 24 weeks: every 12 weeks until progression, i.e. 9M, 12M, 15M etc. 24
3.2.7 Discontinuation of subject at a scheduled Data event 24
3.2.8 Discontinuation as an unscheduled event visit 24
3.2.9 Adverse event/ Serious Adverse event reporting 24

1. PURPOSE

The purpose of this document is to clarify the standard operation procedures for SMART-TRIAL in ProBio. SMART-TRIAL shall be used to both collect and store, all research related data for ProBio. Data is collected through the secure user interface of SMART-TRIAL (https://app.smart-trial.co) from both healthcare professionals and subjects. Additional information and how-to's can be found on the SMART-TRIAL help site: https://help.smart-trial.co/

2. PROCESS

2.1 Side menu

The side menu is an integral part of SMART-TRIAL, it is here you will find most short cuts needed - the most important for data collection being "Site Overview" and specific sites. Please see *Figure 1*

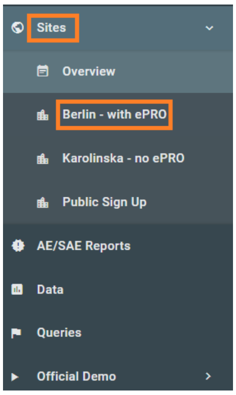

Figure 1

Depending on your access to the study, you can see one, some or all sites in the study. Whenever there is referred to accessing the site overview in this SOP, it is done as shown on *Figure 1.*

2.2 Subjects

2.2.1 Enrolment

When a subject is to be enrolled, a subject-profile must be created within SMART-TRIAL. This can be done in the site overview. The "site overview" can be accessed from the side menu (see *Figure 1*) by clicking "Sites", and selecting one of your sites. In the site overview you will see the "New Subject" button (see *Figure 2*)

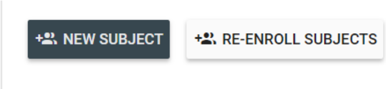

Figure 2

To enroll a subject, click on the "New Subject" button. From there, you can directly enroll subjects-profiles within a site, or enroll already created subject-profiles (if applicable).
This will bring up the window seen in *Figure 3*, these field will change depending on what attributes are required for the study.

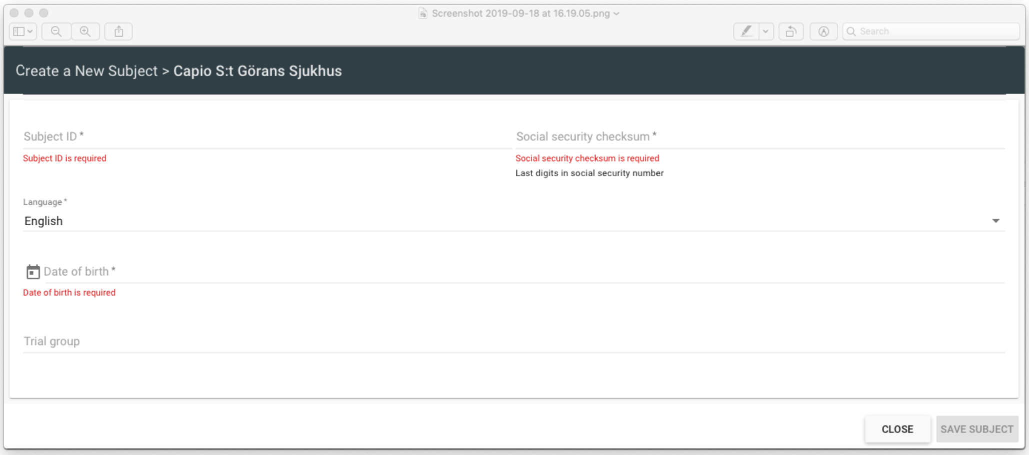

Figure 3

Subject Id, Date of birth are mandatory to fill out. Optional subject profile attribute is the Social Security Number Checksum (last 4 digits of the "personnummer" in Sweden) and the Trial Group, which may be entered after the subject has been randomized to either Standard of Care or Experimental arm. Subject ID should adhere to the following convention:

Sites in Sweden	Site ID	Subject ID start number	Subject ID end number
Capio S:t Görans sjukhus	SG	SG1001	SG1099
Karolinska Universitetssjukhuset	KS	KS1101	KS1199
Akademiska sjukhuset	AK	AK1201	AK1299
Norrlands Universitetssjukhus	NU	NU1301	NU1399
Skånes Universitetssjukhus	SK	SK1501	SK1599
Länssjukhuset i Kalmar	KAL	KAL1601	KAL1699
Länssjukhuset i Ryhov	RY	RY1701	RY1799
Universitetssjukhuset i Örebro	UO	UO1801	UO1899
Sahlgrenska Universitetssjukhuset	SA	SA1901	SA1999
Länssjukhuset i Sundsvall	SU	SU2001	SU2099
Helsingborgs lasarett	HE	HE2101	HE2199
Centrallasarettet i Växjö	VX	VX2201	VX2299
Universitetssjukhuset Linköping	LIN	LIN2301	LIN2399
Centralsjukhuset Karlstad	KAR	KAR2401	KAR2499

Sites in Belgium	Site ID	Subject ID start number	Subject ID end number
University Hospital, Gent	UZG	UZG3001	UZG3099
AZ Sint-Jan, Brugge	AZSJ	AZSJ3101	AZSJ3199
AZ Nikolaas, Sint-Niklaas	NIKO	NIKO3201	NIKO3299
Jessa Ziekenhuis, Hasselt	JESS	JESS3301	JESS3399
Ziekenhuis Oost-Limburg, Genk	ZOLG	ZOLG3401	ZOLG3499
AZ Groeninge, Kortrijk	AZGR	AZGR3501	AZGR3599
AZ Sint-Lucas, Brugge	AZSLB	AZSLB3601	AZSLB3699
CHU de Liège, Liège	CHUL	CHUL3701	CHUL3799
AZ Damiaan, Oostende	DAMO	DAMO3801	DAMO3899
AZ Sint Lucas, Gent	SLG	SLG3901	SLG3999
Onze-Lieve-Vrouwziekenhuis, Aalst	OLVA	OLVA4001	OLVA4099
Jan Palfijn, Gent	JAPA	JAPA4101	JAPA4199

2.2.2 Discontinue subjects and re-enrol subjects

2.2.2.1 Discontinuation

Throughout the course of the ProBio trial, subjects may experience the primary endpoint (i.e. no longer clinical benefit; NLCB), causing the subject to be either re-randomised or exit the ProBio trial. In general, the occurrence of the NLCB endpoint can occur at different timepoints, either:

- At a scheduled data event visit

  or

- Or in between data event visits (i.e. unscheduled event)

In case the NLCB is reached **<u>during a scheduled data event</u>**, the clinical trial nurse or physician completes the standard therapy evaluation form, which in case of NLCB will ask to initiate the Discontinuation event form (*Figure 4*).

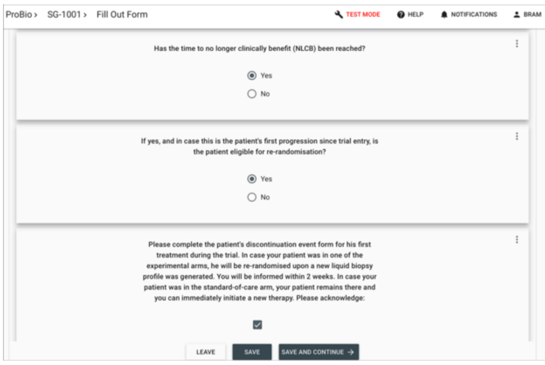

Figure 4

To discontinue a subject, navigate to the site where the subject is enrolled, as shown in *Figure 5*, and click on "Discontinue". This will bring up a box, where you can choose which subjects to discontinue.
The discontinuation form will be shown – fill them out in accordance with your study protocol.

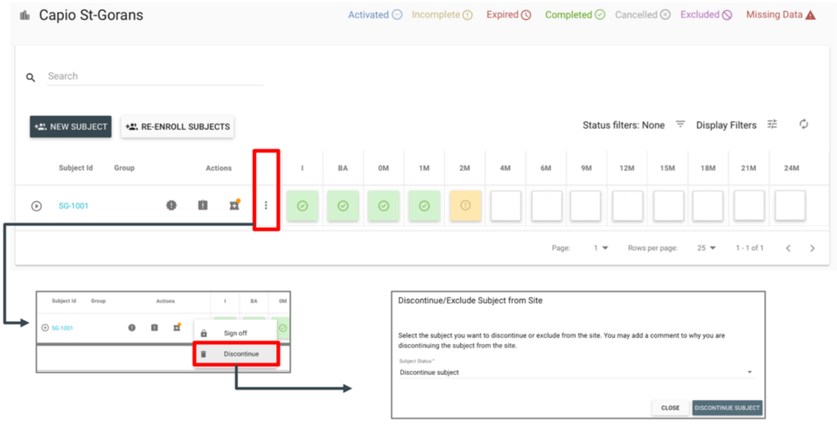

Figure 5

However, NLCB might also be reached **<u>in between data event visits</u>**. In this case, an unscheduled event must first be registered (*Figure 6*), which will activate the same standard data event forms as if it was a regular data event visit. Upon completion, the eCRF system will ask to initiate and complete the Discontinuation event form, as depicted in *Figure 4 & 5*.

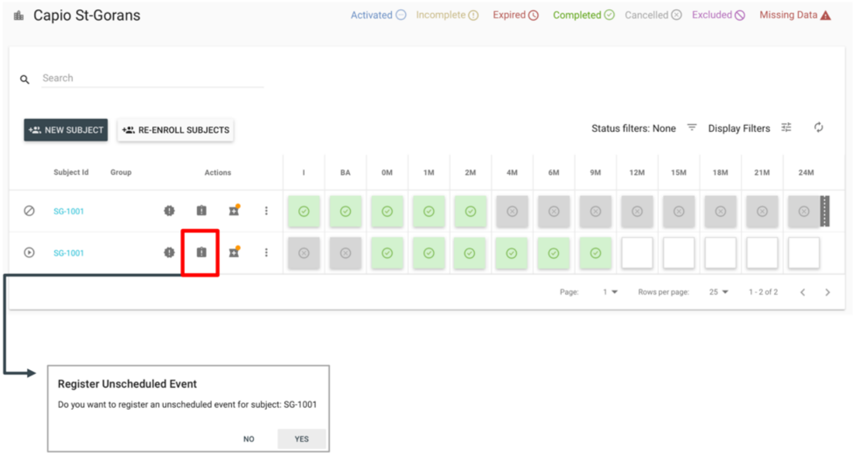

Figure 6

NOTE: Should you discontinue a subject, all data that has been collected before discontinuing a subject is still stored and will be available when data is exported. All completed data events can be seen by changing the active filter in the site overview, see *Figure 5*, to "Cancelled".

2.2.2.2 Re-enrolment

The ProBio trial monitors the subject during several lines of systemic therapy. Thus, in case the NLCB endpoint has been reached, eligible subjects will remain in the study and will be re-enrolled for re-randomisation. The clinical trial nurse or physician should re-enroll a discontinued subject (i.e. after his first randomisation), by clicking "Re-Enroll Subject" and selecting the particular patient, see *Figure 7A*.

A new process for that patient will be generated, after which the I and BA data event may be skipped by immediately clicking and starting the "0M" Data event and selecting the option 'Disable prior uncompleted data events' (*Figure 7B*). In case the patient was initially randomised to the standard-of-care (SOC) arm, the patient will remain there, which allows the clinical trial nurse or physician to immediately complete the "0M" Data event form. When the patient was randomised to one of the experimental (i.e. biomarker signature-therapy combination) arms, the results of the new liquid biopsy profile and re-randomisation need to be awaited prior to completing the "0M" Data event form.

A) **B)**

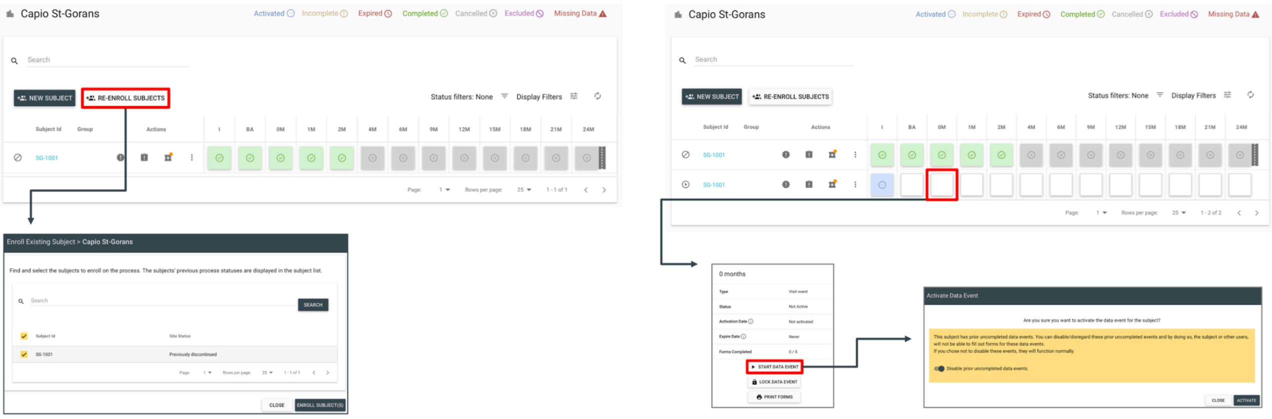

Figure 7

2.2.3 Sign off subjects

All events for a single subject can be signed off from the Site Overview Menu. To sign off a subject, go to the Site Overview Menu and under the “Actions” menu, press the three dots and a new menu will appear, click on the “Sign Off” and enter your SMART-TRIAL password. This will sign off the subject. Once the subject is signed off, a lock icon together with the date and name of collaborator who signed the subject off, will appear under the Subject id, and the data events will be locked, see *Figure 8*.

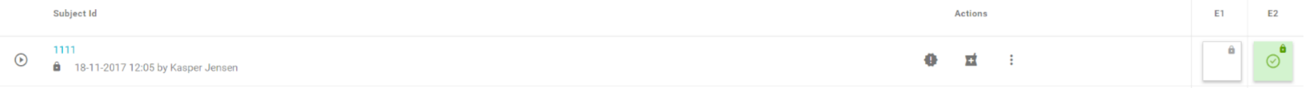

Figure 8

NOTE: Sign-off requires a special permission.

2.2.4 Exclude subjects

Subjects can get excluded if they do not comply with the inclusion/exclusion criteria. Additionally, Subjects need to be excluded when no biomarker signature can be inferred. This will be communicated to the recruiting centre by the ProBio study team. The exclude a patient, following procedure (*Figure 9*) needs to be performed:

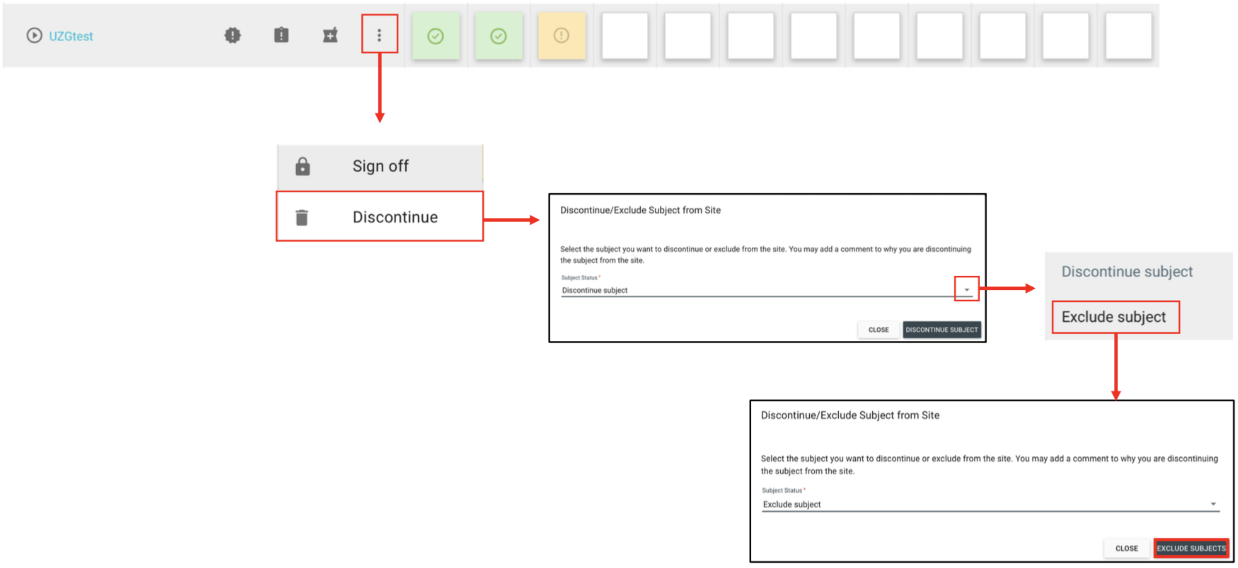

Figure 9

2.3 Study Overview and Data input

2.3.1 Main Site Overview

Users responsible for reviewing subject status, or completing visit forms (case report forms), can access individual subject status for each site, by accessing the “Site-name” sub-menu under “Sites” in the left side menu, as seen on *Figure 1*.

In the site overview, you are shown a list of all subjects enrolled to the site and individual “Data events” are shown for each subject as colored boxes. The status of each subject is represented by coloring the box. By clicking onto the individual box, you can view detailed information for each event/visit, fill out visit forms, print out visit forms, fill out forms for subjects (if required), or re-send forms to subjects.

To the top right (see *Figure 10*) you have a filter selector, which allows you to filter subjects after their status. This enables you to get a quick overview e.g. of all excluded subjects, or all completed subjects. By default, this filter is not on, which gives you an overview of all subjects.

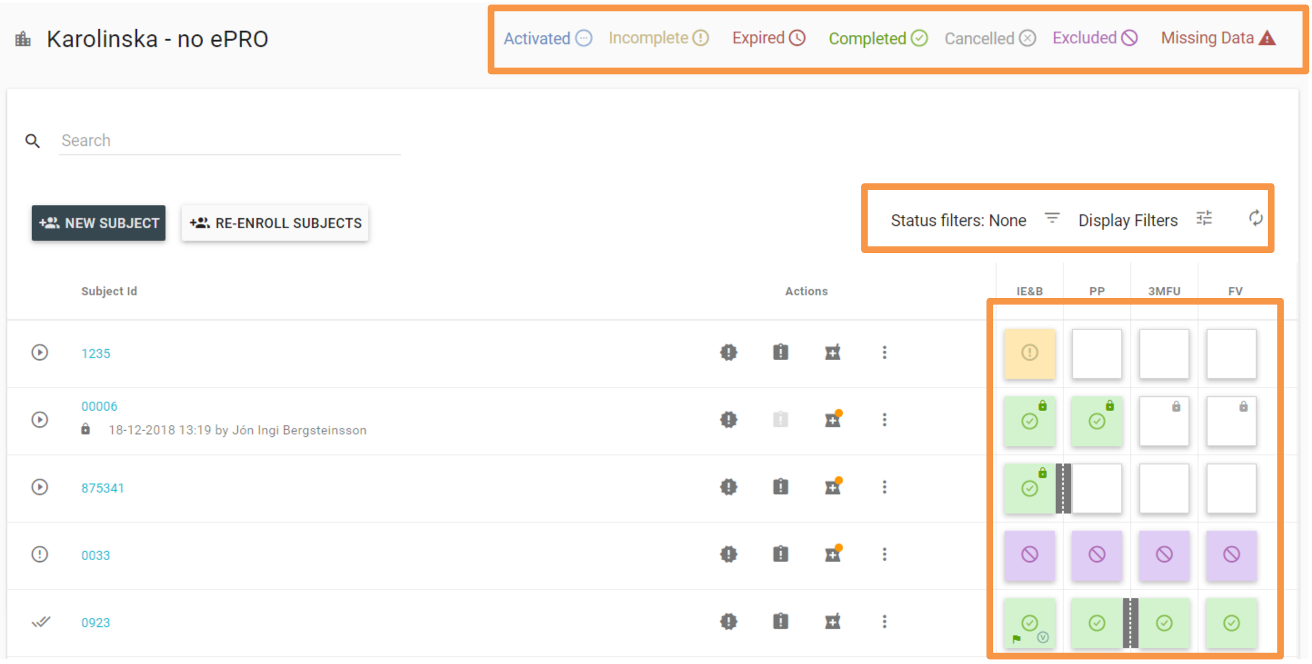

Figure 10

The different color codes, lets you know if a specific data event is either Activated, Incomplete or etc. (See *Figure 10*) Use this color or the filter to get a better overview of your data collection.

If a data event is Green, the data event is completed, and all mandatory data points have been answered in the data event.

If the field is Red, the data event has expired, because the deadline for the data event was exceeded.

Blue means that the data event has been activated and its possible to fill out the forms in the event.

If the data event is orange the data event is incomplete, this can either be due to missing information or because the subject has left some of the questions in the form unanswered.

Purple means that the subject and their data event has been excluded from the study, already collected data is **not** lost.

Under "Display Filters" you can customize the site overview to your liking, the customization is available until logout.
You can select which subject attributes you wish to see, e.g. if email, name and subject id is collected, you can choose to only have subject id shown. Likewise, you can choose which status icons you wish to see on the data events, e.g. if you are interested in seeing where there are open queries, you can select only the query icon.

2.3.2 AE/SAE and Medication notification

From the site overview you are able to access the subject's AE/SAE Reports, Medication registration, or Medication Accounting. When an AE/SAE Report or Medication reports are available an orange dot will appear together with the icon in the list (See *Figure 11*).

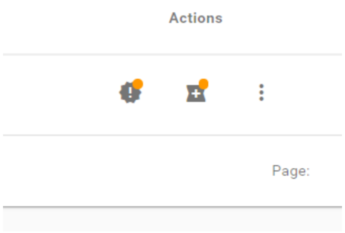

Figure 11

2.3.3 Locking Data Events – Freeze Answers

It's possible to lock (freeze data) for individual data events. You can lock the data event at any time in the study. When a data event is locked, a lock icon will appear in the top right corner of the event (see *Figure 12*). To lock a data event click on the specific data event and press "Lock Data Event", see *Figure 12*.

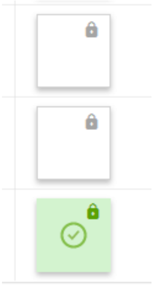

Figure 12

NOTE: The data event can be unlocked again if necessary.
NOTE: To lock and unlock data events, users require specific lock and unlock permission.

2.3.4 Collecting data – Subjects (Not applicable in ProBio)

If a process includes subject data events, i.e. where subjects are required to answer specific forms, they will either receive an e-mail or SMS with a unique link which can be used to fill out the forms. Each subject must open this link in a web browser (on a computer, or a mobile device) to complete the forms required for the specific event. No actions are required by users, as subjects should be able to complete these forms for themselves.

NOTE: if required, users with the right permissions, can fill out subject events for subjects who are not able to open the links or complete the forms by themselves. This can be done by accessing the unique link via the "site overview" by clicking on the specific subject event square (see section *'2.3.1 Main Site Overview'*). This is action is always logged, and can therefore be traced back to the user if done.

2.3.5 Collecting data – Healthcare Professionals

When you are to fill out a form during a visit event, you must do this from the site overview (see *'2.3.1 Main Site Overview'*). Find the relevant subject, and click the visit event square, you will then be presented with a menu from the right. Using this menu, you can 'Start Data Event', as seen on *Figure 13*.

NOTE: If the data event has been started, you can click on "fill out visit forms" to collect data.

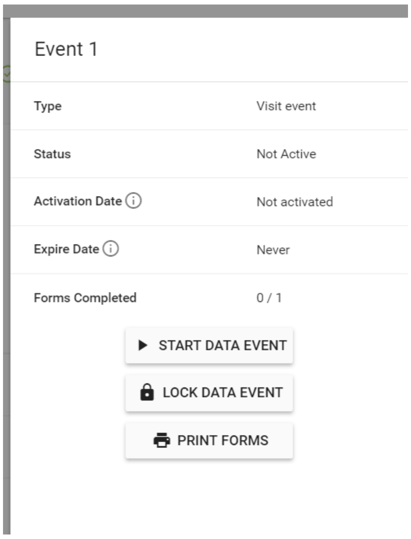

Figure 13

2.3.6 Jumping between forms in a visit event

When filling out forms during a visit event, it's possible to navigate between the different forms associated with the data event, should a form fit better with the work flow. This means you can jump between forms and fill them out at in an order of your choosing.

NOTE: You must click "Update and continue" to save any data input, if not, data will be lost.

2.3.7 Question Comments during visit events

When inputting data to a form (as a visit form), users can add individual comments to each question answer by clicking the menu button icon to the right of each question (see *Figure 14*). Here you can note anything that could be of relevance for the data input.

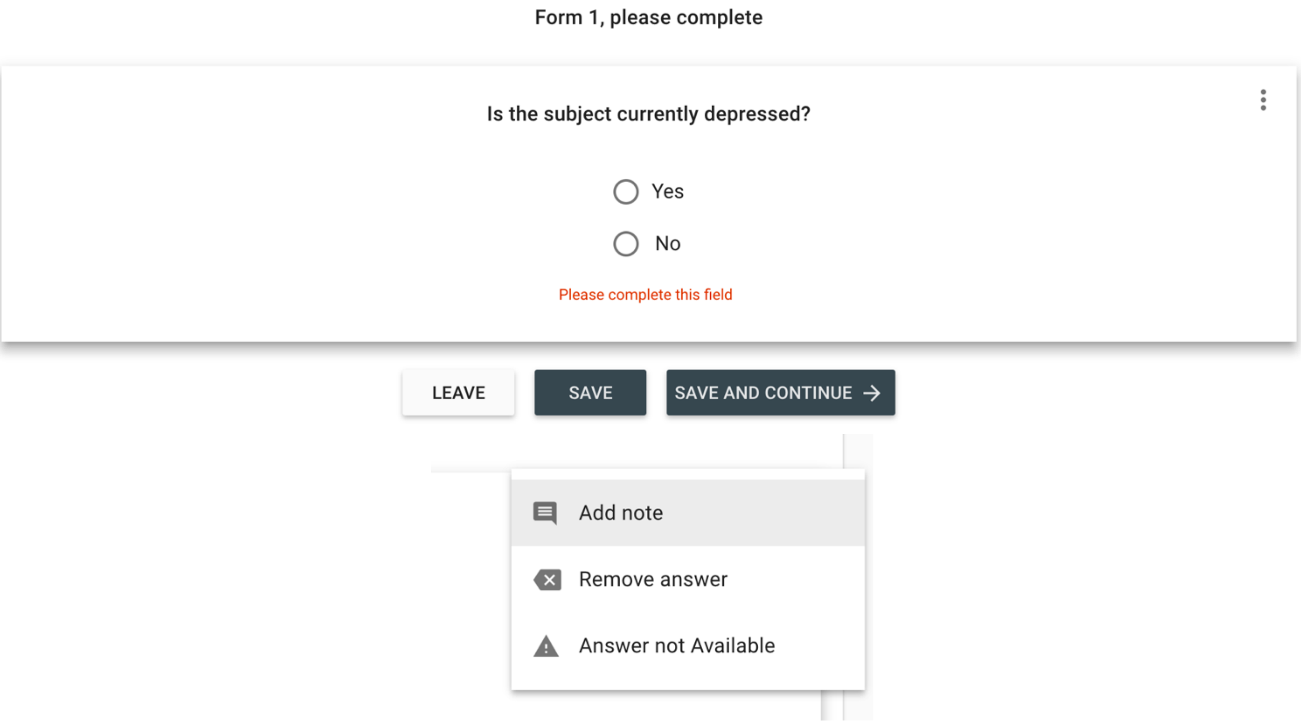

Figure 14

2.3.8 Remove selected answer

If you need to remove a selected answer, e.g. if you have selected a choice from a multiple-choice question, you can remove your answer by pressing the question "menu button" to the top right (see *Figure 14*) and select "Remove Answer"

2.3.9 Mark mandatory question answer as not available

If you need to mark a mandatory question as "Not available" or as missing data, you can press the question "menu button" in the top right (See *Figure 14*) and select "Answer not available"

2.3.10 Review subject/visit forms data

For reviewing form answers you can access completed forms for individuals via the site overview by clicking the relevant data event squares and choose "View Form Answers" from the side menu (see *Figure 15*)

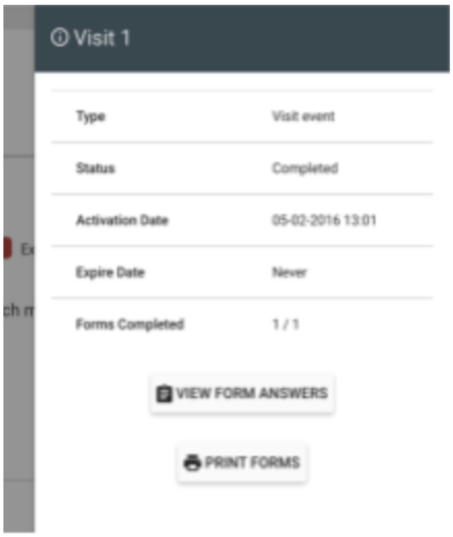

Figure 15

2.3.11 Change/edit answers/data

If data must be changed for an individual subject, this can be done while reviewing forms.
While in the review data view, if you have sufficient permissions, you are able to edit the data within the form, by clicking "Edit Answers" to the top left of the form (see *Figure 16*).

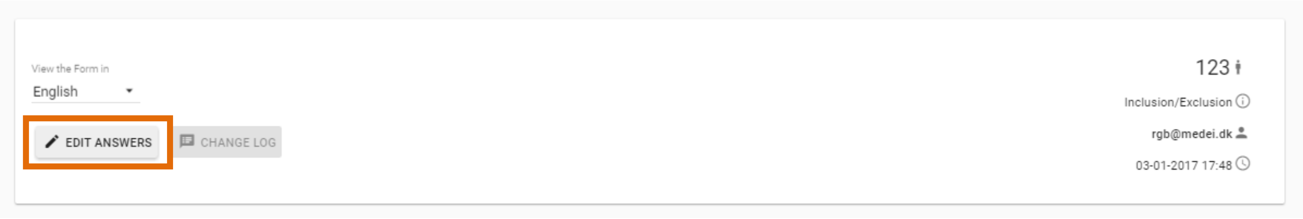

Figure 16

This button enables input change in every field of the form. When you have changed the relevant input data fields, you must click the "Update" button in the top or bottom of the form. This requires you to submit a "reason for change", where you shall register the reason for why data change was performed.
Should you regret any changes you have made, you can click the "Discard Changes" button, located same place as "Edit Answers".

NOTE: any changes made to data or any study specific information is registered in the audit log of the study.

2.3.12 Overview of subjects and subject detailed information

From within the individual site overviews, you can access detailed information about each subject by clicking on the identifier hyperlink (see *Figure 17*).

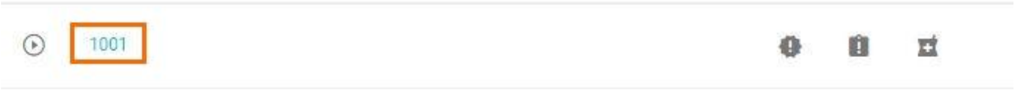

Figure 17

Here you can access: detailed profile information, overview of their data collection history, medication (concomitant and IMP), medication accounting overview and any AE/SAE reports created for the subject. You can access any of these by clicking the tabs in as shown on the figure below.

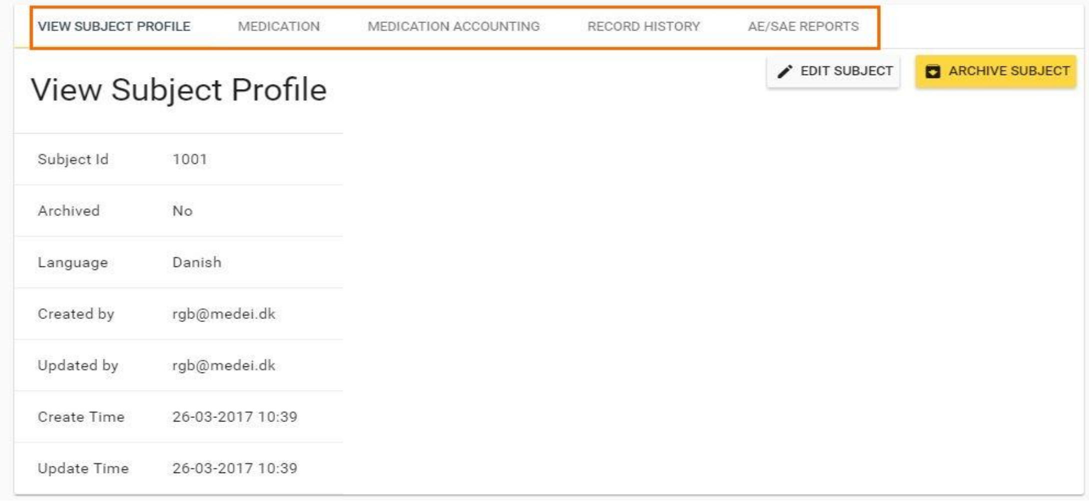

Figure 18

By clicking on "Record History", you can gain access to a complete overview of all data collection events for the specific subject.

From there, you can review each form answer by clicking the action button to the far right in the list (see *Figure 19*). where you can review each form for every specific data event (See *Figure 20*)

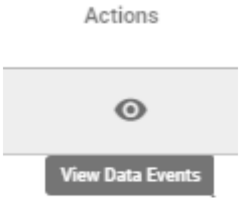

Figure 19

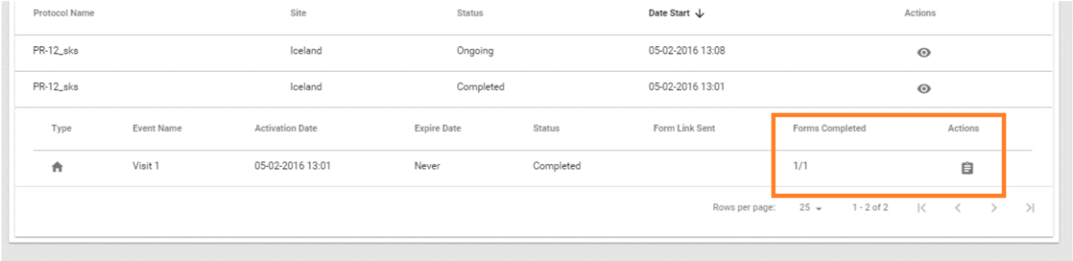

c

2.4 Medication and medication accounting

2.4.1 Register IMP dose and concomitant medication

If you are to register IMP dose or concomitant medication, this can be done from within individual subject profiles. You can access the medication view from the "Site Overview" by clicking the medication button (see *Figure 21*) or by viewing the individual subject profile as done in section *'2.3.12 Overview of subject'*.

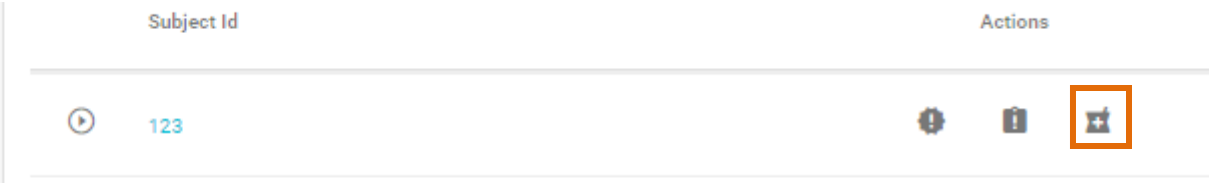

Figure 21

From the medication overview (see *Figure 22*) you can click "Add medication" to add a medication entry to the list, which can be either an IMP dosage entry or concomitant medication entry.

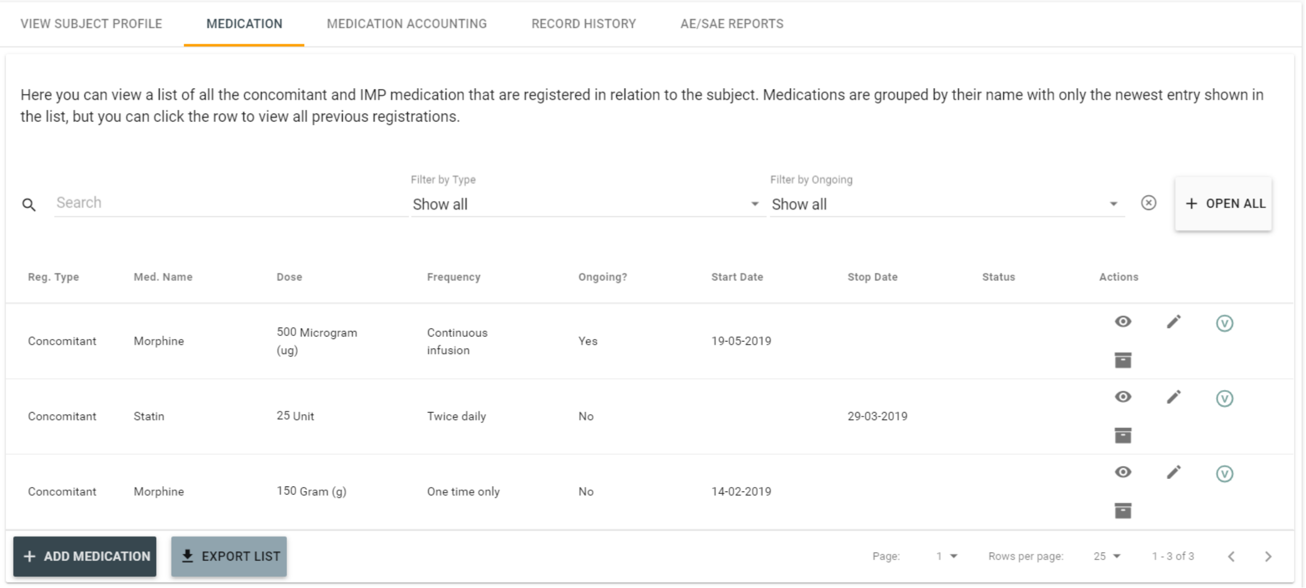

Figure 22

When adding a new medication entry to the list you must specify if this is an IMP or concomitant medicine registration. Afterwards you must input: the required fields (such as name of the medication, dose, unit, if ongoing etc., as seen on *Figure 23*.

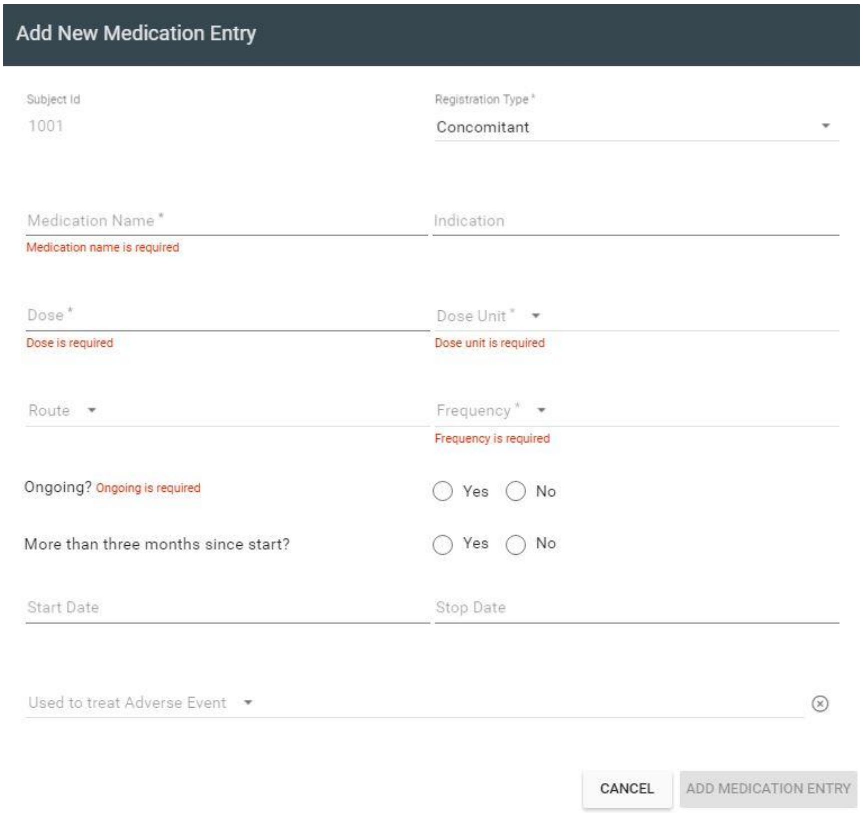

Figure 23

If multiple entries have been registered for the same medication, you can review the previous entries by clicking the individual medication entry rows in the list, this will present (see *Figure 24*). Individual medication entries can also be updated by clicking the action button to the far right in the list.

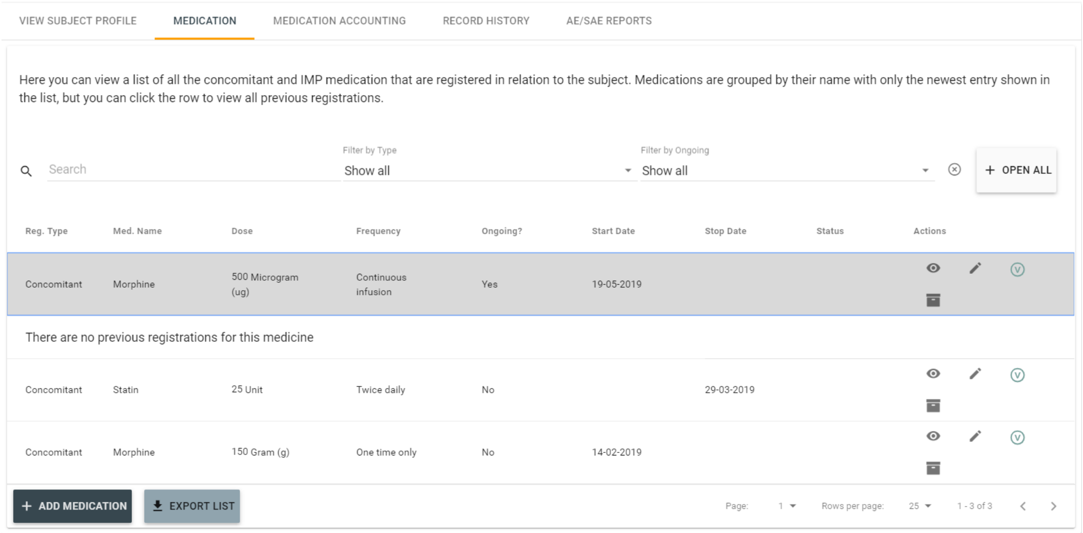

Figure 24

2.4.2 IMP accounting (not used in ProBio in protocol version 3.0)

IMP accounting can be accessed from the subject profile (see *Figure 25*) by clicking on the "Medication Accounting" tab, see below.

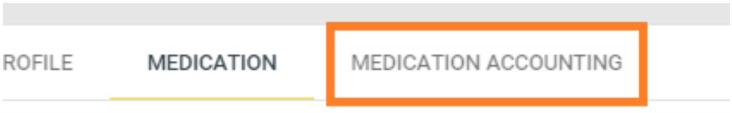

Figure 25

From the medication accounting view, you can handle all IMP accounting for individual subjects by clicking the “Register Medication”. This will show a dialog where you can specify the registration type (lost, returned, or delivered), medication name, the amount, batch number, and package type. The list overview will give you a quick indicator of all IMP accounting entries.

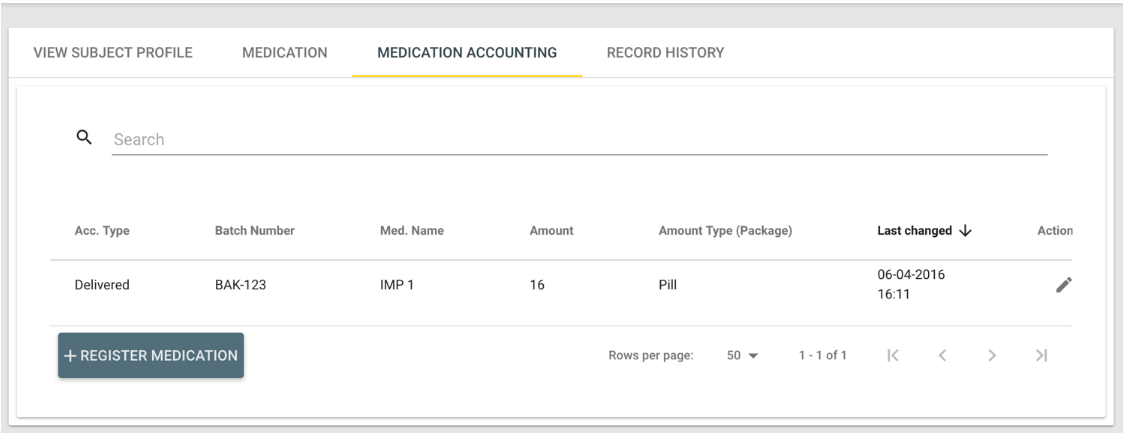

Figure 26

2.5 Discontinued events

If discontinued events are supported in the study, a form will appear when a subject is “Discontinued”. This will cancel all uncompleted data events, and mark the subject’s enrolment status as discontinued (the icon in the left side of the subject identifier will change), see *Figure 27*.
To review the Discontinue forms, go to the subject profile and find the forms under the “Record History”, see *Figure 26*.

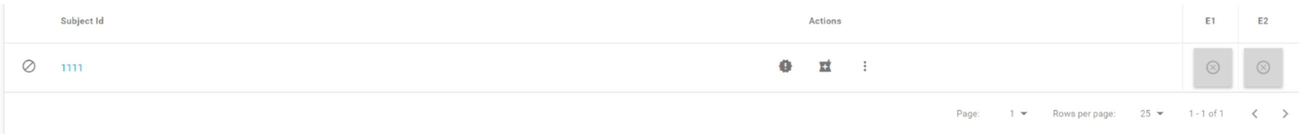

Figure 27

NOTE: You can still access the data from filled-out data events and review answers.

2.6 Unscheduled events

If process for a site supports unscheduled events, an additional icon will appear under the Actions menu in site overview, see *Figure 27*. From here unscheduled events can be registered for individual subjects.

Unscheduled event shall be registered in case progressive disease "occurs" in-between scheduled follow-up visits and subject needs to be discontinued.

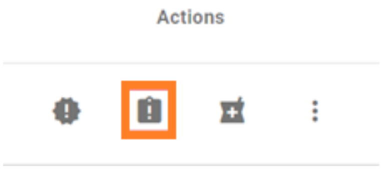

Figure 28

From a dialogue box will pop up the forms to fill out during the unscheduled event, i.e. the Physical examination form and the Treatment response evaluation form.

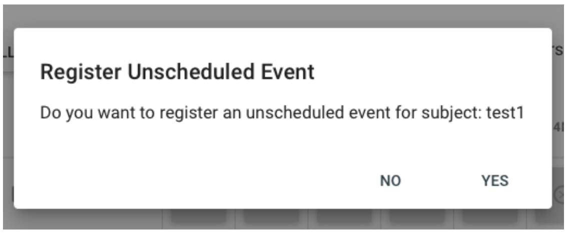

Figure 29

All unscheduled visits can be reviewed from the Site Overview menu where a grey marking will appear once a unscheduled events has been filled out, see *Figure 30*.

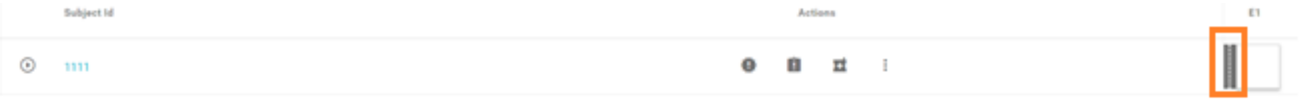

Figure 30

When clicking the grey marking, a menu will appear with the registration time, status and who registered the event and allows you to see the form and lock the event, see *Figure 31*.

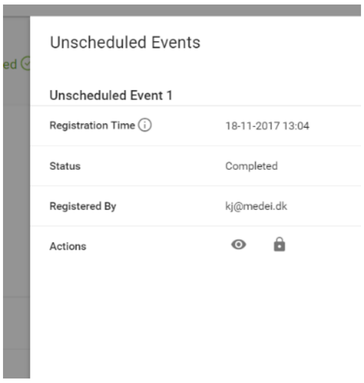

Figure 31

2.7 Adverse Events / Serious Adverse Events and Serious Adverse Reaction

All collaborators who have permission can report Adverse Events for subjects. From the site overview, you can click the “View Adverse Event report” button from the action column for individual subjects (see *Figure 32*).

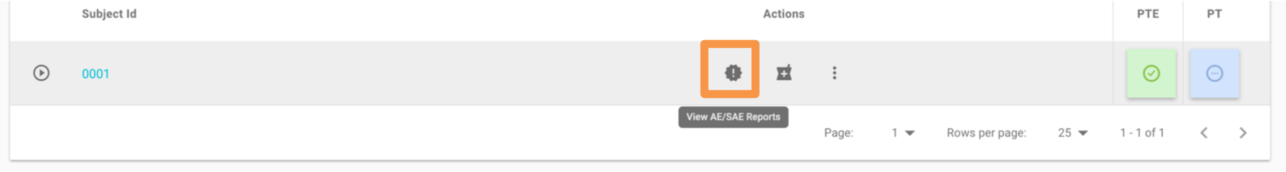

Figure 32

From there, you can click the “New AE/SAE Report” button to create a new report.

The AE/SAE form is quite similar to a paper version, in what it contains (see *Figure 33*). General information fields can be filled in by everyone when reporting an AE/SAE. For classification, restricted fields require a user with medical doctor SG(MD)/investigator privileges to fill out classifying values and sign. If SAE requires sponsor evaluation, a user with sponsor permissions can complete those fields, and sign sponsor evaluation and follow up.

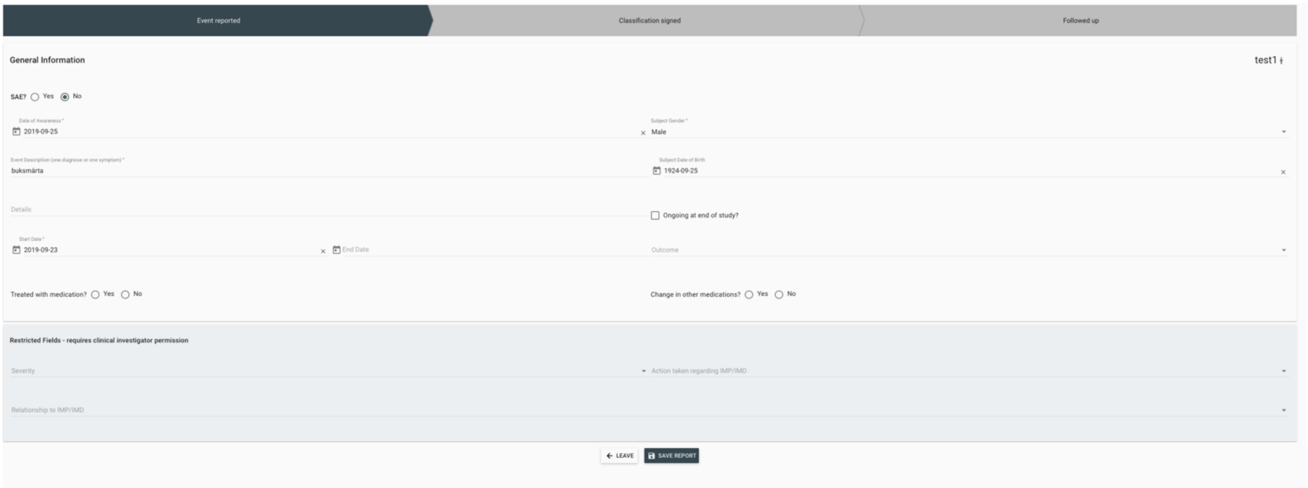

Figure 33

To view ALL AE/SAE use the left side menu and click on “AE/SAE Reports” (see *Figure 34*). You have the option to filter by site in the top right corner. “Status” on *Figure 34* shows how far along the report is. To access the AE/SAE click on the action button.

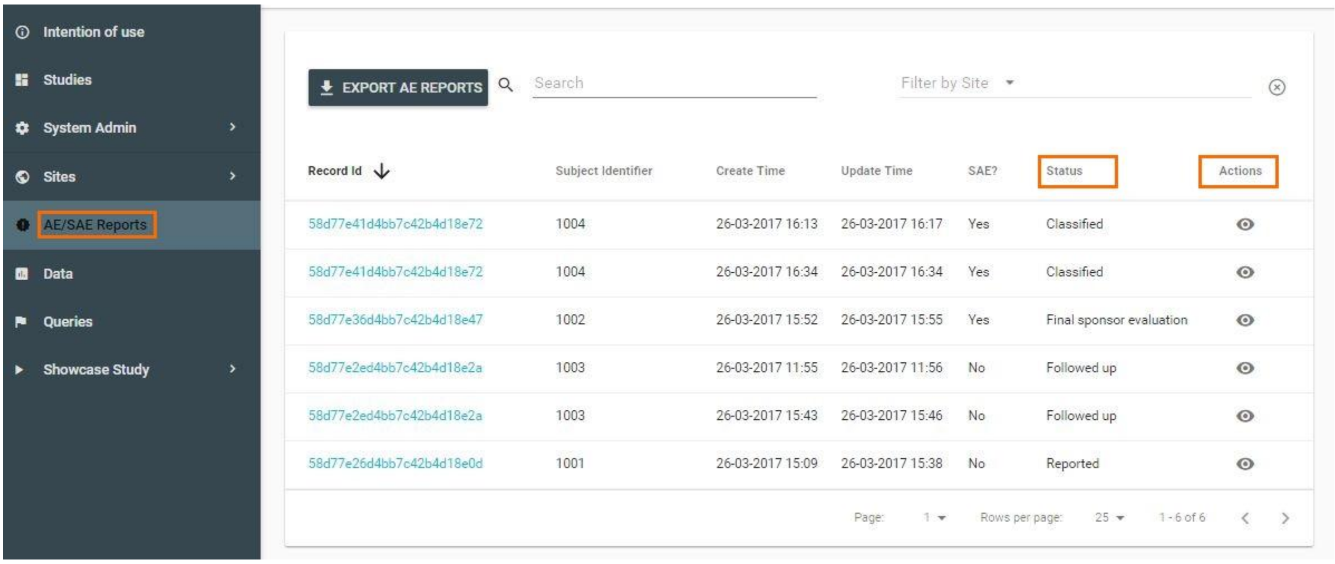

Figure 34

An AE is filled out in 3 steps (requires “SAE?” to be marked NO):

1) A user reports an AE by filling in the general information fields.
    a. Should it be a user with investigator/MD permissions the restricted fields can also be filled in at this time.
2) Users with **investigator/MD permissions** are notified and must sign the report to classify it.
    a. **NOTE**: This requires the user to input their password.
3) To follow up on the report a user must edit the fields, to ensure STOP DATE is completed. Save the report and then it can be signed as “Followed up”.

A SAE is filled out in 5 steps (requires “SAE?” to be marked YES).

1) An initial report is filled out by a user, by filling in the general information fields and marking the report as an SAE.
2) Users with **investigator/MD permissions** are notified and must sign the report to classify it. By signing the report, the investigator/MD accepts the report as correct.
    a. They should fill in the restricted fields if possible, before signing
    b. **NOTE**: This requires the user to input their password
3) Sponsor is notified about the SAE, and is required to look through the SAE report, complete sponsor evaluation and sign it.
4) A STOP DATE is added by a user and an investigator/MD can sign the SAE as followed up.
5) Sponsor is notified about the follow up and does a final evaluation of the report. Once sponsor is satisfied with the final report, it shall be signed off.

SAR (SUSAR)

Incase “Relationship to IMP/Device” is marked as **possible, probable** or **related** a SAR form will be presented in the bottom of the report, where the user should elaborate on the relationship to IMP/IMD and the action taken.

NOTE: when an AE/SAE report has been created, you can link concomitant medication to the report, from the medication module. For example, if you want to indicate which medication were used to treat the event.

2.7.1 Instructions for reporting AE/SAE and SAR

Please see the *'8. PHARMACOVIGILANCE'* in the MASTER study protocol and pharmacovigilance chapter in the treatment-specific subprotocols for detailed information on the reporting of AE/SAE. The method for collecting and recording AEs will be spontaneous reporting and open question e.g. "How have you felt since previous visit" during the treatment and follow-up period.

Events available at the first administration of IMP are part of baseline information (pre-existing conditions). These events will be considered as AE, only if they worsen after first administration of IMP.
Elective procedures or surgery planned before first administration of IMP is not considered as an AE/SAE.
The evaluation period for safety will start at the time a signed and dated informed consent is obtained to at least 30 days after the last dose of study treatment. Adverse events including laboratory adverse events will be graded and summarized according to the CTCAE criteria.

3. OVERVIEW OF SMART-TRIAL DATA EVENTS FORMS DURING PROBIO

3.1 Data events during ProBio-mHSPC

To be completed upon protocol amendment.

3.2 Data events during ProBio-mCRPC

The data events i.e. patient visits in the ProBio process are: Inclusion (I), Baseline Assessment (BA) and the subsequent follow-up visits at predefined moments (*Figure 35*).

Each ProBio data event consists of multiple forms that need to be completed during the study. These forms are described in this document with guidance on how to complete them.

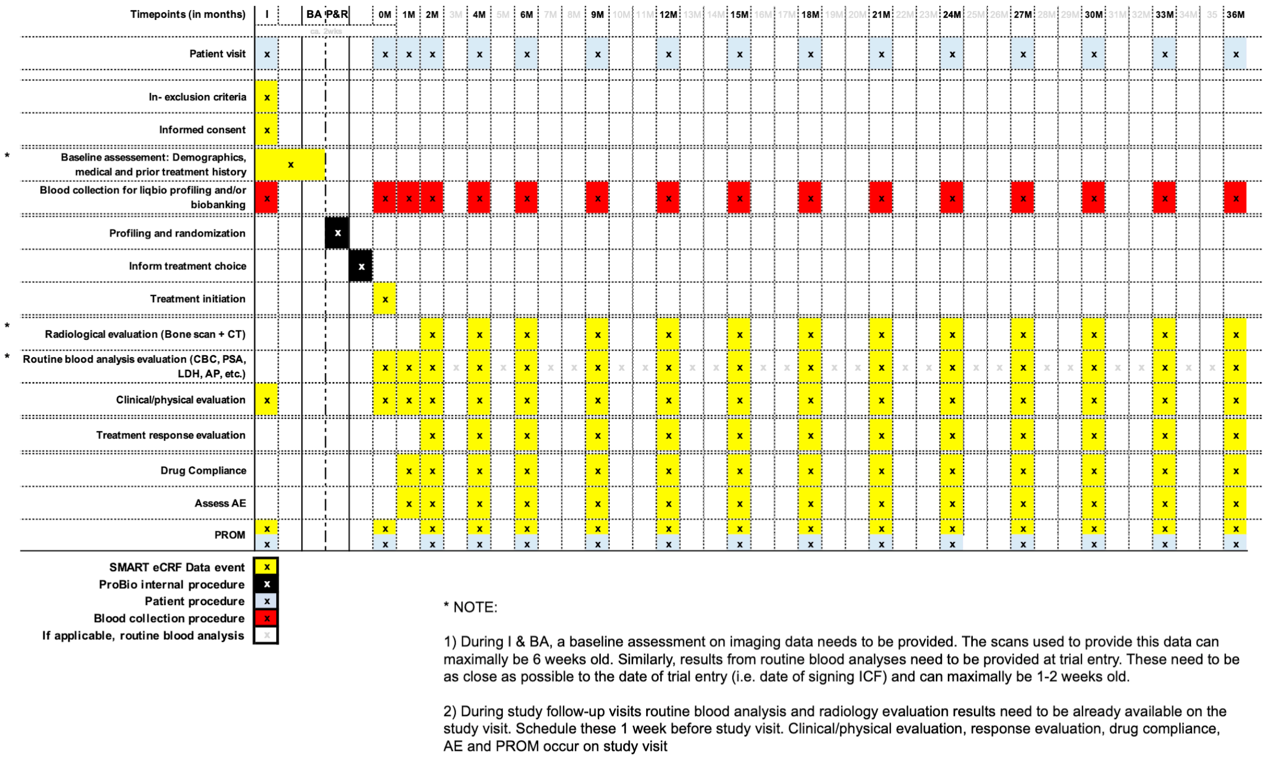

Figure 35 - Overview of data events in ProBio-mCRPC

3.2.1 Data event: Inclusion (I)

When a patient has been included in the study, 6 forms are to be completed. Please complete immediately when the patient is included, this will trigger that an email is sent to the sponsor informing us that blood will arrive for analysis and randomization. The data requested are important for us to be able to choose the correct sequencing for DNA analysis and for performing correct randomization.

1. Informed Consent
2. Inclusion/ Exclusion criteria
3. Physical examination-baseline
4. Clinical Characteristics
5. Treatment history
6. Routine clinical laboratory analysis

3.2.2 Data event: Baseline Assessment (BA)

The 4 forms can be completed when data is available.

1. Baseline assessment – Tumor characteristics
2. Baseline assessment – Imaging new
3. Patient Reported Outcome Measures – EORTC QLQ-C30 modified
4. Patient Reported Outcome Measures – EuroQol EQ-5D-5L

3.2.3 Data event: 0 Month (0M) – Treatment start

The result of the randomization is reported and the treatment is initiated. 5 forms need to be completed:

1. Treatment initiation form
2. Physical examination – follow-up
3. Routine clinical laboratory analysis
4. Patient reported Outcome measures – EORTC QLQ-C30 modified
5. Patient reported Outcome Measures – EuroQoL EQ-5D-5L

Do not forget to enter information about the treatment (both IMP and concomitant medication) in the Medication form in SMART Trial. Please see the SOP for using SMART- Trial in ProBio.

3.2.4 Data event: 1 Month follow-up (1M)

1. 1 month evaluation form
2. Routine clinical laboratory analysis
3. Patient Reported Outcome Measures - EORTC QLQ-C30 modified
4. Patient Reported Outcome Measures - EuroQol EQ-5D-5L

3.2.5 Data event during first 24 weeks: every 8 weeks, i.e. 2M, 4M & 6M

From the 2 months follow -up scheduled response evaluation is performed.

1. Follow-up evaluation form
2. Physical Examination - follow up
3. Routine clinical laboratory analysis
4. Radiologic evaluation - follow up new
5. Treatment response evaluation
6. Patient Reported Outcome Measures - EORTC QLQ-C30 modified
7. Patient Reported Outcome Measures - EuroQol EQ-5D-5L

3.2.6 Data event after the first 24 weeks: every 12 weeks until progression, i.e. 9M, 12M, 15M etc.

1. Follow-up evaluation form
2. Physical Examination - follow up
3. Routine clinical laboratory analysis
4. Radiologic evaluation - follow up new
5. Treatment response evaluation
6. Patient Reported Outcome Measures - EORTC QLQ-C30 modified
7. Patient Reported Outcome Measures - EuroQol EQ-5D-5L

3.2.7 Discontinuation of subject at a scheduled Data event

If a subject has progressed and this is documented during a scheduled follow-up, one form has to be completed.

1. Study discontinuation

3.2.8 Discontinuation as an unscheduled event visit

If a subject has progressed in between two scheduled follow-up visits, an unscheduled visit needs to be completed. This unscheduled visit consists of the following forms:

1. Follow-up evaluation form
2. Physical Examination - follow up
3. Routine clinical laboratory analysis
4. Radiologic evaluation - follow up new
5. Treatment response evaluation
6. Patient Reported Outcome Measures - EORTC QLQ-C30 modified
7. Patient Reported Outcome Measures - EuroQol EQ-5D-5L
8. Study discontinuation

3.2.9 Adverse event/ Serious Adverse event reporting

In case an AE/SAE needs to be reported, please use the AE/SAE reports in SMART-Trial. For more information please see the SOP for using SMART-Trial in ProBio.

AUXILIARY RESEARCH OBJECTIVES

ProBio

Trial title:

ProBio:
An outcome-adaptive and randomised multi-arm biomarker driven study in patients with metastatic prostate cancer

Coordinating Investigator and Sponsor's representative

Henrik Grönberg
Karolinska Institutet

TABLE OF CONTENTS

TABLE OF CONTENTS 2

1. Can ctDNA fraction dynamics replace PCWG3 for therapy response assessment? 3

2. Can ctDNA fraction bursts predict therapy response? 3

3. Retrospective analysis of the ctDNA profile to identify new biomarker signature - treatment associations 4

4. Analysis of cell-free DNA methylomes 4

6. RNA Analysis of thrombocytes 5

7. Prospective DNA analysis of CTCs 5

8. RNA analysis of CTCs 5

9. cfRNA and cfDNA analysis of urine 6

10. Prospective evaluation of clinical validity of PSMA-PET/CT-scan in mCRPC (CUTR-01 study) 6

11. Development of a new Patient Reported Outcome Measure (PROM) instrument to evaluate the Quality of Life (QoL) of patients with advanced prostate cancer 7

12. Quantitative analysis of androgen receptor perturbations using a blood-based liquid biopsy as a treatment-predictive biomarker for men with metastatic castration- resistant prostate cancer. 7

1. Can ctDNA fraction dynamics replace PCWG3 for therapy response assessment?

Tumour burden estimates by circulating tumour cells (CTCs) and ctDNA is correlated (Carreira et al. 2014), as verified by our pilot data (*Figure 1 - CTC enumeration in 7.5 ml of blood using the Cellsearch platform correlated with the ctDNA fraction*) (Mayrhofer et al. 2018). High CTC counts or ctDNA fraction is associated with poor prognosis before start of therapy (Annala et al. 2018; De Laere et al. 2019). Additionally, CTC and ctDNA dynamics at 4, 12 and 12 weeks is strongly associated with response to therapy (Goodall et al. 2017; Heller et al. 2018; Lorente et al. 2016). We will therefore test the hypothesis that ctDNA fraction dynamics can replace the response assessment according to the PCWG3 criteria, and will additionally benchmark ctDNA dynamics against CTC response measures (i.e. changes and CTC0 response endpoint) in a subpopulation of patients in whom prospective CTC profiling will be performed (see *'7. Prospective DNA analysis of CTCs'*). To explore this hypothesis blood will be collected on all men at 4, 8, 12 weeks and each subsequent visit before progression. The discriminatory strength of PCWG3 vs. ctDNA fraction dynamics in relation to PFS will be performed using the weighted c-index.

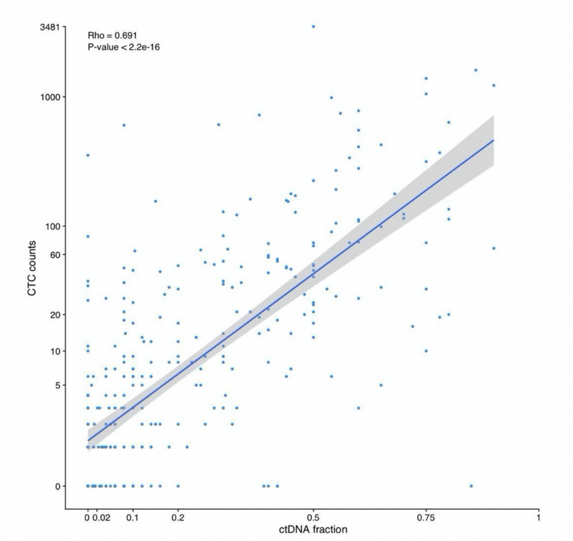

Figure 1 - CTC enumeration in 7.5 ml of blood using the Cellsearch platform correlated with the ctDNA fraction.

2. Can ctDNA fraction bursts predict therapy response?

Recent data from immunotherapy in melanoma (Xi et al. 2016) and EGFR targeted therapy in non-small cell lung cancer (Husain et al. 2017) suggest that the ctDNA fraction increases within the first week, if the patient is responding. The increase, or burst, in ctDNA fraction is speculated to be caused by drug-induced tumor apoptosis. To explore if an early ctDNA fraction burst has predictive value we will conduct a pilot on (n = 20) patients from each treatment arm if we are able to establish a feasible blood sampling procedure. Blood will be collected on a daily basis up to two weeks after the start of therapy.

3. Retrospective analysis of the ctDNA profile to identify new biomarker signature - treatment associations

The biomarker signatures applied at the start of the trial were selected based on known associations to therapy response. However, the ctDNA profile applied here comprehensively investigates the somatic alteration landscape reported for mCRCP, including alterations in e.g. *RB1, PTEN, PIK3CA*. Retrospective analysis will be performed for each treatment arm 1) when a treatment-biomarker signature combination graduates or 2) continuously as 50 patients have been included into a treatment arm. If an additional biomarker signature is associated with therapy response, protocol amendments will be sent to the regional ethical vetting boards and medical products agency or regulatory authorities for approval to evaluate the novel treatment-biomarker signature combination prospectively.

4. Analysis of cell-free DNA methylomes

Beside the detection of genomic alterations in cfDNA, the characterisation of large-scale epigenetic alterations by liquid biopsies is gaining momentum for the diagnosis and prognostication of cancer, especially in the early-stage disease. The ProBio trial will prospectively biobank plasma samples for future retrospective characterization of cell-free DNA methylation patterns using an immunoprecipitation-based protocol (Shen et al. 2018).

5. Analysis of cell-free RNA

Although the ctDNA-based targeted sequencing enables for the identification of somatic variation relevant for therapy selection such as DNA repair deficiency, microsatellite instability and alterations in the androgen receptor, additional information could potentially be obtained if transcriptomic information was accessible from the cancer. Cell-free RNA (cfRNA), originating mainly from extracellular vesicles including e.g. exosomes and apoptotic bodies, has the potential to provide treatment-relevant information from RNA. Profiling of cfRNA have demonstrated the presence of tumor derived transcripts (Del Re et al. 2017; Enderle et al. 2015). In addition, sequencing of cell-free RNA was recently demonstrated to improve mutation detection of mutation detection from liquid biopsies (Möhrmann et al. 2018). However, its full potential has not been thoroughly evaluated. In mCRPC, access to the RNA compartment would allow for the identification of additional treatment related biomarkers such as: androgen receptor splice variants (De Laere et al. 2019; Laere et al. 2019); kinase gene-fusions (Rubin et al. 2011); transcripts defining aggressive neuroendocrine mCRPC (Beltran et al. 2016); outlier kinase expression indicating sensitivity to inhibitors (Kothari et al. 2013); expressed mutations, relevant for neoantigen identification and immunotherapy (Liu and Mardis 2017). A pilot will be performed on ten samples from the pilot already analyzed for ctDNA with 1) known gene-fusions 2) detected point mutations and 3) high ctDNA-burden have. If cfRNA sequencing enables detection of cancer derived transcript, a detailed study plan will be established to explore the ability of cfRNA profiling to predict treatment response.

6. RNA Analysis of thrombocytes

Blood platelets contain information tumor derived transcripts which harbor information on tumor localization and enables sensitive detection localized and metastasized cancer (Best et al. 2017; Best et al. 2015). Biobanking protocols will be established to enable retrospective analysis of RNA from blood platelets in ProBio to investigate if blood-platelet derived RNA profiles may be applied to predict treatment response.

7. Prospective DNA analysis of CTCs

Although ctDNA is detected in the majority of mCRPC patients, copy-number alteration (CNA) profiling remains challenging in samples below 0.2 ctDNA fraction. However, high CTC counts may be detected in a proportion of low-fraction ctDNA samples (Mayrhofer et al. 2018). Therefore, CTCs may complement ctDNA profiling by enabling robust CNA profiling. Although papers exist that report successful mutational (Lohr et al. 2014) or CNA profiles from mPC patients (Gupta et al. 2017; Magbanua et al. 2013), prospective profiling is currently lacking. The high fraction of apoptotic CTCs (Swennenhuis et al. 2009) and challenges with performing single cell analysis (Navin 2014), requiring dedicated low-throughput infrastructure have prevented broad clinical applicability of CTCs. We will establish a workflow to enable analysis of DNA from single CTCs. The workflow will be applied on a pilot set of ten to a hundred patients to investigate 1) feasibility of the procedure to match a clinical relevant turnover and 2) the success-rate of single-cell profiling of CTCs. If successful, a more detailed project plan will be established to enable prospective CTC profiling to improve biomarker signature identification in the ProBio trial.

8. RNA analysis of CTCs

RNA sequencing of CTCs allows access to the expressed genes of the metastasis which may increase the clinical utility of liquid-biopsy based profiling for reasons established in exploratory objective 4. Single CTC RNA-sequencing is challenging for reasons established in exploratory objective 6. Although reports exist with where single CTC RNAseq has been performed (Miyamoto et al. 2015), most articles perform analysis of CTCs in a background of white blood cells (Antonarakis et al. 2014; De Laere et al. 2017; Singhal et al. 2018; Miyamoto et al. 2018). Biobanking protocols will be established to enable storage of enriched CTCs in a background of white blood cells to enable interrogation of metastasis derived transcripts. In addition, experimental approaches will be applied at multiple sites to enable single-cell RNA sequencing of CTCs. If pilots are successful, a more detailed project plan will be established for prospective profiling of CTCs in ProBio.

9. cfRNA and cfDNA analysis of urine

Recent publications demonstrate the presence of tumor-specific transcripts (McKiernan et al. 2016) and DNA molecules (Xia et al. 2016) in urine of men with localized and advanced prostate cancer, without the need for prostate massage. We will therefore collect urine from the study participants in ProBio to establish if cfRNA- and cfDNA extracted from urine carry clinically relevant information as described in exploratory objectives 1-4.

10. Prospective evaluation of clinical validity of PSMA-PET/CT-scan in mCRPC (CUTR-01 study)

CUTR-01 is a prospective, diagnostic, interventional cohort study evaluating the prognostic and therapy-predictive value of 68GaPSMA-11-PET/CT imaging and monitoring in patients with metastatic castration-resistant prostate cancer patients starting a first-line treatment (PI: Brieuc Sautois, brieuc.sautois@chuliege.be). ProBio patients enrolled at CHU Liege (Belgium), or other Belgian sites upon CUTR-01 protocol amendment, will be asked to additionally participate in this imaging study on top of ProBio. The primary objective of the study is to determine the prognostic and predictive value of baseline and follow-up PSMA-PET/CT imaging in patients with mCRPC starting a first-line treatment, i.e. abiraterone, enzalutamide, docetaxel or others. Imaging intervals within CUTR-01 do not interfere with the ProBio conventional imaging intervals (*Figure 2 - Imaging intervals during ProBio and CUTR-01*). However, in comparison to ProBio, CUTR01-enrolled patients will additionally be imaged after 1 month follow-up. PSMA-PET/CT image evaluation of target lesions will be correlated with response rates at 3 months, time to biochemical (i.e. PSA) progression (PSA-PFS), time to radiological progression by conventional imaging (rPFS) and time to no longer clinically benefiting (NLCB-PFS), all according to Prostate Cancer Working Group 3 (PCWG3) criteria. Inter- and intrapatient prevalence of tracer uptake discordance between PSMA-PET/CT and conventional imaging (CI) will be studied. The CUTR-01 protocol is available as an appendix.

FOLLOW UP (wk)

PCWG3	Every 8wk during first 24wk							Every 12wk till PD			
wk	**0**	**4**	**8**	**12**	**16**	**20**	**24**	**36**	**48**	**FU**	**PD**
mo	*0*	*1*	*2*	*3*	*4*	*5*	*6*	*9*	*12*		
**PROBIO CI**											
CI	x		x		x		x	x	x	Q12w	x
**CUTR01 CI + PSMA/PET**											
CI	x	x	x		x		x	x	x	Q12w	x
PSMA/PET	x	x			x		x		x	-	x

Figure 2 - Imaging intervals during ProBio and CUTR-01

11. Development of a new Patient Reported Outcome Measure (PROM) instrument to evaluate the Quality of Life (QoL) of patients with advanced prostate cancer

During the ProBio study patient-reported outcome (PROM) Quality-of-Life (QoL) data will be collected by means of standard questionnaires EuroQoL 5D (EQ-5D), EORTC-QLQ-C30 and the Brief Pain Inventory-Short Form (BPI-SF). Although these PROM instruments have been validated extensively and are being used in all new oncology trials, they lack a certain level of detail to describe the QoL state in men with advanced prostate cancer in the 21st century. Using the ProBio framework, the investigators will seek to establish and validate a novel PROM instrument, which would better question and reflect the QoL of the patient. This will among others be achieved by the active involvement and support from multiple prostate cancer patient advocacy groups.

12. Quantitative analysis of androgen receptor perturbations using a blood-based liquid biopsy as a treatment-predictive biomarker for men with metastatic castration- resistant prostate cancer.

During the ProBio study we seek to infer the treatment-predictive potential of a composite androgen receptor (*AR*) biomarker in men with metastatic castration-resistant prostate cancer (mCRPC). Our recent post-hoc analysis using circulating tumor DNA (ctDNA) and circulating tumor cell (CTC)-derived RNA suggested that the number of somatic alterations and/or splice variants in the *AR*, coined *AR*-burden, is associated with poor response to AR signalling inhibitors (ARSi) (De Laere et al. 2019). Upon updating our AR burden assay we will test the hypothesis if patients with high AR burden respond better to taxanes than ARSi. This will be achieved by retrospective analysis of biobanked plasma for cfRNA extractions and blood platelet pellets during the ProBio trial. We will investigate the prognostic value of the AR burden biomarker in the subpopulations of patients treated with ARSi or with chemotherapy. This will be estimated by Cox regression, modelling the progression free survival time as a function of the assigned treatment, the AR burden biomarker, and other relevant clinical features. The prognostic strength of AR burden will be quantified by a hazard ratio (HR) estimate with 95% confidence intervals, including an interaction term between treatment groups and the AR burden biomarker to account for a differential effect of the treatment in the biomarker-defined groups. We will test our hypothesis by testing if the interaction term in the multivariable Cox model is different from zero (i.e. to evaluate predictive potential). We will perform analysis after 150 mCRPC patients have been treated with ARSi and 150 have been treated with chemotherapy. Our power calculation incorporated a HR and prevalence of relevant AR burden as described previously (De Laere et al. 2019), PFS estimates for taxane-treated patients from the FIRSTANA trial (Oudard et al. 2017), and a postulated hazard ratio of 0.33, which resulted in a 96% power to detect if the interaction term is significantly different from zero.

APPENDICES

Trial title:

ProBio:
An outcome-adaptive and randomised multi-arm biomarker driven study in patients with metastatic prostate cancer

Coordinating Investigator and Sponsor's representative

Henrik Grönberg
Karolinska Institutet

TABLE OF CONTENTS

TABLE OF CONTENTS **2**

APPENDIX 1: LIST OF ABBREVIATIONS AND DEFINITIONS OF TERMS **3**

APPENDIX 2: ECOG PERFORMANCE STATUS (KARNOFSKY CONVERSION) **4**

APPENDIX 3: NEW YORK HEART ASSOCIATION CLASSIFICATION (NYHA) **5**

APPENDIX 4: RESPONSE EVALUATION CRITERIA IN SOLID TUMORS (RECIST V1.1) **6**

APPENDIX 5: PROSTATE CANCER WORKING GROUP 3 (PCWG3) CRITERIA **13**

APPENDIX 6: VARIABLES **16**

APPENDIX 7: REVISION HISTORY **19**

APPENDIX 8: REFERENCES **31**

APPENDIX 1: LIST OF ABBREVIATIONS AND DEFINITIONS OF TERMS

Abbreviation or term	Explanation
AE	Adverse Event
AR	Androgen Receptor
CNA	Copy-Number Alteration
CTCs	Circulating Tumor Cells
DFS	Disease-Free Survival
eCRF	electronic Case Report Form
FAP	Full Analysis Population
DRD	DNA-repair deficiency
DSMB	Data and Safety Monitoring Board
EAU guidelines	European Association of Urology guidelines
GCP	Good Clinical Practice
HR	Hazard Ratio
ICF	Informed Consent Form
ICH	International Conference on Harmonization
IXRS	Interactive Response System
IEC	Independent Ethics Committee
IMP	Investigational Medicinal Product
MedDRA	Medical Dictionary for Regulatory Activities
mCRPC	metastatic Castration Resistant Prostate Cancer
mHSPC	metastatic Hormone Sensitive Prostate Cancer
mPC	metastatic Prostate Cancer
MSI	Microsatellite Instable
MV-COX	Multivariable Cox
nmCRPC	non-metastatic Castrate Resistant Prostate Cancer
OS	Overall Survival
PFS	Progression Free overall Survival
PP	Per Protocol
QC	Quality Control
PRO	Patient reported outcome
CA	Competent Authorities
SAE	Serious Adverse Event
SmPC	Summary of Product Characteristics
SUSAR	Suspected Unexpected Serious Adverse Reaction
TTR	Time To Recurrence
T-EF	TMPRSS2-ERG gene fusion
TP53m	TP53-alterations

APPENDIX 2: ECOG PERFORMANCE STATUS (KARNOFSKY CONVERSION)

0 Fully active, able to carry on all predisease performance without restriction (Karnofsky 90-100)

1 Restricted in physically strenuous activity but ambulatory and able to carry out work on a light or sedentary nature, eg, light housework, office work. (Karnofsky 70-80)

2 Ambulatory and capable of all self-care but unable to carry out any work activities. Up and about more than 50% of waking hours. (Karnofsky 50-60)

3 Capable of only limited self-care; confined to bed or chair more than 50% of waking hours. (Karnofsky 30-40)

4 Completely disabled. Cannot carry on any self-care. Totally confined to bed or chair. (Karnofsky 10-20)

5 Dead. (Karnofsky 0)

APPENDIX 3: NEW YORK HEART ASSOCIATION CLASSIFICATION (NYHA)

The following table presents the New York Heart Association classification of cardiac disease:

Class	Functional Capacity	Objective Assessment
I	Patients with cardiac disease but without resulting limitations of physical activity. Ordinary physical activity does not cause undue fatigue, palpitation, dyspnea, or anginal pain.	No objective evidence of cardiovascular disease
II	Patients with cardiac disease resulting in slight limitation of physical activity. They are comfortable at rest. Ordinary physical activity results in fatigue, palpitation, dyspnea, or anginal pain.	Objective evidence of minimal cardiovascular disease
III	Patients with cardiac disease resulting in marked limitation of physical activity. They are comfortable at rest. Less than ordinary activity causes fatigue, palpitation, dyspnea, or anginal pain.	Objective evidence of moderately severe cardiovascular disease
IV	Patients with cardiac disease resulting in inability to carry on any physical activity without discomfort. Symptoms of heart failure or the anginal syndrome may be present even at rest. If any physical activity is undertaken, discomfort is increased.	Objective evidence of severe cardiovascular disease

APPENDIX 4: RESPONSE EVALUATION CRITERIA IN SOLID TUMORS (RECIST V1.1)

The following information was extracted from Section 3, Section 4, and Appendix I of the New response evaluation criteria in solid tumors: revised RECIST guideline (version 1.1) authored by Eisenhauer et al (complete publication in Eisenhauer EA, Therasse P, Bogaerts et al., New response evaluation criteria in solid tumours: Revised RECIST guideline (version 1.1). Eur J Cancer 2009;45:228-247).

Measurability of tumor at baseline

Definitions
At baseline, tumor lesions/lymph nodes will be categorized measurable or non-measurable as follows:

Measurable
Tumor lesions: Must be accurately measured in at least one dimension (longest diameter in the plane of measurement is to be recorded) with a minimum size of:

- 10 mm by CT scan (CT scan slice thickness no greater than 5 mm)

The following two methods of measure are not allowed in this protocol:

- 10 mm caliper measurement by clinical exam (lesions which cannot be accurately measured with calipers should be recorded as non-measurable)
- 20 mm by chest X-ray
- Malignant lymph nodes: To be considered pathologically enlarged and measurable, a lymph node must be ≥15 mm in short axis when assessed by CT scan (CT scan slice thickness recommended to be no greater than 5 mm). At baseline and in follow-up, only the short axis will be measured and followed. See also 'Baseline documentation of target and non-target lesions' in the RECIST guideline for information on lymph node measurement.

Non-measurable
All other lesions, including small lesions (longest diameter <10 mm or pathological lymph nodes with ≥10 to <15 mm short axis) as well as truly non-measurable lesions. Lesions considered truly nonmeasurable include: leptomeningeal disease, ascites, pleural or pericardial effusion, inflammatory breast disease, lymphangitic involvement of skin or lung, and abdominal masses/abdominal organomegaly identified by physical exam that is not measurable by reproducible imaging techniques.

Specifications by methods of measurements

Measurement of lesions
All measurements should be recorded in metric notation, using calipers if clinically assessed. All baseline evaluations should be performed as close as possible to the treatment start and never more than 4 weeks before the beginning of the treatment.

Method of assessment

The same method of assessment and the same technique should be used to characterize each identified and reported lesion at baseline and during follow-up. Imaging based evaluation should always be done rather than clinical examination.

Tumor response evaluation

Assessment of overall tumor burden and measurable disease
To assess objective response or future progression, it is necessary to estimate the overall tumor burden at baseline and use this as a comparator for subsequent measurements.

Baseline documentation of 'target' and 'non-target' lesions
When more than one measurable lesion is present at baseline all lesions up to a maximum of five lesions total (and a maximum of two lesions per organ) representative of all involved organs should be identified as target lesions and will be recorded and measured at baseline (this means in instances where patients have only one or two organ sites involved a maximum of two and four lesions respectively will be recorded). For evidence to support the selection of only five target lesions, see analyses on a large prospective database in the article by Bogaerts et al. (Reference #10 in Eisenhauer publication). Target lesions should be selected on the basis of their size (lesions with the longest diameter), be representative of all involved organs, but in addition should be those that lend themselves to reproducible repeated measurements. It may be the case that, on occasion, the largest lesion does not lend itself to reproducible measurement in which circumstance the next largest lesion, which can be measured reproducibly, should be selected.

Lymph nodes merit special mention since they are normal anatomical structures, which may be visible by imaging even if not involved by tumor. As noted in Section 3, pathological nodes which are defined as measurable and may be identified as target lesions must meet the criterion of a short axis of ≥15 mm by CT scan. Only the short axis of these nodes will contribute to the baseline sum. The short axis of the node is the diameter normally used by radiologists to judge if a node is involved by solid tumor. Nodal size is normally reported as two dimensions in the plane in which the image is obtained (for CT scan this is almost always the axial plane; for MRI the plane of acquisition may be axial, sagittal or coronal). The smaller of these measures is the short axis. For example, an abdominal node which is reported as being 20 mm•30 mm has a short axis of 20 mm and qualifies as a malignant, measurable node. In this example, 20 mm should be recorded as the node measurement (See also the example in Fig. 4 in Appendix II of the Eisenhauer reference). All other pathological nodes (those with short axis ≥10 mm but <15 mm) should be considered non-target lesions. Nodes that have a short axis <10 mm are considered non-pathological and should not be recorded or followed.

A sum of the diameters (longest for non-nodal lesions, short axis for nodal lesions) for all target lesions will be calculated and reported as the baseline sum diameters. If lymph nodes are to be included in the sum, then as noted above, only the short axis is added into the sum. The baseline sum diameters will be used as reference to further characterize any objective tumor regression in the measurable dimension of the disease.

All other lesions (or sites of disease) including pathological lymph nodes should be identified as nontarget lesions and should also be recorded at baseline. Measurements are not required and these lesions should be followed as ‘present’, ‘absent’, or in rare cases ‘unequivocal progression’ (more details to follow). In addition, it is possible to record multiple nontarget lesions involving the same organ as a single item on the case record form (e.g. ‘multiple enlarged pelvic lymph nodes’ or ‘multiple liver metastases’).

Response criteria
This section provides the definitions of the criteria used to determine objective tumor response for target lesions.

Evaluation of target lesions

Complete Response (CR): Disappearance of all target lesions. Any pathological lymph nodes (whether target or non-target) must have reduction in short axis to <10 mm.

Partial Response (PR): At least a 30% decrease in the sum of diameters of target lesions, taking as reference the baseline sum diameters.

Progressive Disease: At least a 20% increase in the sum of diameters of target lesions, taking as reference the smallest sum on study (this includes the baseline sum if that is the smallest on study). In addition to the relative increase of 20%, the sum must also demonstrate an absolute increase of at least 5 mm. (Note: the appearance of one or more new lesions is also considered progression).

Stable Disease (SD): Neither sufficient shrinkage to qualify for PR nor sufficient increase to qualify for progressive disease, taking as reference the smallest sum diameters while on study.

Special notes on the assessment of target lesions

Lymph nodes. Lymph nodes identified as target lesions should always have the actual short axis measurement recorded (measured in the same anatomical plane as the baseline examination), even if the nodes regress to below 10 mm on study. This means that when lymph nodes are included as target lesions, the ‘sum’ of lesions may not be zero even if complete response criteria are met, since a normal lymph node is defined as having a short axis of <10 mm. Case report forms or other data collection methods may therefore be designed to have target nodal lesions recorded in a separate section where, in order to qualify for CR, each node must achieve a short axis <10 mm. For PR, SD and progressive disease, the actual short axis measurement of the nodes is to be included in the sum of target lesions.

Target lesions that become ‘too small to measure’. While on study, all lesions (nodal and non-nodal) recorded at baseline should have their actual measurements recorded at each subsequent evaluation, even when very small (eg 2 mm). However, sometimes lesions or lymph nodes which are recorded as target lesions at baseline become so faint on CT scan that the radiologist may not feel

comfortable assigning an exact measure and may report them as being ‘too small to measure’. When this occurs it is important that a value be recorded on the CRF. If it is the opinion of the radiologist that the lesion has likely disappeared, the measurement should be recorded as 0 mm. If the lesion is believed to be present and is faintly seen but too small to measure, a default value of 5 mm should be assigned (Note: It is less likely that this rule will be used for lymph nodes since they usually have a definable size when normal and are frequently surrounded by fat such as in the retroperitoneum; however, if a lymph node is believed to be present and is faintly seen but too small to measure, a default value of 5 mm should be assigned in this circumstance as well). This default value is derived from the 5 mm CT slice thickness (but should not be changed with varying CT slice thickness). The measurement of these lesions is potentially non reproducible; therefore providing this default value will prevent false responses or progressions based upon measurement error. To reiterate, however, if the radiologist is able to provide an actual measure, that should be recorded, even if it is below 5 mm.

Lesions that split or coalesce on treatment. When non-nodal lesions ‘fragment’, the longest diameters of the fragmented portions should be added together to calculate the target lesion sum. Similarly, as lesions coalesce, a plane between them may be maintained that would aid in obtaining maximal diameter measurements of each individual lesion. If the lesions have truly coalesced such that they are no longer separable, the vector of the longest diameter in this instance should be the maximal longest diameter for the ‘coalesced lesion’.

Evaluation of non-target lesions

This section provides the definitions of the criteria used to determine the tumor response for the group of non-target lesions. While some non-target lesions may actually be measurable, they need not be
measured and instead should be assessed only qualitatively at the timepoints specified in the protocol.

Complete Response (CR): Disappearance of all non-target lesions and normalization of tumor marker level. All lymph nodes must be non-pathological in size (<10 mm short axis).

Non-CR/Non-progressive disease: Persistence of one or more non-target lesion(s) and/or maintenance of tumor marker level above the normal limits.

Progressive Disease: Unequivocal progression (see comments below) of existing non-target lesions. (Note: the appearance of one or more new lesions is also considered progression).

Special notes on assessment of progression of non-target disease

The concept of progression of non-target disease requires additional explanation as follows:

When the patient also has measurable disease. In this setting, to achieve 'unequivocal progression' on
the basis of the non-target disease, there must be an overall level of substantial worsening in non-target disease such that, even in presence of SD or PR in target disease, the overall tumor burden has increased sufficiently to merit discontinuation of therapy. A modest 'increase' in the size of one or more non-target lesions is usually not sufficient to quality for unequivocal progression status. The designation of overall progression solely on the basis of change in non-target disease in the face of SD or PR of target disease will therefore be extremely rare.

When the patient has only non-measurable disease. This circumstance arises in some Phase studies when it is not a criterion of study entry to have measurable disease. The same general concepts apply here as noted above, however, in this instance there is no measurable disease assessment to factor into the interpretation of an increase in non-measurable disease burden. Because worsening in non-target disease cannot be easily quantified (by definition: if all lesions are truly non-measurable) a useful test that can be applied when assessing patients for unequivocal progression is to consider if the increase in overall disease burden based on the change in non-measurable disease is comparable in magnitude to the increase that would be required to declare progressive disease for measurable disease: i.e. an increase in tumor burden representing an additional 73% increase in 'volume' (which is equivalent to a 20% increase diameter in a measurable lesion). Examples include an increase in a pleural effusion from 'trace' to 'large', an increase in lymphangitic disease from localized to widespread, or may be described in protocols as 'sufficient to require a change in therapy.' If 'unequivocal progression' is seen, the patient should be considered to have had overall progressive disease at that point. While it would be ideal to have objective criteria to apply to non-measurable disease, the very nature of that disease makes it impossible to do so, therefore the increase must be substantial.

New lesions

The appearance of new malignant lesions denotes disease progression; therefore, some comments on detection of new lesions are important. There are no specific criteria for the identification of new radiographic lesions; however, the finding of a new lesion should be unequivocal: i.e. not attributable to differences in scanning technique, change in imaging modality or findings thought to represent something other than tumor (for example, some 'new' bone lesions may be simply healing or flare of pre-existing lesions). This is particularly important when the patient's baseline lesions show partial or complete response. For example, necrosis of a liver lesion may be reported on a CT scan report as a 'new' cystic lesion, which it is not.

A lesion identified on a follow-up study in an anatomical location that was not scanned at baseline is considered a new lesion and will indicate disease progression. An example of this is the patient who has visceral disease at baseline and while on study has a CT or MRI brain ordered which reveals metastases.

The patient's brain metastases are considered to be evidence of progressive disease even if he/she did not have brain imaging at baseline.

If a new lesion is equivocal, for example because of its small size, continued therapy and follow-up evaluation will clarify if it represents truly new disease. If repeat scans confirm there is definitely a new lesion, then progression should be declared using the date of the initial scan.

Timepoint response

It is assumed that at each protocol specified timepoint, a response assessment occurs. Table 1 in this attachment provides a summary of the overall response status calculation at each timepoint for patients who have measurable disease at baseline.

When patients have non-measurable (therefore non-target) disease only, Table 2 in this attachment is to be used.

Missing assessments and inevaluable designation

When no imaging/measurement is done at all at a particular timepoint, the patient is not evaluable (NE) at that timepoint. If only a subset of lesion measurements are made at an assessment, usually the case is also considered NE at that timepoint, unless a convincing argument can be made that the contribution of the individual missing lesion(s) would not change the assigned timepoint response. This would be most likely to happen in the case of progressive disease. For example, if a patient had a baseline sum of 50 mm with three measured lesions and at follow-up only two lesions were assessed, but those gave a sum of 80 mm, the patient will have achieved progressive disease status, regardless of the contribution of the missing lesion.

Best overall response: all timepoints

The best overall response is determined once all the data for the patient is known.

Best response determination in studies where confirmation of complete or partial response IS NOT required: Best response in these studies is defined as the best response across all timepoints (for example, a patient who has SD at first assessment, PR at second assessment, and progressive disease on last assessment has a best overall response of PR). When SD is believed to be best response, it must also meet the protocol specified minimum time from baseline. If the minimum time is not met when SD is otherwise the best timepoint response, the patient’s best response depends on the subsequent assessments.

For example, a patient who has SD at first assessment, progressive disease at second and does not meet minimum duration for SD, will have a best response of progressive disease. The same patient lost to follow-up after the first SD assessment would be considered inevaluable.

Table 1 - Timepoint response: patients with Target (+/- non-target) disease			
**Target lesions**	**Non-target lesions**	**New lesions**	**Overall response**
CR	CR	No	CR
CR	Non-CR/non-PD	No	PR
CR	Not evaluated	No	PR
PR	Non-PD or not all evaluated	No	PR
SD	Non-PD or not all evaluated	No	SD
Not all evaluated	Non-PD	No	NE
PD	Any	Yes or No	PD
Any	PD	Yes or No	PD
Any	Any	Yes	PD
CR = complete response; PR = partial response; SD = stable disease; PD = progressive disease; NE = not evaluable.			

Table 2 - Timepoint response: patients with non-target disease only		
**Non-target lesions**	**New lesions**	**Overall response**
CR	No	CR
Non-CR/non-PD	No	Non-CR/non-PD[a]
Not all evaluated	No	NE
Unequivocal PD	Yes or No	PD
Any	Yes	PD
CR = complete response; PD = progressive disease; NE = not evaluable.		
a 'Non-CR/non-PD' is preferred over 'stable disease' for non-target disease since SD is increasingly used as endpoint for assessment of efficacy in some studies so to assign this category when no lesions can be measured is not advised.		

APPENDIX 5: PROSTATE CANCER WORKING GROUP 3 (PCWG3) CRITERIA

The following information was extracted from the recommendations of the Prostate Cancer Clinical Trials Working Group 3 authored by Scher et al (complete publication in Scher HI, Morris MJ, Stadler WM, Higano C, Basch E, Fizazi K, et al. Trial Design and Objectives for Castration-Resistant Prostate Cancer: Updated Recommendations From the Prostate Cancer Clinical Trials Working Group 3. 2016. pp. 1402–18.).

On-treatment evaluations should include physical examinations, symptom assessments, and laboratory studies to assess safety, with appropriate attribution to the disease or therapy. Imaging should include cross-sectional imaging of the chest, abdomen, and pelvis, as well as bone scintigraphy, regardless of whether patients have involvement of those sites at baseline. Imaging strategies restricted to known sites of disease risk missing disease progression at new sites.

To minimize patient exposure to ineffective treatment, and to better assess the timing of the antitumor effects of an agent for which the optimal timing is not known, an 8- to 9-week assessment interval for the first 6 months and every 12 weeks thereafter is advised, as applied in ProBio. PCWG3 retains the recommendation to report outcomes by manifestation (eg, host or tumor biomarkers, symptoms, site of spread) and not to report grouped categorizations of response such as complete response, partial response, or progression on the basis of multiple manifestations. In patients who show evidence of benefit, PCWG3 advises continuing therapy in the case of an isolated PSA rise after an initial decline until radiographic or clinical progression is manifest.
PCWG2 encouraged the continuation of treatment if a rising PSA or worsening of an isolated disease site that was not clinically significant was the sole indicator of disease progression and the patient was otherwise tolerating therapy. Now, recognizing the biologic heterogeneity of individual metastatic lesions, PCWG3 draws the distinction between documenting progression for consistency of reporting (eg, recording the date of documented progression in a site of disease such as a lymph node that is unlikely to adversely affect prognosis) versus the decision to stop therapy.

To address this, PCWG3 introduces the no longer clinically benefiting (NLCB) reporting metric defined as the date and the specific reason(s) a therapy was ultimately discontinued. This end point permits individualized provider-patient decisions to continue or discontinue a treatment based on the primary therapeutic objective for which it is being administered and assessed, be it quality of life, PROs, or survival.
As an example, in cases in which multiple sites of disease continue to respond but one to two sites grow, focal therapy such as radiation or surgery could be administered to the resistant site(s) and systemic therapy continued. Similarly, therapy may be continued if progression by PSA or imaging is slow and the disease-related symptoms that were present at baseline remain controlled. Important here is to record in detail the specific reasons why a therapy was ultimately discontinued, which may include clinical deterioration (clarifying whether it is disease or therapy related) or need for a change in systemic therapy. PCWG3 cannot define the risks/benefits at the individual level for continuation of therapy beyond progression, but sets the goal of prospectively defining the circumstances in which scenarios are identified where continuing a therapy is justified.
Suggested Outcome Measures for Clinical Trials in Metastatic Prostate Cancer: Report by Disease Manifestation – Table 5 from Scher et al. (JCO, 2016)

Table 5. Suggested Outcome Measures for Clinical Trials in Metastatic Prostate Cancer: Report by Disease Manifestation

Variable	PCWG2 (2008)	PCWG3 (2015)
Histology	Not addressed	Encourage rebiopsy of metastatic sites or local recurrence at progression to evaluate for histologic (ie, neuroendocrine/ small cell) transformation; in the context of clinical trials, encourage rebiopsy for biomarker assessment
Blood-based markers		
PSA	Recognize that a favorable effect on PSA may be delayed for ≥ 12 weeks, even for a cytotoxic drug	Retained
	Monitor PSA by cycle but plan to continue through early rises for a minimum of 12 weeks unless other evidence of progression	Retained
	Ignore early rises (before 12 weeks) in determining PSA response	Retained
	For control/relieve/eliminate end points:	For control/relieve/eliminate end points:
	Record the percent change from baseline (rise or fall) at 12 weeks, and separately, the maximal change (rise or fall) at any time using a waterfall plot	Retained, except with timing (8-9 or 12 weeks) depending on trial design
		Separately report the proportion of patients who have undergone radical prostatectomy and achieved a nadir less than 0.2 ng/mL *v* primary radiation therapy–treated patients who achieved a nadir less than 0.5 ng/mL
		Describe absolute changes in PSA over time from baseline to best response
	For delay/prevent end points (progression):	For delay/prevent end points (progression):
	After decline from baseline: record time from start of therapy to first PSA increase that is ≥ 25% and ≥ 2 ng/mL above the nadir, and which is confirmed by a second value ≥ 3 weeks later (ie, a confirmed rising trend); the requirement for an increase of 5 ng/mL was decreased to 2 ng/mL, and the requirement for a 50% increase was reduced to 25%	Retained (standards for reporting PSA progression date may not indicate a need to stop treatment)
	Recording the duration of PSA decline of little value	Retained
	No decline from baseline: PSA progression ≥ 25% increase and ≥ 2 ng/mL increase from baseline beyond 12 weeks	Relate to mechanism of drug and anticipated timing of potential favorable/unfavorable effects on PSA, if present
CTC	Not addressed	Enumerate at the start of treatment: Record as favorable (four or fewer cells per 7.5 mL of blood) or unfavorable (five or more cells per 7.5 mL)
		If unfavorable, monitor for changes after treatment
		For control/relieve/eliminate end points:
		Report as change from unfavorable (five or more cells per 7.5 mL of blood) to favorable (four or fewer cells per 7.5 mL) and separately, the percent change from baseline using a waterfall plot
		For delay/prevent end points: no validated definition exists (however, rising CTC counts are associated with a poor prognosis)
LDH, total alkaline phosphatase, bone-specific alkaline phosphatase, urine *N*-telopeptide, hemoglobin, NLR	Not addressed	Descriptively report changes over time, may include the proportion showing normalization of a given biomarker and/or waterfall plots of percent change from baseline in a given biomarker
		Report institutional normal ranges to determine normalization of a given biomarker
Imaging biomarkers: nodal and visceral		
For control/relieve/eliminate end points		
General	Record changes in nodal sites separately from visceral sites	Record changes in lymph nodes, lung, liver, adrenal, and CNS sites separately

(continued on following page)

Table 5. Suggested Outcome Measures for Clinical Trials in Metastatic Prostate Cancer: Report by Disease Manifestation (continued)

Variable	PCWG2 (2008)	PCWG3 (2015)
	Use RECIST with caveats:<br>Record changes in size using waterfall plot<br>Confirm favorable change with second scan<br>Record complete elimination of disease at any site separately	Record up to five lesions per site of disease<br>Use RECIST 1.1 with caveats:<br>Record changes in size using waterfall plot<br>Confirm favorable change with second scan<br>Record complete elimination of disease at any site separately
Nodes	Only report changes in lymph nodes that were ≥ 2 cm in the long axis at baseline	Only report changes in lymph nodes that were ≥1.5 cm in the short axis<br>Record changes in pelvic (regional) nodes *v* extrapelvic (distant/metastatic) nodes separately
Visceral	Use RECIST with caveats above	Use RECIST 1.1 with caveats:<br>Record changes in liver, lung, adrenal, and CNS separately<br>Only report changes in lesions ≥ 1.0 cm in the longest dimension
For delay/prevent end points		
Nodal and visceral	Use RECIST criteria for progression, with additional requirement that progression be confirmed by a second scan ≥ 6 weeks later (the second scan is particularly important when anticipated effect on PSA is delayed, or for biologic therapies)	General:<br>Record changes in nodal and visceral (lung, liver, adrenal, and CNS) disease separately<br>Use RECIST 1.1 but clearly record type of progression (growth of existing lesions *v* development of new lesions) separately by site<br>The recommendations apply to both nmCRPC and mCRPC<br>Record up to five lesions per site of spread<br>Report the proportion who have not progressed at fixed time points (6 or 12 months)
	Note that for some treatments, a lesion may increase in size before it decreases	Retained
Nodal	As above	Previously normal (< 1.0-cm) lymph nodes must have grown by ≥ 5 mm in the short axis from baseline or nadir and be ≥ 1.0 cm in the short axis to be considered to have progressed<br>Nodes that have progressed to 1.0 to less than 1.5 cm are pathologic, subject to clinical discretion, and nonmeasurable<br>For existing pathologic adenopathy (≥ 1.5 cm), progression is defined per RECIST 1.1
Imaging biomarkers: bone		
Metastatic	For control/relieve/eliminate end points:	For control/relieve/eliminate end points:
	Record changes as improved or stable (no new lesions) or worse (new lesions)	Retained with addition of resolved bone lesion
	Changes in intensity of uptake alone do not constitute progression or regression	Retained
	No new lesions: continue therapy in absence of other signs of progression	Retained
	New lesions (See Progression below)	Retained
	For delay/prevent end points (progression):<br>Progression:<br>Exclude pseudoprogression in the absence of symptoms or other signs of progression	For delay/prevent end points (progression):<br>Progression:<br>Retained
	At least two new lesions on first post-treatment scan, with at least two additional lesions on the next scan (2+2 rule)	
	If at least two additional new lesions are seen on the next (confirmatory) scan, the date of progression is the date of the first post-treatment scan, when the first two new lesions were documented	Retained

(continued on following page)

Table 5. Suggested Outcome Measures for Clinical Trials in Metastatic Prostate Cancer: Report by Disease Manifestation (continued)

Variable	PCWG2 (2008)	PCWG3 (2015)
	For all scans after the first post-treatment scan, at least two new lesions	For scans after the first post-treatment scan, at least two new lesions relative to the first post-treatment scan confirmed on a subsequent scan
	Date of progression is the date of the scan that first documents the second lesion	Retained
	Changes in intensity of uptake alone do not constitute either progression or regression	Retained
		Report the proportion of patients who have not progressed at fixed time intervals (6 and 12 months)
nmCRPC	Not addressed	Nonmetastatic to metastatic progression:<br>Any new unequivocal bone lesion, except if that lesion appears in the first post-treatment scan; in that case, document the event, continue treatment until 2 additional new lesions appear, and record both events
Patient-reported outcomes	Consider independently of other outcome measures	Pain palliation assessment requires a patient population with clinically meaningful pain at baseline (eg, ≥ 4 on a 10-point pain intensity scale) and response defined as a clinically meaningful score improvement at a subsequent time point (eg, a 30% relative or 2-point absolute improvement from baseline at 12 weeks, confirmed at least 2 weeks later, without an overall increase in opiate use)
	For control/relieve/eliminate end points:<br>Document pain and analgesia at entry with a lead-in period and measure repeatedly at 3- to 4-week intervals	For control/relieve/eliminate end points:<br>Serial (eg, daily × 7 days) assessments at each time point can improve the stability of values
	Perform serial assessments of global changes in HRQoL, urinary or bowel compromise, pain management, additional anticancer therapy	Principles may be extended for any PRO for which a clinically meaningful baseline PRO score has been determined together with a responder definition that is based on a sustained clinically meaningful score improvement
	Ignore early changes (≤ 12 weeks) in pain or HRQoL in absence of compelling evidence of disease progression	
	For delay/prevent end points:<br>Confirm response or progression of pain or HRQoL end points ≥ 3 weeks later	For delay/prevent end points:<br>Patients with any level of baseline pain, including no pain, are eligible to be evaluated for prevent/delay end points; those without pain are followed for development of pain, whereas those with baseline pain are followed for progression (eg, a 2-point increase without an overall decrease in opiate use)<br>Pain assessment should be administered at treatment discontinuation and once again if feasible (eg, 2 to 4 weeks later)<br>Time to deterioration of physical function and/or HRQoL scores should also be included, with a priori thresholds defining clinically meaningful deterioration score changes that are based on prior published data for the selected questionnaire

Abbreviations: CTC, circulating tumor cell; HRQoL, health-related quality of life; LDH, lactate dehydrogenase; mCRPC, metastatic castration-resistant prostate cancer; NLR, neutrophil/ lymphocyte ratio; nmCRPC, nonmetastatic castration-resistant prostate cancer; PCWG2, Prostate Cancer Clinical Trials Working Group 2; PCWG3, Prostate Cancer Clinical Trials Working Group 3; PRO, patient-reported outcome; PSA, prostate-specific antigen; RECIST, Response Evaluation Criteria in Solid Tumors.

APPENDIX 6: VARIABLES

- ICF signed (y/n)
- ICF date
- In/exclusion criteria (y/n)

- Physical examination – Trial entry and Follow-up
  - ECOG performance (0-2)
  - Blood pressure (mmHg)
  - Heart rate (bpm)
  - Height (cm)
  - Weight (kg)

- Clinical characteristics at trial entry
  - 1L mCRPC (y/n)
  - PSA progression (y/n)
  - PSADT (mo)
  - Disease in Bone (y/n)
    - Type of Bone disease (stable/progressive)
      - Type of progressive Bone lesions (pre-existing/new)
  - Disease in LN (y/n)
    - Type of LN disease (stable/progressive)
      - LN lesion(s) location (pelvic/extrapelvic)
      - Type of progressive LN lesions (pre-existing/new)
  - Disease in Viscera (y/n)
    - Type of Bone disease (stable/progressive)
      - visceral lesion(s) sites
      - Type of progressive Bone lesions (pre-existing/new)
  - Analgesics use (y/n)
  - BHA use (y/n)
    - Which BHA
  - Comorbidities (y/n)
    - Which?
  - Cardiovascular disease history (y/n)

- Treatment history
  - Primary Tx for loc disease (RALP/RT/HT/AM/unknown/NA)
    - Surgical margin (R0/R1/unknown)
    - LND (y/n)
      - Type LND (pelvic/extended)
    - Type HT? (medical/surgical)

  - Systemic Tx for BCR, mHSPC, nmCRPC (y/n)
    - Which?
      - Type HT? (medical/surgical)
      - 1st gen anti-androgen?
        - Indication AA? (Flare prevention / CAB)?
      - Date 1st cycle docetaxel for mHSPC (date)
      - Number of cycles docetaxel (1-6)
  - Local EBRT for LV DN mHSPC (y/n)
  - Systemic Tx mCRPC (y/n)
    - Which?
      - Start and stop (date)
  - Salvage or palliative RT (y/n)

- Tumor characteristics and history
  - Date diagnosis
  - iPSA
  - TNM
  - ISUP/Gleason
    - SC/NED features (y/n)
  - Date evidence M1 disease

- Routine blood analysis – Trial entry and Follow-up
  - Hb
  - RBC
  - WBC
  - Neu
  - Lym
  - Mon
  - Platelets
  - PSA
  - T
  - LDH
  - ALP
  - Albumin
  - Creatine
  - AST
  - ALT
  - Bilirubin

- Imaging analysis – Trial entry and Follow-up
  - Which imaging modalities (tc99 / CT / MRI / PET/CT)
    - Date of imaging
  - Bone metastases by Tc99 (y/n)
    - Number of bone metastases (1 / 2-4 / 5-9 / 10-19 / ≥20 / diffuse)
    - Indication of progressive disease (y/n)
      - Type of progressive Bone lesions (pre-existing/new)

  - Metastases by CT (y/n)
    - Which sites (LN / visceral / bone / other)
      - Number of measurable lesions (1-20)
      - Location of lesions
    - Indication of progressive disease (y/n)
      - Type of progressive lesions (pre-existing/new)

- PROM – Trial entry and Follow-up
  - EORTC QLQ-C30
  - EQ-5D-5L
  - BPI-SF

- Follow-up treatment evaluation
  - Date of follow-up visit
  - Treatment compliance
  - PSA evaluation (increasing/decreasing/stable)
  - Radiological evaluation (SD/radiological response/PD/not evaluable)
  - Symptoms of clinical progression

- (S)AE
  - Date of Awareness
  - Event description
  - End date
  - Severity
  - Relationship to IMP
  - Action taken

- Medication
  - Concomitant/IMP
  - Medication name
  - Dose
  - Frequency
  - Start date
  - Stop date

- Genomics
  - ctDNA fraction
  - Any somatic clonal drivers including gene and driver type (homozygous deletion, focal amplification, point mutation, indel, structural variant)
  - Biallelic inactivation status
  - Any relevant high-penetrant germline alteration
  - Biomarker signature

APPENDIX 7: REVISION HISTORY

7.1 GENERAL DESCRIPTION OF PROTOCOL CHANGES

Number	Document date	Revision (main changes from previous version)
1.0	22 June, 2018	Initial protocol
2.0	05 Nov, 2018	Removal of RA223 and addition of Carboplatin as a possible treatment.<br>Addition of urine samples.
3.0	01 Feb, 2019	Clarification on carboplatin, update statistical analysis plan, minor modification on inclusion-and exclusion criteria, editorial changes.<br><br>Addition of a technical pilot study.
4.0	1 Dec, 2020	Expansion of the ProBio trial towards the metastatic hormone-sensitive prostate cancer setting<br><br>Restructuring of protocol based on the “Recommendation Paper on the Initiation and Conduct of Complex Clinical Trials”<br><br>Update trial sites<br><br>Definition of future therapy modalities upon sponsor involvement and protocol amendment<br><br>Introduce the combination therapy niraparib plus abiraterone plus prednisone for mHSPC and mCRPC as company-sponsored IMP<br><br>Generalization blood collection and processing procedures according to site/country of patient accrual<br><br>Update auxiliary research projects
4.1	24 Mar, 2021	In MASTER PROTOCOL<br>- in chapter 4.2, clarification in treatment sequencing<br>- single ICF procedure<br>- chapter 7. study procedures<br>- chapter ethics and consent<br><br>In SUB PROTOCOLS<br>- adding FDC abiraterone plus niraparib in subprotocol<br>- v1.0 → v1.1<br><br>In APPENDICES<br>- adding APPENDIX 6: VARIABLES<br>- v1.0 → v1.1

7.2 DETAILED DESCRIPTION OF PROTOCOL CHANGES BETWEEN TWO LATEST VERSIONS

Version 3.0	Version 4.0
**ProBio protocol design**	
**Conventional protocol design**	**Structured protocol design**<br>ProBio is an outcome-adaptive and randomised multi-arm biomarker driven study in patients with metastatic prostate cancer. Given the complexity of the trial design and conduct, the clinical protocol has been built up in a structured way, following the recommendations from the Clinical Trials Facilitation and Coordination Group (CTFG), a working group of the Heads of Medicines Agencies on clinical trials. The structured protocol design is characterised by a master protocol with several sub-protocols, trial conduct supplement and appendices. The trial and structured protocol design allow for extensive adaptations where e.g. arms with new IMPs are being opened and closed or new biomarker signatures are being incorporated during the conduct of the trial via substantial amendments of new/existing sub-protocols and trial conduct supplements.
**Hypothesis**	
The proposed hypothesis is that treatment decisions based on biomarker signatures identified by sequencing ctDNA significantly will increase the progression free survival (PFS) in patients with metastatic castrate resistant prostate cancer (mCRPC) compared to current clinical standard of care.	The proposed hypothesis is that treatment class decisions based on biomarker signatures identified by sequencing ctDNA and/or diagnostic biopsies (in case of low/undetectable circulating tumour burden in patients with de novo mHSPC) significantly will increase the progression free survival (PFS) in patients with metastatic hormone- sensitive (mHSPC) and castration-resistant (mCRPC) prostate cancer compared to current clinical standard of care (SOC). The goal is to identify in which biomarker signature a therapy class is superior to SOC.
**Trial population**<br>*Male patients, aged above 18 years, with histologically confirmed prostate adenocarcinoma, initiating systemic therapy for metastatic disease, encompassing:*	
N/A	Newly diagnosed (i.e. de novo) metastatic hormone-sensitive prostate cancer (mHSPC)
Patients with metastatic castration resistant prostate cancer. Patients are not eligible if they have received maximum two of the study drugs, prior to study inclusion.	First-line mCRPC (i.e. progressive metastatic prostate cancer under castrate levels (<50 ng/dl) of serum testosterone, as defined by the EAU guidelines)

<table>
<tr><th colspan="2">Primary clinical efficacy objectives and endpoints</th></tr>
<tr><td>To determine whether treatment choice based on a biomarker signature can improve PFS compared to standard of care in male patients with mCRPC, where standard of care is defined as clinician-patient treatment decision without access to information on the bio-marker profile.
Progression free survival is defined using Time to no longer clinical benefit (NLCB), Prostate Cancer Working Group (PCWG3 guidelines)</td><td>To determine whether treatment class choice based on a biomarker signature can improve PFS compared to standard of care in male patients with mHSPC and mCRPC, where standard of care is defined as clinician-patient treatment decision without access to information on the bio-marker profile.
Progression free survival (PFS) is defined according to disease stage at trial entry, using:
For mHSPC: Time to development of castration-resistance (EAU guidelines)
For mCRPC: Time to no longer clinical benefit (NLCB), Prostate Cancer Working Group (PCWG3 guidelines)</td></tr>
<tr><th colspan="2">Secondary clinical efficacy objectives and endpoints</th></tr>
<tr><td>To determine whether treatment choices based on a biomarker signature can, compared to standard of care:
● improve treatment response rate (RR) after 4 months of treatment
● improve overall survival
● improve quality of life. QoL will be assessed using the following instruments: EQ-5D-5L, EORTC QLQ-C30
● improve health economy</td><td>To determine whether treatment class choices based on a biomarker signature can:
● improve PSA-PFS
● improve radiographic PFS (rPFS)
● improve treatment response rate (RR) after 3-4 months of treatment
● improve PFS2, defined as the time from the initial study randomisation to the 2nd progression or death from any cause
● improve overall survival, defined as the time from the initial study randomisation to death from any cause
● improve quality of life. QoL will be assessed using the following instruments: EQ-5D-5L, EORTC QLQ-C30, BPI-SF
● improve health economy</td></tr>
<tr><td>To identify additional predictive and prognostic biomarkers</td><td>To identify additional predictive and prognostic biomarkers</td></tr>
<tr><td>Identify a certain treatment that is superior for a certain biomarker signature compared to other treatments (efficacy)</td><td>Identify a certain treatment class that is superior for a certain biomarker signature compared to other treatments classes (efficacy)</td></tr>
</table>

Identify superior treatment combinations (i.e. is treatment A followed by treatment B superior to treatment B followed by treatment A given a biomarker signature)	Identify superior treatment sequencing regimens (i.e. is treatment A followed by treatment B superior to treatment B followed by treatment A given a biomarker signature)
To determine whether treatment choices based on a biomarker signature, compared to standard of care, does not increase toxicity	To determine whether treatment choices based on a biomarker signature, compared to standard of care, does not increase toxicity
**Biomarker signatures used for randomisation:**<br>*The initial pre-defined biomarker signatures are defined as tumour properties or mutations in certain genes/pathways identified as potentially important in prostate cancer treatment response. These encompass the androgen receptor (AR), TP53, Homologous Recombination Repair deficiency (HRD) and the TMPRSS2-ERG fusion. Depending on the disease stage at trial entry (i.e. de novo mHSPC and mCRPC), the AR biomarker will be approached differently. AR perturbations are uncommon in mHSPC and emerge later during the course of the disease due to the selection pressure of androgen deprivation therapy. Thus, the biomarker signature TP53 wild-type will be applied in the mHSPC setting whereas TP53/AR wildtype will be investigated for mCRPC.*	
Androgen receptor alterations<br>HRR-repair deficiency - HRD<br>TP53 inactivation<br>TMPRSS2-ERG gene fusion	Androgen receptor alterations<br>HRR-repair deficiency - HRD<br>TP53 inactivation<br>TMPRSS2-ERG gene fusion<br>Other biomarker signatures upon drug availability and protocol amendment, such as:<br>TMB-H/MSI+/CDK12-<br>PI3K pathway alterations
**Blinding of biomarker signatures**<br>*Patients and treating physicians will be blinded to biomarker signature, both in the experimental- and control arms, but not to study drug. One exception is if the genomic analysis identifies a germline mutation in high penetrance genes, that are associated with hereditary cancer syndrome, such as:*	

<table>
<tr><td>BRCA1 and BRCA2 mutations

After consultation with the DSMB of the ProBio study, it was recommended to the study team to disclose detection of somatic alterations in the BRCA1/2 genes upon 2nd progression in the mCRPC setting and to immediately disclose MSI+ status at screening to the treating physician.</td><td>(encompassing but not limited to) APC, BRCA1, BRCA2, CDH1, CDK4, CDKN2A, DICER1, MET, MLH1, MSH2, MSH6, PMS2, MSH3, PALB2, POLD1, POLE, PTEN, RB1 and TP53.

This information might be important for not only the patients but also his relatives. Upon detection of any pathogenic or clinically relevant germline events, the mutational status of the gene(s) will be disclosed immediately to the treating physician, who is then responsible to initiate a genetic consult, upon discussion and consent from the patient.

In case of detection of somatic alterations in the BRCA1/2 genes, the mutational status will be disclosed upon 2nd progression in the mCRPC setting.

An MSI+ status at screening will be disclosed immediately to the treating physician.</td></tr>
<tr><td colspan="2">Randomisation

Patients who fulfil all inclusion criteria, none of the exclusion criteria, and where all data is available for randomisation will be randomised to either control arm (standard of care) or one of the experimental arms.</td></tr>
<tr><td></td><td>Patients in the experimental arm can be randomised to the following treatment classes, depending on national guidelines, availability and reimbursement criteria:</td></tr>
<tr><td colspan="2">For mHSPC</td></tr>
<tr><td>N/A</td><td>AR signaling inhibitors (ARSi)
● Abiraterone acetate plus prednisone
● Apalutamide
● Other ARSi upon approval from authorities and protocol amendment

Taxane-based chemotherapy
● Docetaxel

Other investigational agent(s) sponsored by pharmaceutical company
● Niraparib + abiraterone acetate plus predisone

Other agents upon sub-protocol amendment, e.g.
● Checkpoint inhibitor
● PI3K pathway inhibitor
● PSMA-targeted therapy</td></tr>
<tr><td colspan="2">For mCRPC</td></tr>
</table>

• Enzalutamide<br>• Abiraterone<br>• Cabazitaxel<br>• Docetaxel<br>• Carboplatin	AR signaling inhibitors (ARSi)<br>• Enzalutamide<br>• Abiraterone acetate plus prednisone<br>Taxane-based chemotherapy<br>• Cabazitaxel<br>• Docetaxel<br>Platinum based chemotherapy<br>• Carboplatin<br>Other investigational agents sponsored by pharma:<br>• Niraparib + abiraterone acetate plus prednisone<br>Other agents upon protocol amendment:<br>• Checkpoint inhibitor<br>• PI3K signalling inhibitor<br>• PSMA-targeted therapy
**Patient sample collection for routine laboratory measurements:**	
One 2-4 ml EDTA blood type to analyze:<br>• haemoglobin, platelet count, white blood cells including neutrophil count	One 2-4 ml EDTA blood tube to analyze:<br>• Haemoglobin<br>• CBC (platelet, red blood cells, total white blood cells, absolute neutrophil, lymphocyte and monocyte counts.
One 2-4 ml lithium heparin blood tube to analyze:<br>PSA, creatinine, albumin, LDH, ALP, ALT and AST.	One 2-4 ml lithium heparin blood tube to analyze:<br>testosterone, PSA, creatinine, albumin, bilirubin, LDH, ALP, ALT and AST.
**Sample collection for liquid and/or tissue biobanking for molecular profiling:**	
Genomic profiling of cell-free DNA and germline DNA - Blood will be collected in 2 x 10 ml Streck Cell-Free DNA BCT tubes.	Genomic profiling of cell-free DNA and germline DNA: Blood will be collected in 2 x 10 ml preservative tubes such as Streck Cell-Free DNA BCT tubes.
3 x 10 ml blood for exploratory objectives will be collected in either Streck cell-free DNA BCT, EDTA tubes or selected tubes tailored for the exploratory objectives.	3 x 10 ml blood for exploratory objectives will be collected in either preservative tubes such as Streck cell-free DNA BCT, EDTA tubes or selected tubes tailored for the exploratory objectives
Other specific samples can be collected in accordance with local/regional standard of care procedures. 25-50 mL of first-catch urine will be collected in designated collection cups for exploratory retrospective biomarkers studies.	Other specific samples can be collected in accordance with local/regional standard of care procedures. 25-50 mL of first-catch urine will be collected in designated collection cups for exploratory retrospective biomarkers studies.

	Diagnostic prostate biopsies from men with de novo mHSPC. 4 sections x 10 µm section thickness, >2 mm total length of cancer. Biopsies with <50% need to be macro dissected
**Inclusion criteria**	
Distant metastatic disease documented by positive bone scan or metastatic lesions on CT or MRI. Radiology taken within 6 weeks of inclusion may be used, if older a new scan needs to be taken.	Distant metastatic disease documented by positive bone scan or metastatic lesions on CT or MRI. Radiology taken within 6 weeks of inclusion may be used, if older a new scan needs to be taken. With the advent of novel imaging modalities using radionuclides, e.g. 68Ga-PSMA-11 PET/CT, the ProBio trial will allow for future incorporation of these imaging modalities upon availability of validated guidelines, progression criteria and protocol amendment. Until then novel imaging cannot be used for inclusion and response/progression endpoints.
Adequate health as assessed by the investigator to receive all available treatments in the trial<br><br>Adequate organ and bone-marrow function, i.e. haemoglobin ≥ 100 g/L (blood transfusion not less than 21 days prior to screening), absolute neutrophil count ≥ 1.5 x 109/L, platelets ≥100 x 109/L and Total bilirubin < 1.5 x ULN (patients with Gilberts Syndrome bilirubin < 40 µg/L) and AST and ALT ≤ 1.5 x ULN (or ≤ 3 x ULN in the presence of liver metastases) and serum creatinine not greater than 1 x ULN (if serum creatinine is between 1 and 1.5 x ULN, patients may be eligible provided that the calculated GFR is at least 50 ml/min using Cockcroft-Gault method)<br>Albumin greater than or equal to 28 g/L	Adequate health, hematologic, hepatic, and renal function, as assessed by the investigator, to receive all available treatments in the trial in each disease state (mHSPC and mCRPC)(i.e. haemoglobin ≥ 100 g/L (blood transfusion not less than 21 days prior to screening), absolute neutrophil count ≥ 1.5 x 10^9/L, platelets ≥100 x 10^9/L and Total bilirubin < 1.5 ULN (patients with Gilberts Syndrome bilirubin < 40 µg/L) and AST and ALT ≤ 1.5 ULN (or ≤ 3 ULN in the presence of liver metastases) and serum creatinine not greater than 1 ULN (if serum creatinine is between 1 and 1.5 ULN, patients may be eligible provided that the calculated GFR is at least 50 ml/min measured directly by 24-hour urine sampling OR using Cockcroft-Gault method)<br><br>Albumin greater than or equal to 28 l/L
	Agrees to use an effective contraceptive method during and up to 6 months after study drug treatment, and should not donate sperm during this period.
ECOG/WHO performance score 0-2	ECOG/WHO performance score 0-2
Able to understand the patient information and sign written informed consent	Able to understand the patient information and sign written informed consent
**Exclusion criteria**	

	The determination of a biomarker signature is necessary to randomise patients during ProBio. Patients will therefore be excluded in case of:<br>● For patients with mCRPC: undetectable levels of ctDNA.<br>● For patients with mHSPC: failure to detect ctDNA or somatic alterations from the primary tumour biopsies
Other malignancies within 5 years except non-melanoma skin cancer	Other malignancies within 5 years except non-melanoma skin cancer
Within 6 months of randomisation: myocardial infarction, unstable angina, angioplasty, bypass surgery, stroke, TIA, or congestive heart failure NYHA class III or IV	Within 6 months of randomisation: myocardial infarction, unstable angina, angioplasty, bypass surgery, stroke, TIA, or congestive heart failure NYHA class III or IV
Uncontrolled hypertension (systolic blood pressure ≥160 mmHg or diastolic BP ≥95 mmHg). Subjects with a history of hypertension are allowed provided blood pressure is controlled by anti-hypertensive treatment	Uncontrolled hypertension. Subjects with a history of hypertension are allowed provided blood pressure is controlled by anti-hypertensive treatment
Received more than two of the study treatments included in the ProBio study, prior to study inclusion, for the CRPC indication	Upon entering the mHSPC phase of the trial, prior systemic therapy (including ADT) is not allowed. Patients with mCRPC may not enter the trial when they have already received a 1st line systemic therapy for mCRPC
Any severe acute or chronic medical condition that places the patient at increased risk of serious toxicity or interferes with the interpretation of study results	Any severe acute or chronic medical condition that places the patient at increased risk of serious toxicity or interferes with the interpretation of study results
Unable to comply with study procedures	Unable to comply with study procedures
Current participation in another clinical trial that will be in conflict with the present study, administration of an investigational therapeutic or invasive surgical procedure within 28 days prior to study enrolment	Current participation in another clinical trial that will be in conflict with the present study, administration of an investigational therapeutic or invasive surgical procedure within 28 days prior to study enrolment

Patients who are unlikely to comply with the protocol (e.g. uncooperative attitude, inability to return for subsequent visits) and/or otherwise considered by the Investigator to be unlikely to complete the study	Patients who are unlikely to comply with the protocol (e.g. uncooperative attitude, inability to return for subsequent visits) and/or otherwise considered by the Investigator to be unlikely to complete the study
Any condition or situation which, in the opinion of the investigator, would put the subject at risk, may confound study results, or interfere with the subject’s participation in this study	Any condition or situation which, in the opinion of the investigator, would put the subject at risk, may confound study results, or interfere with the subject’s participation in this study
Any medical condition that would make use of the study treatments contraindicated, according to the SmPC, e.g. significant heart or liver disease. The investigator should check the SmPC and/or IB for the assigned study treatments.	Any medical condition that would make use of the study treatments contraindicated, according to the SmPC, e.g. significant heart or liver disease. The investigator should check the SmPC and/or IB for the assigned study treatments.
**Study procedures**	
Patients entering the mHSPC platform:	
**N/A**	Visit 1 (Screening phase 1):<br>Firstly, men with suspicion of metastatic prostate cancer will be invited to donate blood for biomarker research and to potentially participate in the ProBio trial. The purpose of this phase in the screening procedure is to identify patients that are eligible for the study, draw blood for routine clinical laboratory analysis and biobanking *prior to initiating androgen deprivation therapy (ADT).*<br>Written informed consent for blood collection and biobanking will be obtained.
**N/A**	Visit 2 (Screening phase 2):<br>After diagnosis and staging is done and if metastatic disease is detected by imaging, the patient will be asked to participate in the study. The Investigator will explain to the patient the nature and aim of the study, its procedures, possible side effects, requirements and restrictions. The patient will sign the second written informed consent before any study related procedures are performed. The patient’s biobanked plasma, tissue and germline DNA samples prior to ADT are retrieved from the biobank and processed for biomarker signature analysis and clinical characteristics and data are collected.
**N/A**	Schedule of activities in mHSPC, please see Study protocol.

Patients entering the mCRPC platform:	
Visit 1 (Screening):<br>The purpose of visit 1 is to select patients that are eligible for the study, obtain informed consent, draw blood for routine clinical laboratory analysis, genomic analysis, biobanking and record patient information.	Visit 1 (Screening):<br>The purpose of visit 1 is to select patients that are eligible for the study, obtain informed consent, draw blood for routine clinical laboratory analysis, genomic analysis, biobanking and record patient information. These eligible patients may enter the mCRPC platform from ProBio directly when starting 1st line systemic therapy (regardless of prior disease history), or may enter upon reaching mCRPC after being treated in the mHSPC platform from ProBio.
Schedule of activities as described in study protocol.	Schedule of activities are the same as in protocol v.3.0.
**Study design**	
outcome-adaptive, multi-arm, open-label, multiple assignment randomised biomarker driven platform trial in patients with metastatic castrate resistant prostate cancer.	outcome-adaptive, multi-arm, open-label, multiple assignment randomised biomarker driven platform trial in patients with metastatic hormone-sensitive and castration-resistant prostate cancer
**Duration of treatment**<br>The duration of treatment will last until the patient:	
NA	For mHSPC: develops castration-resistant disease, as defined by EAU guidelines
No longer has a clinical benefit from the treatment defined as clinical and/or radiological progression of the disease or intolerable toxicity.	For mCRPC: upon evaluation of the biochemical, clinical and/or radiological progression of the disease or intolerable toxicity, indication that the patient no longer has a clinical benefit from the treatment, as defined by PCWG3 criteria.
**Duration of patient´s involvement in the trial:**	
Treatment will continue until disease progression, withdrawal of consent, or the occurrence of unacceptable toxicity. Patients can be re-assigned to another treatment within the study based on the SMART (Sequential, Multiple Assignment, Randomised Trial) concept.	Treatment will continue until disease progression, withdrawal of consent, or the occurrence of unacceptable toxicity. Patients can be reassigned to another treatment within the study based on the SMART (Sequential, Multiple Assignment, Randomised Trial) concept two additional times for a maximum of three randomisations.

**Sample Size / number of patients planned:**	
Number of patients planned: 750	ProBio is a platform trial, and new arms may be added to the platform throughout the course of the trial. Therefore, there is no maximum number of patients planned. Each signature-treatment class combination may receive a maximum of 300 and 150 patients in the mHSPC and the mCRPC setting, respectively.
**Statistical considerations**	
All data will be presented using descriptive statistics. Results will be presented in total and by treatment group. Continuous variables will be summarized using mean, standard deviation, inter quartile range, median, minimum and maximum. Categorical variables will be summarized using the number and percentage of patients.	All data will be presented using descriptive statistics. Results will be presented in total, by treatment class, and across biomarker signatures. Continuous variables will be summarized using measures of central tendency and variability. Categorical variables will be summarized using absolute and relative frequencies.
Randomisation between assignment to the control arm or the biomarker driven arm will be stratified on biomarker signatures, previous treatment, and fraction of ctDNA and will therefore occur after the results from the ctDNA profiling is obtained. Patients with too little tumor burden to permit ctDNA profiling will still be randomised and treated within the study, initially without access to biomarker signature information. As these patients' disease develop, tumor burden will increase and permit identification of somatic alterations, after which treatment will be assigned based on biomarker signature in the biomarker driven arm.<br>Randomisation to the control arm vs. to the biomarker driven arms will be done within each signature according to a 1:1 ratio between the controls (for that signature) and the largest treatment group within the experimental arm.	Randomisation between assignment to the control arm or the biomarker driven arms will depend upon the patient's biomarker subgroup combination (i.e. the combination of the selected binary biomarkers shown in Table 6) and treatment history, and will therefore occur after the results from the ctDNA profiling is obtained. Patients with too little tumor burden to permit ctDNA profiling will be excluded from the ProBio study and will not be randomised.<br>Randomisation to the control arm vs. to the biomarker driven arms will be done within each biomarker subgroup combination. The randomisation algorithm is designed so that the probability of being randomised to the control group is equal to the largest of the probabilities of receiving an active treatment (this is true for any biomarker subgroup combination).
Interim analyses and data monitoring:<br>This is a Bayesian outcome-adaptive trial and both safety- and efficacy endpoints will be continuously evaluated. Early stopping is defined by study treatment arms reaching pre-specified criteria	Interim analyses and data monitoring:<br>This is a Bayesian outcome-adaptive trial and both safety- and efficacy endpoints will be continuously evaluated. The accumulated outcome data will be analyzed monthly to evaluate treatments and to update the randomisation probabilities. If any of the stopping rules is met (graduation or futility), we will provide detailed results to the DSMB that will decide about early stopping a specific arm in the trial. Early stopping is defined by study treatment arms reaching pre-specified criteria.

**PROMs (patient reported outcomes)**	
EQ-5D-5L	EQ-5D-5L
EORTC QLQ-C30	EORTC QLQ-C30
	BPI-SF (Brief Pain Inventory -Short Form)
**Exploratory objectives**	
Can ctDNA fraction dynamics replace PCWG3 for therapy response assessment	Can ctDNA fraction dynamics replace radiographic and biochemical response evaluation for therapy response assessment?
Can ctDNA fraction bursts predict therapy response?	Can ctDNA fraction bursts predict therapy response?
Retrospective analysis of the ctDNA profile to identify new biomarker signature- treatment associations	Retrospective analysis of the gene panel profile to identify new biomarker signature- treatment associations
	Analysis of cell-free DNA methylomes
Analysis of cell-free RNA	Analysis of cell-free RNA
RNA analysis of thrombocytes	RNA analysis of thrombocytes
Prospective DNA analysis of CTCs	Prospective DNA analysis of CTCs
RNA analysis of CTCs	RNA analysis of CTCs
	Prospective evaluation of clinical validity of PSMA-PET/CT-scan in 1st line mCRPC (CUTR-01 observational cohort study, PI: B. Sautois, CHU Liège, Belgium)
	Development of a new patient-reported outcome measure (PROM) instrument to evaluate the quality-of-life (QoL) of patients with advanced prostate cancer

APPENDIX 8: REFERENCES

Abida, Wassim, David Campbell, Akash Patnaik, Jeremy D. Shapiro, Brieuc Sautois, Nicholas J. Vogelzang, Eric G. Voog, et al. 2020. “Non-BRCA DNA Damage Repair Gene Alterations and Response to the PARP Inhibitor Rucaparib in Metastatic Castration-Resistant Prostate Cancer: Analysis from the Phase 2 TRITON2 Study.” Clinical Cancer Research. https://doi.org/10.1158/1078-0432.ccr-20-0394.

Abida, Wassim, Joanna Cyrta, Glenn Heller, Davide Prandi, Joshua Armenia, Ilsa Coleman, Marcin Cieslik, et al. 2019. “Genomic Correlates of Clinical Outcome in Advanced Prostate Cancer.” Proceedings of the National Academy of Sciences of the United States of America 116 (23): 11428–36.

Abida, Wassim, Michael L. Cheng, Joshua Armenia, Sumit Middha, Karen A. Autio, Hebert Alberto Vargas, Dana Rathkopf, et al. 2018. “Analysis of the Prevalence of Microsatellite Instability in Prostate Cancer and Response to Immune Checkpoint Blockade.” JAMA Oncology, December. https://doi.org/10.1001/jamaoncol.2018.5801.

Ali, S. A., A. Hoyle, N. D. James, C. Parker, C. Brawley, G. Attard, H. Douis, et al. 2019. “850PD - Benefit of Prostate Radiotherapy for Patients with Lymph Node Only or<4 Bone Metastasis and No Visceral Metastases: Exploratory Analyses of Metastatic Site and Number in the STAMPEDE ‘M1|RT Comparison.’” Annals of Oncology: Official Journal of the European Society for Medical Oncology / ESMO 30 (October): v330.

Alsop, Kathryn, Sian Fereday, Cliff Meldrum, Anna deFazio, Catherine Emmanuel, Joshy George, Alexander Dobrovic, et al. 2012. “BRCA Mutation Frequency and Patterns of Treatment Response in BRCA Mutation-Positive Women with Ovarian Cancer: A Report from the Australian Ovarian Cancer Study Group.” Journal of Clinical Oncology: Official Journal of the American Society of Clinical Oncology 30 (21): 2654–63.

André, Fabrice, Eva Ciruelos, Gabor Rubovszky, Mario Campone, Sibylle Loibl, Hope S. Rugo, Hiroji Iwata, et al. 2019. “Alpelisib for PIK3CA-Mutated, Hormone Receptor–Positive Advanced Breast Cancer.” The New England Journal of Medicine 380 (20): 1929–40.

Annala, Matti, Gillian Vandekerkhove, Daniel Khalaf, Sinja Taavitsainen, Kevin Beja, Evan W. Warner, Katherine Sunderland, et al. 2018. “Circulating Tumor DNA Genomics Correlate with Resistance to Abiraterone and Enzalutamide in Prostate Cancer.” Cancer Discovery, January. https://doi.org/10.1158/2159-8290.CD-17-0937.

Antonarakis, Emmanuel S., Changxue Lu, Brandon Luber, Chao Liang, Hao Wang, Yan Chen, John L. Silberstein, et al. 2018. “Germline DNA-Repair Gene Mutations and Outcomes in Men with Metastatic Castration-Resistant Prostate Cancer Receiving First-Line Abiraterone and Enzalutamide.” European Urology, February. https://doi.org/10.1016/j.eururo.2018.01.035.

Antonarakis, Emmanuel S., Changxue Lu, Brandon Luber, Hao Wang, Yan Chen, Yezi Zhu, John L. Silberstein, et al. 2017. “Clinical Significance of Androgen Receptor Splice Variant-7 mRNA Detection in Circulating Tumor Cells of Men With Metastatic Castration-Resistant Prostate Cancer Treated With First- and Second-Line Abiraterone and Enzalutamide.” Journal of Clinical Oncology: Official Journal of the American Society of Clinical Oncology 35 (19): 2149–56.

Antonarakis, Emmanuel S., Changxue Lu, Hao Wang, Brandon Luber, Mary Nakazawa, Jeffrey C. Roeser, Yan Chen, et al. 2014. “AR-V7 and Resistance to Enzalutamide and Abiraterone in Prostate Cancer.” The New England Journal of Medicine 371 (11): 1028–38.

Armstrong, Andrew J., Mohammed Al-Adhami, Ping Lin, Teresa Parli, Jennifer Sugg, Joyce Steinberg, Bertrand Tombal, et al. 2019. “Association Between New Unconfirmed Bone Lesions and Outcomes in Men With Metastatic Castration-Resistant Prostate Cancer Treated With Enzalutamide: Secondary Analysis of the PREVAIL

and AFFIRM Randomized Clinical Trials.” JAMA Oncology, December. https://doi.org/10.1001/jamaoncol.2019.4636.

Armstrong, Andrew J., Russell Z. Szmulewitz, Daniel P. Petrylak, Jeffrey Holzbeierlein, Arnauld Villers, Arun Azad, Antonio Alcaraz, et al. 2019. “ARCHES: A Randomized, Phase III Study of Androgen Deprivation Therapy With Enzalutamide or Placebo in Men With Metastatic Hormone-Sensitive Prostate Cancer.” Journal of Clinical Oncology: Official Journal of the American Society of Clinical Oncology 37 (32): 2974–86.

Armstrong, Andrew J., Susan Halabi, Jun Luo, David M. Nanus, Paraskevi Giannakakou, Russell Z. Szmulewitz, Daniel C. Danila, et al. 2019. “Prospective Multicenter Validation of Androgen Receptor Splice Variant 7 and Hormone Therapy Resistance in High-Risk Castration-Resistant Prostate Cancer: The PROPHECY Study.” Journal of Clinical Oncology: Official Journal of the American Society of Clinical Oncology 37 (13): 1120–29.

Asaoka, Yoshinari, Hideaki Ijichi, and Kazuhiko Koike. 2015. “PD-1 Blockade in Tumors with Mismatch-Repair Deficiency.” The New England Journal of Medicine.

Attard, Gerhardt, Michael Borre, Howard Gurney, Yohann Loriot, Corina Andresen-Daniil, Ranjith Kalleda, Trinh Pham, Mary-Ellen Taplin, and on behalf of the PLATO collaborators. 2018. “Abiraterone Alone or in Combination With Enzalutamide in Metastatic Castration-Resistant Prostate Cancer With Rising Prostate-Specific Antigen During Enzalutamide Treatment.” Journal of Clinical Oncology. https://doi.org/10.1200/jco.2018.77.9827.

Barbieri, Christopher E., Arul M. Chinnaiyan, Seth P. Lerner, Charles Swanton, and Mark A. Rubin. 2017. “The Emergence of Precision Urologic Oncology: A Collaborative Review on Biomarker-Driven Therapeutics.” European Urology 71 (2): 237–46.

Beer, Tomasz M., Andrew J. Armstrong, Dana E. Rathkopf, Yohann Loriot, Cora N. Sternberg, Celestia S. Higano, Peter Iversen, et al. 2014. “Enzalutamide in Metastatic Prostate Cancer before Chemotherapy.” The New England Journal of Medicine 371 (5): 424–33.

Beer, Tomasz M., Andrew J. Armstrong, Dana Rathkopf, Yohann Loriot, Cora N. Sternberg, Celestia S. Higano, Peter Iversen, et al. 2017. “Enzalutamide in Men with Chemotherapy-Naïve Metastatic Castration-Resistant Prostate Cancer: Extended Analysis of the Phase 3 PREVAIL Study.” European Urology 71 (2): 151–54.

Beer, Tomasz M., Eugene D. Kwon, Charles G. Drake, Karim Fizazi, Christopher Logothetis, Gwenaelle Gravis, Vinod Ganju, et al. 2017. “Randomized, Double-Blind, Phase III Trial of Ipilimumab Versus Placebo in Asymptomatic or Minimally Symptomatic Patients With Metastatic Chemotherapy-Naive Castration-Resistant Prostate Cancer.” Journal of Clinical Oncology: Official Journal of the American Society of Clinical Oncology 35 (1): 40–47.

Beltran, Himisha, Davide Prandi, Juan Miguel Mosquera, Matteo Benelli, Loredana Puca, Joanna Cyrta, Clarisse Marotz, et al. 2016. “Divergent Clonal Evolution of Castration-Resistant Neuroendocrine Prostate Cancer.” Nature Medicine 22 (3): 298–305.

Beltran, Himisha, Kenneth Eng, Juan Miguel Mosquera, Alexandros Sigaras, Alessandro Romanel, Hanna Rennert, Myriam Kossai, et al. 2015. “Whole-Exome Sequencing of Metastatic Cancer and Biomarkers of Treatment Response.” JAMA Oncology 1 (4): 466–74.

Berruti, Alfredo, Anna Pia, and Massimo Terzolo. 2011. “Abiraterone and Increased Survival in Metastatic Prostate Cancer.” The New England Journal of Medicine.

Best, Myron G., Nik Sol, Irsan Kooi, Jihane Tannous, Bart A. Westerman, François Rustenburg, Pepijn Schellen, et al. 2015. “RNA-Seq of Tumor-Educated Platelets Enables Blood-Based Pan-Cancer, Multiclass, and Molecular Pathway Cancer Diagnostics.” Cancer Cell 28 (5): 666–76.

Best, Myron G., Nik Sol, Sjors G. J. G. In ’t Veld, Adrienne Vancura, Mirte Muller, Anna-Larissa N. Niemeijer, Aniko V. Fejes, et al. 2017. “Swarm Intelligence-Enhanced Detection of Non-Small-Cell Lung Cancer Using Tumor-Educated Platelets.” Cancer Cell 32 (2): 238–52.e9.

Bono, J. S. de, U. De Giorgi, and D. N. Rodrigues. 2019. “Randomized Phase II Study Evaluating Akt Blockade with Ipatasertib, in Combination with Abiraterone, in Patients with Metastatic Prostate Cancer with and without ….” Clinical Cancer Research: An Official Journal of the American Association for Cancer Research. http://clincancerres.aacrjournals.org/content/25/3/928.abstract.

Bono, Johann de et al. 2020. “PROfound: Phase 3 Study of Olaparib versus Enzalutamide or Abiraterone for Metastatic Castration-Resistant Prostate Cancer (mCRPC) with Homologous... | OncologyPRO.” Accessed December 19, 2019b. https://oncologypro.esmo.org/Meeting-Resources/ESMO-2019-Congress/PROfound-Phase-3-study-of-olaparib-versus-enzalutamide-or-abiraterone-for-metastatic-castration-resistant-prostate-cancer-mCRPC-with-homologous-recombination-repair-HRR-gene-alterations.

Bono, Johann de, Joaquin Mateo, Karim Fizazi, Fred Saad, Neal Shore, Shahneen Sandhu, Kim N. Chi, et al. 2020. “Olaparib for Metastatic Castration-Resistant Prostate Cancer.” The New England Journal of Medicine, April. https://doi.org/10.1056/NEJMoa1911440.

Bono, Johann S. de, Johann S. de Bono, Simon Chowdhury, Susan Feyerabend, Tony Elliott, Enrique Grande, Amal Melhem-Bertrandt, et al. 2018. “Antitumour Activity and Safety of Enzalutamide in Patients with Metastatic Castration-Resistant Prostate Cancer Previously Treated with Abiraterone Acetate Plus Prednisone for ≥24 Weeks in Europe.” European Urology. https://doi.org/10.1016/j.eururo.2017.07.035.

Bono, Johann Sebastian de, Stephane Oudard, Mustafa Ozguroglu, Steinbjørn Hansen, Jean-Pascal Machiels, Ivo Kocak, Gwenaëlle Gravis, et al. 2010. “Prednisone plus Cabazitaxel or Mitoxantrone for Metastatic Castration-Resistant Prostate Cancer Progressing after Docetaxel Treatment: A Randomised Open-Label Trial.” The Lancet 376 (9747): 1147–54.

Bryant, Helen E., Niklas Schultz, Huw D. Thomas, Kayan M. Parker, Dan Flower, Elena Lopez, Suzanne Kyle, Mark Meuth, Nicola J. Curtin, and Thomas Helleday. 2005. “Specific Killing of BRCA2-Deficient Tumours with Inhibitors of poly(ADP-Ribose) Polymerase.” Nature 434 (7035): 913–17.

Burdett, Sarah, Liselotte M. Boevé, Fiona C. Ingleby, David J. Fisher, Larysa H. Rydzewska, Claire L. Vale, George van Andel, et al. 2019. “Prostate Radiotherapy for Metastatic Hormone-Sensitive Prostate Cancer: A STOPCAP Systematic Review and Meta-Analysis.” European Urology 76 (1): 115–24.

Cabel, Luc, Elika Loir, Gwenaelle Gravis, Pernelle Lavaud, Christophe Massard, Laurence Albiges, Giulia Baciarello, Yohann Loriot, and Karim Fizazi. 2017. “Long-Term Complete Remission with Ipilimumab in Metastatic Castrate-Resistant Prostate Cancer: Case Report of Two Patients.” Journal for Immunotherapy of Cancer 5 (April): 31.

Cancer Genome Atlas Research Network. 2015. “The Molecular Taxonomy of Primary Prostate Cancer.” Cell 163 (4): 1011–25.

Carreira, Suzanne, Alessandro Romanel, Jane Goodall, Emily Grist, Roberta Ferraldeschi, Susana Miranda, Davide Prandi, et al. 2014. “Tumor Clone Dynamics in Lethal Prostate Cancer.” Science Translational Medicine 6 (254): 254ra125.

Chen, William S., Rahul Aggarwal, Li Zhang, Shuang G. Zhao, George V. Thomas, Tomasz M. Beer, David A. Quigley, et al. 2019. “Genomic Drivers of Poor Prognosis and Enzalutamide Resistance in Metastatic Castration-Resistant Prostate Cancer.” European Urology 76 (5): 562–71.

Cheng, Heather H., Colin C. Pritchard, Thomas Boyd, Peter S. Nelson, and Bruce Montgomery. 2016. “Biallelic Inactivation of BRCA2 in Platinum-Sensitive Metastatic Castration-Resistant Prostate Cancer.” European Urology 69 (6): 992–95.

Chi, Kim N., Neeraj Agarwal, Anders Bjartell, Byung Ha Chung, Andrea J. Pereira de Santana Gomes, Robert Given, Álvaro Juárez Soto, et al. 2019. “Apalutamide for Metastatic, Castration-Sensitive Prostate Cancer.” The New England Journal of Medicine 381 (1): 13–24.

Chung, Jon H., Ninad Dewal, Ethan Sokol, Paul Mathew, Robert Whitehead, Sherri Z. Millis, Garrett M. Frampton, et al. 2019. “Prospective Comprehensive Genomic Profiling of Primary and Metastatic Prostate Tumors.” JCO Precision Oncology 3 (May). https://doi.org/10.1200/PO.18.00283.

Clarke, N. W., A. Ali, F. C. Ingleby, A. Hoyle, C. L. Amos, G. Attard, C. D. Brawley, et al. 2019. “Addition of Docetaxel to Hormonal Therapy in Low- and High-Burden Metastatic Hormone Sensitive Prostate Cancer: Long-Term Survival Results from the STAMPEDE Trial.” Annals of Oncology: Official Journal of the European Society for Medical Oncology / ESMO 30 (12): 1992–2003.

Clarke, Noel, Pawel Wiechno, Boris Alekseev, Nuria Sala, Robert Jones, Ivo Kocak, Vincenzo Emanuele Chiuri, et al. 2018. “Olaparib Combined with Abiraterone in Patients with Metastatic Castration-Resistant Prostate Cancer: A Randomised, Double-Blind, Placebo-Controlled, Phase 2 Trial.” The Lancet Oncology 19 (7): 975–86.

Clinseq Team. n.d. “Autoseq - Autoseq.” Accessed October 2, 2020. https://autoseq-docs.readthedocs.io.

Cornford, Philip, Joaquim Bellmunt, Michel Bolla, Erik Briers, Maria De Santis, Tobias Gross, Ann M. Henry, et al. 2017. “EAU-ESTRO-SIOG Guidelines on Prostate Cancer. Part II: Treatment of Relapsing, Metastatic, and Castration-Resistant Prostate Cancer.” European Urology 71 (4): 630–42.

Davis, I. D., A. J. Martin, M. R. Stockler, S. Begbie, K. N. Chi, S. Chowdhury, X. Coskinas, et al. 2019. “ENZAMET Trial Investigators and the Australian and New Zealand Urogenital and Prostate Cancer Trials Group, Enzalutamide with Standard First-Line Therapy in Metastatic Prostate Cancer.” The New England Journal of Medicine 381: 121–31.

Davis, Ian D., Andrew J. Martin, Martin R. Stockler, Stephen Begbie, Kim N. Chi, Simon Chowdhury, Xanthi Coskinas, et al. 2019. “Enzalutamide with Standard First-Line Therapy in Metastatic Prostate Cancer.” The New England Journal of Medicine 381 (2): 121–31.

De Laere, Bram, Piet Ost, Henrik Grönberg, and Johan Lindberg. 2019. “Has the PROPHECY of AR-V7 Been Fulfilled?” Journal of Clinical Oncology: Official Journal of the American Society of Clinical Oncology 37 (24): 2181–82.

De Laere, Bram, Pieter-Jan van Dam, Tom Whitington, Markus Mayrhofer, Emanuela Henao Diaz, Gert Van den Eynden, Jean Vandebroek, et al. 2017. “Comprehensive Profiling of the Androgen Receptor in Liquid Biopsies

from Castration-Resistant Prostate Cancer Reveals Novel Intra-AR Structural Variation and Splice Variant Expression Patterns." European Urology 72 (2): 192–200.

De Laere, Bram, Steffi Oeyen, Markus Mayrhofer, Tom Whitington, Pieter-Jan van Dam, Peter Van Oyen, Christophe Ghysel, et al. 2018. "TP53 Outperforms Other Androgen Receptor Biomarkers to Predict Abiraterone or Enzalutamide Outcome in Metastatic Castration-Resistant Prostate Cancer." Clinical Cancer Research: An Official Journal of the American Association for Cancer Research, September. https://doi.org/10.1158/1078-0432.CCR-18-1943.

Del Re, Marzia, Elisa Biasco, Stefania Crucitta, Lisa Derosa, Eleonora Rofi, Cinzia Orlandini, Mario Miccoli, et al. 2017. "The Detection of Androgen Receptor Splice Variant 7 in Plasma-Derived Exosomal RNA Strongly Predicts Resistance to Hormonal Therapy in Metastatic Prostate Cancer Patients." European Urology 71 (4): 680–87.

Enderle, Daniel, Alexandra Spiel, Christine M. Coticchia, Emily Berghoff, Romy Mueller, Martin Schlumpberger, Markus Sprenger-Haussels, et al. 2015. "Characterization of RNA from Exosomes and Other Extracellular Vesicles Isolated by a Novel Spin Column-Based Method." PloS One 10 (8): e0136133.

Farmer, Hannah, Nuala McCabe, Christopher J. Lord, Andrew N. J. Tutt, Damian A. Johnson, Tobias B. Richardson, Manuela Santarosa, et al. 2005. "Targeting the DNA Repair Defect in BRCA Mutant Cells as a Therapeutic Strategy." Nature 434 (7035): 917–21.

Ferlay, J., M. Colombet, I. Soerjomataram, T. Dyba, G. Randi, M. Bettio, A. Gavin, O. Visser, and F. Bray. 2018. "Cancer Incidence and Mortality Patterns in Europe: Estimates for 40 Countries and 25 Major Cancers in 2018." European Journal of Cancer 103 (November): 356–87.

Ferraldeschi, Roberta, Daniel Nava Rodrigues, Ruth Riisnaes, Susana Miranda, Ines Figueiredo, Pasquale Rescigno, Praful Ravi, et al. 2015. "PTEN Protein Loss and Clinical Outcome from Castration-Resistant Prostate Cancer Treated with Abiraterone Acetate." European Urology 67 (4): 795–802.

Feyerabend, Susan, Fred Saad, Tracy Li, Tetsuro Ito, Joris Diels, Suzy Van Sanden, Peter De Porre, et al. 2018. "Survival Benefit, Disease Progression and Quality-of-Life Outcomes of Abiraterone Acetate plus Prednisone versus Docetaxel in Metastatic Hormone-Sensitive Prostate Cancer: A Network Meta-Analysis." European Journal of Cancer 103 (November): 78–87.

Fizazi, Karim, Howard I. Scher, Arturo Molina, Christopher J. Logothetis, Kim N. Chi, Robert J. Jones, John N. Staffurth, et al. 2012. "Abiraterone Acetate for Treatment of Metastatic Castration-Resistant Prostate Cancer: Final Overall Survival Analysis of the COU-AA-301 Randomised, Double-Blind, Placebo-Controlled Phase 3 Study." The Lancet Oncology 13 (10): 983–92.

Fizazi, Karim, Namphuong Tran, Luis Fein, Nobuaki Matsubara, Alfredo Rodriguez-Antolin, Boris Y. Alekseev, Mustafa Özgüroğlu, et al. 2017. "Abiraterone plus Prednisone in Metastatic, Castration-Sensitive Prostate Cancer." The New England Journal of Medicine 377 (4): 352–60.

Food, U. S., Drug Administration, and Others. 2017. "FDA Approves First Cancer Treatment for Any Solid Tumor with a Specific Genetic Feature." US Food and Drug Administration, Silver Spring. https://www.fda.gov/newsevents/newsroom/pressannouncements/ucm560167.htm.

Francini, Edoardo, Steven Yip, Shubidito Ahmed, Haocheng Li, Luke Ardolino, Carolyn P. Evan, Marina Kaymakcalan, et al. 2018. "Clinical Outcomes of First-Line Abiraterone Acetate or Enzalutamide for Metastatic Castration-Resistant Prostate Cancer After Androgen Deprivation Therapy Docetaxel or ADT Alone for

Metastatic Hormone-Sensitive Prostate Cancer." Clinical Genitourinary Cancer. https://doi.org/10.1016/j.clgc.2017.12.012.

Galletti, Giuseppe, Alexandre Matov, Himisha Beltran, Jacqueline Fontugne, Juan Miguel Mosquera, Cynthia Cheung, Theresa Y. MacDonald, et al. 2014. "ERG Induces Taxane Resistance in Castration-Resistant Prostate Cancer." Nature Communications 5 (November): 5548.

Goodall, Jane, Joaquin Mateo, Wei Yuan, Helen Mossop, Nuria Porta, Susana Miranda, Raquel Perez-Lopez, et al. 2017. "Circulating Cell-Free DNA to Guide Prostate Cancer Treatment with PARP Inhibition." Cancer Discovery 7 (9): 1006–17.

Gravis, Gwenaelle, Jean-Marie Boher, Florence Joly, Michel Soulié, Laurence Albiges, Franck Priou, Igor Latorzeff, et al. 2016. "Androgen Deprivation Therapy (ADT) plus Docetaxel versus ADT Alone in Metastatic Non Castrate Prostate Cancer: Impact of Metastatic Burden and Long-Term Survival Analysis of the Randomized Phase 3 GETUG-AFU15 Trial." European Urology 70 (2): 256–62.

Grossman Barton et al. 2003. "Neoadjuvant Chemotherapy plus Cystectomy Compared with Cystectomy Alone for Locally Advanced Bladder Cancer." 2003. The New England Journal of Medicine 349 (19): 1880–1880.

Gundem, Gunes, Peter Van Loo, Barbara Kremeyer, Ludmil B. Alexandrov, Jose M. C. Tubio, Elli Papaemmanuil, Daniel S. Brewer, et al. 2015. "The Evolutionary History of Lethal Metastatic Prostate Cancer." Nature 520 (7547): 353–57.

Gupta, Santosh, Jing Li, Gabor Kemeny, Rhonda L. Bitting, Joshua Beaver, Jason A. Somarelli, Kathryn E. Ware, Simon Gregory, and Andrew J. Armstrong. 2017. "Whole Genomic Copy Number Alterations in Circulating Tumor Cells from Men with Abiraterone or Enzalutamide-Resistant Metastatic Castration-Resistant Prostate Cancer." Clinical Cancer Research: An Official Journal of the American Association for Cancer Research 23 (5): 1346–57.

Heller, Glenn, Robert McCormack, Thian Kheoh, Arturo Molina, Matthew R. Smith, Robert Dreicer, Fred Saad, et al. 2018. "Circulating Tumor Cell Number as a Response Measure of Prolonged Survival for Metastatic Castration-Resistant Prostate Cancer: A Comparison With Prostate-Specific Antigen Across Five Randomized Phase III Clinical Trials." Journal of Clinical Oncology: Official Journal of the American Society of Clinical Oncology 36 (6): 572–80.

Henzler, Christine, Yingming Li, Rendong Yang, Terri McBride, Yeung Ho, Cynthia Sprenger, Gang Liu, et al. 2016. "Truncation and Constitutive Activation of the Androgen Receptor by Diverse Genomic Rearrangements in Prostate Cancer." Nature Communications 7 (November): 13668.

Herberts, Cameron, Andrew J. Murtha, Simon Fu, Gang Wang, Elena Schönlau, Hui Xue, Dong Lin, et al. 2020. "Activating AKT1 and PIK3CA Mutations in Metastatic Castration-Resistant Prostate Cancer." European Urology, May. https://doi.org/10.1016/j.eururo.2020.04.058.

Higano, Celestia. 2019. "Enzalutamide, Apalutamide, or Darolutamide: Are Apples or Bananas Best for Patients?" Nature Reviews Urology. https://doi.org/10.1038/s41585-019-0186-2.

Holmes, Michael G., Erik Foss, Gabby Joseph, Adam Foye, Brooke Beckett, Daria Motamedi, Jack Youngren, et al. 2017. "CT-Guided Bone Biopsies in Metastatic Castration-Resistant Prostate Cancer: Factors Predictive of Maximum Tumor Yield." Journal of Vascular and Interventional Radiology: JVIR 28 (8): 1073–81.e1.

Hong, Matthew K. H., Geoff Macintyre, David C. Wedge, Peter Van Loo, Keval Patel, Sebastian Lunke, Ludmil B. Alexandrov, et al. 2015. "Tracking the Origins and Drivers of Subclonal Metastatic Expansion in Prostate Cancer." Nature Communications 6 (April): 6605.

Hoyle, Alex P., Adnan Ali, Nicholas D. James, Adrian Cook, Christopher C. Parker, Johann S. de Bono, Gerhardt Attard, et al. 2019. "Abiraterone in 'High-' and 'Low-Risk' Metastatic Hormone-Sensitive Prostate Cancer." European Urology 76 (6): 719–28.

Husain, Hatim, Vladislava O. Melnikova, Karena Kosco, Brian Woodward, Soham More, Sandeep C. Pingle, Elizabeth Weihe, et al. 2017. "Monitoring Daily Dynamics of Early Tumor Response to Targeted Therapy by Detecting Circulating Tumor DNA in Urine." Clinical Cancer Research: An Official Journal of the American Association for Cancer Research 23 (16): 4716–23.

Hussain, Maha, Karim Fizazi, Fred Saad, Per Rathenborg, Neal Shore, Ubirajara Ferreira, Petro Ivashchenko, et al. 2018. "Enzalutamide in Men with Nonmetastatic, Castration-Resistant Prostate Cancer." The New England Journal of Medicine 378 (26): 2465–74.

Hussain, Maha, Stephanie Daignault-Newton, Przemyslaw W. Twardowski, Costantine Albany, Mark N. Stein, Lakshmi P. Kunju, Javed Siddiqui, et al. 2018. "Targeting Androgen Receptor and DNA Repair in Metastatic Castration-Resistant Prostate Cancer: Results From NCI 9012." Journal of Clinical Oncology: Official Journal of the American Society of Clinical Oncology 36 (10): 991–99.

Isaacsson Velho, Pedro Henrique, Fahad Qazi, Sayeedul Hasan, Michael Anthony Carducci, Mario A. Eisenberger, Phuoc T. Tran, and Emmanuel S. Antonarakis. 2018. "Efficacy of Radium-223 in Bone-Metastatic Prostate Cancer Patients with and without Homologous Repair Deficiency (HRD) Mutations." Journal of Clinical Orthodontics: JCO 36 (15_suppl): e17023–e17023.

Isaacsson Velho, Pedro, Fahad Qazi, Sayeedul Hassan, Michael A. Carducci, Samuel R. Denmeade, Mark C. Markowski, Daniel L. Thorek, et al. 2019. "Efficacy of Radium-223 in Bone-Metastatic Castration-Resistant Prostate Cancer with and Without Homologous Repair Gene Defects." European Urology 76 (2): 170–76.

James, Nicholas D., Johann S. de Bono, Melissa R. Spears, Noel W. Clarke, Malcolm D. Mason, David P. Dearnaley, Alastair W. S. Ritchie, et al. 2017. "Abiraterone for Prostate Cancer Not Previously Treated with Hormone Therapy." The New England Journal of Medicine 377 (4): 338–51.

James, Nicholas D., Matthew R. Sydes, Noel W. Clarke, Malcolm D. Mason, David P. Dearnaley, Melissa R. Spears, Alastair W. S. Ritchie, et al. 2016. "Addition of Docetaxel, Zoledronic Acid, or Both to First-Line Long-Term Hormone Therapy in Prostate Cancer (STAMPEDE): Survival Results from an Adaptive, Multiarm, Multistage, Platform Randomised Controlled Trial." The Lancet 387 (10024): 1163–77.

Karim Fizazi et al. 2019. "Abiraterone Acetate plus Prednisone in Patients with Newly Diagnosed High-Risk Metastatic Castration-Sensitive Prostate Cancer (LATITUDE): Final Overall Survival Analysis of a Randomised, Double-Blind, Phase 3 Trial." The Lancet Oncology. https://doi.org/10.1016/s1470-2045(19)30082-8.

Kelland, Lloyd. 2007. "The Resurgence of Platinum-Based Cancer Chemotherapy." Nature Reviews. Cancer 7 (8): 573–84.

Kentepozidis, N., A. Soultati, S. Giassas, N. Vardakis, A. Kalykaki, A. Kotsakis, E. Papadimitraki, N. Pantazopoulos, V. Bozionellou, and V. Georgoulias. 2012. "Paclitaxel in Combination with Carboplatin as Salvage Treatment in Patients with Castration-Resistant Prostate Cancer: A Hellenic Oncology Research Group Multicenter Phase II Study." Cancer Chemotherapy and Pharmacology. https://doi.org/10.1007/s00280-012-1896-9.

Khalaf, Daniel J., Matti Annala, Sinja Taavitsainen, Daygen L. Finch, Conrad Oja, Joanna Vergidis, Muhammad Zulfiqar, et al. 2019. "Optimal Sequencing of Enzalutamide and Abiraterone Acetate plus Prednisone in Metastatic Castration-Resistant Prostate Cancer: A Multicentre, Randomised, Open-Label, Phase 2, Crossover Trial." The Lancet Oncology. https://doi.org/10.1016/s1470-2045(19)30688-6.

Kohli, Manish, Winston Tan, Tiantian Zheng, Amy Wang, Carlos Montesinos, Calven Wong, Pan Du, et al. 2020. "Clinical and Genomic Insights into Circulating Tumor DNA-Based Alterations across the Spectrum of Metastatic Hormone-Sensitive and Castrate-Resistant Prostate Cancer." EBioMedicine 54 (April): 102728.

Kothari, Vishal, Iris Wei, Sunita Shankar, Shanker Kalyana-Sundaram, Lidong Wang, Linda W. Ma, Pankaj Vats, et al. 2013. "Outlier Kinase Expression by RNA Sequencing as Targets for Precision Therapy." Cancer Discovery 3 (3): 280–93.

Kwon, Eugene D., Charles G. Drake, Howard I. Scher, Karim Fizazi, Alberto Bossi, Alfons J. M. van den Eertwegh, Michael Krainer, et al. 2014. "Ipilimumab versus Placebo after Radiotherapy in Patients with Metastatic Castration-Resistant Prostate Cancer That Had Progressed after Docetaxel Chemotherapy (CA184-043): A Multicentre, Randomised, Double-Blind, Phase 3 Trial." The Lancet Oncology 15 (7): 700–712.

Kyriakopoulos, Christos E., Yu-Hui Chen, Michael A. Carducci, Glenn Liu, David F. Jarrard, Noah M. Hahn, Daniel H. Shevrin, et al. 2018. "Chemohormonal Therapy in Metastatic Hormone-Sensitive Prostate Cancer: Long-Term Survival Analysis of the Randomized Phase III E3805 CHAARTED Trial." Journal of Clinical Oncology: Official Journal of the American Society of Clinical Oncology 36 (11): 1080–87.

Laere, Bram De, Bram De Laere, Prabhakar Rajan, Henrik Grönberg, Luc Dirix, Johan Lindberg, and for the CORE-ARV-CTC and ProBIO Investigators. 2019. "Androgen Receptor Burden and Poor Response to Abiraterone or Enzalutamide in TP53 Wild-Type Metastatic Castration-Resistant Prostate Cancer." JAMA Oncology. https://doi.org/10.1001/jamaoncol.2019.0869.

Lallous, Nada, Stanislav V. Volik, Shannon Awrey, Eric Leblanc, Ronnie Tse, Josef Murillo, Kriti Singh, et al. 2016. "Functional Analysis of Androgen Receptor Mutations That Confer Anti-Androgen Resistance Identified in Circulating Cell-Free DNA from Prostate Cancer Patients." Genome Biology 17 (January): 10.

Lavaud, Pernelle, Gwenaëlle Gravis, Stéphanie Foulon, Florence Joly, Stéphane Oudard, Frank Priou, Igor Latorzeff, et al. 2018. "Anticancer Activity and Tolerance of Treatments Received Beyond Progression in Men Treated Upfront with Androgen Deprivation Therapy With or Without Docetaxel for Metastatic Castration-Naïve Prostate Cancer in the GETUG-AFU 15 Phase 3 Trial." European Urology 73 (5): 696–703.

Le, Dung T., Jennifer N. Durham, Kellie N. Smith, Hao Wang, Bjarne R. Bartlett, Laveet K. Aulakh, Steve Lu, et al. 2017. "Mismatch Repair Deficiency Predicts Response of Solid Tumors to PD-1 Blockade." Science 357 (6349): 409–13.

Lee, Lisa, Siraj Ali, Elizabeth Genega, Dallas Reed, Ethan Sokol, and Paul Mathew. 2018. "Aggressive-Variant Microsatellite-Stable POLE Mutant Prostate Cancer With High Mutation Burden and Durable Response to Immune Checkpoint Inhibitor Therapy." JCO Precision Oncology. https://doi.org/10.1200/po.17.00097.

Liu, David, Elizabeth R. Plimack, Jean Hoffman-Censits, Levi A. Garraway, Joaquim Bellmunt, Eliezer Van Allen, and Jonathan E. Rosenberg. 2016. "Clinical Validation of Chemotherapy Response BiomarkerERCC2in Muscle-Invasive Urothelial Bladder Carcinoma." JAMA Oncology. https://doi.org/10.1001/jamaoncol.2016.1056.

Liu, Wennuan, Sari Laitinen, Sofia Khan, Mauno Vihinen, Jeanne Kowalski, Guoqiang Yu, Li Chen, et al. 2009. "Copy Number Analysis Indicates Monoclonal Origin of Lethal Metastatic Prostate Cancer." Nature Medicine 15 (5): 559–65.

Liu, X. Shirley, and Elaine R. Mardis. 2017. "Applications of Immunogenomics to Cancer." Cell 168 (4): 600–612.

Lohr, Jens G., Viktor A. Adalsteinsson, Kristian Cibulskis, Atish D. Choudhury, Mara Rosenberg, Peter Cruz-Gordillo, Joshua M. Francis, et al. 2014. "Whole-Exome Sequencing of Circulating Tumor Cells Provides a Window into Metastatic Prostate Cancer." Nature Biotechnology 32 (5): 479–84.

Lombard, Alan P., Chengfei Liu, Cameron M. Armstrong, Vito Cucchiara, Xinwei Gu, Wei Lou, Christopher P. Evans, and Allen C. Gao. 2017. "ABCB1 Mediates Cabazitaxel-Docetaxel Cross-Resistance in Advanced Prostate Cancer." Molecular Cancer Therapeutics 16 (10): 2257–66.

Lorente, David, Aurelius Omlin, Zafeiris Zafeiriou, Daniel Nava-Rodrigues, Raquel Pérez-López, Carmel Pezaro, Niven Mehra, et al. 2016. "Castration-Resistant Prostate Cancer Tissue Acquisition From Bone Metastases for Molecular Analyses." Clinical Genitourinary Cancer 14 (6): 485–93.

Lorente, David, David Olmos, Joaquin Mateo, Diletta Bianchini, George Seed, Martin Fleisher, Daniel C. Danila, et al. 2016. "Decline in Circulating Tumor Cell Count and Treatment Outcome in Advanced Prostate Cancer." European Urology 70 (6): 985–92.

Magbanua, Mark Jesus M., Eduardo V. Sosa, Ritu Roy, Lauren E. Eisenbud, Janet H. Scott, Adam Olshen, Dan Pinkel, Hope S. Rugo, and John W. Park. 2013. "Genomic Profiling of Isolated Circulating Tumor Cells from Metastatic Breast Cancer Patients." Cancer Research 73 (1): 30–40.

Mailankody, Sham, and Vinay Prasad. 2015. "Five Years of Cancer Drug Approvals: Innovation, Efficacy, and Costs." JAMA Oncology 1 (4): 539–40.

Mainwaring, P. N., L. Zhang, S. D. Mundle, K. Liu, E. Pollozi, A. Gray, and M. Wildgust. 2019. "488PCorrelation of Progression Free Survival-2 and Overall Survival in Solid Tumours." Annals of Oncology: Official Journal of the European Society for Medical Oncology / ESMO 30 (Supplement_5). https://doi.org/10.1093/annonc/mdz244.050.

Mateo, Joaquin, George Seed, Claudia Bertan, Pasquale Rescigno, David Dolling, Ines Figueiredo, Susana Miranda, et al. 2020. "Genomics of Lethal Prostate Cancer at Diagnosis and Castration Resistance." The Journal of Clinical Investigation 130 (4): 1743–51.

Mateo, Joaquin, Heather H. Cheng, Himisha Beltran, David Dolling, Wen Xu, Colin C. Pritchard, Helen Mossop, et al. 2018. "Clinical Outcome of Prostate Cancer Patients with Germline DNA Repair Mutations: Retrospective Analysis from an International Study." European Urology, January. https://doi.org/10.1016/j.eururo.2018.01.010.

Mateo, Joaquin, Nuria Porta, Diletta Bianchini, Ursula McGovern, Tony Elliott, Robert Jones, Isabel Syndikus, et al. 2019. "Olaparib in Patients with Metastatic Castration-Resistant Prostate Cancer with DNA Repair Gene Aberrations (TOPARP-B): A Multicentre, Open-Label, Randomised, Phase 2 Trial." The Lancet Oncology, December. https://doi.org/10.1016/S1470-2045(19)30684-9.

Mateo, Joaquin, Suzanne Carreira, Shahneen Sandhu, Susana Miranda, Helen Mossop, Raquel Perez-Lopez, Daniel Nava Rodrigues, et al. 2015. "DNA-Repair Defects and Olaparib in Metastatic Prostate Cancer." The New England Journal of Medicine 373 (18): 1697–1708.

Maughan, Benjamin L., Liana B. Guedes, Kenneth Boucher, Gaurav Rajoria, Zach Liu, Szczepan Klimek, Roberto Zoino, Emmanuel S. Antonarakis, and Tamara L. Lotan. 2018. “p53 Status in the Primary Tumor Predicts Efficacy of Subsequent Abiraterone and Enzalutamide in Castration-Resistant Prostate Cancer.” Prostate Cancer and Prostatic Diseases, January. https://doi.org/10.1038/s41391-017-0027-4.

Mayrhofer, Markus, Bram De Laere, Tom Whitington, Peter Van Oyen, Christophe Ghysel, Jozef Ampe, Piet Ost, et al. 2018. “Cell-Free DNA Profiling of Metastatic Prostate Cancer Reveals Microsatellite Instability, Structural Rearrangements and Clonal Hematopoiesis.” Genome Medicine 10 (1): 85.

McGuire, W. P., W. J. Hoskins, M. F. Brady, P. R. Kucera, E. E. Partridge, K. Y. Look, D. L. Clarke-Pearson, and M. Davidson. 1996. “Cyclophosphamide and Cisplatin Compared with Paclitaxel and Cisplatin in Patients with Stage III and Stage IV Ovarian Cancer.” The New England Journal of Medicine 334 (1): 1–6.

McKiernan, James, Michael J. Donovan, Vince O’Neill, Stefan Bentink, Mikkel Noerholm, Susan Belzer, Johan Skog, et al. 2016. “A Novel Urine Exosome Gene Expression Assay to Predict High-Grade Prostate Cancer at Initial Biopsy.” JAMA Oncology 2 (7): 882–89.

Miyamoto, David T., Richard J. Lee, Mark Kalinich, Joseph A. LiCausi, Yu Zheng, Tianqi Chen, John D. Milner, et al. 2018. “An RNA-Based Digital Circulating Tumor Cell Signature Is Predictive of Drug Response and Early Dissemination in Prostate Cancer.” Cancer Discovery. https://doi.org/10.1158/2159-8290.cd-16-1406.

Miyamoto, David T., Yu Zheng, Ben S. Wittner, Richard J. Lee, Huili Zhu, Katherine T. Broderick, Rushil Desai, et al. 2015. “RNA-Seq of Single Prostate CTCs Implicates Noncanonical Wnt Signaling in Antiandrogen Resistance.” Science 349 (6254): 1351–56.

Morris, Michael J., R. Bryan Rumble, Ethan Basch, Sebastien J. Hotte, Andrew Loblaw, Dana Rathkopf, Paul Celano, Rick Bangs, and Matthew I. Milowsky. 2018. “Optimizing Anticancer Therapy in Metastatic Non-Castrate Prostate Cancer: American Society of Clinical Oncology Clinical Practice Guideline.” Journal of Clinical Oncology. https://doi.org/10.1200/jco.2018.78.0619.

Mota, Jose Mauricio, Ethan Barnett, Jones T. Nauseef, Bastien Nguyen, Konrad H. Stopsack, Andreas Wibmer, Jessica R. Flynn, et al. 2020. “Platinum-Based Chemotherapy in Metastatic Prostate Cancer With DNA Repair Gene Alterations.” JCO Precision Oncology, no. 4 (May): 355–66.

Möhrmann, Lino, Helen J. Huang, David S. Hong, Apostolia M. Tsimberidou, Siqing Fu, Sarina A. Piha-Paul, Vivek Subbiah, et al. 2018. “Liquid Biopsies Using Plasma Exosomal Nucleic Acids and Plasma Cell-Free DNA Compared with Clinical Outcomes of Patients with Advanced Cancers.” Clinical Cancer Research: An Official Journal of the American Association for Cancer Research 24 (1): 181–88.

Navin, Nicholas E. 2014. “Cancer Genomics: One Cell at a Time.” Genome Biology 15 (8): 452.

OncologyPRO. n.d. “IPATential150: Phase III Study of Ipatasertib (ipat) plus Abiraterone (abi) vs Placebo (pbo) plus Abi in Metastatic Castration-Resistant Prostate c.” Accessed October 7, 2020a. https://oncologypro.esmo.org/meeting-resources/esmo-virtual-congress-2020/ipatential150-phase-iii-study-of-ipatasertib-ipat-plus-abiraterone-abi-vs-placebo-pbo-plus-abi-in-metastatic-castration-resistant-prostate-c.

OncologyPRO. n.d. “Pre-Specified Interim Analysis of GALAHAD: A Phase 2 Study of Niraparib in Patients (pts) with Metastatic Castration-Resistant Prostate Cancer (mCR... | OncologyPRO.” Accessed December 19, 2019a. https://oncologypro.esmo.org/Meeting-Resources/ESMO-2019-Congress/Pre-specified-interim-analysis-of-GAL

AHAD-A-phase-2-study-of-niraparib-in-patients-pts-with-metastatic-castration-resistant-prostate-cancer-mCRPC-and-biallelic-DNA-repair-gene-defects-DRD.

Park, Kyung, Scott A. Tomlins, Kumaran M. Mudaliar, Ya-Lin Chiu, Raquel Esgueva, Rohit Mehra, Khalid Suleman, et al. 2010. “Antibody-Based Detection of ERG Rearrangement-Positive Prostate Cancer.” Neoplasia 12 (7): 590–98.

Parker, C., S. Nilsson, D. Heinrich, S. I. Helle, J. M. O’Sullivan, S. D. Fosså, A. Chodacki, et al. 2013. “Alpha Emitter Radium-223 and Survival in Metastatic Prostate Cancer.” The New England Journal of Medicine 369 (3): 213–23.

Parker, Christopher C., Nicholas D. James, Christopher D. Brawley, Noel W. Clarke, Alex P. Hoyle, Adnan Ali, Alastair W. S. Ritchie, et al. 2018. “Radiotherapy to the Primary Tumour for Newly Diagnosed, Metastatic Prostate Cancer (STAMPEDE): A Randomised Controlled Phase 3 Trial.” The Lancet 392 (10162): 2353–66.

Pennington, Kathryn P., Tom Walsh, Maria I. Harrell, Ming K. Lee, Christopher C. Pennil, Mara H. Rendi, Anne Thornton, et al. 2014. “Germline and Somatic Mutations in Homologous Recombination Genes Predict Platinum Response and Survival in Ovarian, Fallopian Tube, and Peritoneal Carcinomas.” Clinical Cancer Research: An Official Journal of the American Association for Cancer Research 20 (3): 764–75.

Plimack, Elizabeth R., Roland L. Dunbrack, Timothy A. Brennan, Mark D. Andrake, Yan Zhou, Ilya G. Serebriiskii, Michael Slifker, et al. 2015. “Defects in DNA Repair Genes Predict Response to Neoadjuvant Cisplatin-Based Chemotherapy in Muscle-Invasive Bladder Cancer.” European Urology 68 (6): 959–67.

Plymate, Stephen R., Adam Sharp, and Johann S. de Bono. 2018. “Nuclear Circulating Tumor Cell Androgen Receptor Variant 7 in Castration-Resistant Prostate Cancer: The Devil Is in the Detail.” JAMA Oncology.

Polak, Paz, Jaegil Kim, Lior Z. Braunstein, Rosa Karlic, Nicholas J. Haradhavala, Grace Tiao, Daniel Rosebrock, et al. 2017. “A Mutational Signature Reveals Alterations Underlying Deficient Homologous Recombination Repair in Breast Cancer.” Nature Genetics 49 (10): 1476–86.

Pomerantz, Mark M., Sandor Spisák, Li Jia, Angel M. Cronin, Istvan Csabai, Elisa Ledet, A. Oliver Sartor, et al. 2017. “The Association between Germline BRCA2 Variants and Sensitivity to Platinum-Based Chemotherapy among Men with Metastatic Prostate Cancer.” Cancer 123 (18): 3532–39.

Quigley, David, Joshi J. Alumkal, Alexander W. Wyatt, Vishal Kothari, Adam Foye, Paul Lloyd, Rahul Aggarwal, et al. 2017. “Analysis of Circulating Cell-Free DNA Identifies Multiclonal Heterogeneity of BRCA2 Reversion Mutations Associated with Resistance to PARP Inhibitors.” Cancer Discovery. https://doi.org/10.1158/2159-8290.cd-17-0146.

Rajpar, Shanna, Alexandra Carmel, Zahira Merabet, Philippe Vielh, Stã©phanie Foulon, Francois Lesaunier, Remy Delva, et al. 2017. “The Benefit of Combining Docetaxel to Androgen Deprivation Therapy in Localized and Metastatic Castration-Sensitive Prostate Cancer as Predicted by ERG Status: An Analysis of Two GETUG Phase III Trials.” Journal of Clinical Orthodontics: JCO 35 (15_suppl): 5012–5012.

Rathkopf, Dana E., Tomasz M. Beer, Yohann Loriot, Celestia S. Higano, Andrew J. Armstrong, Cora N. Sternberg, Johann S. de Bono, et al. 2018. “Radiographic Progression-Free Survival as a Clinically Meaningful End Point in Metastatic Castration-Resistant Prostate Cancer: The PREVAIL Randomized Clinical Trial.” JAMA Oncology 4 (5): 694–701.

Razavi, Pedram, Bob T. Li, David N. Brown, Byoungsok Jung, Earl Hubbell, Ronglai Shen, Wassim Abida, et al. 2019. "High-Intensity Sequencing Reveals the Sources of Plasma Circulating Cell-Free DNA Variants." Nature Medicine 25 (12): 1928–37.

Rescigno, Pasquale, David Lorente, David Dolling, Roberta Ferraldeschi, Daniel Nava Rodrigues, Ruth Riisnaes, Susana Miranda, et al. 2018. "Docetaxel Treatment in PTEN- and ERG-Aberrant Metastatic Prostate Cancers." European Urology Oncology 1 (1): 71–77.

Robinson, Dan, Eliezer M. Van Allen, Yi-Mi Wu, Nikolaus Schultz, Robert J. Lonigro, Juan-Miguel Mosquera, Bruce Montgomery, et al. 2015. "Integrative Clinical Genomics of Advanced Prostate Cancer." Cell 161 (5): 1215–28.

Romanel, Alessandro, Delila Gasi Tandefelt, Vincenza Conteduca, Anuradha Jayaram, Nicola Casiraghi, Daniel Wetterskog, Samanta Salvi, et al. 2015. "Plasma AR and Abiraterone-Resistant Prostate Cancer." Science Translational Medicine 7 (312): 312re10.

Ross, Robert W., Tomasz M. Beer, Susanna Jacobus, Glenn J. Bubley, Mary-Ellen Taplin, Christopher W. Ryan, Jiaoti Huang, William K. Oh, and Prostate Cancer Clinical Trials Consortium. 2008. "A Phase 2 Study of Carboplatin plus Docetaxel in Men with Metastatic Hormone-Refractory Prostate Cancer Who Are Refractory to Docetaxel." Cancer 112 (3): 521–26.

Rubin, Mark A., Christopher A. Maher, and Arul M. Chinnaiyan. 2011. "Common Gene Rearrangements in Prostate Cancer." Journal of Clinical Oncology: Official Journal of the American Society of Clinical Oncology 29 (27): 3659–68.

Ryan, Charles J., Matthew R. Smith, Johann S. de Bono, Arturo Molina, Christopher J. Logothetis, Paul de Souza, Karim Fizazi, et al. 2013. "Abiraterone in Metastatic Prostate Cancer without Previous Chemotherapy." The New England Journal of Medicine 368 (2): 138–48.

Ryan, Charles J., Matthew R. Smith, Karim Fizazi, Fred Saad, Peter F. A. Mulders, Cora N. Sternberg, Kurt Miller, et al. 2015. "Abiraterone Acetate plus Prednisone versus Placebo plus Prednisone in Chemotherapy-Naive Men with Metastatic Castration-Resistant Prostate Cancer (COU-AA-302): Final Overall Survival Analysis of a Randomised, Double-Blind, Placebo-Controlled Phase 3 Study." The Lancet Oncology 16 (2): 152–60.

Sailer, Verena, Marc H. Schiffman, Myriam Kossai, Joanna Cyrta, Shaham Beg, Brian Sullivan, Bradley B. Pua, et al. 2017. "Bone Biopsy Protocol for Advanced Prostate Cancer in the Era of Precision Medicine." Cancer, December. https://doi.org/10.1002/cncr.31173.

Scher, Howard I., Karim Fizazi, Fred Saad, Mary-Ellen Taplin, Cora N. Sternberg, Kurt Miller, Ronald de Wit, et al. 2012. "Increased Survival with Enzalutamide in Prostate Cancer after Chemotherapy." The New England Journal of Medicine 367 (13): 1187–97.

Schiewer, M. J., J. F. Goodwin, S. Han, J. C. Brenner, M. A. Augello, J. L. Dean, F. Liu, et al. 2012. "Dual Roles of PARP-1 Promote Cancer Growth and Progression." Cancer Discovery. https://doi.org/10.1158/2159-8290.cd-12-0120.

Shen, Shu Yi, Rajat Singhania, Gordon Fehringer, Ankur Chakravarthy, Michael H. A. Roehrl, Dianne Chadwick, Philip C. Zuzarte, et al. 2018. "Sensitive Tumour Detection and Classification Using Plasma Cell-Free DNA Methylomes." Nature 563 (7732): 579–83.

Singhal, Udit, Yugang Wang, James Henderson, Yashar S. Niknafs, Yuanyuan Qiao, Amy Gursky, Alexander Zaslavsky, et al. 2018. “Multigene Profiling of CTCs in mCRPC Identifies a Clinically Relevant Prognostic Signature.” Molecular Cancer Research. https://doi.org/10.1158/1541-7786.mcr-17-0539.

Smith, M. R., S. K. Sandhu, W. K. Kelly, H. I. Scher, E. Efstathiou, P. N. Lara, E. Y. Yu, et al. 2019. “LBA50Pre-Specified Interim Analysis of GALAHAD: A Phase II Study of Niraparib in Patients (pts) with Metastatic Castration-Resistant Prostate Cancer (mCRPC) and Biallelic DNA-Repair Gene Defects (DRD).” Annals of Oncology: Official Journal of the European Society for Medical Oncology / ESMO 30 (Supplement_5). https://doi.org/10.1093/annonc/mdz394.043.

Smith, Matthew R., Fred Saad, Dana E. Rathkopf, Peter F. A. Mulders, Johann S. de Bono, Eric J. Small, Neal D. Shore, et al. 2017. “Clinical Outcomes from Androgen Signaling–directed Therapy after Treatment with Abiraterone Acetate and Prednisone in Patients with Metastatic Castration-Resistant Prostate Cancer: Post Hoc Analysis of COU-AA-302.” European Urology. https://doi.org/10.1016/j.eururo.2017.03.007.

Smith, Matthew R., Fred Saad, Simon Chowdhury, Stéphane Oudard, Boris A. Hadaschik, Julie N. Graff, David Olmos, et al. 2018. “Apalutamide Treatment and Metastasis-Free Survival in Prostate Cancer.” The New England Journal of Medicine, February. https://doi.org/10.1056/NEJMoa1715546.

Soest, R. J. van, M. E. van Royen, E. S. de Morrée, J. M. Moll, W. Teubel, E. A. C. Wiemer, R. H. J. Mathijssen, R. de Wit, and W. M. van Weerden. 2013. “Cross-Resistance between Taxanes and New Hormonal Agents Abiraterone and Enzalutamide May Affect Drug Sequence Choices in Metastatic Castration-Resistant Prostate Cancer.” European Journal of Cancer. https://doi.org/10.1016/j.ejca.2013.09.026.

Sonnenblick, Amir, Evandro de Azambuja, Hatem A. Azim, and Martine Piccart. 2015. “An Update on PARP Inhibitors—moving to the Adjuvant Setting.” Nature Reviews Clinical Oncology. https://doi.org/10.1038/nrclinonc.2014.163.

Spritzer, Charles E., P. Diana Afonso, Emily N. Vinson, James D. Turnbull, Karla K. Morris, Adam Foye, John F. Madden, Kingshuk Roy Choudhury, Phillip G. Febbo, and Daniel J. George. 2013. “Bone Marrow Biopsy: RNA Isolation with Expression Profiling in Men with Metastatic Castration-Resistant Prostate Cancer—Factors Affecting Diagnostic Success.” Radiology 269 (3): 816–23.

Steinestel, Julie, Christof Bernemann, Andres J. Schrader, and Jochen K. Lennerz. 2017. “Re: Emmanuel S. Antonarakis, Changxue Lu, Brandon Luber, et Al. Clinical Significance of Androgen Receptor Splice Variant-7 mRNA Detection in Circulating Tumor Cells of Men with Metastatic Castration-Resistant Prostate Cancer Treated with First- and Second-Line Abiraterone and Enzalutamide. J Clin Oncol 2017;35:2149-56: AR-V7 Testing: What’s in It for the Patient?” European Urology 72 (6): e168–69.

Sternberg, Cora N., Daniel P. Petrylak, Oliver Sartor, J. Alfred Witjes, Tomasz Demkow, Jean-Marc Ferrero, Jean-Christophe Eymard, et al. 2009. “Multinational, Double-Blind, Phase III Study of Prednisone and Either Satraplatin or Placebo in Patients with Castrate-Refractory Prostate Cancer Progressing after Prior Chemotherapy: The SPARC Trial.” Journal of Clinical Oncology: Official Journal of the American Society of Clinical Oncology 27 (32): 5431–38.

Stopsack, Konrad H., Subhiksha Nandakumar, Andreas G. Wibmer, Samuel Haywood, Emily S. Weg, Ethan S. Barnett, Chloe J. Kim, et al. 2020. “Oncogenic Genomic Alterations, Clinical Phenotypes, and Outcomes in Metastatic Castration-Sensitive Prostate Cancer.” Clinical Cancer Research: An Official Journal of the American Association for Cancer Research, March. https://doi.org/10.1158/1078-0432.CCR-20-0168.

Sweeney, Christopher J., Yu-Hui Chen, Michael Carducci, Glenn Liu, David F. Jarrard, Mario Eisenberger, Yu-Ning Wong, et al. 2015. “Chemohormonal Therapy in Metastatic Hormone-Sensitive Prostate Cancer.” The New England Journal of Medicine 373 (8): 737–46.

Swennenhuis, Joost F., Arjan G. J. Tibbe, Rianne Levink, Ronald C. J. Sipkema, and Leon W. M. M. Terstappen. 2009. “Characterization of Circulating Tumor Cells by Fluorescence in Situ Hybridization.” Cytometry. Part A: The Journal of the International Society for Analytical Cytology 75 (6): 520–27.

Sydes, M. R., M. R. Spears, M. D. Mason, N. W. Clarke, D. P. Dearnaley, J. S. de Bono, G. Attard, et al. 2018. “Adding Abiraterone or Docetaxel to Long-Term Hormone Therapy for Prostate Cancer: Directly Randomised Data from the STAMPEDE Multi-Arm, Multi-Stage Platform Protocol.” Annals of Oncology: Official Journal of the European Society for Medical Oncology / ESMO 29 (5): 1235–48.

Tutt, Andrew, Holly Tovey, Maggie Chon U. Cheang, Sarah Kernaghan, Lucy Kilburn, Patrycja Gazinska, Julie Owen, et al. 2018. “Carboplatin in BRCA1/2-Mutated and Triple-Negative Breast Cancer BRCAness Subgroups: The TNT Trial.” Nature Medicine 24 (5): 628–37.

Van Allen, E. M., K. W. Mouw, P. Kim, G. Iyer, N. Wagle, H. Al-Ahmadie, C. Zhu, et al. 2014. “Somatic ERCC2 Mutations Correlate with Cisplatin Sensitivity in Muscle-Invasive Urothelial Carcinoma.” Cancer Discovery. https://doi.org/10.1158/2159-8290.cd-14-0623.

Wallis, Christopher J. D., Zachary Klaassen, Bimal Bhindi, Hanan Goldberg, Thenappan Chandrasekar, Ann M. Farrell, Stephen A. Boorjian, Girish S. Kulkarni, Robert Jeffrey Karnes, and Raj Satkunasivam. 2018. “Comparison of Abiraterone Acetate and Docetaxel with Androgen Deprivation Therapy in High-Risk and Metastatic Hormone-Naïve Prostate Cancer: A Systematic Review and Network Meta-Analysis.” European Urology 73 (6): 834–44.

Wang, L., S. M. Dehm, D. W. Hillman, H. Sicotte, W. Tan, M. Gormley, V. Bhargava, et al. 2017. “A Prospective Genome-Wide Study of Prostate Cancer Metastases Reveals Association of Wnt Pathway Activation and Increased Cell Cycle Proliferation with Primary Resistance to Abiraterone Acetate-Prednisone.” Annals of Oncology: Official Journal of the European Society for Medical Oncology / ESMO, October. https://doi.org/10.1093/annonc/mdx689.

Willis, Jason, Martina I. Lefterova, Alexander Artyomenko, Pashtoon Murtaza Kasi, Yoshiaki Nakamura, Kabir Mody, Daniel V. T. Catenacci, et al. 2019. “Validation of Microsatellite Instability Detection Using a Comprehensive Plasma-Based Genotyping Panel.” Clinical Cancer Research: An Official Journal of the American Association for Cancer Research.

Wit, Ronald de, Johann de Bono, Cora N. Sternberg, Karim Fizazi, Bertrand Tombal, Christian Wülfing, Gero Kramer, et al. 2019. “Cabazitaxel versus Abiraterone or Enzalutamide in Metastatic Prostate Cancer.” The New England Journal of Medicine 381 (26): 2506–18.

Wu, Yi-Mi, Marcin Cieślik, Robert J. Lonigro, Pankaj Vats, Melissa A. Reimers, Xuhong Cao, Yu Ning, et al. 2018. “Inactivation of CDK12 Delineates a Distinct Immunogenic Class of Advanced Prostate Cancer.” Cell 173 (7): 1770–82.e14.

Wyatt, Alexander W., Arun A. Azad, Stanislav V. Volik, Matti Annala, Kevin Beja, Brian McConeghy, Anne Haegert, et al. 2016. “Genomic Alterations in Cell-Free DNA and Enzalutamide Resistance in Castration-Resistant Prostate Cancer.” JAMA Oncology 2 (12): 1598–1606.

Wyatt, Alexander W., Matti Annala, Rahul Aggarwal, Kevin Beja, Felix Feng, Jack Youngren, Adam Foye, et al. 2017. “Concordance of Circulating Tumor DNA and Matched Metastatic Tissue Biopsy in Prostate Cancer.” Journal of the National Cancer Institute 109 (12). https://doi.org/10.1093/jnci/djx118.

Xi, Liqiang, Trinh Hoc-Tran Pham, Eden C. Payabyab, Richard M. Sherry, Steven A. Rosenberg, and Mark Raffeld. 2016. “Circulating Tumor DNA as an Early Indicator of Response to T-Cell Transfer Immunotherapy in Metastatic Melanoma.” Clinical Cancer Research: An Official Journal of the American Association for Cancer Research 22 (22): 5480–86.

Xia, Yun, Chiang-Ching Huang, Rachel Dittmar, Meijun Du, Yuan Wang, Hongyan Liu, Niraj Shenoy, Liang Wang, and Manish Kohli. 2016. “Copy Number Variations in Urine Cell Free DNA as Biomarkers in Advanced Prostate Cancer.” Oncotarget. https://doi.org/10.18632/oncotarget.9027.

Zafeiriou, Zafeiris, Diletta Bianchini, Robert Chandler, Pasquale Rescigno, Wei Yuan, Suzanne Carreira, Maialen Barrero, et al. 2019. “Genomic Analysis of Three Metastatic Prostate Cancer Patients with Exceptional Responses to Carboplatin Indicating Different Types of DNA Repair Deficiency.” European Urology. https://doi.org/10.1016/j.eururo.2018.09.048.

Zehir, Ahmet, Ryma Benayed, Ronak H. Shah, Aijazuddin Syed, Sumit Middha, Hyunjae R. Kim, Preethi Srinivasan, et al. 2017. “Mutational Landscape of Metastatic Cancer Revealed from Prospective Clinical Sequencing of 10,000 Patients.” Nature Medicine 23 (6): 703–13.
