# Supplementary material for: Androgen receptor pathway inhibitors and taxanes in metastatic prostate cancer: an outcome-adaptive randomized platform trial
Source: Nat Med. 2024 Aug 20;30(11):3291–302. doi: 10.1038/s41591-024-03204-2 (PMC11564108; doi:10.1038/s41591-024-03204-2)
Supplement: Supplementary file 2 — Reporting Summary [file 41591_2024_3204_MOESM2_ESM.pdf]

nature portfolio

Corresponding author(s): Henrik Grönberg
Last updated by author(s): Jul 10, 2024

Reporting Summary

Nature Portfolio wishes to improve the reproducibility of the work that we publish. This form provides structure for consistency and transparency in reporting. For further information on Nature Portfolio policies, see our Editorial Policies and the Editorial Policy Checklist.

Statistics

For all statistical analyses, confirm that the following items are present in the figure legend, table legend, main text, or Methods section.

n/a	Confirmed	
☐	☒	The exact sample size (*n*) for each experimental group/condition, given as a discrete number and unit of measurement
☐	☒	A statement on whether measurements were taken from distinct samples or whether the same sample was measured repeatedly
☐	☒	The statistical test(s) used AND whether they are one- or two-sided<br>*Only common tests should be described solely by name; describe more complex techniques in the Methods section.*
☐	☒	A description of all covariates tested
☐	☒	A description of any assumptions or corrections, such as tests of normality and adjustment for multiple comparisons
☐	☒	A full description of the statistical parameters including central tendency (e.g. means) or other basic estimates (e.g. regression coefficient) AND variation (e.g. standard deviation) or associated estimates of uncertainty (e.g. confidence intervals)
☐	☒	For null hypothesis testing, the test statistic (e.g. *F*, *t*, *r*) with confidence intervals, effect sizes, degrees of freedom and *P* value noted<br>*Give P values as exact values whenever suitable.*
☐	☒	For Bayesian analysis, information on the choice of priors and Markov chain Monte Carlo settings
☐	☒	For hierarchical and complex designs, identification of the appropriate level for tests and full reporting of outcomes
☐	☒	Estimates of effect sizes (e.g. Cohen's *d*, Pearson's *r*), indicating how they were calculated

Our web collection on statistics for biologists contains articles on many of the points above.

Software and code

Policy information about availability of computer code

Data collection	eCRF clinical data collection (SMART-TRIAL, Greenlight Guru, Denmark)

Data analysis	DNA sequencing data analysis and computational genomics was performed using the following tools:<br>BWA MEM (v.0.7.17-r1188)<br>GATK (v4.1.2.0)<br>Mutect2 (v4.1.2.0)<br>Strelka (v2.9.10)<br>VarDict (v1.8.3)<br>VarScan2 (v2.4.4)<br>SomaticSeq (v3.7.3)<br>Ensembl Variant Effect Predictor (v110.1)<br>HaplotypeCaller (v1.2.0)<br>SVcaller (v0.1)<br>CNVkit (v0.9.11)<br>Jumble (v0.1, https://github.com/ClinSeq/jumble)<br>PureCN (v1.12.2)<br>IGV (v2.16.2)<br><br>Data and statistical analysis was performed in R (version 4.2.2). The code for the statistical analysis is available via a github repository https://github.com/alecri/arpi_all.

For manuscripts utilizing custom algorithms or software that are central to the research but not yet described in published literature, software must be made available to editors and reviewers. We strongly encourage code deposition in a community repository (e.g. GitHub). See the Nature Portfolio guidelines for submitting code & software for further information.

Data

Policy information about availability of data

All manuscripts must include a data availability statement. This statement should provide the following information, where applicable:

- Accession codes, unique identifiers, or web links for publicly available datasets
- A description of any restrictions on data availability
- For clinical datasets or third party data, please ensure that the statement adheres to our policy

All data relevant for the interpretation of our findings reported in this manuscript areis provided in the main manuscript or the supplemental information. The data supporting the findings of this trial can be accessed under the following conditions: Requests for data access should be directed to the corresponding author, Henrik Grönberg, at henrik.gronberg@ki.se. Access to the sequencing data requires approval from the Swedish Ethical Review Authority and an agreement with the data protection and legal unit at Karolinska Institutet. Data providing information on individual outcomes or genotypes is classified as personal registry information under Swedish law (Personal Data Act), thus prohibiting submission to a public repository. The data can be used for retrospective auxiliary research questions upon ethical committee (EC) approval. Researchers must provide a study-specific protocol conforming to local guidelines. Requests will be processed within 3-4 months upon submission of a complete and compliant auxiliary research-specific study protocol. For further details or to initiate a request, please contact Henrik Grönberg, at henrik.gronberg@ki.se.

Human research participants

Policy information about studies involving human research participants and Sex and Gender in Research.

Reporting on sex and gender	Not applicable (cfr. a trial in men with metastatic prostate cancer)
Population characteristics	Patients with progressive metastatic castration-resistant prostate cancer
Recruitment	Patient accrual occurred at 24 sites across three countries (Sweden, Belgium, and Norway). Patient accrual was performed by the treating physician, following the per protocol eligibility criteria. As screening of patients occurred by the treating physican, there was no risk for self-selection bias. Upon assessment of ProBio's in- and exclusion criteria, patient's were asked to participate in the study by the treating physician, upon which the patient provided his informed consent.
Ethics oversight	Ethics boards and regulatory authorities in Sweden, Belgium, Norway, and Switzerland. The trial was approved by ethics boards in Sweden (ID: 2018/2206-32; 22.10.2018), Belgium (ID: BC-06057; 20.03.2020), Norway (ID: Søknadsnummer 81005/58005; 24.06.2020), and Switzerland (ID: BASEC 2021-02495; 01.03.2022).

Note that full information on the approval of the study protocol must also be provided in the manuscript.

Field-specific reporting

Please select the one below that is the best fit for your research. If you are not sure, read the appropriate sections before making your selection.

☒ Life sciences ☐ Behavioural & social sciences ☐ Ecological, evolutionary & environmental sciences

For a reference copy of the document with all sections, see nature.com/documents/nr-reporting-summary-flat.pdf

Life sciences study design

All studies must disclose on these points even when the disclosure is negative.

Sample size	Sample size was derived from extensive simulation studies in the planning phase of the trial (Crippa et al. Trials, 2018)
Data exclusions	No data were excluded
Replication	Replication of the study findings is not applicable within the current report, as the study reports on the results of a randomized controlled clinical trial. Findings may be replicated if one would set-up a new (validation) trial.
Randomization	The absence or presence of the four selected biomarkers (i.e. AR, TP53, homologous recombination deficiency, and TMPRSS2-ERG gene fusion) generates 16 distinct biomarker subgroup combinations. Each patient is exclusively assigned to one subgroup. Randomization is stratified on these subgroups, leading to varying randomization probabilities across different subgroup levels. While early termination is based on the evaluation of therapy classes within biomarker signatures, treatment arms are also compared against the control arm within specific biomarker subgroup combinations. Probabilities of superiority derived in the latter comparisons are utilized to update the initial randomization probabilities in each biomarker subgroup combination. We ensured that the randomization probabilities for the control arm, within each subgroup, are equal to or higher than the maximum randomization probabilities in the experimental arms.<br>Additionally, randomization takes into account prior systemic therapy exposure, and avoids sequencing or rechallenging with second generation AR pathway inhibitors (e.g. no randomization to enzalutamide when the patient was previously treated with abiraterone acetate). However, sequencing with taxanes was allowed (i.e. cabazitaxel after prior therapy with docetaxel). To facilitate this, randomization probabilities for non-permitted treatment sequences were set to zero.
Blinding	Participating physicians, both in the control and investigational treatment arms, are blinded to the biomarker results

Reporting for specific materials, systems and methods

We require information from authors about some types of materials, experimental systems and methods used in many studies. Here, indicate whether each material, system or method listed is relevant to your study. If you are not sure if a list item applies to your research, read the appropriate section before selecting a response.

Materials & experimental systems

n/a	Involved in the study	
☒	☐	Antibodies
☒	☐	Eukaryotic cell lines
☒	☐	Palaeontology and archaeology
☒	☐	Animals and other organisms
☐	☒	Clinical data
☒	☐	Dual use research of concern

Methods

n/a	Involved in the study	
☒	☐	ChIP-seq
☒	☐	Flow cytometry
☒	☐	MRI-based neuroimaging

Clinical data

Policy information about clinical studies

All manuscripts should comply with the ICMJE guidelines for publication of clinical research and a completed CONSORT checklist must be included with all submissions.

Clinical trial registration	NCT03903835
Study protocol	Clinical Study Protocol is provided in the Supplementary Appendix
Data collection	Between February 2019 and November 2022, clinical data was collected from 343 patients
Outcomes	The primary endpoint was the time to no longer clinically benefitting per PCWG3 criteria, defined as the specific reason a therapy was ultimately discontinued, according to the physician's judgment by taking into account the evaluation of PSA, conventional radiology, and the clinical assessment. Overall survival and serious adverse event rates were secondary endpoints. Information regarding all-cause mortality was updated biannually using electronic health records. Details on outcome definitions are provided in the Protocol.
